# Targeting RAC1 reactivates pyroptosis to reverse paclitaxel resistance in ovarian cancer by suppressing P21‐activated kinase 4

**DOI:** 10.1002/mco2.719

**Published:** 2024-09-02

**Authors:** Jiangchun Wu, Yong Wu, Tianyi Zhao, Xiangwei Wang, Qinhao Guo, Simin Wang, Siyu Chen, Xingzhu Ju, Jin Li, Xiaohua Wu, Zhong Zheng

**Affiliations:** ^1^ Department of Gynaecologic Oncology Fudan University Shanghai Cancer Center, Fudan University Shanghai China; ^2^ Department of Oncology, Shanghai Medical College Fudan University Shanghai China; ^3^ Department of Nuclear Medicine Fudan University Shanghai Cancer Center Shanghai China

**Keywords:** ovarian cancer, paclitaxel resistance, pyroptosis, RAC1

## Abstract

Pyroptosis may play an important role in the resistance of ovarian cancer (OC) to chemotherapy. However, the mechanism by which pyroptosis modulation can attenuate chemotherapy resistance has not been comprehensively studied in OC. Here, we demonstrated that RAS‐associated C3 botulinum toxin substrate 1 (RAC1) is highly expressed in OC and is negatively correlated with patient outcomes. Through cell function tests and in vivo tumor formation tests, we found that RAC1 can promote tumor growth by mediating paclitaxel (PTX) resistance. RAC1 can mediate OC progression by inhibiting pyroptosis, as evidenced by high‐throughput automated confocal imaging, the release of lactate dehydrogenase (LDH), the expression of the inflammatory cytokines IL‐1β/IL‐18 and the nucleotide oligomerization domain‐like receptor family pyrin domain‐containing 3 (NLRP3) inflammasome. Mechanically, RNA‐seq, gene set enrichment analysis (GSEA), coimmunoprecipitation (Co‐IP), mass spectrometry (MS), and ubiquitination tests further confirmed that RAC1 inhibits caspase‐1/gasdermin D (GSDMD)‐mediated canonical pyroptosis through the P21‐activated kinase 4 (PAK4)/mitogen‐activated protein kinase (MAPK) pathway, thereby promoting PTX resistance in OC cells. Finally, the whole molecular pathway was verified by the results of in vivo drug combination tests, clinical specimen detection and the prognosis. In summary, our results suggest that the combination of RAC1 inhibitors with PTX can reverse PTX resistance by inducing pyroptosis through the PAK4/MAPK pathway.

## Graphical abstract

Pyroptosis may play an important role in chemotherapy resistance of ovarian cancer. RAS‐associated C3 botulinum toxin substrate 1 (RAC1) is overexpressed in ovarian cancer patients and is negatively associated with patient outcomes. It can drive gasdermin D (GSDMD)‐mediated pyroptosis reversal of paclitaxel resistance through the P21‐activated kinase 4 (PAK4)/mitogen‐activated protein kinase (MAPK) pathway. It was further demonstrated to reverse paclitaxel resistance via PAK4 in animal experiments and clinical samples.

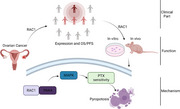

## 1 INTRODUCTION

Ovarian cancer (OC) is the deadliest malignancy of the female reproductive system worldwide.^1^, ^2^ Due to the lack of early screening, 70% of OC patients are diagnosed at an advanced stage.^1^ The current standard treatment for advanced OC is surgery combined with paclitaxel (PTX) and platinum chemotherapy.^3^ However, chemotherapy resistance is an important factor contributing to the high mortality rate of OC.^4^, ^5^ At present, the exact molecular mechanism of OC resistance to chemotherapy drugs is unclear.

PTX is used as a first‐line chemotherapy agent in the treatment of a variety of tumors, including OC, but resistance often limits its clinical use.^6^, ^7^ The mechanism of PTX resistance has not been fully elucidated. Recent studies have shown that it often involves multifactorial processes, including the overexpression of P‐glycoprotein, ATP‐dependent PTX pumping out of cells, the regulation of prosurvival or prodeath regulators, and changes in tubulin or microtubules.^8^, ^9^

Our previous studies suggested that RhoGDI2 (a Ras superfamily member) expression may be mediated by RAS‐related C3 botulinum toxin substrate 1 (RAC1)‐dependent multidrug resistance gene 1 (Mdr‐1).^10^ In 2018, Cardama et al. reviewed the literature on RAC1 and radiotherapy, chemotherapy, and targeted tolerance, suggesting that RAC1 may be an ideal therapeutic target for reversing resistance.^11^ Studies have shown that RAC1 can play a regulatory role by targeting the downstream molecule P21‐activated kinase 4 (PAK4).^12^ Although studies have shown that PAK4 is associated with chemotherapy resistance in a variety of tumors, only one report has shown that PAK4 can inhibit resistance in a three‐dimensional (3D) spheroid model of platinum‐resistant OC, and the specific underlying molecular mechanism is not fully understood.^13^, ^14^, ^15^

Pyroptosis is a novel mechanism that leads to cell death through the activation of multiple inflammasomes, including caspases, resulting in the cleavage and polymerization of members of the gasdermin family.^16^, ^17^ Recent studies have shown that pyroptosis is associated with resistance to multiple chemotherapeutic agents, including PTX.^18^, ^19^ Both cisplatin and PTX can induce caspase‐3/gasdermin E (GSDME)‐dependent pyroptosis in the lung cancer cell line A549 to varying degrees.^20^ However, the specific role and mechanism of pyroptosis in PTX resistance are not clear.

In this study, we demonstrated that RAC1 can mediate PTX resistance to inhibit OC cell death by stabilizing its expression through the targeting of PAK4.

## 2 RESULTS

### 2.1 RAC1 expression is significantly upregulated in patients with OC, and its expression is negatively correlated with patient prognosis

**FIGURE 1:**
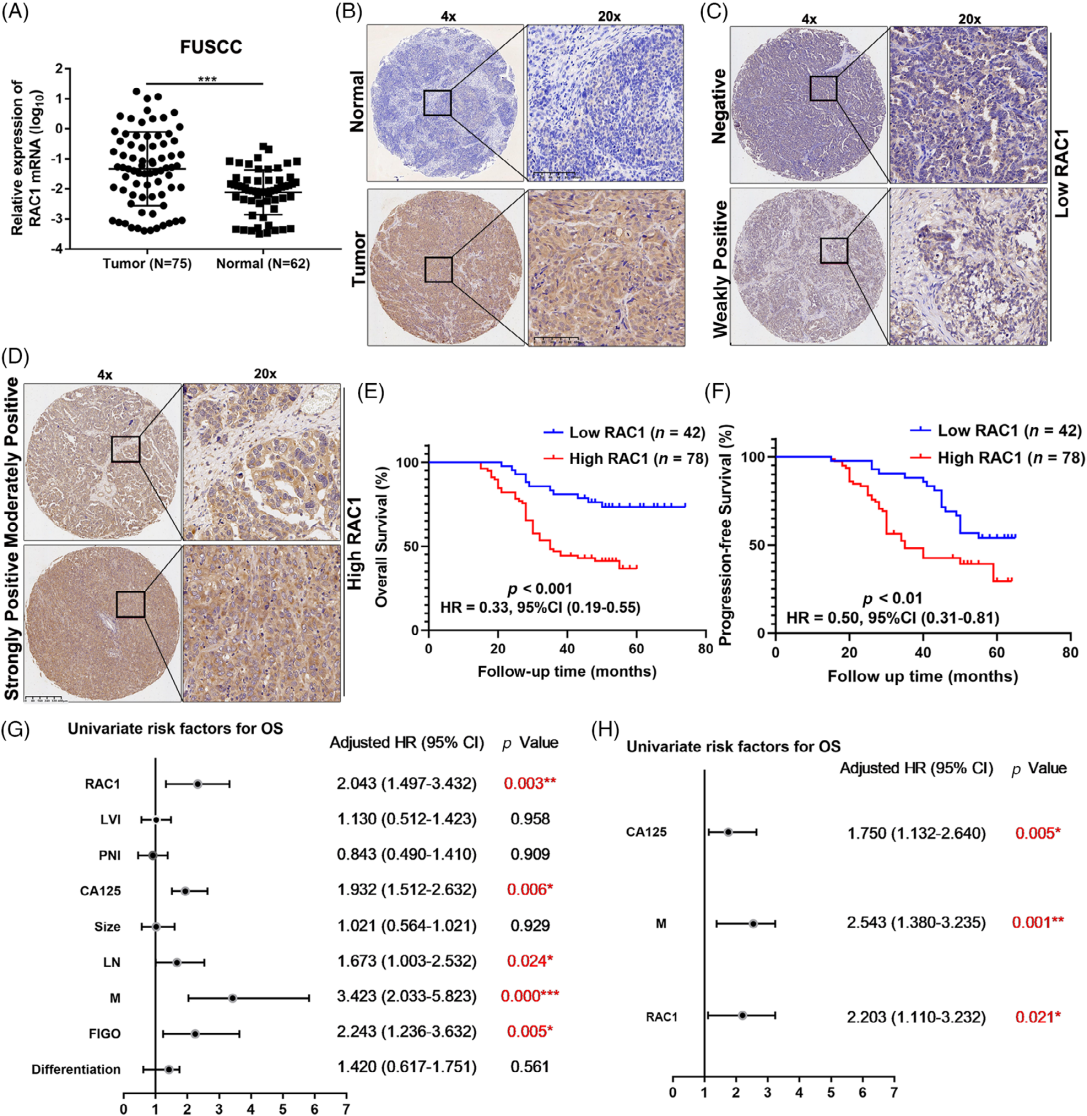
RAS‐associated C3 botulinum toxin substrate 1 (RAC1) is overexpressed in ovarian cancer (OC) tissues from patients and is negatively associated with patient outcomes. (A) According to the analysis of data from our center, RAC1 mRNA expression in OC tissue was significantly higher than that in normal ovarian epithelial tissue (***p < 0.001). (B) Immunohistochemistry (IHC) analysis revealed that RAC1 expression was significantly elevated in OC tissues compared with normal epithelial tissues. (C–F) We divided patients into two groups according to the IHC results for RAC1 expression and found that patients with high RAC1 expression experienced shorter survival (*p*‐ overall survival [OS] < 0.001, hazard ratio [HR] = 0.33, 95% confidence interval [CI] = 0.19–0.55; *p‐progression‐free survival [PFS] < 0.01, HR = 0.50, 95% CI = 0.31–0.81). (G, H) Univariate and multivariate analyses revealed that RAC1 was an independent prognostic factor for OC (the IHC figures on the right represent the partial magnification in the small box on the left (20× vs. 4×); the *p* value in red indicates *p *< 0.05; “ns” indicates no statistically significant difference, *p < 0.05, **p < 0.01, and ***p < 0.001).

First, we detected RAC1 expression at the mRNA and protein levels (immunohistochemistry, IHC) in OC and normal ovarian epithelial samples from our center, and the results revealed that the RAC1 expression level was significantly increased in OC (Figure 1A,B), and the RAC1 mRNA level was consistent with that reported in the Gene Expression Profiling Interactive Analysis (GEPIA) database (Figure S1A).

Furthermore, we divided the IHC results of OC samples into scores of 0, 1, 2, and 3 according to the immunoreactivity score (IRS), among which 0 and 1 were assigned to the low‐expression group, and 2 and 3 were assigned to the high‐expression group (Figure 1C,D).^21^ The results revealed that patients in the high RAC1 expression group had shorter overall survival (OS) and progression‐free survival (PFS; *p*‐OS < 0.001, hazard ratio [HR] = 0.33, 95% confidence interval [CI] = 0.19–0.55; **p‐PFS < 0.01, HR = 0.50, 95% CI = 0.31–0.81; Figure 1E,F). This finding is consistent with the results of the Kaplan–Meier (K–M) plotter (kmplot.com) database (Figure S1B).

Univariate regression analysis revealed that RAC1 expression was significantly associated with poor OS (HR = 2.043, 95% CI = 1.497–3.432, **p < 0.01; Figure 1G). Other variables associated with survival included carbohydrate antigen 125 (CA125) level, lymph node (LN) involvement, distant metastasis (M) status, and International Federation of Gynaecology and Obstetrics (FIGO) stage. Multivariate regression analysis revealed that high RAC1 expression was independently associated with poor OS in OC patients (HR = 2.203, 95% CI = 1.110–3.232, *p < 0.05; Figure 1H). In addition, further analysis of the relationships between RAC1 expression and clinicopathological parameters revealed that RC1 expression was significantly correlated with distant metastasis (**p = 0.025; Table S1), which was consistent with most reported results.

Overall, these data further demonstrate that RAC1 expression is significantly associated with the prognosis of OC patients and can be used as a biomarker for predicting the OC prognosis.

### 2.2 RAC1 promotes OC cell proliferation, cell migration, and invasion in vitro

**FIGURE 2:**
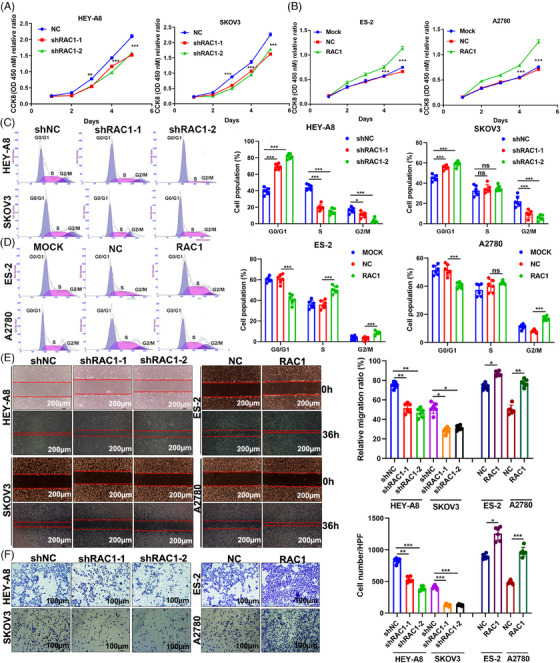
RAS‐associated C3 botulinum toxin substrate 1 (RAC1) promotes ovarian cancer (OC) cell proliferation, migration, and invasion in vitro. (A, B) CCK‐8 and (C, D) cell cycle assays were performed to assess changes in proliferation. (E) Wound healing assays showed that shRNA‐mediated RAC1 knockdown suppressed the migration of HEY‐A8 and SKOV3 cells. However, RAC1 overexpression significantly promoted ES‐2 and A2780 cell migration. (F) Transwell assays revealed that invasion was suppressed when RAC1 was silenced. The ability of RAC1 to promote the invasion of ES‐2 and A2780 cells was confirmed by RAC1 overexpression (“ns” indicates no statistically significant difference, *p < 0.05, **p < 0.01, and ***p < 0.001).

We detected the expression of RAC1 at the protein and mRNA levels in eight OC cell lines (Figure S2A,B). For the highly expressing HEY‐A8 and SKOV3 cell lines, two independent stable RAC1‐expressing cell lines were constructed to effectively reduce RAC1 expression. We constructed stable RAC1‐overexpressing ES‐2 and A2780 cells with low expression (Figure S2C,D). CCK8 and cell cycle assays revealed that RAC1 knockdown significantly inhibited the proliferation of HEYA8 and SKOV3 OC cells and increased the proportion of G0/G1 phase cells (Figure 2A–C). The overexpression of RAC1 significantly promoted the proliferation of ES‐2 and A2780 cells and decreased the proportion of cells in G0/G1 phase (Figure 2B–D). Taken together, these data indicate that RAC1 can promote the proliferation of OC cells.

RAC1 has been reported to be associated with migration, invasion and metastasis in OC.^22^, ^23^ Wound healing and Transwell assays revealed that RAC1 knockdown significantly inhibited the migration and invasion of HEY‐A8 and SKOV OC cells and that overexpression of RAC1 significantly enhanced these phenotypes (Figure 2E,F). In conclusion, these phenotypic results suggest that RAC1 can significantly promote OC cell migration and invasion. In addition, an analysis of clinical data from our center showed that RAC1 is significantly associated with LN metastasis and distant metastasis in OC patients.

### 2.3 The RAC1 inhibitor NSC23766 (NSC) enhances the antitumor effect of PTX in vitro and in vivo

**FIGURE 3:**
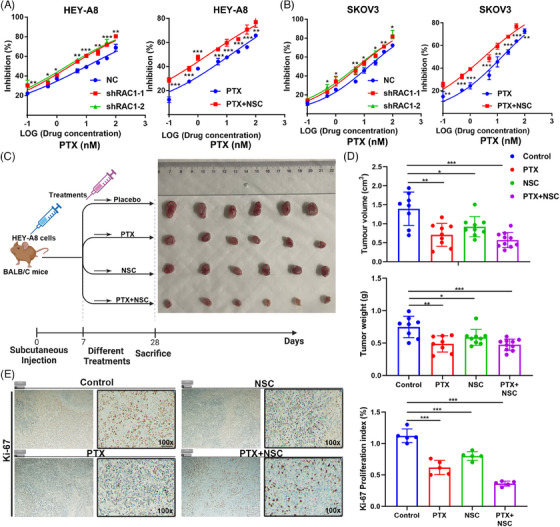
The RAS‐associated C3 botulinum toxin substrate 1 (RAC1) inhibitor NSC enhances the antitumor effect of paclitaxel (PTX) in vitro and in vivo. (A, B) RAC1 knockdown or the administration of the RAC1 inhibitor NSC23766 (NSC) promoted PTX resistance in ovarian cancer (OC) cells. (C) The difference in tumor growth at 1 month after subcutaneous injection of the same number of cells with different treatments; the tumor volume was measured weekly in different groups. (D) Four weeks after subcutaneous injection, the tumor mass differed among the different groups. (E) Immunohistochemistry (IHC) staining showed that compared with the placebo, PTX combined with NSC significantly decreased Ki67 expression (“ns” indicates no statistically significant difference, *p < 0.05, **p < 0.01, and ***p < 0.001).

PTX is the first‐line chemotherapy agent for OC.^23^ We treated the control group and the RAC1‐knockdown group with PTX to explore the effect of RAC1 on the antitumor efficacy of PTX, and the results showed that RAC1 knockdown could significantly enhance the antitumor effect of PTX. Similarly, NSC combined with PTX was compared with PTX alone, and the results showed that NSC could enhance the antitumor effect of PTX (Figure 3A,B). These results demonstrate that RAC1 can promote OC progression by promoting PTX resistance.

Through animal experiments, we found that PTX or NSC alone could significantly inhibit the growth of subcutaneously transplanted tumors and NSC combined with PTX could enhance this inhibitory effect (Figure 3C,D). Further IHC analysis of the tumors revealed that the combined treatment significantly reduced the Ki‐67 expression level (Figure 3E). The above experiments indicate that the RAC1 inhibitor NSC can increase the antitumor effect of PTX in OC.

### 2.4 RAC1 can promote pyroptosis inhibition by mediating PTX resistance

During the treatment of OC, cancer cells gradually undergo apoptosis and are resistant to PTX chemotherapy.^24^ However, whether this resistance mechanism is involved in pyroptosis is unclear.

**FIGURE 4:**
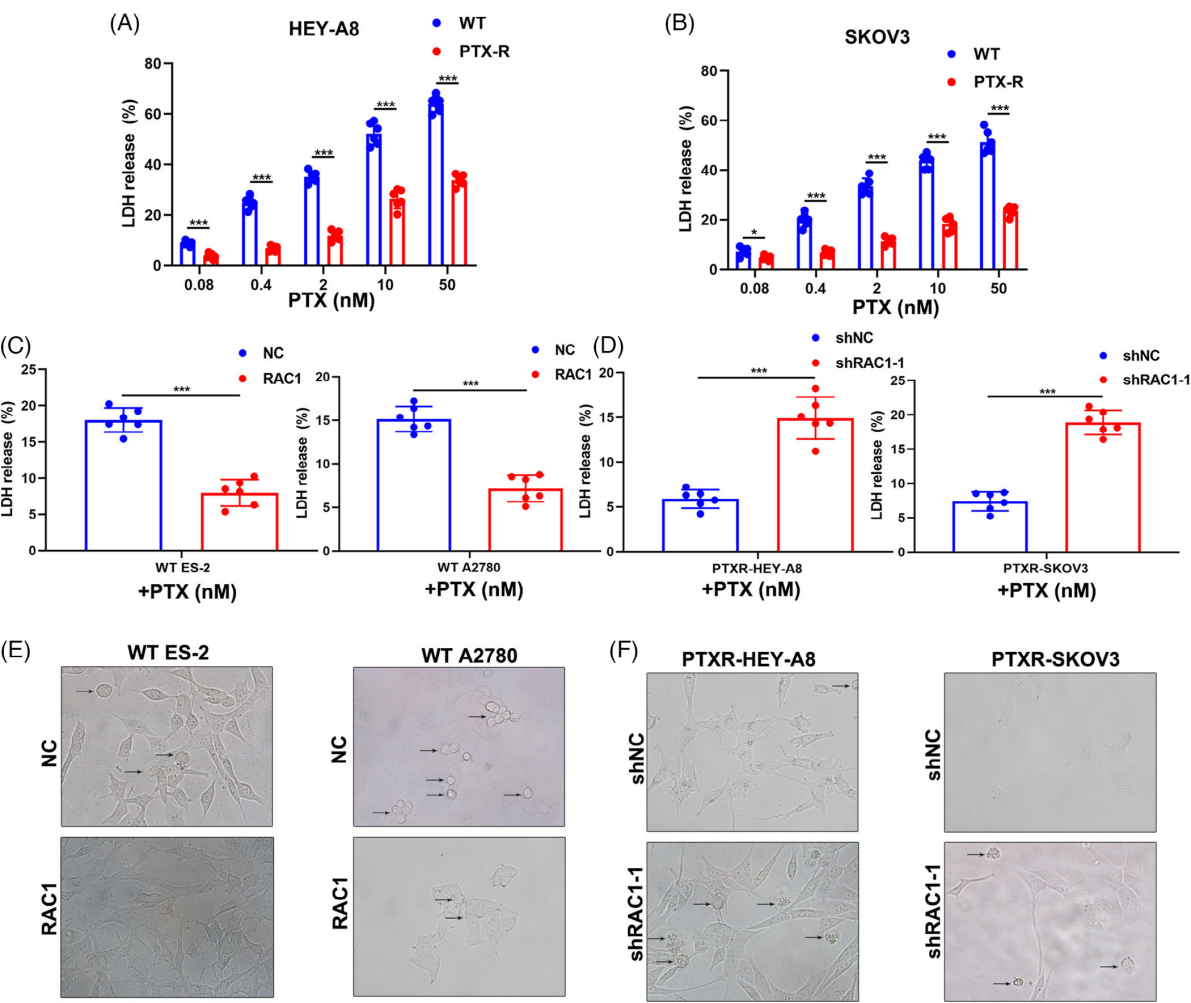
RAS‐associated C3 botulinum toxin substrate 1 (RAC1) can promote pyroptosis inhibition by mediating paclitaxel (PTX) resistance. (A, B) The lactate dehydrogenase (LDH) assay results revealed that high doses of PTX could induce pyroptosis in HEY‐A8 and SKOV3 WT ovarian cancer (OC) cells, whereas PTXR cells did not undergo chemotherapy‐induced pyroptosis in a dose‐dependent manner. (C) Comparison of the amount of LDH released from WT ES2 and A2780 cells after PTX was administered to the NC group and RAC1‐overexpressing group; the results showed that the amount of LDH released from the RAC1‐overexpressing group was significantly lower than that from the control group. (D) LDH release was significantly increased after RAC1 expression was knocked down in PTXR HEY‐A8 and SKOV3 cells. (E) Representative high‐throughput automated confocal images of WT ES‐2 cells and WT A2780 cells after NC treatment or RAC1 overexpression (the arrow points to cells in a pyroptotic state). (F) Representative high‐throughput automated confocal images of PTXR‐HEY‐A8 cells and PTXR‐SKOV3 cells after shNC or RAC1 knockdown (“ns” indicates no statistically significant difference, *p < 0.05, **p < 0.01, and ***p < 0.001).

Lactate dehydrogenase (LDH) assays can be used to detect pyroptosis.^25^ LDH release tests revealed that a high dose of PTX‐induced pyroptosis in the OC cell lines HEY‐A8 and SKOV3 WT in a dose‐dependent manner. However, PTX‐resistant (PTXR) cell lines did not show dose dependence of PTX‐induced pyroptosis (Figure 4A,B). Next, we compared LDH release in WT ES2 and A2780 cells after PTX was administered to the control group and the group transduced with the lentivirus containing the RAC1 overexpression plasmid, and the results indicated that LDH release was distinctly reduced in the RAC1 overexpression group (Figure 4C). The above experiments proved that RAC1 inhibited PTX‐induced LDH release. We knocked down RAC1 in PTXR HEY‐A8 and SKOV3 cells and observed a significant increase in LDH release (Figure 4D). High‐resolution confocal imaging was further applied to analyze pyroptosis in cells subjected to the aforementioned treatments, and the trend of the results was consistent with that of the LDH release experiment (Figure 4E,F).

These findings suggest that knocking down RAC1 can reverse the inhibition of pyroptosis induced by PTX resistance.

### 2.5 Chemotherapy‐induced GSDMD‐mediated OC pyroptosis is driven by RAC1‐controlled caspase‐1‐associated inflammasome activation

**FIGURE 5:**
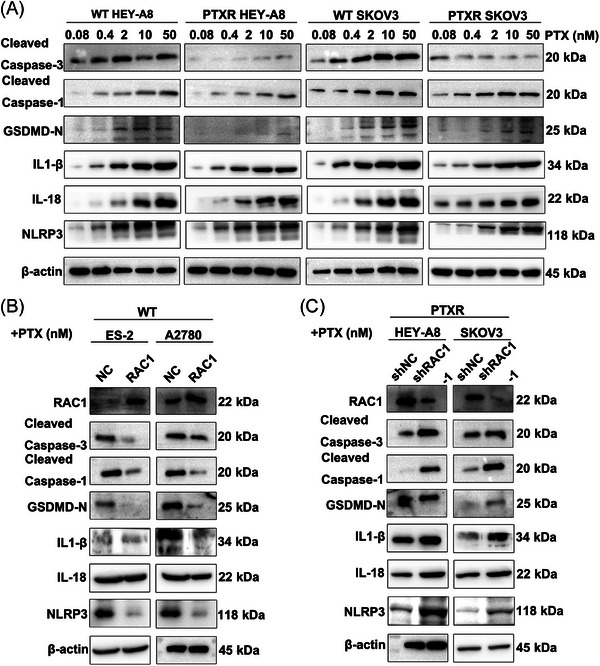
Chemotherapy‐induced caspase‐1 inflammasome activation drives gasdermin D (GSDMD)‐mediated pyroptosis, which is controlled by RAS‐associated C3 botulinum toxin substrate 1 (RAC1), in ovarian cancer (OC). (A) WT HEY‐A8 cells and SKOV3 cells and PTXR HEY‐A8 cells and SKOV3 cells were treated with different concentrations of paclitaxel (PTX) (0.08 nM, 0.4 nM, 2 nM, 10 nM, or 50 nM). A high dose of PTX increased the levels of the pyroptosis‐related molecular markers cleaved caspase‐1, cleaved caspase‐3, and GSDMD‐N, the release of the inflammatory factors IL‐1β/IL‐18, and the level of the nucleotide oligomerization domain‐like receptor family pyrin domain‐containing 3 (NLRP3) inflammasome. The expression of pyroptosis‐related molecular markers and the release of inflammatory factors and inflammasomes in drug‐resistant cells were significantly decreased. (B) In WT ES‐2 and A2780 cells, RAC1 overexpression significantly reduced the levels of cleaved caspase‐1, cleaved caspase‐3, and GSDMD‐N and the release of the inflammatory factors IL‐1β/IL‐18 and the NLRP3 inflammasome after the administration of 10 nM PTX. (C) In PTXR HEY‐A8 and SKOV3 cells, RAC1 knockdown significantly increased the levels of cleaved caspase‐1, cleaved caspase‐3, and GSDMD‐N and the release of the inflammatory factors IL‐1β/IL‐18 and the NLRP3 inflammasome (“ns” indicates no statistically significant difference, *p < 0.05, **p < 0.01, and ***p < 0.001).

WT HEY‐A8 cells and SKOV3 cells and PTXR HEY‐A8 cells and SKOV3 OC cells were treated with different concentrations of PTX (0.08 nM, 0.4 nM, 2 nM, 10 nM, or 50 nM). A high dose of PTX increased the expression levels of the pyroptosis‐related molecular markers cleaved caspase‐3, cleaved caspase‐1, and gasdermin D (GSDMD)‐N. The expression levels of pyroptosis‐related molecular markers and the release of the inflammatory factors IL‐1β/IL‐18 and the nucleotide oligomerization domain‐like receptor family pyrin domain‐containing 3 (NLRP3) inflammasome in PTXR OC cells were significantly decreased (Figure 5A). Therefore, we concluded that high doses of PTX can cause pyroptosis in WT OC cell lines.

In WT ES‐2 and A2780 cells, RAC1 overexpression significantly reduced the levels of cleaved caspase‐1, cleaved caspase‐3, GSDMD‐N, the inflammatory factor IL‐1β/IL‐18 and the NLRP3 inflammasome after the administration of 10 nM PTX (Figure 5B). In PTXR HEY‐A8 and SKOV3 cells, RAC1 knockdown significantly increased the levels of cleaved caspase‐1, cleaved caspase‐3, GSDMD‐N, the inflammatory factor IL‐1β/IL‐18 and the NLRP3 inflammasome (Figure 5C). Therefore, we concluded that RAC1 can mediate pyroptosis in OC cells through GSDMD‐mediated activation of the caspase‐1 inflammasome.

### 2.6 RAC1 drives GSDMD‐mediated pyroptosis in OC through the PAK4/MAPK pathway

**FIGURE 6:**
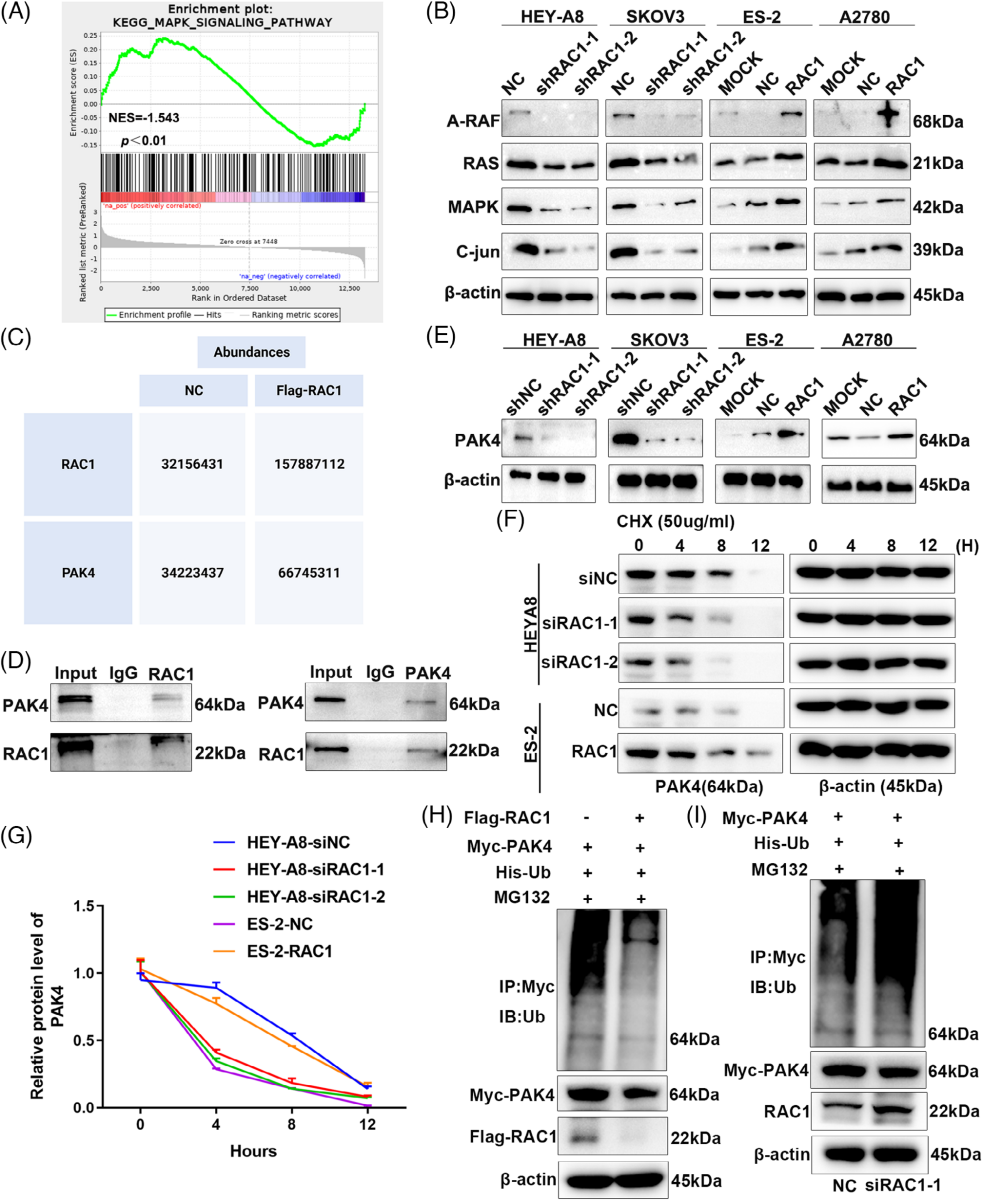
RAS‐associated C3 botulinum toxin substrate 1 (RAC1) drives gasdermin D (GSDMD)‐mediated pyroptosis in ovarian cancer (OC) through the P21‐activated kinase 4 (PAK4)/mitogen‐activated protein kinase (MAPK) pathway. (A) Gene set enrichment analysis (GSEA) revealed that RAC1 knockdown significantly reduced the expression of genes involved in the MAPK pathway. (B) Molecular markers of the MAPK pathway were detected by WB, and the results revealed that knockdown or overexpression of RAC1 significantly suppressed or promoted the expression of A‐RAF, RAS, MAPK, C‐jun, and so forth. (C) We transiently transfected Myc‐RAC1 and NC plasmids into HEK293T cells, subjected protein lysates to coimmunoprecipitation (Co‐IP) and subjected the Co‐IP products to a mass spectrometry (MS) analysis. An analysis of the MS data revealed that PAK4 was more highly expressed in the Myc‐RAC1 group than in the NC group (the data in the table are the expression levels determined by sequencing). (D) HEY‐A8 Co‐IP results showing that RAC1 can bind to PAK4. (E) Knockdown or overexpression of RAC1 suppressed or promoted PAK4 protein expression, respectively. (F, G) After the knockdown or overexpression of RAC1, cyclohexane (CHX) was added to inhibit protein synthesis to observe the degradation of PAK4. The results revealed that the degradation rate of PAK4 was accelerated after the reduction of RAC1 expression (***p < 0.001), whereas the half‐life of PAK4 increased after the overexpression of RAC1 (***p < 0.01). (H) HEK293T cells were transfected with Flag‐RAC1, His‐Ub, and Myc‐PAK4 overexpression plasmids and treated with MG132. The results revealed that the level of ubiquitination in the RAC1‐overexpressing group decreased. (I) We transfected Myc‐PAK4 and His‐Ub overexpression plasmids into HEK293T cells, knocked down RAC1 and treated the cells with MG132. The results showed that the level of ubiquitination in the RAC1‐knockdown group increased, indicating that RAC1 knockdown can promote the ubiquitination of the PAK4 protein (“ns” indicates no statistically significant difference, *p < 0.05, **p < 0.01, and ***p < 0.001).

Transcriptome sequencing and gene set enrichment analysis (GSEA) were performed on the OC cell lines in the HEY‐A8/RAC1‐knockdown group and the NC no‐load group, and the mitogen‐activated protein kinase (MAPK) pathway was highly enriched in the RAC1‐knockdown group (Figure 6A and Table S2). Through further verification of the expression of MAPK pathway‐related molecular markers, RAC1 depletion significantly inhibited the enrichment of the MAPK signaling pathway, whereas RAC1 overexpression significantly promoted the enrichment of the MAPK signaling pathway (Figure 6B).

Studies have shown that RAC1 can target PAK4‐mediated pyroptosis in tumor cells.^12^ After the Myc‐RAC1 or NC plasmid was transfected into HEK293T cells, protein coimmunoprecipitation (Co‐IP) and mass spectrometry (MS) were performed. The results revealed significantly higher expression of PAK4 in the Myc‐RAC1 group than in the NC group (Figure 6C, Tables S3 and S4). Therefore, we can infer that RAC1 can bind to the targeted binding protein PAK4; a positive correlation was observed between the expression levels of the two proteins. We then further verified the forward and reverse Co‐IP results in the OC cell line HEY‐A8 and found that RAC1 could bind the PAK4 protein (Figure 6D). When RAC1 was knocked down or overexpressed, PAK4 protein expression decreased or increased, respectively (Figure 6E).

We added cyclohexane (CHX) to RAC1‐knockdown or RAC1‐overexpressing cell lines, and the proteins were collected at different time points (0, 4, 8, and 12 h) to further explore the mechanism by which RAC1 regulates PAK4.^21^ The experimental results revealed that the degradation rate of the PAK4 protein increased after RAC1 knockdown. The overexpression of RAC1 slowed the degradation rate of the PAK4 protein (Figure 6F,G). These results indicated that RAC1 could inhibit the degradation of the PAK4 protein. The methods of protein degradation vary, but ubiquitination has been reported to be more likely. Subsequent ubiquitination experiments revealed that the ubiquitination level in the RAC1‐overexpressing group decreased, whereas that in the RAC1‐knockdown group increased (Figure 6H,I). Therefore, we concluded that RAC1 can inhibit the ubiquitination and degradation of PAK4.

### 2.7 PAK4 is associated with the patient prognosis

These studies suggest that PAK4 can be positively regulated by RAC1 in vitro. Here, we performed PAK4 IHC staining on the same batch of TMAs and divided the degree of staining into a low RAC1 expression group and a high RAC1 expression group according to the aforementioned IRS scoring method. The results revealed that 71.43% of patients in the low RAC1 expression group had low PAK4 expression. PAK4 was highly expressed in 73.08% of patients in the high RAC1 expression group (Figure S4A,B). The results showed that patients with high PAK4 protein expression had a poor prognosis (***p‐OS < 0.01, HR = 0.48, 95% CI = 0.28–0.80; *p‐PFS < 0.001, HR = 0.32, 95% CI = 0.20–0.53; Figure S4C,D). In conclusion, the above results show that PAK4 is positively correlated with RAC1 expression at the protein level and negatively correlated with the patient prognosis.

### 2.8 RAC1 regulates OC progression by regulating the MAPK signaling pathway through the targeting of PAK4

**FIGURE 7:**
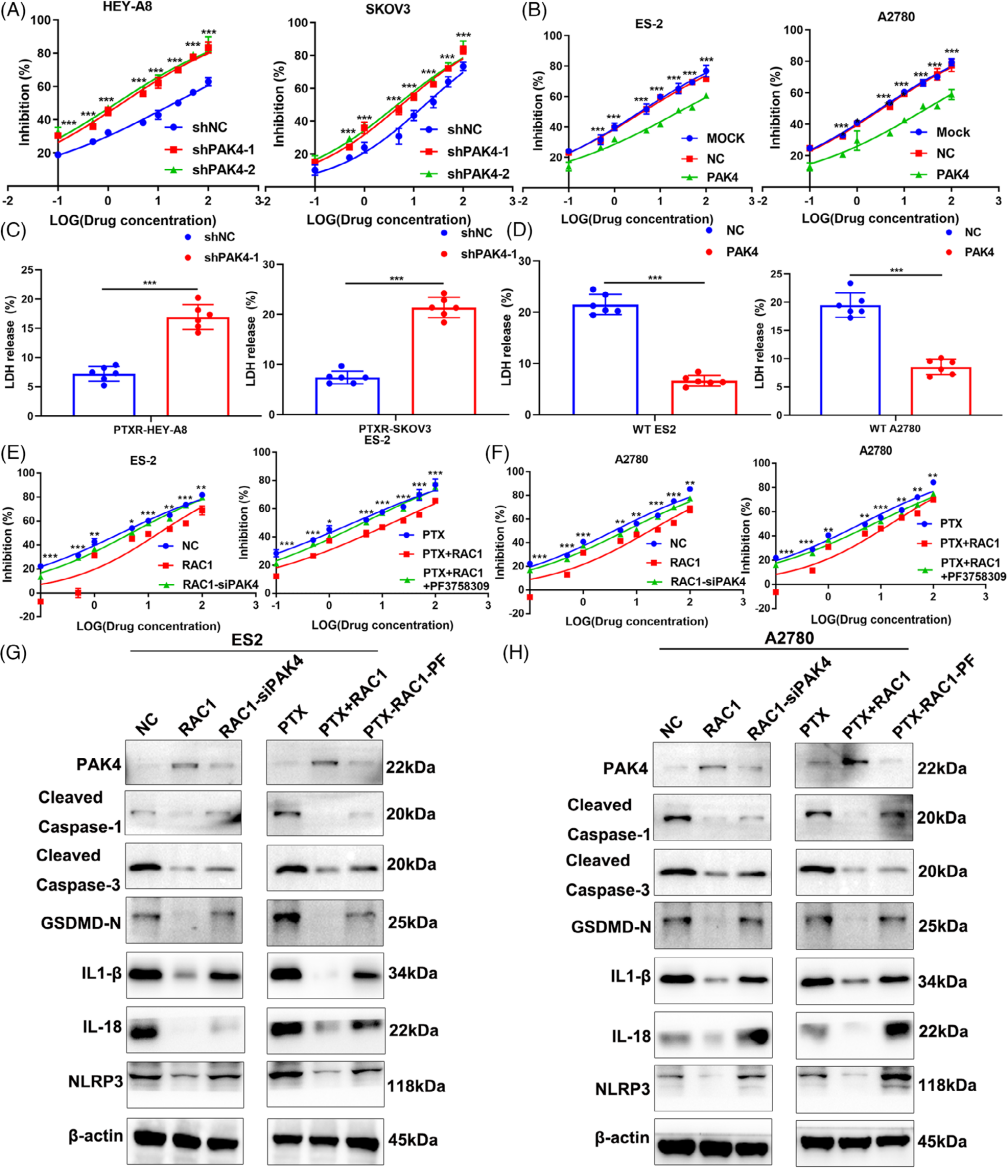
RAS‐associated C3 botulinum toxin substrate 1 (RAC1) regulates ovarian cancer (OC) progression by regulating the mitogen‐activated protein kinase (MAPK) signaling pathway through the targeting of P21‐activated kinase 4 (PAK4). (A) We showed that knocking down PAK4 in the OC cell lines HEY‐A8 and SKOV3 attenuated paclitaxel (PTX) resistance. (B) Overexpression of PAK4 in the OC cell lines ES‐2 and A2780 promoted PTX resistance. (C) In the PTXR HEY‐A8 and SKOV3 cell lines, PAK4 knockdown promoted lactate dehydrogenase (LDH) release (***p‐PTXR‐HEY‐A8 < 0.001, ***p‐PTXR‐SKOV3 < 0.001). (D) Overexpression of PAK4 significantly inhibited LDH release from WT ES‐2 cells and WT A2780 cells (***p‐WT‐ES2 < 0.001, ***p‐WT‐A2780 < 0.001). (E, F) Knocking down PAK4 with the PAK4 inhibitor PF reversed the tumor‐promoting effects of RAC1 overexpression. (G, H) PAK4 knockdown or the administration of PAK4 inhibitors reversed the downregulation of pyroptosis‐related molecular markers, the release of the inflammatory factors IL‐1β/IL‐18, and the expression of the nucleotide oligomerization domain‐like receptor family pyrin domain‐containing 3 (NLRP3) inflammasome induced by the overexpression of RAC1 (“ns” indicates no statistically significant difference, *p < 0.05, **p < 0.01, and ***p < 0.001).

Many studies indicate that PAK4 can promote resistance to chemotherapy drugs such as platinum‐based chemotherapies, but its effect on sensitivity to PTX has not been reported.^26^ Therefore, we knocked down PAK4 expression in the OC cell lines HEY‐A8 and SKOV3 and found that PAK4 knockdown attenuated PTX resistance (Figure 7A). Similarly, the overexpression of PAK4 in the OC cell lines ES‐2 and A2780 promoted PTX resistance (Figure 7B). These results indicate that PAK4 can promote PTX resistance in OC cell lines. In the PTXR HEY‐A8 and SKOV3 cell lines, PAK4 knockdown promoted LDH release (***p‐PTXR‐HEY‐A8 < 0.001, ***p‐PTXR‐SKOV3 < 0.001; Figure 7C). The overexpression of PAK4 significantly inhibited LDH release from the WT ES‐2 cell line and WT A2780 cell line (***p‐WT‐ES2 < 0.001, ***p‐WT‐A2780 < 0.001; Figure 7D). These results indicate that PAK4 can inhibit LDH release. Collectively, these results suggest that PAK4 may promote PTX resistance in OC cell lines by inhibiting pyroptosis.

Next, we further verified the roles of RAC1 and PAK4 in pyroptosis in OC cell lines through a rescue experiment. We knocked down PAK4 in the RAC1‐overexpressing OC cell lines ES‐2 and A2780 and found that PAK4 knockdown reversed the tumor‐promoting effects of RAC1 overexpression. The effects were similar when the RAC1‐overexpressing ES‐2 and A2780 cell lines were treated with PAK4 inhibitors (Figure 7E,F).

We further validated the protein expression of classical caspase‐1‐dependent pyroptosis markers.^27^ Moreover, knocking down PAK4 or administering PAK4 inhibitors reversed the downregulation of pyroptosis‐related molecular markers induced by RAC1 overexpression (Figure 7G,H). Therefore, these results suggest that RAC1 can mediate PTX resistance by targeting PAK4, thereby inhibiting the classical caspase‐1‐dependent pyroptosis pathway.

### 2.9 In animal models and clinical specimens, RAC1 can promote PTX resistance in OC cells by targeting the PAK4/MAPK signaling pathway and mediating GSDMD‐mediated pyroptosis

The above studies demonstrated that RAC1 can target PAK4 and mediate PTX resistance through the MAPK pathway, ultimately leading to caspase‐1/GSDMD‐mediated pyroptosis. Next, we further verified this mechanism at the animal level and in clinical specimens.

**FIGURE 8:**
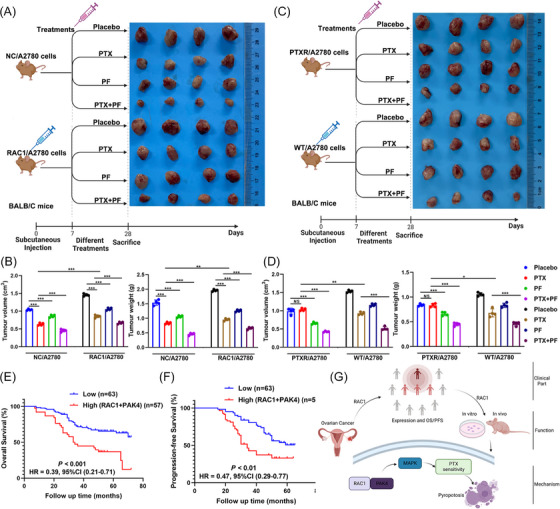
In animal models and clinical specimens, RAS‐associated C3 botulinum toxin substrate 1 (RAC1) was shown to promote paclitaxel (PTX) resistance in ovarian cancer (OC) cells by targeting the P21‐activated kinase 4 (PAK4)/mitogen‐activated protein kinase (MAPK) signaling pathway and mediating gasdermin D (GSDMD)‐related pyroptosis. (A–D) Using animal experiments, we found that PF can significantly inhibit the formation of tumors by OC cells, reverse the stimulatory effect of RAC1 overexpression, and promote the resensitization of PTXR cells to PTX. The combination of PF with PTX can enhance the inhibitory effect. (E, F) Patients with high expression of both the RAC1 and PAK4 proteins in OC tissue samples in the TMA were included in the high‐expression group, while other patients were included in the low‐expression group; the relationships of these expression subgroups with the clinical prognosis were analyzed. The results revealed that patients with high expression of both RAC1 and PAK4 had a poorer prognosis than those in the other groups did (***p‐overall survival [OS] < 0.001, hazard ratio [HR] = 0.39, 95% confidence interval [CI] = 0.21–0.71; **p‐progression‐free survival [PFS] < 0.01, HR = 0.47, 95% CI = 0.29–0.77). (G) Flow diagram (“ns” indicates no statistically significant difference, *p < 0.05, ** *p* < 0.01, and *** *p* < 0.001).

We performed an axillary injection of RAC1‐overexpressing A2780 cell lines or empty vector‐containing cell lines on day 0 and administered a placebo, PTX alone, a PAK4 inhibitor (PF3758309, PF) alone, or PF combined with PTX beginning on day 7 until week 4 to investigate the role of PAK4 in RAC‐1‐mediated PTX resistance in vivo.^28^ At the beginning of the fourth week, the mice were sacrificed, and the results revealed that PF inhibited the growth of subcutaneous OC tumors. Compared with PF alone or PTX alone, PF combined with PTX had a greater inhibitory effect on the growth of subcutaneous OC tumors. PF reversed the RAC1 overexpression‐mediated increase in tumor growth; moreover, PTX combined with PF increased the effects of PF alone on the RAC1 overexpression group (Figure 8A,B). In addition, compared with PF alone or PTX alone, PF combined with PTX significantly inhibited tumor growth in the PTXR model group (Figure 8C,D). IHC staining of tumors from the animals showed that PF promoted the expression of pyroptosis‐related proteins and that PF combined with PTX increased the expression of pyroptosis‐related proteins (Figure S5).

We further verified the relationship between RAC1/PAK4 expression and the patient prognosis in clinical samples. The group with high expression of these two genes at the same time had a worse prognosis than the other groups did, which was consistent with the results of the cell and animal experiments described above.

## 3 DISCUSSION

Our data suggest that RAC1 expression is upregulated in PTX‐resistant cancer cells, thereby promoting signaling via the PAK4/MAPK pathway and inhibiting chemotherapy‐induced pyroptosis. These findings are unexpected and require a reconsideration of the role of pyroptosis in PTX resistance and progression of OC.

Our previous studies revealed that RAC1 can increase the sensitivity of gastric cancer to chemotherapy, but the specific mechanism is not yet understood. With increasing research in this area, RAC1 has been found to promote OC proliferation, migration, invasion, angiogenesis, the EMT, and chemotherapy resistance.^22^, ^23^ In recent years, studies have shown that chemotherapy can induce pyroptosis, a new type of cell death that is different from traditional apoptotic methods, but its specific role and mechanism are unknown.^19^, ^29^

By analyzing GEPIA data, we found that RAC1 was significantly overexpressed in OC samples compared with normal samples. IHC was used to detect RAC1 expression in a TMA of samples from our center, and quantitative reverse transcription‐polymerase chain reaction (qRT‒PCR) was used to detect RAC1 mRNA levels in clinical tissues. The results also revealed that RAC1 was significantly highly expressed in OC. Therefore, RAC1 may promote cancer in OC patients. The K–M survival analysis of the database revealed that patients with high RAC1 expression had a poorer prognosis than those with low RAC1 expression. Patients with intermediate TMA staining were divided into high and low‐expression groups according to the IRS, and the results revealed that patients with high expression had poorer OS and PFS than those with low expression. Further univariate and multivariate analyses of the clinicopathological characteristics of patients revealed that RAC1 could be an independent prognostic risk factor. Taken together, these results suggest that RAC1 expression is elevated in OC and is negatively correlated with the patient prognosis.

We then conducted in vitro and in vivo studies to evaluate the relationship between RAC1 and OC. We demonstrated that knockdown or overexpression of RAC1 can inhibit or promote OC cell proliferation, migration, and invasion in vitro. RAC1 knockdown or treatment with RAC1 inhibitors can sensitize OC cells to the chemotherapeutic effect of PTX. In this study, we also showed that RAC1 can promote cell migration, invasion, and PTX resistance in OC. Moreover, our in vivo experiments proved that RAC1 inhibitors inhibited the proliferation of OC cells and that combination with PTX increased the inhibitory effect of PTX on the proliferation of OC cells. We concluded that inhibiting RAC1 in vivo can increase the effect of PTX chemotherapy on OC.

In the present study, we documented that inhibiting RAC1 can increase the effect of PTX chemotherapy on OC in vitro and in vivo. We further showed that PTX can increase LDH release in WT OC cells and induce caspase‐1 inflammasome activation, which drives GSDMD‐mediated pyroptosis. Therefore, PTX can induce pyroptosis in OC cells through caspase‐1/GSDMD signaling. In addition, overexpression of RAC1 has been shown to inhibit PTX‐induced LDH release and caspase‐1/GSDMD‐mediated pyroptosis. Inhibition of RAC1 increases PTX‐induced LDH release and caspase‐1/GSDMD‐mediated pyroptosis in PTXR OC cells. Therefore, we concluded that RAC1 can promote OC resistance by inhibiting PTX‐induced pyroptosis mediated by the caspase‐1/GSDMD pathway.

Studies have reported that RAC1 can promote tumor progression through the P38/MAPK, WNT, ERK, and other pathways.^22^, ^30^, ^31^ By performing RNA‐seq and GSEA, we found that the enrichment of the MAPK pathway was significantly reduced after RAC1 knockdown. Furthermore, knockdown or overexpression of RAC1 significantly decreased or increased the expression of A‐Raf, RAS, MAPK, and c‐Jun, respectively.^32^, ^33^, ^34^ The above results revealed that RAC1‐related genes are significantly enriched in the MAPK pathway. By reviewing the literature and performing Co‐IP–MS, we found that RAC1 can play a procancer role by targeting PAK4.^35^, ^36^, ^37^ We also proved that RAC1 can bind to PAK4 through Co‐IP. An analysis of the RNA‐seq data revealed that knocking down RAC1 led to the downregulation of PAK4 expression. Therefore, we subsequently knocked down or overexpressed RAC1 and found that PAK4 expression was reduced or increased, respectively. We verified whether RAC1 exerts its effects through the downstream factor PAK4 by conducting a follow‐up rescue experiment. At present, only one report has shown that inhibiting PAK4 can reverse the effects on 3D spheroids in a model of platinum‐resistant OC.^38^ Our results showed that knocking down PAK4 or administering the PAK4 inhibitor PF attenuated the PTX resistance and pyroptosis inhibition mediated by RAC1 overexpression‐induced activation of the caspase‐1/GSDMD pathway.

Finally, through animal experiments, we found that PF could significantly inhibit tumor formation by OC cells and reverse the tumor‐promoting effect of RAC1 overexpression. The combination of PF with PTX increased the inhibitory effect, and PF promoted the resensitization of PTXR cells to PTX. Patients with high expression of both RAC1 and PAK4 had a poor prognosis. Therefore, we showed that RAC1 can promote PTX resistance in OC cells in vivo by targeting PAK4 and inhibiting pyroptosis.

### 3.1 Limitations

Our study has several limitations. Although we demonstrated that pyroptosis in OC cells plays a crucial role in PTX resistance in vitro, the role of PTX‐induced pyroptosis in OC patient samples remains to be further explored. In addition, this study did not assess any models of resistance to multiple chemotherapeutic drugs.

## 4 CONCLUSIONS

Overall, we found that the RAC1/PAK4/MAPK pathway inhibits caspase‐1/GSDMD‐mediated canonical pyroptosis, thereby promoting PTX resistance in OC cells (Figure 8G). Therefore, our data provide proof of principle for targeting pyroptosis to overcome PTX resistance in OC and provide a basis for the subsequent clinical translation of RAC1 inhibitors combined with PTX in PTX‐resistant patients.

## 5 MATERIALS AND METHODS

### 5.1 Patient dataset

All cell and animal experiments and clinical samples used in this study were authorized by the Ethics Committee of the Shanghai Cancer Center of Fudan University (FUSCC) and approved by the Ethics Committee of the World Health Organization. All patient samples, including samples from 120 patients with a normal ovarian epithelium and 120 OC patients, were obtained from our center and prepared into tissue chips. Moreover, the clinical data of the patients were collected. Seventy‐five patients with OC and 62 patients with a normal ovarian epithelium were randomly selected for the mRNA analysis.

### 5.2 Statistical analysis

In this study, the K–M method was used for the survival analysis. The Cox risk model was used for univariate and multivariate analyses. Photoshop, GraphPad Prism 8, and Biorender were used for image generation and synthesis. IBM SPSS 23.0 and GraphPad Prism 8 were used for statistical analyses. The results from all statistical analyses are presented as two‐sided *p* values, which are shown as the means ± SDs (* *p* < 0.05, ** *p* < 0.01, and *** *p* < 0.001).

## AUTHOR CONTRIBUTIONS

Jiangchun Wu: conceptualization, investigation, data curation, writing of the original draft. Yong Wu, Tianyi Zhao, and Xiangwei Wang: formal analysis, resources, methodology, validation, software, visualization. Qinhao Guo, Siyu Chen, Simin Wang, and Xingzhu Ju: project administration, supervision, writing – review and editing. Jin Li, Xiaohua Wu, and Zhong Zheng: funding acquisition, project administration, supervision, writing – review and editing. All the authors have read and approved the final manuscript.

## CONFLICT OF INTEREST STATEMENT

The authors declare no conflicts of interest.

## ETHICS STATEMENT

All experimental protocols in this study were approved by the Ethics Committee of Experimental Research, Shanghai Medical College, Fudan University (No. 2023‐0009), and all patients provided informed consent and signed consent forms.

## Supporting information

Supporting Information

## Data Availability

The data that support the results of this study are available from the corresponding author upon reasonable request.
