# Supplementary material for: Targeting RAC1 reactivates pyroptosis to reverse paclitaxel resistance in ovarian cancer by suppressing P21‐activated kinase 4
Source: MedComm (2020). 2024 Sep 2;5(9):e719. doi: 10.1002/mco2.719 (PMC11366825; doi:10.1002/mco2.719)
Supplement: Supplementary file 1 — Supporting Information [file MCO2-5-e719-s001.docx]

**Supplementary Information for**

**Targeting RAC1 reactivates pyroptosis to reverse paclitaxel resistance in ovarian cancer by suppressing P21-activated kinase 4**

Jiangchun Wu^1,2*^, Yong Wu^1,2*^, Tianyi Zhao^1,2*^, Xiangwei Wang^1,3*^, Qinhao Guo^1,2^, Simin Wang^1,2^, Siyu Chen^1,2^, Xingzhu Ju^1,2^, Jin Li^1,2#^, Xiaohua Wu^1,2#^, and Zhong Zheng^1,2#^

^1^Department of Gynecologic Oncology, Fudan University Shanghai Cancer Center, Fudan University, Shanghai, China.

^2^Department of Oncology, Shanghai Medical College, Fudan University, Shanghai, China.

^3^Department of Nuclear Medicine, Fudan University Shanghai Cancer Center, Shanghai, China.

*These authors contributed equally to this work.

^#^ These authors contributed equally to this work.

**Correspondence**

Zhong Zheng, Department of Gynecologic Oncology, Fudan University Shanghai Cancer Center; Department of Oncology, Shanghai Medical College, Fudan University, Shanghai 200032, Shanghai, China.

E-mail: alizheng@126.com

Xiaohua Wu, Department of Gynecologic Oncology, Fudan University Shanghai Cancer Center; Department of Oncology, Shanghai Medical College, Fudan University, Shanghai 200032, Shanghai, China.

E-mail: [docwxh1963@163.com](mailto:docwxh1963@163.com)

Jin Li, Department of Gynecologic Oncology, Fudan University Shanghai Cancer Center; Department of Oncology, Shanghai Medical College, Fudan University, Shanghai 200032, Shanghai, China.

E-mail: dr.jinli@yahoo.com

**This PDF file includes:**

Materials and Methods

Figures and Figure Legends S1 to S5

Table S1 to S6

**MATERIALS AND METHODS**

**TMA**

In this study, the tissue chip contained 120 normal ovarian epithelium and 120 ovarian cancer tissues. As described in our previous article, TMA was prepared from ovarian tumor tissue in ovarian cancer patients and normal ovarian epithelial tissue in normal patients. In this paper, the tissue was embedded by using paraffin wax. Through the method of fine needle drilling, tens to hundreds of small cylindrical tissues were collected from many different patients' tumor tissues or normal patients' epithelial tissue wax blocks (donor wax blocks), and neatly arranged into another empty blank wax block (recipient wax blocks) to make tissue chip wax blocks. Finally, the tissue wax blocks were sliced and the sections were transferred to a slide to make TMA.

The detailed steps are as follows: First, make the recipient wax block, the size of 20mm width x 45mm length. Then, holes were drilled on the wax block with a spacing of 0.1mm and a hole diameter of 0.6mm, and the coordinates of each hole were precisely positioned. Furthermore, the coordinates of each representative point on the wax block were labeled according to the HE staining results, including ovarian tissue from ovarian cancer patients or normal ovarian epithelial tissue. By using stainless steel needle (depth :2-3 mm; Inside diameter 0.6 mm) to fix the tissue in the pore of the recipient wax block. Finally, the prepared acceptor wax blocks were pre-cooled at 4℃ for about 4 hours and sliced with a microtome. A total of about 30 slices (thickness: 5µm) were quickly generated and transferred to an empty slide^1^.

**qRT‒PCR**

First, RNA was extracted, the supernatant of cells or tissues was discarded, and the supernatant was washed with PBS buffer for 3 times. Add 500ul TRIZOL to each hole, crack on ice for 2 minutes, blow with the gun head until there is no slag like material, suction and put into a new 1.5ml EP tube. Add 100ul chloroform, 4℃, 12000g, 10min centrifuge; Absorb 200ul of supernatant into a new 1.5ml EP tube; Add isopropyl alcohol, mix well, leave at room temperature for 15-30 min, 4℃, 12000g, 10min centrifuge; Pour away the liquid, add 1 ml of pre-cooled 75% ethanol, mix well, 4℃, 7500g, centrifuge for 5min; Pour off the supernatant, put it upside down on a napkin, 5-10 minutes, add 40ul DEPC water to each tube, blow and dissolve; Measure the concentration. Then reverse transcription and PCR were performed according to standard procedures. According to the instruction manual, we use a conventional TRIZOL reagent (Invitrogen, Carlsbad, CA, USA) to extract total RNA from a cell or tissue sample, The TB Green PCR Master Mix Kit (TaKaRa, Dalian, China) was used during this period and was eventually executed in the ABI 7900HT Real-Time PCR system (Applied Biosystems, USA). All primer sequences are shown in Table S5.

**IHC**

The TMA were roasted at 60 ° C for 2 hours to prevent peeling. Dewaxing of xylene I for 2 hours, xylene II for 20 minutes, followed by 100% ethanol, 95% ethanol, 80% ethanol, 70% ethanol for 5 minutes each. Rinse with running water twice, rinse with distilled water twice, soak with PBS buffer for 5 minutes 3 times. Microwave antigen repair: The slices were placed in a container of citrate buffer, boiled in the microwave for 4 minutes, soaked in PBS buffer for 5 minutes, and repeated 3 times. Incubate at 3% H_2_O_2_ at room temperature for 10-15 minutes, soak in PBS buffer for 5 minutes, repeat 3 times. The circle was drawn with immunohistochemical pen and closed with normal sheep serum (about 100 microliters) for 10 minutes. Discard the serum and add the corresponding antibody. TMA and animal sample slides were rinsed and the primary antibodies against RAC1 (1:2000; Cell Signaling Technology; CST), Cy3-conjugated goat anti-rabbit secondary antibody (1:5000; Invitrogen; Supplementary Table S6). The expression of RAC1 was scored by IRS, with scores of 0 (no staining), 1 (light yellow), 2 (brownish-yellow), and 3 (brown). The final score is negative 0 (0-1), weak positive 1 (2-3), medium positive 2 (4-7), and strong positive 3 (8-12) ^2-4^.

**Cell culture and treatments**

The human OC cell lines SKOV3, HEY-A8, A2780, ES-2, OVCA429, HO8910, TOV-21G, and OVCA433 were obtained from the American Type Culture Collection (ATCC) and authenticated by short tandem repeat profiling. The above OC cell lines were cultured in high-sugar Dulbecco's modified Eagle's medium (DMEM; Gibco, USA), in which 10% fetal bovine serum (FBS; Gibco, USA) and 1% penicillin-streptomycin (Gibco, USA). In addition, in the cell half-life experiment, 50 μg/ml cycloheximide (CHX, Sigma-Aldrich) was applied to the corresponding cells for 0h, 4h, 8h, 12h. The cells were then treated with protease inhibitor MG132 (20 μM, Selleckchem) for 6 hours, and cells were collected for WB experiments.

**Plasmids**

RAC1 overexpression plasmid was successfully established by cloning the cDNA of target gene RAC1 into PGMLV-CMV-EF1-ZsGreen1-T2A-Puro vector (System Biosciences, CA, USA). Plasmid targeting RAC1 shRNA was constructed using U6-MCS-CMV-ZsGreen1-PGK-Puro vector (System Biosciences, CA, USA). All the SiRNA and corresponding control empty vectors in the study were from Lncbio (Shanghai, China). The siRNA sequences used in this research are shown in Table S5. Lipofectamine RNAi MAX (Invitrogen, CA, USA) was used for siRNA transfection in all experiments. According to the manufacturer's instructions, Lipofectamine 3000 (Invitrogen, CA, USA) was used for transient transfection. After successful transfection, puro was used for resistance screening. Cell lines were expanded after screening. The amplified cell lines were verified by Western blotting (WB) and qRT-PCR. Lipofectamine RNAi MAX (Invitrogen, CA, USA) is mainly used for siRNA transfection.

**Western blotting**

WB is executed as described above ^5^. In this study, all cells applied to WB were lysed at 10% NP40(ratio :890µl NP40 buffer + 100µl NP40 + 10µl protease inhibitor cocktail) for 15-30 min and centrifuged at 4℃ for 30 min after lysation. The supernatant was collected and the protein concentration was quantified by Bio-Rad protein detection kit (Hercules, CA, USA). Each sample was prepared into an equal concentration solution, about 15-20µg of protein in each sample was separated by 10% SDS-PAGE electrophoresis, and then transferred to a polyvinylidene fluoride (PVDF) membrane. Seal with 5% milk for 15 min, and wash with Tris Buffered Saline Tween (TBST) after sealing, three times for 10 min each time. The corresponding primary antibody was proportioned with the diluent of the primary antibody according to the instructions. The film cut according to the molecular weight of marker was completely immersed in the prepared primary antibody and incubated at 4℃ overnight. On the second day, wash with TBST and repeat three times for 10 min each time; The diluted solution of secondary antibody derived from the corresponding species was incubated for 2 hours, followed by another cleaning with TBST buffer, repeated three times for 10 min each time. Finally, ECL luminescent solution was used for exposure. All antibody related information in this study is shown in Table S6.

**CCK-8 assay**

OC cell lines with stable knockout or stable overexpression and no-load were expanded to a logarithmic growth phase, and then counted, and 2000 cells were implanted in 96-well plates. After 1 day in a warm box, the cells were attached to the wall, the medium was discarded, and the Counting Kit 8 solution (CCK8, MedChem Express, USA) reagent was diluted 10-fold with the culture medium. After 1 hour, the absorbance value was detected at 450nm wavelength. Absorbance values were measured on the second, third, fourth and fifth days of the same time period every day to assess cell proliferation capacity ^6^.

**Cell cycle assay**

OC cells of logarithmic growth stage were inoculated into 6-well plates (2*10^5^ cells per well, appropriately adjusted according to cell size and proliferation rate), and 2ml culture medium was added to each well. 24 hours later, after the cells adhere to the wall, digest and collect the adherent cells. The blowing process must be gentle and full to avoid cell damage and adhesion. Centrifuge at 2000rpm for 4 min, discard the supernatant, and wash with PBS buffer for 3 times. A small amount of PBS buffer was added to the cell precipitation, the cells were gently and fully resuspended, and the resuspended cells were added to 70% ice ethanol pre-cooled at 4℃ for fixation, gently blown evenly, sealed with a sealing film, and kept overnight at 4℃. The next day, centrifuge at 2000rpm for 4 min, suck the fixative, and wash it twice with PBS to remove the fixative. The cells were added with 200ul working solution containing propyl iodide (PI, 0.05mg /ml) and RnaseA (0.1mg /ml, RNA removal), and incubated at room temperature for 30 minutes away from light. The cell cycle was measured by flow cytometry ^7^.

**Cell migration and invasion assays**

In wound healing experiment, OC cells were counted and evenly spread in 6-well plates for culture, with equal number of each well. 24 hours later, after the cells were attached to the wall, a mark was made along the center line with the tip of a 1ml pipette. The mark was photographed at 0 h or 36 h, respectively, and the relative mobility of OC cells treated in different groups was calculated ^8, 9^. For migration analysis, a 24-well plate with a Transwell chamber (8µm aperture, Corning) was used in this study. The concentration of fetal bovine serum (FBS) in upper and lower cavities was different (upper cavity :10% FBS; Lower cavity: without FBS). Dissolved extracellular matrix Matrigel (Corning) glue was added to the surface of the chamber and pre-cooled at 4 ° C for 12 hours. 4×104 cells were added to the medium in the upper cavity. Culture for 12 hours, absorb the medium in the upper and lower cavities, wash with running water, add 4% paraformaldehyde (PFA) and fix for 15 minutes. Absorb PFA, wash with PBS three times, add 0.1% crystal violet and dye for 15 minutes. Allow to dry and take pictures under the microscope ^10, 11^.

**Drug treatment and half-maximal inhibitory concentration (IC50)**

OC Cells of different groups (4×10^3^ Cells /well) were counted, evenly spread in 96-well plates, shaken well, and cultured overnight in incubators. After 1day, different groups were given PTX with corresponding different concentration gradients (0.08, 0.4, 2, 10, 50 nM). 2 days later, each well of the drug and the medium were removed, washed with PBS buffer, and repeated 3 times. 90µl medium was added and 10µl CCK8 solution was added to each well and further incubated in an incubator at 37°C. After incubation for 1 h, the absorbance of each group was measured at 450nm wavelength, and the proliferation rate of ovarian cancer cells in each group was calculated. PTX is purchased from FUSCC Pharmacy and used within one month of purchase.

**RNA sequencing (RNA-seq)**

Firstly, the corresponding groups of cells were re-suspended with PBS and transferred into 1.5ml EP tubes. The samples were then sequenced by OEbiotech (P.R. China). HiSeq3000 system (Illumina, USA) was used to determine differential gene expression. The following truncation values were used to determine differential gene expression: break change (FC)| > 2 (P<0.05). The mean location reading per million (FPKM) values of overlapping genes in cell subpopulations transfected with these two independent Sinas were further analyzed. Finally, we performed GSEA pathway and KEGG analysis respectively.

**Co-IP and MS analysis**

The cells were washed with PBS buffer, repeated three times, and discarded. Then IP product (IP product ratio: NP40 buffer 890 µl, 10% NP40 buffer 100 µl, protease inhibitor 10 µl) was added, digested on ice for 30 min, mixed with a shaker after cracking, centrifuged with 12000g at 4℃ for 15 min, and the supernatant was transferred to a new EP tube. The protein concentration was determined by BCA method, and the solution with the same concentration was prepared by 5x loading buffer and boiled in a metal bath at 100℃ for 30 min. Then, magnetic beads with corresponding antibodies were added and mixed overnight at a uniform speed on a Ferris wheel at 4℃. The eluted products were separated by gel electrophoresis and transferred to an inhibited PVDF membrane (Millipore). Finally, the corresponding primary and secondary antibodies were used for detection. At the same time, mass spectrum analysis of the products after the end of the Ferris wheel was carried out to obtain the corresponding data.

**Ubiquitination assay**

HEK293T cells were transfected with His-Ub, Myc-PAK4 or Flag-RAC1 overexpression plasmid. 36 h after transfection, MG132 protease inhibitor (20µM, Selleckchem) was added and treated for 6 h. The cells were subjected to digestion and WB tests, and ubiquitin antibodies, PAK4 and RAC1 antibodies were added for protein ubiquitin experiments. The WB test is the same as above ^12^.

**In vivo assays**

BALB/c female nude mice (4-6 weeks old, 18-20 g) were purchased from the Shanghai Institute of Family Planning and raised under SPF standard conditions in the animal Laboratory of Shanghai Cancer Center, Fudan University. To establish a subcutaneous tumor model, 5×106 HEYA8, or A2780/NC, or A2780/RAC1, or WT A2780, or PTXR A2780 cells were resuspended in 0.1 ml sterile PBS and randomly injected into the side of mice (6 cells per group). One week after the cells were injected, each group was treated with placebo, PTX (20 mg/kg, qw, ip), NSC (NSC23766, 2.5 mg/kg, qd, ip), PTX+NSC, PF (PF3758309, 2mg /kg, qd, ip) and PF+PTX, respectively. The details are shown in the figure. The tumor volume was measured weekly during growth, and the tumor volume was calculated as (length*width^2^)*0.5. After 4 weeks of cell injection, the mice were killed and the tumor mass and volume of the mice were measured.

**High throughput automated confocal and LDH release assays**

High throughput automated confocal images and LDH release experiments were carried out on cells with different concentrations of PTX, different treatment times and different drug resistance, among which the LDH experiment was shown as follows ^13^. One percent NP40 or 2.5% Triton 100 μL was added to the cells. Three replicate wells were used for each of the above conditions, and the cells were cultured in a 5% CO2 incubator at 37°C for 4 h. The 96-well culture plate was subsequently centrifuged at 1500 r/min for 5 minutes. One hundred microlitres of the supernatant was added to each well, the samples were placed in a 96-well culture plate on the flat bottom, and 100 μL of LDH matrix solution was added for 3 minutes. The optical density (OD) was measured at 490 nM by adding 30 μL of 1 mol/L HCl to each well ^14^.

**Online tools**

The prognostic values of RAC1 and PAK4 were screened in Kaplan-Meier Plotter (http://kmplot.com/analysis/index.php?p=service).

**References**

**1.** Wu J, Wu Y, Chen S, et al. PARP1-stabilised FOXQ1 promotes ovarian cancer progression by activating the LAMB3/WNT/β-catenin signalling pathway. *Oncogene.* Mar 2024;43(12):866-883.

**2.** Specht E, Kaemmerer D, Sanger J, Wirtz RM, Schulz S, Lupp A. Comparison of immunoreactive score, HER2/neu score and H score for the immunohistochemical evaluation of somatostatin receptors in bronchopulmonary neuroendocrine neoplasms. *Histopathology.* Sep 2015;67(3):368-377.

**3.** Hofmann M, Stoss O, Shi D, et al. Assessment of a HER2 scoring system for gastric cancer: results from a validation study. *Histopathology.* Jun 2008;52(7):797-805.

**4.** Beilner D, Kuhn C, Kost BP, et al. Nuclear receptor corepressor (NCoR) is a positive prognosticator for cervical cancer. *Arch Gynecol Obstet.* Nov 2021;304(5):1307-1314.

**5.** Wang S, Li J, Xie J, et al. Programmed death ligand 1 promotes lymph node metastasis and glucose metabolism in cervical cancer by activating integrin beta4/SNAI1/SIRT3 signaling pathway. *Oncogene.* Jul 2018;37(30):4164-4180.

**6.** Zhang L, Cheng H, Yue Y, Li S, Zhang D, He R. H19 knockdown suppresses proliferation and induces apoptosis by regulating miR-148b/WNT/beta-catenin in ox-LDL -stimulated vascular smooth muscle cells. *J Biomed Sci.* Feb 7 2018;25(1):11.

**7.** Zhang S, Cheng J, Quan C, et al. circCELSR1 (hsa_circ_0063809) Contributes to Paclitaxel Resistance of Ovarian Cancer Cells by Regulating FOXR2 Expression via miR-1252. *Mol Ther Nucleic Acids.* Mar 6 2020;19:718-730.

**8.** Mascharak S, Talbott HE, Januszyk M, et al. Multi-omic analysis reveals divergent molecular events in scarring and regenerative wound healing. *Cell Stem Cell.* Feb 3 2022;29(2):315-327 e316.

**9.** Monika P, Chandraprabha MN, Rangarajan A, Waiker PV, Chidambara Murthy KN. Challenges in Healing Wound: Role of Complementary and Alternative Medicine. *Front Nutr.* 2021;8:791899.

**10.** Liu Z, Wang Y, Dou C, et al. Hypoxia-induced up-regulation of VASP promotes invasiveness and metastasis of hepatocellular carcinoma. *Theranostics.* 2018;8(17):4649-4663.

**11.** Yang P, Li J, Peng C, et al. TCONS_00012883 promotes proliferation and metastasis via DDX3/YY1/MMP1/PI3K-AKT axis in colorectal cancer. *Clin Transl Med.* Oct 2020;10(6):e211.

**12.** Akimov V, Barrio-Hernandez I, Hansen SVF, et al. UbiSite approach for comprehensive mapping of lysine and N-terminal ubiquitination sites. *Nat Struct Mol Biol.* Jul 2018;25(7):631-640.

**13.** Su L, Chen Y, Huang C, et al. Targeting Src reactivates pyroptosis to reverse chemoresistance in lung and pancreatic cancer models. *Sci Transl Med.* Jan 11 2023;15(678):eabl7895.

**14.** Laguna-Goya R, Utrero-Rico A, Talayero P, et al. IL-6-based mortality risk model for hospitalized patients with COVID-19. *J Allergy Clin Immunol.* Oct 2020;146(4):799-807 e799.

**Supplementary Figures and Legends**

**Supplementary Fig. S1 RAC1 is overexpressed in OC and clinically associated with patient prognosis, related to Figure 1. A** By querying the GEPIA database, it was found that RAC1 expression was significantly elevated in OC (**P* < 0.05). **B** High RAC1 expression in the GEPIA database was associated with poor PFS (**P-PFS* < 0.05).


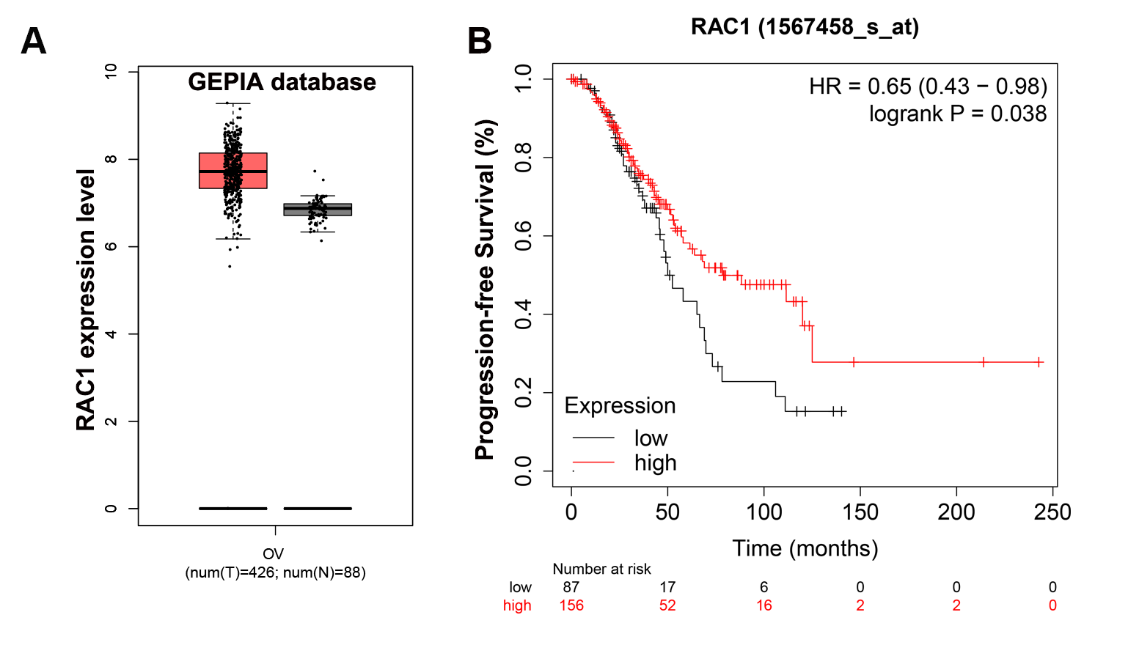


**Supplementary Fig. S2 Basic expression of RAC1 in OC cell lines and validation of stable cell line construction.** **A, B** WB and qRT‒PCR analyses of RAC1 expression in 8 OC cell lines. **C, D** qRT‒PCR and WB analyses of RAC1 levels following RAC1 overexpression and knockdown.


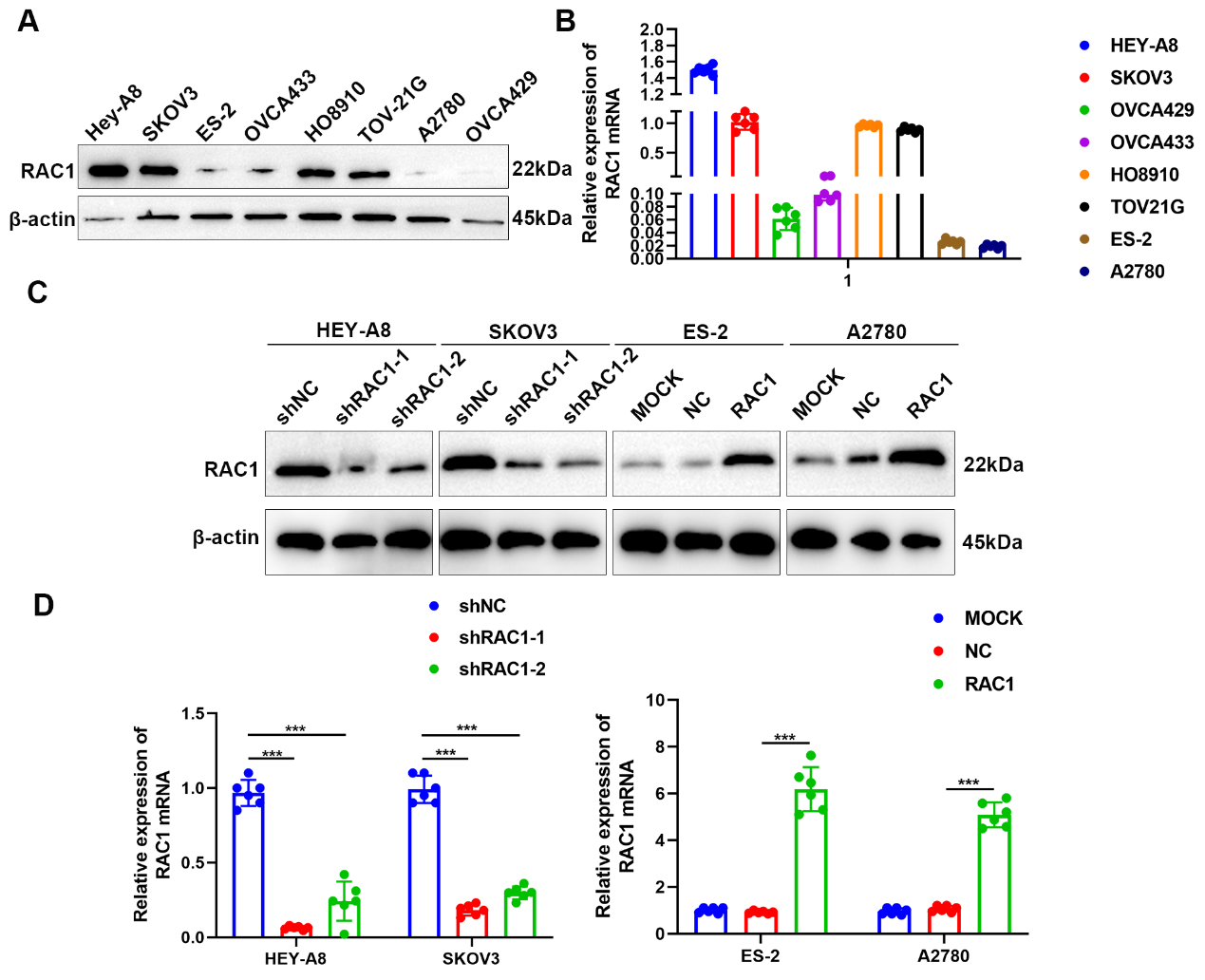


**Supplementary Fig. S3 PAK4 is clinically associated with patient prognosis. A, B** High RAC1 expression in the GEPIA database was associated with poor PFS (****P-OS* < 0.001, ****P-PFS* < 0.001).


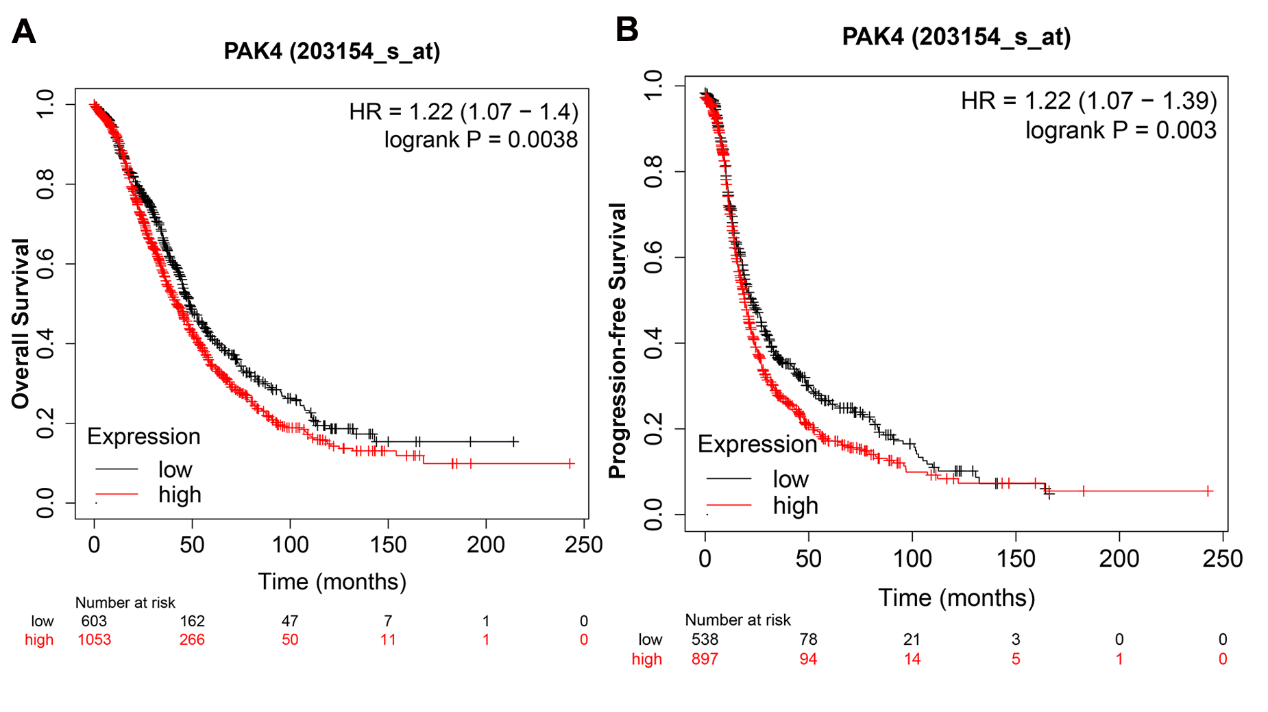


**Supplementary Fig. S4 PAK4 is clinically associated with patient prognosis in our center. AB** By IHC analysis of the correlation at the protein level, 71.43% of patients with low RAC1 expression had low PAK4 expression, and 73.08% of patients with high RAC1 expression had high PAK4 expression (The IHC figures on the right represents the partial magnification in the small box on the left (20x vs. 4x)). **CD** PAK4 protein expression levels were grouped, and patients with high PAK4 protein expression were shown to have a poorer prognosis than those with low expression (Negative: N, Weekly Positive: W, Moderately Positive: M, Strongly Positive: S; ****P-OS* < 0.01, hazard ratio (HR) = 0.48, 95% CI (0.28-0.80); **P-PFS* < 0.001, HR= 0.32, 95% CI (0.20-0.53)).


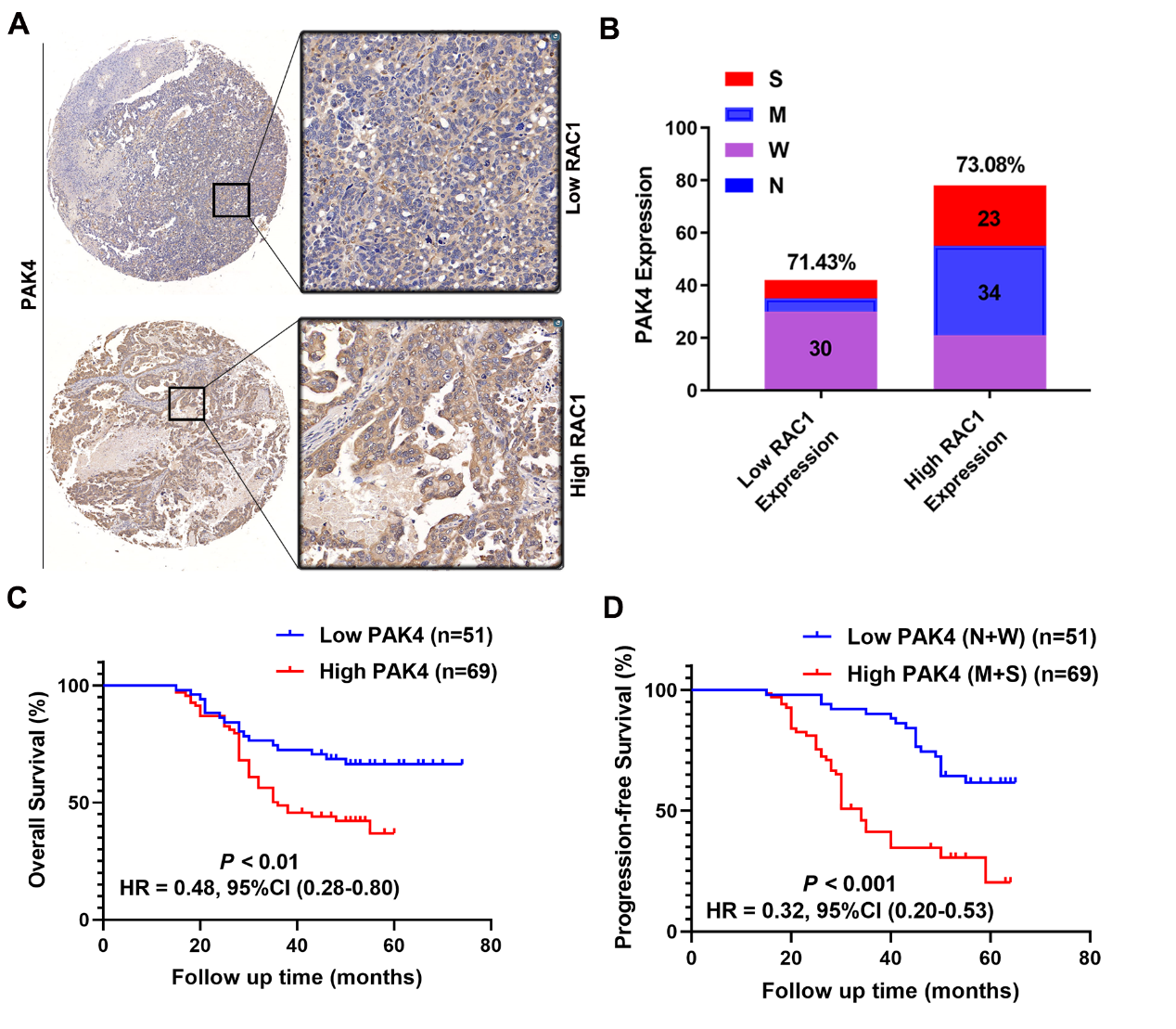


**Supplementary Fig. S5 IHC staining of animal tumors showed that PF could promote the expression of pyroptosis-related proteins, and PF combined with PTX could enhance the expression of pyroptosis-related proteins.**


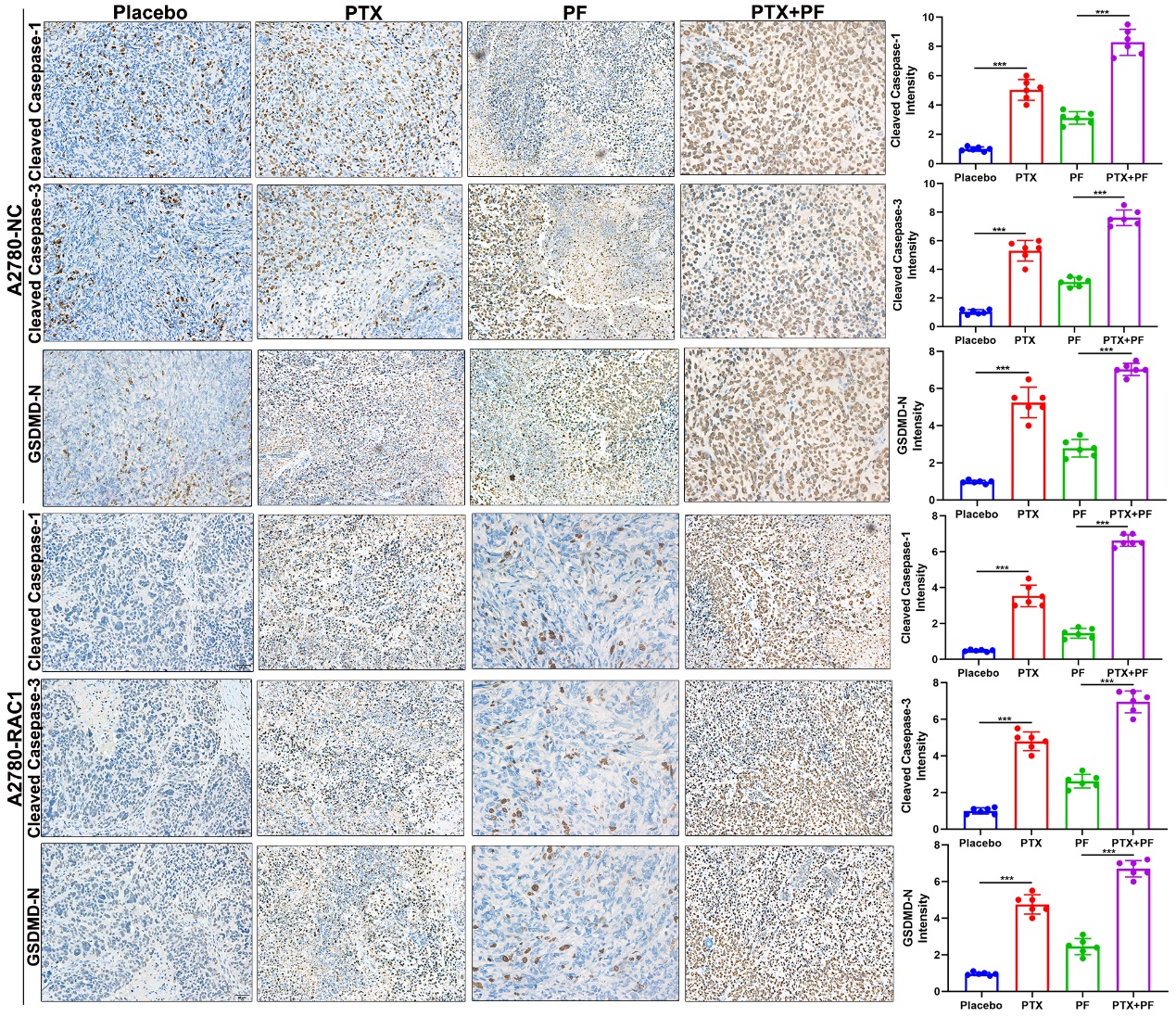


**Supplementary Table S1 Correlation between RAC1 and clinicopathological parameters in patients with OC.**

| Variables | n (%) | Expression of RAC1 | | χ^2^ | P value |
| --- | --- | --- | --- | --- | --- |
|  |  | Low | High |  |  |
| Age (years) |  |  |  | 1.597 | 0.206 |
| ≤ 55 | 62 (51.67) | 25 | 37 |  |  |
| > 55 | 58 (48.33) | 17 | 41 |  |  |
| Size (cm) |  |  |  | 1.218 | 0.270 |
| ≤ 5 | 69 (57.5) | 27 | 42 |  |  |
| > 5 | 51 (42.5) | 15 | 36 |  |  |
| FIGO stage |  |  |  | 10.966 | 0.001* |
| Ⅰ-Ⅱ | 61 (50.83) | 30 | 31 |  |  |
| Ⅲ–Ⅳ | 59 (49.17) | 12 | 47 |  |  |
| Poor histologic differentiation |  |  |  | 3.052 | 0.081 |
| No | 50 (41.67) | 13 | 37 |  |  |
| Yes | 70 (58.33) | 29 | 41 |  |  |
| Lymphatic Vascular invasion |  |  |  | 0.617 | 0.432 |
| No | 63 (52.5) | 20 | 43 |  |  |
| Yes | 57 (47.5) | 22 | 35 |  |  |
| Lymphatic metastasis |  |  |  | 0.003 | 0.953 |
| No | 71 (59.17) | 25 | 46 |  |  |
| Yes | 49 (40.83) | 17 | 32 |  |  |
| Distant metastasis |  |  |  | 5.018 | 0.025* |
| No | 68 (56.67) | 18 | 50 |  |  |
| Yes | 52 (43.33) | 24 | 28 |  |  |

**Supplementary Table S2. RNA-seq analyses of the differentially gene expression profile affected by RAC1 knockdown.**

| GeneID | | | HEY-A8-NC HEY-A8-Si1 | | |  |
| --- | --- | --- | --- | --- | --- | --- |
| TGFBI | | 0.473226784 | | 0.020264995 |  | |
| ITGAX | | 0.250884991 | | 0.012086607 |  | |
| NTN3 | | 0.519850504 | | 0.02862201 |  | |
| GMPR | | 0.537627027 | | 0.034534212 |  | |
| SIK1 | | 0.141032844 | | 0.012078902 |  | |
| TNFRSF13C | | 1.973889377 | | 0.182580232 |  | |
| KRT15 | | 0.707021713 | | 0.066058469 |  | |
| LOC728392 | | 0.790296125 | | 0.076146433 |  | |
| SBK2 | | 2.633732398 | | 0.253764787 |  | |
| OLFM2 | | 0.840081062 | | 0.084462574 |  | |
| PALM3 | | 1.108714654 | | 0.115488241 |  | |
| GS1-259H13.2 | | 1.295829998 | | 0.13620602 |  | |
| DISC1 | | 0.896683735 | | 0.095334696 |  | |
| GFAP | | 0.163720758 | | 0.018028326 |  | |
| LAPTM5 | | 0.230451585 | | 0.025376479 |  | |
| LOC390937 | | 0.620198098 | | 0.068293928 |  | |
| PADI3 | | 1.659251411 | | 0.189477708 |  | |
| OPRL1 | | 0.376824257 | | 0.043569223 |  | |
| GALNT9 | | 0.271869151 | | 0.034926766 |  | |
| SCT | | 0.765513915 | | 0.098344831 |  | |
| CYP2A6 | | 0.752494971 | | 0.0966723 |  | |
| LOC107987205 | | 0.196914572 | | 0.025297424 |  | |
| PRR29 | | 0.142363913 | | 0.018289354 |  | |
| NOTCH3 | | 6.998921134 | | 1.014178786 |  | |
| SLC18A3 | | 2.40736201 | | 0.352334581 |  | |
| LOC107987175 | | 0.696359841 | | 0.107352809 |  | |
| LSMEM2 | | 0.1871688 | | 0.028854473 |  | |
| TRIML2 | | 0.179689345 | | 0.027701419 |  | |
| S1PR5 | | 0.150070222 | | 0.023135251 |  | |
| WNT11 | | 1.804836886 | | 0.282586257 |  | |
| ITGB4 | | 0.501500858 | | 0.082835138 |  | |
| MMP9 | | 0.441961943 | | 0.073000829 |  | |
| UBA7 | | 0.31162786 | | 0.051472966 |  | |
| VPREB3 | | 1.134530879 | | 0.194336111 |  | |
| IDH2 | | 89.70687423 | | 15.44279939 |  | |
| HIST1H1E | | 1.597014804 | | 0.289647452 |  | |
| TESC | | 1.224274044 | | 0.222044189 |  | |
| ACOT11 | | 0.880946643 | | 0.160826725 |  | |
| DCST2 | | 0.742494031 | | 0.137357984 |  | |
| HIST1H4J | | 1.657179936 | | 0.319344452 |  | |
| PYCARD | | 0.381601589 | | 0.07353598 |  | |
| GYLTL1B | | 1.112704747 | | 0.214422152 |  | |
| ZNF853 | | 0.229376383 | | 0.044201643 |  | |
| PLEKHG6 | | 0.188364003 | | 0.036298411 |  | |
| PRR25 | | 0.243985135 | | 0.047016801 |  | |
| CRYM | | 8.088717589 | | 1.567839918 |  | |
| LRRC58 | | 8.235427193 | | 1.620289654 |  | |
| CYP2E1 | | 1.548324985 | | 0.306892508 |  | |
| GYG2 | | 0.400098697 | | 0.081158356 |  | |
| GLI1 | | 1.679230204 | | 0.343087208 |  | |
| RTN4RL2 | | 0.863674408 | | 0.17923567 |  | |
| CCDC188 | | 0.973038279 | | 0.204554103 |  | |
| CDHR5 | | 0.464998325 | | 0.097752902 |  | |
| TRPV4 | | 2.479663299 | | 0.523660178 |  | |
| SERF1B | | 1.124390487 | | 0.23908859 |  | |
| POP7 | | 34.26112832 | | 7.318881422 |  | |
| TRPC6 | | 2.869497088 | | 0.62850356 |  | |
| TMEM240 | | 0.462141047 | | 0.101778536 |  | |
| NTRK1 | | 0.194429962 | | 0.042819821 |  | |
| RASSF5 | | 0.283944747 | | 0.062533897 |  | |
| LOC107985555 | | 13.82709509 | | 3.075477722 |  | |
| PITPNM3 | | 0.464061365 | | 0.103337024 |  | |
| TFCP2L1 | | 8.909371919 | | 2.03208568 |  | |
| PAK3 | | 0.293834457 | | 0.06733539 |  | |
| TERT | | 0.963980485 | | 0.22291495 |  | |
| CMTM2 | | 1.387478968 | | 0.320846542 |  | |
| RAB42 | | 0.364710718 | | 0.084337259 |  | |
| BIRC7 | | 1.778924514 | | 0.415521289 |  | |
| PALM | | 1.050882072 | | 0.246532334 |  | |
| TMEM121 | | 0.797800407 | | 0.187160483 |  | |
| NAV2 | | 2.068634823 | | 0.506129879 |  | |
| NPTX1 | | 1.1257668 | | 0.282282405 |  | |
| EFHD1 | | 2.067269896 | | 0.522002385 |  | |
| SEMA3F | | 6.626903655 | | 1.681939106 |  | |
| TRIM73 | | 0.651645129 | | 0.167432437 |  | |
| HIST1H4E | | 0.586826317 | | 0.150778017 |  | |
| SULT1A2 | | 0.256502634 | | 0.06590529 |  | |
| OSBPL6 | | 0.21219987 | | 0.054522223 |  | |
| FAM132B | | 0.305044497 | | 0.078377542 |  | |
| C1QTNF9B-AS1 | | 1.650996429 | | 0.424203824 |  | |
| LBH | | 0.286293784 | | 0.07355977 |  | |
| QRFP | | 0.237375023 | | 0.060990679 |  | |
| CHST2 | | 2.228199998 | | 0.577089466 |  | |
| NUPR1 | | 9.784084829 | | 2.555455929 |  | |
| FOXO6 | | 1.16738409 | | 0.307261148 |  | |
| SEMA4A | | 7.001338624 | | 1.894777079 |  | |
| GRIN2C | | 0.484816412 | | 0.131301184 |  | |
| MYLK2 | | 0.369646652 | | 0.101760316 |  | |
| C2orf48 | | 1.628427602 | | 0.448291107 |  | |
| LTC4S | | 1.54786072 | | 0.426111787 |  | |
| DHRS3 | | 2.886137259 | | 0.798601478 |  | |
| PPM1H | | 0.419598903 | | 0.116795173 |  | |
| KCNK5 | | 0.916201913 | | 0.255441795 |  | |
| ADGRB2 | | 1.494370327 | | 0.421671034 |  | |
| HOOK1 | | 10.03489366 | | 2.843621398 |  | |
| MADCAM1 | | 0.911610693 | | 0.25888301 |  | |
| AIFM3 | | 0.485760442 | | 0.14041164 |  | |
| PTGER1 | | 0.415169639 | | 0.120006993 |  | |
| SERPINF2 | | 2.239394565 | | 0.647308914 |  | |
| SPEG | | 1.108313764 | | 0.320981262 |  | |
| SCARF2 | | 5.921398352 | | 1.715658061 |  | |
| INHBE | | 0.635467645 | | 0.186600943 |  | |
| NFATC4 | | 1.513703041 | | 0.448762992 |  | |
| EMP1 | | 1.025690363 | | 0.30408334 |  | |
| ARHGEF10L | | 3.750655561 | | 1.113907842 |  | |
| FAM69B | | 14.44325577 | | 4.296162577 |  | |
| TMEM238 | | 6.015412969 | | 1.803186646 |  | |
| SV2A | | 9.652247538 | | 2.921972806 |  | |
| REPS2 | | 0.259631108 | | 0.078621456 |  | |
| GPR161 | | 1.774645416 | | 0.538092487 |  | |
| CERS4 | | 0.857796523 | | 0.260473409 |  | |
| PPP2CA | | 66.7132488 | | 20.38874769 |  | |
| CORO1A | | 0.530281691 | | 0.16349946 |  | |
| TMEM130 | | 0.601504952 | | 0.18545942 |  | |
| DACT3 | | 0.817113653 | | 0.251937119 |  | |
| ATP2A1 | | 2.940603933 | | 0.916309051 |  | |
| NDRG4 | | 9.878160603 | | 3.080808562 |  | |
| HECW1 | | 1.107367901 | | 0.3462052 |  | |
| KIF7 | | 3.275072512 | | 1.024423566 |  | |
| XKR5 | | 1.333879974 | | 0.41804861 |  | |
| FAM71F2 | | 0.342348984 | | 0.107509704 |  | |
| GNMT | | 1.850466257 | | 0.581111929 |  | |
| ITGB7 | | 0.535262012 | | 0.168785817 |  | |
| 1-Sep | | 1.665117164 | | 0.525066519 |  | |
| ARHGAP6 | | 6.715421748 | | 2.121978856 |  | |
| ASB4 | | 1.311356299 | | 0.416216722 |  | |
| EVC | | 2.286445287 | | 0.727512425 |  | |
| ATP6V1G2 | | 1.058093365 | | 0.336875526 |  | |
| B4GALNT4 | | 7.210436493 | | 2.301879834 |  | |
| RBFOX3 | | 0.941908035 | | 0.301039349 |  | |
| FAM65C | | 0.80953995 | | 0.259020893 |  | |
| AIF1L | | 2.622493142 | | 0.840925247 |  | |
| LRFN1 | | 2.46028962 | | 0.791551782 |  | |
| SNAP25 | | 0.976122853 | | 0.315526499 |  | |
| GLO1 | | 80.51732949 | | 26.04739961 |  | |
| CHDH | | 5.348631894 | | 1.744262109 |  | |
| SLAIN1 | | 6.94825654 | | 2.269750477 |  | |
| NCCRP1 | | 7.138976616 | | 2.333658542 |  | |
| KCNAB2 | | 1.116843315 | | 0.367139434 |  | |
| TSPAN33 | | 5.699694627 | | 1.877358291 |  | |
| LOC105378862 | | 0.369779047 | | 0.122156115 |  | |
| SRGAP3 | | 0.716562396 | | 0.236715626 |  | |
| STON1 | | 0.458203045 | | 0.151366889 |  | |
| LOC105371727 | | 0.668667811 | | 0.220893701 |  | |
| DHRS4L1 | | 1.080694103 | | 0.357006149 |  | |
| HIST1H3A | | 2.201328571 | | 0.727206555 |  | |
| DACT2 | | 1.95891596 | | 0.649856295 |  | |
| KLF15 | | 0.858081192 | | 0.28531893 |  | |
| MAL | | 285.1843429 | | 94.93883043 |  | |
| ZBTB12 | | 2.787660994 | | 0.92960913 |  | |
| CEP126 | | 0.554706057 | | 0.185551545 |  | |
| NYNRIN | | 1.708222006 | | 0.57154406 |  | |
| HSPD1 | | 319.0299854 | | 107.0495986 |  | |
| PAF1 | | 40.6577405 | | 13.70795475 |  | |
| TNFSF13B | | 0.4417492 | | 0.148971616 |  | |
| RARRES2 | | 1.592324041 | | 0.536981358 |  | |
| SSPO | | 0.699986343 | | 0.236968657 |  | |
| DMBT1 | | 0.475711992 | | 0.162187841 |  | |
| GNG7 | | 2.640987859 | | 0.900410595 |  | |
| ZBTB47 | | 4.11105206 | | 1.403793442 |  | |
| LAMB3 | | 1.302149321 | | 0.444683937 |  | |
| MDK | | 81.26945685 | | 27.79328624 |  | |
| TWSG1 | | 18.96623906 | | 6.492486957 |  | |
| HIST4H4 | | 1.6109237 | | 0.551876819 |  | |
| PSPN | | 1.40913071 | | 0.482745753 |  | |
| MVB12B | | 1.259941147 | | 0.432935891 |  | |
| LOC107983970 | | 3.392604445 | | 1.169898681 |  | |
| GLI2 | | 1.7502491 | | 0.603965096 |  | |
| CERS1 | | 3.854042105 | | 1.330186717 |  | |
| NTN5 | | 1.38098328 | | 0.479016677 |  | |
| PPL | | 1.673600769 | | 0.581424323 |  | |
| DAG1 | | 49.25070635 | | 17.11286944 |  | |
| LEMD1 | | 2.787821152 | | 0.972117853 |  | |
| NRROS | | 1.212713117 | | 0.422875072 |  | |
| APOE | | 93.87215211 | | 32.82624593 |  | |
| NRP2 | | 0.945242094 | | 0.331184553 |  | |
| BOC | | 1.353110223 | | 0.474089344 |  | |
| CYGB | | 8.66183755 | | 3.037229035 |  | |
| PI4K2B | | 6.594204748 | | 2.320457351 |  | |
| SCML2 | | 1.493159387 | | 0.528082226 |  | |
| LDB2 | | 2.807146877 | | 0.994173159 |  | |
| AKR1C1 | | 1.464660766 | | 0.519689804 |  | |
| CFP | | 0.863674408 | | 0.307261148 |  | |
| SLC26A10 | | 0.397957075 | | 0.141577366 |  | |
| LOC105372280 | | 2.266379652 | | 0.806288119 |  | |
| APLP1 | | 7.686627941 | | 2.746821405 |  | |
| GPRC5B | | 9.061234337 | | 3.277355439 |  | |
| SLC16A8 | | 3.855427846 | | 1.398502816 |  | |
| EFNA3 | | 2.276065035 | | 0.829363705 |  | |
| GALNT12 | | 0.822509912 | | 0.300316884 |  | |
| UTRN | | 17.74386386 | | 6.479351466 |  | |
| CRMP1 | | 0.89987196 | | 0.329476185 |  | |
| TTC36 | | 2.497972996 | | 0.920878404 |  | |
| CGN | | 0.692165886 | | 0.255650408 |  | |
| VAMP2 | | 19.81995228 | | 7.340540743 |  | |
| SLC12A5 | | 0.98585659 | | 0.365883674 |  | |
| PRODH | | 113.0667443 | | 42.01872759 |  | |
| VLDLR | | 30.54390358 | | 11.35141436 |  | |
| ARHGEF6 | | 1.129537345 | | 0.420320268 |  | |
| SOX15 | | 1.639942411 | | 0.611656876 |  | |
| DGCR6 | | 23.69057478 | | 8.849576638 |  | |
| GJB1 | | 3.096454443 | | 1.161990363 |  | |
| FBLN1 | | 10.35976449 | | 3.896284052 |  | |
| OSR2 | | 1.49153989 | | 0.561481449 |  | |
| PPP1R3E | | 1.607777909 | | 0.607003113 |  | |
| SPON1 | | 2.181620297 | | 0.829748624 |  | |
| SESN3 | | 15.22142401 | | 5.815691833 |  | |
| PLEKHO1 | | 2.703041145 | | 1.041771232 |  | |
| LOC100996643 | | 0.452817149 | | 0.174518942 |  | |
| SMIM22 | | 0.714578558 | | 0.275403645 |  | |
| CDC42EP5 | | 0.910426014 | | 0.350884644 |  | |
| GPD1 | | 0.478394467 | | 0.184376621 |  | |
| VMO1 | | 0.917220238 | | 0.353503186 |  | |
| LOC107986873 | | 0.604463173 | | 0.232964395 |  | |
| SH3BP1 | | 9.103553288 | | 3.508574017 |  | |
| LOC107985115 | | 0.538561867 | | 0.207565564 |  | |
| AS3MT | | 2.510960113 | | 0.96774184 |  | |
| FOXQ1 | | 7.123229668 | | 2.745343245 |  | |
| SYNE4 | | 0.5402528 | | 0.208217261 |  | |
| HRASLS2 | | 0.489590089 | | 0.188691493 |  | |
| HIST1H4I | | 1.195856873 | | 0.460891722 |  | |
| NRTN | | 2.197683988 | | 0.847002999 |  | |
| MEF2B | | 1.524696908 | | 0.587629005 |  | |
| APC2 | | 1.95534678 | | 0.756364976 |  | |
| NTN4 | | 6.772662896 | | 2.625956616 |  | |
| GUCY1B3 | | 9.712992971 | | 3.767300065 |  | |
| COMMD10 | | 3.58388065 | | 1.395869461 |  | |
| PROCR | | 45.32007447 | | 17.65819879 |  | |
| MMRN2 | | 0.802221461 | | 0.313796496 |  | |
| ZCCHC24 | | 4.888395264 | | 1.912829891 |  | |
| PDZK1 | | 1.698591429 | | 0.667001058 |  | |
| PLXNA4 | | 2.090807054 | | 0.822599618 |  | |
| CLMN | | 0.721383027 | | 0.286714453 |  | |
| PNPLA7 | | 1.57900584 | | 0.627577554 |  | |
| VPS25 | | 74.6530637 | | 29.76395207 |  | |
| BOLA2-SMG1P6 | | 4.090719004 | | 1.633922746 |  | |
| KHDRBS3 | | 3.033674494 | | 1.212503362 |  | |
| SLC25A15 | | 31.10437307 | | 12.48262741 |  | |
| LOC107984115 | | 0.567645135 | | 0.228286395 |  | |
| FAM107A | | 1.405627346 | | 0.565292609 |  | |
| ARNTL2 | | 2.268755583 | | 0.912745132 |  | |
| SIX1 | | 1.838809817 | | 0.740422751 |  | |
| PRKAR1A | | 80.43513621 | | 32.39702237 |  | |
| GIT1 | | 41.50089817 | | 16.75063567 |  | |
| NPIPB6 | | 0.554668571 | | 0.223952878 |  | |
| LOC105378592 | | 2.012814934 | | 0.814540812 |  | |
| SLC48A1 | | 24.65554305 | | 9.981822625 |  | |
| MXRA8 | | 5.208117157 | | 2.108779107 |  | |
| TRANK1 | | 2.75257689 | | 1.115648994 |  | |
| DPEP1 | | 0.602384194 | | 0.244382254 |  | |
| SLC43A3 | | 2.991446867 | | 1.214688674 |  | |
| TMEM221 | | 0.649563016 | | 0.265072441 |  | |
| FLT4 | | 2.558625961 | | 1.04464896 |  | |
| DUSP9 | | 5.544471983 | | 2.265325103 |  | |
| ADAM11 | | 1.179562614 | | 0.482674256 |  | |
| 1-Mar | | 1.715632061 | | 0.703875913 |  | |
| GDF1 | | 2.214636135 | | 0.908960923 |  | |
| ATP2B4 | | 5.406828214 | | 2.22402646 |  | |
| CANT1 | | 15.77693475 | | 6.50712047 |  | |
| STX1B | | 0.646881642 | | 0.267555172 |  | |
| AK5 | | 1.255305381 | | 0.519908345 |  | |
| PCSK9 | | 20.29464738 | | 8.457549442 |  | |
| IRS2 | | 9.968891648 | | 4.158870525 |  | |
| FNTB | | 9.182093652 | | 3.831227156 |  | |
| FAM90A1 | | 0.703445219 | | 0.293705509 |  | |
| CATSPERG | | 2.208963638 | | 0.923042909 |  | |
| DNAH1 | | 2.487755587 | | 1.040881596 |  | |
| COL26A1 | | 0.983830432 | | 0.412146986 |  | |
| NDRG1 | | 15.9447583 | | 6.684564336 |  | |
| KCNE3 | | 4.514969825 | | 1.893785568 |  | |
| LOC100130357 | | 0.703852129 | | 0.295930477 |  | |
| PLAC9 | | 1.205333698 | | 0.506775445 |  | |
| AMBP | | 2.159880962 | | 0.911712821 |  | |
| MCAM | | 2.943583268 | | 1.245366688 |  | |
| G3BP1 | | 19.42392102 | | 8.225679151 |  | |
| EFNB3 | | 10.5840206 | | 4.485306413 |  | |
| RTKN2 | | 2.8850529 | | 1.222701537 |  | |
| LCTL | | 1.548170199 | | 0.656343355 |  | |
| TREH | | 0.795517876 | | 0.337258056 |  | |
| PRAP1 | | 1.898185512 | | 0.804731578 |  | |
| OBSL1 | | 23.49828772 | | 9.967661827 |  | |
| SAMD8 | | 9.553433084 | | 4.055022997 |  | |
| CIART | | 1.052456057 | | 0.447585137 |  | |
| KCNAB3 | | 1.985087175 | | 0.844999019 |  | |
| SRCIN1 | | 0.850445795 | | 0.362636762 |  | |
| CYS1 | | 18.57851688 | | 7.923359835 |  | |
| ADAM8 | | 1.378996825 | | 0.588710945 |  | |
| LOC107986554 | | 1.678206695 | | 0.716590546 |  | |
| LAS1L | | 15.2284823 | | 6.510566232 |  | |
| C15orf65 | | 1.765161075 | | 0.755895111 |  | |
| PRR4 | | 1.17677405 | | 0.503930074 |  | |
| LOC107985330 | | 1.035414297 | | 0.443395572 |  | |
| ARL10 | | 4.120063487 | | 1.766317616 |  | |
| CHST11 | | 2.940575007 | | 1.26143273 |  | |
| UBFD1 | | 21.23947343 | | 9.117979147 |  | |
| SNCG | | 4.642511345 | | 1.995709514 |  | |
| CTDSP1 | | 36.40886798 | | 15.67528327 |  | |
| FZD2 | | 11.19974185 | | 4.82252768 |  | |
| FLRT3 | | 17.67430336 | | 7.626419438 |  | |
| PLA2G3 | | 2.52463222 | | 1.089772507 |  | |
| THEMIS2 | | 1.353606959 | | 0.584292491 |  | |
| AMN | | 0.895827346 | | 0.3866892 |  | |
| LOC105371303 | | 2.518489038 | | 1.091973984 |  | |
| DEPTOR | | 1.221897307 | | 0.532688925 |  | |
| GPT | | 6.064327197 | | 2.6459261 |  | |
| TNNT2 | | 3.304458871 | | 1.443370142 |  | |
| GRK5 | | 1.729738244 | | 0.755540509 |  | |
| FAM149A | | 13.98623727 | | 6.12288777 |  | |
| RGAG4 | | 1.495624943 | | 0.655027799 |  | |
| COLQ | | 1.035340879 | | 0.45344059 |  | |
| PHLDB2 | | 10.7962825 | | 4.740808328 |  | |
| REP15 | | 0.906429412 | | 0.399250658 |  | |
| HOXD3 | | 2.244398043 | | 0.988579346 |  | |
| ARTN | | 1.327704604 | | 0.58480774 |  | |
| PEX7 | | 6.947552886 | | 3.064764288 |  | |
| HSPA12A | | 22.98024254 | | 10.14940011 |  | |
| PCOLCE2 | | 2.470259352 | | 1.092063637 |  | |
| GAMT | | 25.22070482 | | 11.15015794 |  | |
| NOTCH2 | | 13.9395105 | | 6.171701101 |  | |
| C12orf49 | | 12.50498937 | | 5.557246909 |  | |
| RBX1 | | 77.40731379 | | 34.44478863 |  | |
| SMTNL2 | | 36.1056474 | | 16.08204598 |  | |
| LOC101929097 | | 1.06632088 | | 0.475857257 |  | |
| TRABD2B | | 1.641789185 | | 0.733848586 |  | |
| PLPP2 | | 1.655379958 | | 0.740897627 |  | |
| FOXJ1 | | 3.071524341 | | 1.377497914 |  | |
| HSPG2 | | 9.668635759 | | 4.340385248 |  | |
| FRAS1 | | 6.53861992 | | 2.943366555 |  | |
| ERICH5 | | 3.663436806 | | 1.650289714 |  | |
| RADIL | | 1.306101625 | | 0.588568357 |  | |
| KCTD15 | | 9.868486357 | | 4.448807497 |  | |
| VGF | | 5.146218803 | | 2.326069049 |  | |
| CRIM1 | | 62.69852139 | | 28.37811663 |  | |
| PDZD3 | | 1.575416734 | | 0.71319178 |  | |
| PHF19 | | 32.19237598 | | 14.57904894 |  | |
| SCGN | | 1.691844293 | | 0.767116226 |  | |
| MT1G | | 10.19677136 | | 4.631677305 |  | |
| IRF5 | | 2.264294822 | | 1.028838233 |  | |
| RGS19 | | 3.805538576 | | 1.7299321 |  | |
| LHX2 | | 1.19041216 | | 0.541140805 |  | |
| C14orf132 | | 6.361135837 | | 2.894768796 |  | |
| SSC4D | | 9.434881705 | | 4.294647977 |  | |
| CCDC78 | | 7.306625498 | | 3.326949721 |  | |
| FECH | | 6.018719049 | | 2.741412839 |  | |
| MAST1 | | 2.531550215 | | 1.154647824 |  | |
| TP73 | | 1.161443725 | | 0.530522845 |  | |
| FHL1 | | 2.195992941 | | 1.003811957 |  | |
| KCNK6 | | 2.241961129 | | 1.028652052 |  | |
| S100A1 | | 39.23108461 | | 18.08650848 |  | |
| FTH1 | | 4370.127095 | | 2014.769349 |  | |
| CPNE2 | | 28.76628898 | | 13.28375888 |  | |
| CARD9 | | 2.431519312 | | 1.12454975 |  | |
| SH2B2 | | 4.812643538 | | 2.225792352 |  | |
| LOC102725180 | | 1.855746405 | | 0.858261395 |  | |
| UHRF1 | | 15.36843059 | | 7.110360959 |  | |
| TFEB | | 6.765270269 | | 3.138298276 |  | |
| HAP1 | | 2.024506042 | | 0.939200637 |  | |
| NPTXR | | 1.788129924 | | 0.830899665 |  | |
| CADM4 | | 23.38405787 | | 10.86710384 |  | |
| ABAT | | 5.029834805 | | 2.340487262 |  | |
| MSRB1 | | 27.5125813 | | 12.80741786 |  | |
| TMEM37 | | 6.014622923 | | 2.822008416 |  | |
| DSCAML1 | | 5.214258081 | | 2.452546955 |  | |
| NME4 | | 35.84105676 | | 16.88542839 |  | |
| SERPINF1 | | 3.223014989 | | 1.520271238 |  | |
| ALKBH8 | | 3.844070694 | | 1.816973349 |  | |
| TNFRSF11B | | 48.81722915 | | 23.18800083 |  | |
| ADAMTS2 | | 9.062472746 | | 4.325949446 |  | |
| USH1C | | 9.764653866 | | 4.663026963 |  | |
| IFI27L2 | | 15.79111502 | | 7.546649609 |  | |
| C4orf46 | | 5.119695168 | | 2.448337626 |  | |
| MACROD1 | | 13.66324411 | | 6.537813009 |  | |
| OXCT1 | | 24.80949271 | | 11.88629129 |  | |
| NDRG2 | | 6.608936799 | | 3.166703561 |  | |
| YJEFN3 | | 13.14170627 | | 6.296911184 |  | |
| NES | | 9.685766501 | | 4.643192483 |  | |
| FBL | | 259.0869251 | | 124.3233991 |  | |
| GPX3 | | 8.459051926 | | 4.067153526 |  | |
| USP13 | | 7.055600771 | | 3.396411202 |  | |
| CDT1 | | 27.29235965 | | 13.19614841 |  | |
| AGGF1 | | 9.943814669 | | 4.81573416 |  | |
| PERP | | 35.89516456 | | 17.40182297 |  | |
| ZNF467 | | 6.169713716 | | 2.996937014 |  | |
| OAF | | 13.38648948 | | 6.502484176 |  | |
| EPHA4 | | 7.608649194 | | 3.705482603 |  | |
| THBS1 | | 6.92532314 | | 3.375586842 |  | |
| RAB15 | | 17.11626771 | | 8.348861505 |  | |
| ARPIN | | 6.285559872 | | 3.066454891 |  | |
| RAPGEF3 | | 20.41936667 | | 9.962889611 |  | |
| ARFGAP3 | | 17.51855041 | | 8.547564798 |  | |
| MARCKSL1 | | 84.13105744 | | 41.0494599 |  | |
| C12orf57 | | 102.3707063 | | 50.05900047 |  | |
| ENOX1 | | 5.368947082 | | 2.628325942 |  | |
| LAGE3 | | 21.13646569 | | 10.36237412 |  | |
| ADCY6 | | 27.44844277 | | 13.46477079 |  | |
| SLC44A2 | | 26.57870626 | | 13.05622328 |  | |
| KIAA0101 | | 30.30725118 | | 14.94540185 |  | |
| SLC15A4 | | 11.76261647 | | 5.803528283 |  | |
| KIF2A | | 17.47311028 | | 8.640183481 |  | |
| **TGFBI** | | 0.473226784 | | 0.020264995 |  | |
| ITGAX | | 0.250884991 | | 0.012086607 |  | |
| NTN3 | | 0.519850504 | | 0.02862201 |  | |
| GMPR | | 0.537627027 | | 0.034534212 |  | |
| SIK1 | | 0.141032844 | | 0.012078902 |  | |
| TNFRSF13C | | 1.973889377 | | 0.182580232 |  | |
| KRT15 | | 0.707021713 | | 0.066058469 |  | |
| LOC728392 | | 0.790296125 | | 0.076146433 |  | |
| SBK2 | | 2.633732398 | | 0.253764787 |  | |
| OLFM2 | | 0.840081062 | | 0.084462574 |  | |
| PALM3 | | 1.108714654 | | 0.115488241 |  | |
| GS1-259H13.2 | | 1.295829998 | | 0.13620602 |  | |
| DISC1 | | 0.896683735 | | 0.095334696 |  | |
| GFAP | | 0.163720758 | | 0.018028326 |  | |
| LAPTM5 | | 0.230451585 | | 0.025376479 |  | |
| LOC390937 | | 0.620198098 | | 0.068293928 |  | |
| PADI3 | | 1.659251411 | | 0.189477708 |  | |
| OPRL1 | | 0.376824257 | | 0.043569223 |  | |
| GALNT9 | | 0.271869151 | | 0.034926766 |  | |
| SCT | | 0.765513915 | | 0.098344831 |  | |
| CYP2A6 | | 0.752494971 | | 0.0966723 |  | |
| LOC107987205 | | 0.196914572 | | 0.025297424 |  | |
| PRR29 | | 0.142363913 | | 0.018289354 |  | |
| NOTCH3 | | 6.998921134 | | 1.014178786 |  | |
| SLC18A3 | | 2.40736201 | | 0.352334581 |  | |
| LOC107987175 | | 0.696359841 | | 0.107352809 |  | |
| LSMEM2 | | 0.1871688 | | 0.028854473 |  | |
| TRIML2 | | 0.179689345 | | 0.027701419 |  | |
| S1PR5 | | 0.150070222 | | 0.023135251 |  | |
| WNT11 | | 1.804836886 | | 0.282586257 |  | |
| ITGB4 | | 0.501500858 | | 0.082835138 |  | |
| MMP9 | | 0.441961943 | | 0.073000829 |  | |
| UBA7 | | 0.31162786 | | 0.051472966 |  | |
| VPREB3 | | 1.134530879 | | 0.194336111 |  | |
| IDH2 | | 89.70687423 | | 15.44279939 |  | |
| HIST1H1E | | 1.597014804 | | 0.289647452 |  | |
| TESC | | 1.224274044 | | 0.222044189 |  | |
| ACOT11 | | 0.880946643 | | 0.160826725 |  | |
| DCST2 | | 0.742494031 | | 0.137357984 |  | |
| HIST1H4J | | 1.657179936 | | 0.319344452 |  | |
| PYCARD | | 0.381601589 | | 0.07353598 |  | |
| GYLTL1B | | 1.112704747 | | 0.214422152 |  | |
| ZNF853 | | 0.229376383 | | 0.044201643 |  | |
| PLEKHG6 | | 0.188364003 | | 0.036298411 |  | |
| PRR25 | | 0.243985135 | | 0.047016801 |  | |
| CRYM | | 8.088717589 | | 1.567839918 |  | |
| LRRC58 | | 8.235427193 | | 1.620289654 |  | |
| CYP2E1 | | 1.548324985 | | 0.306892508 |  | |
| GYG2 | | 0.400098697 | | 0.081158356 |  | |
| GLI1 | | 1.679230204 | | 0.343087208 |  | |
| RTN4RL2 | | 0.863674408 | | 0.17923567 |  | |
| CCDC188 | | 0.973038279 | | 0.204554103 |  | |
| CDHR5 | | 0.464998325 | | 0.097752902 |  | |
| TRPV4 | | 2.479663299 | | 0.523660178 |  | |
| SERF1B | | 1.124390487 | | 0.23908859 |  | |
| POP7 | | 34.26112832 | | 7.318881422 |  | |
| TRPC6 | | 2.869497088 | | 0.62850356 |  | |
| TMEM240 | | 0.462141047 | | 0.101778536 |  | |
| NTRK1 | | 0.194429962 | | 0.042819821 |  | |
| RASSF5 | | 0.283944747 | | 0.062533897 |  | |
| LOC107985555 | | 13.82709509 | | 3.075477722 |  | |
| PITPNM3 | | 0.464061365 | | 0.103337024 |  | |
| TFCP2L1 | | 8.909371919 | | 2.03208568 |  | |
| PAK3 | | 0.293834457 | | 0.06733539 |  | |
| TERT | | 0.963980485 | | 0.22291495 |  | |
| CMTM2 | | 1.387478968 | | 0.320846542 |  | |
| RAB42 | | 0.364710718 | | 0.084337259 |  | |
| BIRC7 | | 1.778924514 | | 0.415521289 |  | |
| PALM | | 1.050882072 | | 0.246532334 |  | |
| TMEM121 | | 0.797800407 | | 0.187160483 |  | |
| NAV2 | | 2.068634823 | | 0.506129879 |  | |
| NPTX1 | | 1.1257668 | | 0.282282405 |  | |
| EFHD1 | | 2.067269896 | | 0.522002385 |  | |
| SEMA3F | | 6.626903655 | | 1.681939106 |  | |
| TRIM73 | | 0.651645129 | | 0.167432437 |  | |
| HIST1H4E | | 0.586826317 | | 0.150778017 |  | |
| SULT1A2 | | 0.256502634 | | 0.06590529 |  | |
| OSBPL6 | | 0.21219987 | | 0.054522223 |  | |
| FAM132B | | 0.305044497 | | 0.078377542 |  | |
| C1QTNF9B-AS1 | | 1.650996429 | | 0.424203824 |  | |
| LBH | | 0.286293784 | | 0.07355977 |  | |
| QRFP | | 0.237375023 | | 0.060990679 |  | |
| CHST2 | | 2.228199998 | | 0.577089466 |  | |
| NUPR1 | | 9.784084829 | | 2.555455929 |  | |
| FOXO6 | | 1.16738409 | | 0.307261148 |  | |
| SEMA4A | | 7.001338624 | | 1.894777079 |  | |
| GRIN2C | | 0.484816412 | | 0.131301184 |  | |
| MYLK2 | | 0.369646652 | | 0.101760316 |  | |
| C2orf48 | | 1.628427602 | | 0.448291107 |  | |
| LTC4S | | 1.54786072 | | 0.426111787 |  | |
| DHRS3 | | 2.886137259 | | 0.798601478 |  | |
| PPM1H | | 0.419598903 | | 0.116795173 |  | |
| KCNK5 | | 0.916201913 | | 0.255441795 |  | |
| ADGRB2 | | 1.494370327 | | 0.421671034 |  | |
| HOOK1 | | 10.03489366 | | 2.843621398 |  | |
| MADCAM1 | | 0.911610693 | | 0.25888301 |  | |
| AIFM3 | | 0.485760442 | | 0.14041164 |  | |
| PTGER1 | | 0.415169639 | | 0.120006993 |  | |
| SERPINF2 | | 2.239394565 | | 0.647308914 |  | |
| SPEG | | 1.108313764 | | 0.320981262 |  | |
| SCARF2 | | 5.921398352 | | 1.715658061 |  | |
| INHBE | | 0.635467645 | | 0.186600943 |  | |
| NFATC4 | | 1.513703041 | | 0.448762992 |  | |
| EMP1 | | 1.025690363 | | 0.30408334 |  | |
| ARHGEF10L | | 3.750655561 | | 1.113907842 |  | |
| FAM69B | | 14.44325577 | | 4.296162577 |  | |
| TMEM238 | | 6.015412969 | | 1.803186646 |  | |
| SV2A | | 9.652247538 | | 2.921972806 |  | |
| REPS2 | | 0.259631108 | | 0.078621456 |  | |
| GPR161 | | 1.774645416 | | 0.538092487 |  | |
| CERS4 | | 0.857796523 | | 0.260473409 |  | |
| PPP2CA | | 66.7132488 | | 20.38874769 |  | |
| CORO1A | | 0.530281691 | | 0.16349946 |  | |
| TMEM130 | | 0.601504952 | | 0.18545942 |  | |
| DACT3 | | 0.817113653 | | 0.251937119 |  | |
| ATP2A1 | | 2.940603933 | | 0.916309051 |  | |
| NDRG4 | | 9.878160603 | | 3.080808562 |  | |
| HECW1 | | 1.107367901 | | 0.3462052 |  | |
| KIF7 | | 3.275072512 | | 1.024423566 |  | |
| XKR5 | | 1.333879974 | | 0.41804861 |  | |
| FAM71F2 | | 0.342348984 | | 0.107509704 |  | |
| GNMT | | 1.850466257 | | 0.581111929 |  | |
| ITGB7 | | 0.535262012 | | 0.168785817 |  | |
| 1-Sep | | 1.665117164 | | 0.525066519 |  | |
| ARHGAP6 | | 6.715421748 | | 2.121978856 |  | |
| ASB4 | | 1.311356299 | | 0.416216722 |  | |
| EVC | | 2.286445287 | | 0.727512425 |  | |
| ATP6V1G2 | | 1.058093365 | | 0.336875526 |  | |
| B4GALNT4 | | 7.210436493 | | 2.301879834 |  | |
| RBFOX3 | | 0.941908035 | | 0.301039349 |  | |
| FAM65C | | 0.80953995 | | 0.259020893 |  | |
| AIF1L | | 2.622493142 | | 0.840925247 |  | |
| LRFN1 | | 2.46028962 | | 0.791551782 |  | |
| SNAP25 | | 0.976122853 | | 0.315526499 |  | |
| GLO1 | | 80.51732949 | | 26.04739961 |  | |
| CHDH | | 5.348631894 | | 1.744262109 |  | |
| SLAIN1 | | 6.94825654 | | 2.269750477 |  | |
| NCCRP1 | | 7.138976616 | | 2.333658542 |  | |
| KCNAB2 | | 1.116843315 | | 0.367139434 |  | |
| TSPAN33 | | 5.699694627 | | 1.877358291 |  | |
| LOC105378862 | | 0.369779047 | | 0.122156115 |  | |
| SRGAP3 | | 0.716562396 | | 0.236715626 |  | |
| STON1 | | 0.458203045 | | 0.151366889 |  | |
| LOC105371727 | | 0.668667811 | | 0.220893701 |  | |
| DHRS4L1 | | 1.080694103 | | 0.357006149 |  | |
| HIST1H3A | | 2.201328571 | | 0.727206555 |  | |
| DACT2 | | 1.95891596 | | 0.649856295 |  | |
| KLF15 | | 0.858081192 | | 0.28531893 |  | |
| MAL | | 285.1843429 | | 94.93883043 |  | |
| ZBTB12 | | 2.787660994 | | 0.92960913 |  | |
| CEP126 | | 0.554706057 | | 0.185551545 |  | |
| NYNRIN | | 1.708222006 | | 0.57154406 |  | |
| HSPD1 | | 319.0299854 | | 107.0495986 |  | |
| PAF1 | | 40.6577405 | | 13.70795475 |  | |
| TNFSF13B | | 0.4417492 | | 0.148971616 |  | |
| RARRES2 | | 1.592324041 | | 0.536981358 |  | |
| SSPO | | 0.699986343 | | 0.236968657 |  | |
| DMBT1 | | 0.475711992 | | 0.162187841 |  | |
| GNG7 | | 2.640987859 | | 0.900410595 |  | |
| ZBTB47 | | 4.11105206 | | 1.403793442 |  | |
| LAMB3 | | 1.302149321 | | 0.444683937 |  | |
| MDK | | 81.26945685 | | 27.79328624 |  | |
| TWSG1 | | 18.96623906 | | 6.492486957 |  | |
| HIST4H4 | | 1.6109237 | | 0.551876819 |  | |
| PSPN | | 1.40913071 | | 0.482745753 |  | |
| MVB12B | | 1.259941147 | | 0.432935891 |  | |
| LOC107983970 | | 3.392604445 | | 1.169898681 |  | |
| GLI2 | | 1.7502491 | | 0.603965096 |  | |
| CERS1 | | 3.854042105 | | 1.330186717 |  | |
| NTN5 | | 1.38098328 | | 0.479016677 |  | |
| PPL | | 1.673600769 | | 0.581424323 |  | |
| DAG1 | | 49.25070635 | | 17.11286944 |  | |
| LEMD1 | | 2.787821152 | | 0.972117853 |  | |
| NRROS | | 1.212713117 | | 0.422875072 |  | |
| APOE | | 93.87215211 | | 32.82624593 |  | |
| NRP2 | | 0.945242094 | | 0.331184553 |  | |
| BOC | | 1.353110223 | | 0.474089344 |  | |
| CYGB | | 8.66183755 | | 3.037229035 |  | |
| PI4K2B | | 6.594204748 | | 2.320457351 |  | |
| SCML2 | | 1.493159387 | | 0.528082226 |  | |
| LDB2 | | 2.807146877 | | 0.994173159 |  | |
| AKR1C1 | | 1.464660766 | | 0.519689804 |  | |
| CFP | | 0.863674408 | | 0.307261148 |  | |
| SLC26A10 | | 0.397957075 | | 0.141577366 |  | |
| LOC105372280 | | 2.266379652 | | 0.806288119 |  | |
| APLP1 | | 7.686627941 | | 2.746821405 |  | |
| GPRC5B | | 9.061234337 | | 3.277355439 |  | |
| SLC16A8 | | 3.855427846 | | 1.398502816 |  | |
| EFNA3 | | 2.276065035 | | 0.829363705 |  | |
| GALNT12 | | 0.822509912 | | 0.300316884 |  | |
| UTRN | | 17.74386386 | | 6.479351466 |  | |
| CRMP1 | | 0.89987196 | | 0.329476185 |  | |
| TTC36 | | 2.497972996 | | 0.920878404 |  | |
| CGN | | 0.692165886 | | 0.255650408 |  | |
| VAMP2 | | 19.81995228 | | 7.340540743 |  | |
| SLC12A5 | | 0.98585659 | | 0.365883674 |  | |
| PRODH | | 113.0667443 | | 42.01872759 |  | |
| VLDLR | | 30.54390358 | | 11.35141436 |  | |
| ARHGEF6 | | 1.129537345 | | 0.420320268 |  | |
| SOX15 | | 1.639942411 | | 0.611656876 |  | |
| DGCR6 | | 23.69057478 | | 8.849576638 |  | |
| GJB1 | | 3.096454443 | | 1.161990363 |  | |
| FBLN1 | | 10.35976449 | | 3.896284052 |  | |
| OSR2 | | 1.49153989 | | 0.561481449 |  | |
| PPP1R3E | | 1.607777909 | | 0.607003113 |  | |
| SPON1 | | 2.181620297 | | 0.829748624 |  | |
| SESN3 | | 15.22142401 | | 5.815691833 |  | |
| PLEKHO1 | | 2.703041145 | | 1.041771232 |  | |
| LOC100996643 | | 0.452817149 | | 0.174518942 |  | |
| SMIM22 | | 0.714578558 | | 0.275403645 |  | |
| CDC42EP5 | | 0.910426014 | | 0.350884644 |  | |
| GPD1 | | 0.478394467 | | 0.184376621 |  | |
| VMO1 | | 0.917220238 | | 0.353503186 |  | |
| LOC107986873 | | 0.604463173 | | 0.232964395 |  | |
| SH3BP1 | | 9.103553288 | | 3.508574017 |  | |
| LOC107985115 | | 0.538561867 | | 0.207565564 |  | |
| AS3MT | | 2.510960113 | | 0.96774184 |  | |
| FOXQ1 | | 7.123229668 | | 2.745343245 |  | |
| SYNE4 | | 0.5402528 | | 0.208217261 |  | |
| HRASLS2 | | 0.489590089 | | 0.188691493 |  | |
| HIST1H4I | | 1.195856873 | | 0.460891722 |  | |
| NRTN | | 2.197683988 | | 0.847002999 |  | |
| MEF2B | | 1.524696908 | | 0.587629005 |  | |
| APC2 | | 1.95534678 | | 0.756364976 |  | |
| NTN4 | | 6.772662896 | | 2.625956616 |  | |
| GUCY1B3 | | 9.712992971 | | 3.767300065 |  | |
| COMMD10 | | 3.58388065 | | 1.395869461 |  | |
| PROCR | | 45.32007447 | | 17.65819879 |  | |
| MMRN2 | | 0.802221461 | | 0.313796496 |  | |
| ZCCHC24 | | 4.888395264 | | 1.912829891 |  | |
| PDZK1 | | 1.698591429 | | 0.667001058 |  | |
| PLXNA4 | | 2.090807054 | | 0.822599618 |  | |
| CLMN | | 0.721383027 | | 0.286714453 |  | |
| PNPLA7 | | 1.57900584 | | 0.627577554 |  | |
| VPS25 | | 74.6530637 | | 29.76395207 |  | |
| BOLA2-SMG1P6 | | 4.090719004 | | 1.633922746 |  | |
| KHDRBS3 | | 3.033674494 | | 1.212503362 |  | |
| SLC25A15 | | 31.10437307 | | 12.48262741 |  | |
| LOC107984115 | | 0.567645135 | | 0.228286395 |  | |
| FAM107A | | 1.405627346 | | 0.565292609 |  | |
| ARNTL2 | | 2.268755583 | | 0.912745132 |  | |
| SIX1 | | 1.838809817 | | 0.740422751 |  | |
| PRKAR1A | | 80.43513621 | | 32.39702237 |  | |
| GIT1 | | 41.50089817 | | 16.75063567 |  | |
| NPIPB6 | | 0.554668571 | | 0.223952878 |  | |
| LOC105378592 | | 2.012814934 | | 0.814540812 |  | |
| SLC48A1 | | 24.65554305 | | 9.981822625 |  | |
| MXRA8 | | 5.208117157 | | 2.108779107 |  | |
| TRANK1 | | 2.75257689 | | 1.115648994 |  | |
| DPEP1 | | 0.602384194 | | 0.244382254 |  | |
| SLC43A3 | | 2.991446867 | | 1.214688674 |  | |
| TMEM221 | | 0.649563016 | | 0.265072441 |  | |
| FLT4 | | 2.558625961 | | 1.04464896 |  | |
| DUSP9 | | 5.544471983 | | 2.265325103 |  | |
| ADAM11 | | 1.179562614 | | 0.482674256 |  | |
| 1-Mar | | 1.715632061 | | 0.703875913 |  | |
| GDF1 | | 2.214636135 | | 0.908960923 |  | |
| ATP2B4 | | 5.406828214 | | 2.22402646 |  | |
| CANT1 | | 15.77693475 | | 6.50712047 |  | |
| STX1B | | 0.646881642 | | 0.267555172 |  | |
| AK5 | | 1.255305381 | | 0.519908345 |  | |
| PCSK9 | | 20.29464738 | | 8.457549442 |  | |
| IRS2 | | 9.968891648 | | 4.158870525 |  | |
| FNTB | | 9.182093652 | | 3.831227156 |  | |
| FAM90A1 | | 0.703445219 | | 0.293705509 |  | |
| CATSPERG | | 2.208963638 | | 0.923042909 |  | |
| DNAH1 | | 2.487755587 | | 1.040881596 |  | |
| COL26A1 | | 0.983830432 | | 0.412146986 |  | |
| NDRG1 | | 15.9447583 | | 6.684564336 |  | |
| KCNE3 | | 4.514969825 | | 1.893785568 |  | |
| LOC100130357 | | 0.703852129 | | 0.295930477 |  | |
| PLAC9 | | 1.205333698 | | 0.506775445 |  | |
| AMBP | | 2.159880962 | | 0.911712821 |  | |
| MCAM | | 2.943583268 | | 1.245366688 |  | |
| G0S2 | 0.229733667 | | | 0 |  |  |
| CDA | 0.15363439 | | | 0 |  |  |
| GNA15 | 0.12858676 | | | 0 |  |  |
| RSPO4 | 0.108847981 | | | 0 |  |  |
| FST | 0.155306087 | | | 0 |  |  |
| AQP3 | 0.186600474 | | | 0 |  |  |
| NUDT11 | 0.18466905 | | | 0 |  |  |
| LGALS7B | 0.452420289 | | | 0 |  |  |
| KRT14 | 3.065175113 | | | 0 |  |  |
| HIST3H3 | 0.613259935 | | | 0 |  |  |
| CACNA1H | 0.109413215 | | | 0 |  |  |
| KLK14 | 0.375608695 | | | 0 |  |  |
| KRT17 | 1.358698035 | | | 0.036113921 |  |  |

| **Supplementary Table. S3 List of possible RAC1-interacting proteins in group 1 identified by Mass Spectrometry. (Group 1: 293T cells transfected with NC group)** | |
| --- | --- |
| Description | Abundances (Grouped): 1 |
| RAS-related C3 botulinum toxin substrate 1 OS=Homo sapiens OX=9606 GN=EIF1AX PE=1 SV=1 | 32156431 |
| Chromatin target of PRMT1 protein OS=Homo sapiens OX=9606 GN=CHTOP PE=1 SV=1 | 1.82E+08 |
| AP complex subunit sigma OS=Homo sapiens OX=9606 GN=AP2S1 PE=1 SV=1 | 1434603 |
| U6 snRNA-associated Sm-like protein LSm4 (Fragment) OS=Homo sapiens OX=9606 GN=LSM4 PE=1 SV=1 | 10531177 |
| 28S ribosomal protein S29, mitochondrial (Fragment) OS=Homo sapiens OX=9606 GN=DAP3 PE=1 SV=1 | 567428.4 |
| Pantothenate kinase 2, mitochondrial OS=Homo sapiens OX=9606 GN=PANK2 PE=1 SV=2 | 1129517 |
| P21-activated kinases 4 OS=Homo sapiens OX=9606 GN=ARHGEF2 PE=1 SV=1 | 34223437 |
| ATP-dependent DNA helicase Q4 (Fragment) OS=Homo sapiens OX=9606 GN=RECQL4 PE=1 SV=1 | 639739.9 |
| Mediator of RNA polymerase II transcription subunit 16 (Fragment) OS=Homo sapiens OX=9606 GN=MED16 PE=1 SV=1 | 1731932 |
| Mitochondrial GTPase 1 OS=Homo sapiens OX=9606 GN=MTG1 PE=1 SV=1 | 1170482 |
| Casein kinase I isoform alpha OS=Homo sapiens OX=9606 GN=CSNK1A1 PE=1 SV=2 | 4290789 |
| Tubulin epsilon chain OS=Homo sapiens OX=9606 GN=TUBE1 PE=1 SV=1 | 476289.1 |
| Synaptic functional regulator FMR1 OS=Homo sapiens OX=9606 GN=FMR1 PE=2 SV=1 | 10736966 |
| X-DING-CD4 (Fragment) OS=Homo sapiens OX=9606 PE=2 SV=1 | 1044950 |
| Zinc fingers and homeoboxes protein 2 OS=Homo sapiens OX=9606 GN=ZHX2 PE=1 SV=1 |  |
| Casein kinase I isoform gamma-3 OS=Homo sapiens OX=9606 GN=CSNK1G3 PE=1 SV=2 | 1032487 |
| Insulin-like growth factor 2 mRNA-binding protein 2 OS=Homo sapiens OX=9606 GN=IGF2BP2 PE=1 SV=2 | 7378210 |
| TAF6-like RNA polymerase II p300/CBP-associated factor-associated factor 65 kDa subunit 6L OS=Homo sapiens OX=9606 GN=TAF6L PE=1 SV=1 | 579259.2 |
| RNA-binding protein OS=Homo sapiens OX=9606 PE=2 SV=1 | 6973194 |
| Cilia- and flagella-associated protein 20 OS=Homo sapiens OX=9606 GN=CFAP20 PE=1 SV=1 | 9878535 |
| Spindlin-1 OS=Homo sapiens OX=9606 GN=SPIN1 PE=1 SV=3 | 794957.9 |
| MORF4 family-associated protein 1 OS=Homo sapiens OX=9606 GN=MRFAP1 PE=1 SV=1 | 738093.4 |
| Ubiquitin carboxyl-terminal hydrolase 16 OS=Homo sapiens OX=9606 GN=USP16 PE=1 SV=1 | 4966283 |
| RNA-binding protein 8A OS=Homo sapiens OX=9606 GN=RBM8A PE=1 SV=1 | 9338359 |
| General transcription factor 3C polypeptide 3 OS=Homo sapiens OX=9606 GN=GTF3C3 PE=1 SV=1 | 2961887 |
| Signal recognition particle receptor subunit beta OS=Homo sapiens OX=9606 GN=SRPRB PE=1 SV=3 | 1537999 |
| U3 small nucleolar RNA-associated protein 18 homolog OS=Homo sapiens OX=9606 GN=UTP18 PE=1 SV=3 | 1929860 |
| FACT complex subunit SPT16 OS=Homo sapiens OX=9606 GN=SUPT16H PE=1 SV=1 | 14381536 |
| Protein PRRC2C OS=Homo sapiens OX=9606 GN=PRRC2C PE=1 SV=4 | 3.91E+08 |
| AFG3-like protein 2 OS=Homo sapiens OX=9606 GN=AFG3L2 PE=1 SV=2 | 486175.3 |
| Transducin beta-like protein 2 OS=Homo sapiens OX=9606 GN=TBL2 PE=1 SV=1 | 10730535 |
| Centrosomal protein of 170 kDa protein B OS=Homo sapiens OX=9606 GN=CEP170B PE=1 SV=4 | 1366704 |
| Microtubule cross-linking factor 1 OS=Homo sapiens OX=9606 GN=MTCL1 PE=1 SV=5 | 501685.7 |
| Kinesin-like protein KIF3A OS=Homo sapiens OX=9606 GN=KIF3A PE=1 SV=4 | 423867.3 |
| 60S ribosomal protein L36 OS=Homo sapiens OX=9606 GN=RPL36 PE=1 SV=3 | 24178527 |
| RNA-splicing ligase RtcB homolog OS=Homo sapiens OX=9606 GN=RTCB PE=1 SV=1 | 3.82E+08 |
| Serine-threonine kinase receptor-associated protein OS=Homo sapiens OX=9606 GN=STRAP PE=1 SV=1 | 6039164 |
| Hepatoma-derived growth factor-related protein 3 OS=Homo sapiens OX=9606 GN=HDGFL3 PE=1 SV=1 | 1011530 |
| Peptidyl-prolyl cis-trans isomerase-like 1 OS=Homo sapiens OX=9606 GN=PPIL1 PE=1 SV=1 | 8503866 |
| Nucleolar protein 16 OS=Homo sapiens OX=9606 GN=NOP16 PE=1 SV=2 | 11211735 |
| Splicing factor 3B subunit 6 OS=Homo sapiens OX=9606 GN=SF3B6 PE=1 SV=1 | 882903.2 |
| Ribosomal RNA-processing protein 7 homolog A OS=Homo sapiens OX=9606 GN=RRP7A PE=1 SV=2 | 2921082 |
| Putative RNA-binding protein Luc7-like 2 OS=Homo sapiens OX=9606 GN=LUC7L2 PE=1 SV=2 | 2.58E+08 |
| U6 snRNA-associated Sm-like protein LSm2 OS=Homo sapiens OX=9606 GN=LSM2 PE=1 SV=1 | 2875493 |
| Nucleolar protein 58 OS=Homo sapiens OX=9606 GN=NOP58 PE=1 SV=1 | 18988716 |
| Thyroid hormone receptor-associated protein 3 OS=Homo sapiens OX=9606 GN=THRAP3 PE=1 SV=2 | 4.35E+08 |
| AP-3 complex subunit mu-1 OS=Homo sapiens OX=9606 GN=AP3M1 PE=1 SV=1 | 4957075 |
| Exosome complex exonuclease RRP44 OS=Homo sapiens OX=9606 GN=DIS3 PE=1 SV=2 | 422848.9 |
| Developmentally-regulated GTP-binding protein 1 OS=Homo sapiens OX=9606 GN=DRG1 PE=1 SV=1 | 1.33E+08 |
| Histone chaperone ASF1A OS=Homo sapiens OX=9606 GN=ASF1A PE=1 SV=1 | 3112321 |
| Voltage-dependent anion-selective channel protein 3 OS=Homo sapiens OX=9606 GN=VDAC3 PE=1 SV=1 | 717376.8 |
| Eukaryotic translation initiation factor 3 subunit L OS=Homo sapiens OX=9606 GN=EIF3L PE=1 SV=1 | 87605691 |
| Nucleotide-binding oligomerization domain-containing protein 1 OS=Homo sapiens OX=9606 GN=NOD1 PE=1 SV=1 | 20831810 |
| RuvB-like 2 OS=Homo sapiens OX=9606 GN=RUVBL2 PE=1 SV=3 | 4868067 |
| RNA transcription, translation and transport factor protein OS=Homo sapiens OX=9606 GN=RTRAF PE=1 SV=1 | 1.05E+08 |
| Sex comb on midleg-like protein 2 OS=Homo sapiens OX=9606 GN=SCML2 PE=1 SV=1 | 1334411 |
| Cyclin-dependent kinase 11A OS=Homo sapiens OX=9606 GN=CDK11A PE=1 SV=4 | 1.06E+08 |
| Serine/arginine repetitive matrix protein 2 OS=Homo sapiens OX=9606 GN=SRRM2 PE=1 SV=2 | 1.02E+08 |
| Cytosolic carboxypeptidase 1 OS=Homo sapiens OX=9606 GN=AGTPBP1 PE=1 SV=3 | 347544.4 |
| Leydig cell tumor 10 kDa protein homolog OS=Homo sapiens OX=9606 GN=C19orf53 PE=1 SV=1 | 5705855 |
| WD repeat-containing protein 3 OS=Homo sapiens OX=9606 GN=WDR3 PE=1 SV=1 | 4077936 |
| Pre-mRNA-processing factor 19 OS=Homo sapiens OX=9606 GN=PRPF19 PE=1 SV=1 | 2.25E+08 |
| XRRM domain-containing protein (Fragment) OS=Homo sapiens OX=9606 PE=2 SV=1 | 1460206 |
| RNA-binding protein NOB1 OS=Homo sapiens OX=9606 GN=NOB1 PE=1 SV=1 | 1.32E+08 |
| Activator of basal transcription 1 OS=Homo sapiens OX=9606 GN=ABT1 PE=1 SV=1 | 2002812 |
| Mediator of RNA polymerase II transcription subunit 23 OS=Homo sapiens OX=9606 GN=MED23 PE=1 SV=2 | 1049795 |
| Serine/threonine-protein kinase TAO2 OS=Homo sapiens OX=9606 GN=TAOK2 PE=1 SV=2 | 2192607 |
| Zinc finger protein 346 OS=Homo sapiens OX=9606 GN=ZNF346 PE=1 SV=1 | 20696308 |
| Protein argonaute-2 OS=Homo sapiens OX=9606 GN=AGO2 PE=1 SV=3 | 1576382 |
| Apoptotic chromatin condensation inducer in the nucleus OS=Homo sapiens OX=9606 GN=ACIN1 PE=1 SV=2 | 34666719 |
| NTF2-related export protein 1 OS=Homo sapiens OX=9606 GN=NXT1 PE=1 SV=1 | 8446265 |
| G patch domain-containing protein 8 OS=Homo sapiens OX=9606 GN=GPATCH8 PE=1 SV=2 | 7163787 |
| mRNA turnover protein 4 homolog OS=Homo sapiens OX=9606 GN=MRTO4 PE=1 SV=2 | 1326490 |
| Lariat debranching enzyme OS=Homo sapiens OX=9606 GN=DBR1 PE=1 SV=2 | 8258812 |
| Cyclin-L1 OS=Homo sapiens OX=9606 GN=CCNL1 PE=1 SV=1 | 5631970 |
| Anaphase-promoting complex subunit 7 OS=Homo sapiens OX=9606 GN=ANAPC7 PE=1 SV=4 | 1175372 |
| Probable ATP-dependent RNA helicase DDX41 OS=Homo sapiens OX=9606 GN=DDX41 PE=1 SV=2 | 52595985 |
| 18S rRNA aminocarboxypropyltransferase OS=Homo sapiens OX=9606 GN=TSR3 PE=1 SV=1 | 1910619 |
| E3 ISG15--protein ligase HERC5 OS=Homo sapiens OX=9606 GN=HERC5 PE=1 SV=2 | 552862.4 |
| rRNA methyltransferase 2, mitochondrial OS=Homo sapiens OX=9606 GN=MRM2 PE=1 SV=1 | 842265.1 |
| Carboxypeptidase A4 OS=Homo sapiens OX=9606 GN=CPA4 PE=1 SV=2 | 310712.5 |
| Multifunctional methyltransferase subunit TRM112-like protein OS=Homo sapiens OX=9606 GN=TRMT112 PE=1 SV=1 | 9747659 |
| Nuclear fragile X mental retardation-interacting protein 1 OS=Homo sapiens OX=9606 GN=NUFIP1 PE=1 SV=2 | 1550836 |
| Signal recognition particle subunit SRP68 OS=Homo sapiens OX=9606 GN=SRP68 PE=1 SV=2 | 25366572 |
| AF4/FMR2 family member 4 OS=Homo sapiens OX=9606 GN=AFF4 PE=1 SV=1 | 5969035 |
| Armadillo repeat-containing X-linked protein 3 OS=Homo sapiens OX=9606 GN=ARMCX3 PE=1 SV=1 | 13104338 |
| Translocation protein SEC63 homolog OS=Homo sapiens OX=9606 GN=SEC63 PE=1 SV=2 | 25996406 |
| CGG triplet repeat-binding protein 1 OS=Homo sapiens OX=9606 GN=CGGBP1 PE=1 SV=2 | 1661326 |
| Cytochrome b-c1 complex subunit 9 OS=Homo sapiens OX=9606 GN=UQCR10 PE=1 SV=3 | 978372.2 |
| Uncharacterized protein ORC5L (Fragment) OS=Homo sapiens OX=9606 GN=ORC5L PE=3 SV=1 | 2268476 |
| Nuclear RNA export factor 1 OS=Homo sapiens OX=9606 GN=NXF1 PE=1 SV=1 | 27930835 |
| Ribosomal protein S6 kinase beta-2 OS=Homo sapiens OX=9606 GN=RPS6KB2 PE=1 SV=2 | 8443608 |
| Eukaryotic translation initiation factor 3 subunit K OS=Homo sapiens OX=9606 GN=EIF3K PE=1 SV=1 | 12688765 |
| RNA-binding protein 27 OS=Homo sapiens OX=9606 GN=RBM27 PE=1 SV=2 | 1.02E+08 |
| Cleavage and polyadenylation specificity factor subunit 2 OS=Homo sapiens OX=9606 GN=CPSF2 PE=1 SV=2 | 45598915 |
| Ribosome-binding protein 1 OS=Homo sapiens OX=9606 GN=RRBP1 PE=1 SV=5 | 39118514 |
| Protein RCC2 OS=Homo sapiens OX=9606 GN=RCC2 PE=1 SV=2 | 1.41E+08 |
| Uncharacterized protein KIAA1522 OS=Homo sapiens OX=9606 GN=KIAA1522 PE=1 SV=2 | 1050962 |
| Calmodulin-regulated spectrin-associated protein 3 OS=Homo sapiens OX=9606 GN=CAMSAP3 PE=1 SV=2 | 5546061 |
| Very-long-chain (3R)-3-hydroxyacyl-CoA dehydratase 3 OS=Homo sapiens OX=9606 GN=HACD3 PE=1 SV=2 | 21891333 |
| Thyroid transcription factor 1-associated protein 26 OS=Homo sapiens OX=9606 GN=CCDC59 PE=1 SV=2 | 3598553 |
| Thymocyte nuclear protein 1 OS=Homo sapiens OX=9606 GN=THYN1 PE=1 SV=1 | 2311451 |
| Protein cornichon homolog 4 OS=Homo sapiens OX=9606 GN=CNIH4 PE=1 SV=1 | 800944.4 |
| Calmodulin-like protein 5 OS=Homo sapiens OX=9606 GN=CALML5 PE=1 SV=2 | 534652.2 |
| Low-density lipoprotein receptor-related protein 1B OS=Homo sapiens OX=9606 GN=LRP1B PE=1 SV=2 | 483433.7 |
| Insulin-like growth factor 2 mRNA-binding protein 1 OS=Homo sapiens OX=9606 GN=IGF2BP1 PE=1 SV=2 | 81209466 |
| Constitutive coactivator of PPAR-gamma-like protein 1 OS=Homo sapiens OX=9606 GN=FAM120A PE=1 SV=2 | 64599239 |
| G2 and S phase-expressed protein 1 OS=Homo sapiens OX=9606 GN=GTSE1 PE=1 SV=3 | 457111.3 |
| Cyclin-dependent kinase 12 OS=Homo sapiens OX=9606 GN=CDK12 PE=1 SV=2 | 73374162 |
| Mitogen-activated protein kinase kinase kinase 20 OS=Homo sapiens OX=9606 GN=MAP3K20 PE=1 SV=3 | 27064787 |
| H/ACA ribonucleoprotein complex subunit 1 OS=Homo sapiens OX=9606 GN=GAR1 PE=1 SV=1 | 78467679 |
| Non-structural maintenance of chromosomes element 4 homolog A OS=Homo sapiens OX=9606 GN=NSMCE4A PE=1 SV=2 | 510892.9 |
| Glutaminyl-peptide cyclotransferase-like protein OS=Homo sapiens OX=9606 GN=QPCTL PE=1 SV=2 | 11995029 |
| DNA helicase MCM9 OS=Homo sapiens OX=9606 GN=MCM9 PE=1 SV=4 | 4265998 |
| cDNA FLJ20354 fis, clone HEP15013 OS=Homo sapiens OX=9606 PE=2 SV=1 | 1596726 |
| Cell growth-regulating nucleolar protein OS=Homo sapiens OX=9606 GN=LYAR PE=1 SV=2 | 15037767 |
| H/ACA ribonucleoprotein complex subunit 2 OS=Homo sapiens OX=9606 GN=NHP2 PE=1 SV=1 | 24165864 |
| 39S ribosomal protein L16, mitochondrial OS=Homo sapiens OX=9606 GN=MRPL16 PE=1 SV=1 | 2270927 |
| 39S ribosomal protein L22, mitochondrial OS=Homo sapiens OX=9606 GN=MRPL22 PE=1 SV=1 | 6667291 |
| Box C/D snoRNA protein 1 OS=Homo sapiens OX=9606 GN=ZNHIT6 PE=1 SV=1 |  |
| SAFB-like transcription modulator OS=Homo sapiens OX=9606 GN=SLTM PE=1 SV=2 | 3904246 |
| Pre-mRNA-splicing factor RBM22 OS=Homo sapiens OX=9606 GN=RBM22 PE=1 SV=1 | 529544.8 |
| RNA-binding protein 28 OS=Homo sapiens OX=9606 GN=RBM28 PE=1 SV=3 | 2326081 |
| Poly(A) RNA polymerase, mitochondrial OS=Homo sapiens OX=9606 GN=MTPAP PE=1 SV=1 | 1578755 |
| Histone chaperone ASF1B OS=Homo sapiens OX=9606 GN=ASF1B PE=1 SV=1 | 39998754 |
| ATP-dependent RNA helicase DDX18 OS=Homo sapiens OX=9606 GN=DDX18 PE=1 SV=2 | 19328152 |
| ATPase family AAA domain-containing protein 3A OS=Homo sapiens OX=9606 GN=ATAD3A PE=1 SV=2 | 59493661 |
| U3 small nucleolar ribonucleoprotein protein IMP3 OS=Homo sapiens OX=9606 GN=IMP3 PE=1 SV=1 | 8238033 |
| SPATS2-like protein OS=Homo sapiens OX=9606 GN=SPATS2L PE=1 SV=2 | 1765865 |
| Gamma-taxilin OS=Homo sapiens OX=9606 GN=TXLNG PE=1 SV=2 | 4580247 |
| Probable ATP-dependent RNA helicase DDX28 OS=Homo sapiens OX=9606 GN=DDX28 PE=1 SV=2 | 19222212 |
| Zinc finger CCHC domain-containing protein 3 OS=Homo sapiens OX=9606 GN=ZCCHC3 PE=1 SV=2 | 8763868 |
| Obg-like ATPase 1 OS=Homo sapiens OX=9606 GN=OLA1 PE=1 SV=2 | 970430.9 |
| Structural maintenance of chromosomes protein 4 OS=Homo sapiens OX=9606 GN=SMC4 PE=1 SV=2 | 2844603 |
| Ribosome biogenesis protein SLX9 homolog OS=Homo sapiens OX=9606 GN=SLX9 PE=1 SV=2 | 31979497 |
| Phenylalanine--tRNA ligase beta subunit OS=Homo sapiens OX=9606 GN=FARSB PE=1 SV=3 | 4311702 |
| Keratin, type II cuticular Hb4 OS=Homo sapiens OX=9606 GN=KRT84 PE=2 SV=2 | 2588316 |
| Double-stranded RNA-specific editase B2 OS=Homo sapiens OX=9606 GN=ADARB2 PE=1 SV=1 | 1831227 |
| Lymphoid-specific helicase OS=Homo sapiens OX=9606 GN=HELLS PE=1 SV=1 | 20404301 |
| Eukaryotic translation initiation factor 4E transporter OS=Homo sapiens OX=9606 GN=EIF4ENIF1 PE=1 SV=2 | 978119 |
| Nucleolar RNA helicase 2 OS=Homo sapiens OX=9606 GN=DDX21 PE=1 SV=5 | 79332875 |
| Something about silencing protein 10 OS=Homo sapiens OX=9606 GN=UTP3 PE=1 SV=1 | 4447423 |
| Exosome complex component RRP40 OS=Homo sapiens OX=9606 GN=EXOSC3 PE=1 SV=3 | 3346106 |
| Cell death regulator Aven OS=Homo sapiens OX=9606 GN=AVEN PE=1 SV=1 | 8336173 |
| Regulation of nuclear pre-mRNA domain-containing protein 1B OS=Homo sapiens OX=9606 GN=RPRD1B PE=1 SV=1 | 532822.6 |
| 39S ribosomal protein L40, mitochondrial OS=Homo sapiens OX=9606 GN=MRPL40 PE=1 SV=1 | 1219908 |
| Putative RNA-binding protein Luc7-like 1 OS=Homo sapiens OX=9606 GN=LUC7L PE=1 SV=1 | 62048530 |
| NLR family CARD domain-containing protein 4 OS=Homo sapiens OX=9606 GN=NLRC4 PE=1 SV=2 | 14910376 |
| DNA methyltransferase 1-associated protein 1 OS=Homo sapiens OX=9606 GN=DMAP1 PE=1 SV=1 | 1375813 |
| Exosome complex component RRP41 OS=Homo sapiens OX=9606 GN=EXOSC4 PE=1 SV=3 | 8859479 |
| 39S ribosomal protein L47, mitochondrial OS=Homo sapiens OX=9606 GN=MRPL47 PE=1 SV=2 | 2708741 |
| Band 4.1-like protein 5 OS=Homo sapiens OX=9606 GN=EPB41L5 PE=1 SV=3 | 6009132 |
| Chromodomain-helicase-DNA-binding protein 8 OS=Homo sapiens OX=9606 GN=CHD8 PE=1 SV=5 | 4246453 |
| N6-adenosine-methyltransferase non-catalytic subunit OS=Homo sapiens OX=9606 GN=METTL14 PE=1 SV=2 | 1713131 |
| Transcription initiation factor TFIID subunit 9B OS=Homo sapiens OX=9606 GN=TAF9B PE=1 SV=1 | 1555565 |
| Calcyclin-binding protein OS=Homo sapiens OX=9606 GN=CACYBP PE=1 SV=2 | 520978.4 |
| Desmocollin 1, isoform CRA_b OS=Homo sapiens OX=9606 GN=DSC1 PE=4 SV=1 | 2344673 |
| Regulator of nonsense transcripts 2 OS=Homo sapiens OX=9606 GN=UPF2 PE=1 SV=1 | 21289337 |
| Pleckstrin homology domain-containing family A member 5 OS=Homo sapiens OX=9606 GN=PLEKHA5 PE=1 SV=1 | 1267966 |
| Nicotinamide/nicotinic acid mononucleotide adenylyltransferase 1 OS=Homo sapiens OX=9606 GN=NMNAT1 PE=1 SV=1 | 3467757 |
| Ribosome production factor 1 OS=Homo sapiens OX=9606 GN=RPF1 PE=1 SV=2 | 823814.3 |
| WD repeat-containing protein 76 OS=Homo sapiens OX=9606 GN=WDR76 PE=1 SV=2 | 608734.9 |
| Protein ECT2 OS=Homo sapiens OX=9606 GN=ECT2 PE=1 SV=4 | 3532285 |
| Caspase activity and apoptosis inhibitor 1 OS=Homo sapiens OX=9606 GN=CAAP1 PE=1 SV=2 | 881659.7 |
| PRKR-interacting protein 1 OS=Homo sapiens OX=9606 GN=PRKRIP1 PE=1 SV=1 | 1979688 |
| Tudor domain-containing protein 3 OS=Homo sapiens OX=9606 GN=TDRD3 PE=1 SV=1 | 15466942 |
| 3'-5' RNA helicase YTHDC2 OS=Homo sapiens OX=9606 GN=YTHDC2 PE=1 SV=2 | 3587599 |
| Receptor expression-enhancing protein 4 OS=Homo sapiens OX=9606 GN=REEP4 PE=1 SV=1 | 4292480 |
| Coiled-coil domain-containing protein 86 OS=Homo sapiens OX=9606 GN=CCDC86 PE=1 SV=1 | 15116576 |
| STING ER exit protein OS=Homo sapiens OX=9606 GN=STEEP1 PE=1 SV=1 | 5202481 |
| ESF1 homolog OS=Homo sapiens OX=9606 GN=ESF1 PE=1 SV=1 | 13536153 |
| 39S ribosomal protein L46, mitochondrial OS=Homo sapiens OX=9606 GN=MRPL46 PE=1 SV=1 | 13097187 |
| Activity-dependent neuroprotector homeobox protein OS=Homo sapiens OX=9606 GN=ADNP PE=1 SV=1 | 1477501 |
| Anaphase-promoting complex subunit 1 OS=Homo sapiens OX=9606 GN=ANAPC1 PE=1 SV=1 | 870798.8 |
| Nucleotide exchange factor SIL1 OS=Homo sapiens OX=9606 GN=SIL1 PE=1 SV=1 | 19856582 |
| Magnesium transporter protein 1 OS=Homo sapiens OX=9606 GN=MAGT1 PE=1 SV=1 | 7331179 |
| Probable ATP-dependent RNA helicase DDX47 OS=Homo sapiens OX=9606 GN=DDX47 PE=1 SV=1 | 32003858 |
| Pterin-4-alpha-carbinolamine dehydratase 2 OS=Homo sapiens OX=9606 GN=PCBD2 PE=1 SV=4 | 4759219 |
| Rac GTPase-activating protein 1 OS=Homo sapiens OX=9606 GN=RACGAP1 PE=1 SV=1 | 3413027 |
| RNA cytidine acetyltransferase OS=Homo sapiens OX=9606 GN=NAT10 PE=1 SV=2 | 39742680 |
| Large subunit GTPase 1 homolog OS=Homo sapiens OX=9606 GN=LSG1 PE=1 SV=2 | 45490057 |
| Probable E3 ubiquitin-protein ligase makorin-2 OS=Homo sapiens OX=9606 GN=MKRN2 PE=1 SV=2 | 611679.1 |
| WD repeat-containing protein 61 OS=Homo sapiens OX=9606 GN=WDR61 PE=1 SV=1 | 742653.8 |
| RNA exonuclease 4 OS=Homo sapiens OX=9606 GN=REXO4 PE=1 SV=2 | 463964.3 |
| pre-mRNA 3' end processing protein WDR33 OS=Homo sapiens OX=9606 GN=WDR33 PE=1 SV=2 | 69109430 |
| Regulator of nonsense transcripts 3B OS=Homo sapiens OX=9606 GN=UPF3B PE=1 SV=1 | 55261443 |
| GTP-binding protein 4 OS=Homo sapiens OX=9606 GN=GTPBP4 PE=1 SV=3 | 1.09E+08 |
| 28S ribosomal protein S26, mitochondrial OS=Homo sapiens OX=9606 GN=MRPS26 PE=1 SV=1 | 1740843 |
| MKI67 FHA domain-interacting nucleolar phosphoprotein OS=Homo sapiens OX=9606 GN=NIFK PE=1 SV=1 | 3497049 |
| Polymerase delta-interacting protein 3 OS=Homo sapiens OX=9606 GN=POLDIP3 PE=1 SV=2 | 2.06E+08 |
| Nucleolar and spindle-associated protein 1 OS=Homo sapiens OX=9606 GN=NUSAP1 PE=1 SV=1 | 9286938 |
| Serrate RNA effector molecule homolog OS=Homo sapiens OX=9606 GN=SRRT PE=1 SV=1 | 42767340 |
| Krueppel-like factor 16 OS=Homo sapiens OX=9606 GN=KLF16 PE=1 SV=1 | 2779278 |
| Protein LSM14 homolog B OS=Homo sapiens OX=9606 GN=LSM14B PE=1 SV=1 | 63453882 |
| GTP-binding protein 2 OS=Homo sapiens OX=9606 GN=GTPBP2 PE=1 SV=1 | 5093158 |
| Serine/threonine-protein kinase RIO2 OS=Homo sapiens OX=9606 GN=RIOK2 PE=1 SV=2 | 11962022 |
| Spermatogenesis-associated protein 5-like protein 1 OS=Homo sapiens OX=9606 GN=SPATA5L1 PE=1 SV=2 | 306810.8 |
| Guanine nucleotide-binding protein-like 3 OS=Homo sapiens OX=9606 GN=GNL3 PE=1 SV=2 | 84186668 |
| U3 small nucleolar RNA-associated protein 14 homolog A OS=Homo sapiens OX=9606 GN=UTP14A PE=1 SV=1 | 11962680 |
| Nucleolar complex protein 4 homolog OS=Homo sapiens OX=9606 GN=NOC4L PE=1 SV=1 | 21337535 |
| PWP1 homolog (S. cerevisiae) OS=Homo sapiens OX=9606 GN=PWP1 PE=2 SV=1 | 6650109 |
| TNF receptor-associated factor 4 OS=Homo sapiens OX=9606 GN=TRAF4 PE=1 SV=1 | 211149 |
| Probable ATP-dependent RNA helicase DDX23 OS=Homo sapiens OX=9606 GN=DDX23 PE=1 SV=3 | 23632894 |
| Multiple myeloma tumor-associated protein 2 OS=Homo sapiens OX=9606 GN=MMTAG2 PE=1 SV=1 | 20408490 |
| DCN1-like protein 5 OS=Homo sapiens OX=9606 GN=DCUN1D5 PE=1 SV=1 | 17267846 |
| Mini-chromosome maintenance complex-binding protein OS=Homo sapiens OX=9606 GN=MCMBP PE=1 SV=2 | 1285225 |
| RNA-binding protein 42 OS=Homo sapiens OX=9606 GN=RBM42 PE=1 SV=1 | 1852260 |
| Death-inducer obliterator 1 OS=Homo sapiens OX=9606 GN=DIDO1 PE=1 SV=5 | 10868476 |
| INT1 protein (Fragment) OS=Homo sapiens OX=9606 GN=INT1 PE=2 SV=1 | 837617.9 |
| Rhotekin OS=Homo sapiens OX=9606 GN=RTKN PE=1 SV=2 | 1038029 |
| Serine/threonine-protein kinase RIO1 OS=Homo sapiens OX=9606 GN=RIOK1 PE=1 SV=2 | 98584480 |
| Uncharacterized protein C7orf50 OS=Homo sapiens OX=9606 GN=C7orf50 PE=1 SV=1 | 5649618 |
| BUD13 homolog OS=Homo sapiens OX=9606 GN=BUD13 PE=1 SV=1 | 462535.1 |
| Ribosomal protein L27a OS=Homo sapiens OX=9606 GN=L27a PE=4 SV=1 | 7.05E+08 |
| Ribosomal protein 63, mitochondrial OS=Homo sapiens OX=9606 GN=MRPL57 PE=1 SV=1 | 1057385 |
| Methylosome protein 50 OS=Homo sapiens OX=9606 GN=WDR77 PE=1 SV=1 | 59036300 |
| Transcription factor 25 OS=Homo sapiens OX=9606 GN=TCF25 PE=1 SV=1 | 4726007 |
| Glutamate-rich WD repeat-containing protein 1 OS=Homo sapiens OX=9606 GN=GRWD1 PE=1 SV=1 | 28935436 |
| Endophilin-A2 OS=Homo sapiens OX=9606 GN=SH3GL1 PE=1 SV=1 | 507292.1 |
| Plakophilin-2 OS=Homo sapiens OX=9606 GN=PKP2 PE=1 SV=2 | 12026595 |
| BAG family molecular chaperone regulator 1 OS=Homo sapiens OX=9606 GN=BAG1 PE=1 SV=4 | 2455035 |
| Probable rRNA-processing protein EBP2 OS=Homo sapiens OX=9606 GN=EBNA1BP2 PE=1 SV=2 | 3351698 |
| T-complex protein 1 subunit eta OS=Homo sapiens OX=9606 GN=CCT7 PE=1 SV=2 | 3289732 |
| Cell division control protein 6 homolog OS=Homo sapiens OX=9606 GN=CDC6 PE=1 SV=1 | 721317.9 |
| 3-hydroxyacyl-CoA dehydrogenase type-2 OS=Homo sapiens OX=9606 GN=HSD17B10 PE=1 SV=3 | 1043954 |
| Protein SCAF11 OS=Homo sapiens OX=9606 GN=SCAF11 PE=1 SV=2 | 2604003 |
| DnaJ homolog subfamily C member 2 OS=Homo sapiens OX=9606 GN=DNAJC2 PE=1 SV=4 | 3792440 |
| TOB3 OS=Homo sapiens OX=9606 PE=2 SV=1 | 4950231 |
| Msx2-interacting protein OS=Homo sapiens OX=9606 GN=SPEN PE=1 SV=1 |  |
| Flt3-interacting zinc finger protein 1 OS=Homo sapiens OX=9606 GN=FIZ1 PE=1 SV=2 | 874516.2 |
| Cyclin-L2 OS=Homo sapiens OX=9606 GN=CCNL2 PE=1 SV=1 |  |
| Ran-binding protein 9 OS=Homo sapiens OX=9606 GN=RANBP9 PE=1 SV=1 | 727628.4 |
| Transcriptional activator protein Pur-beta OS=Homo sapiens OX=9606 GN=PURB PE=1 SV=3 | 1202014 |
| Serine/threonine-protein phosphatase 1 regulatory subunit 10 OS=Homo sapiens OX=9606 GN=PPP1R10 PE=1 SV=1 | 30478081 |
| Leukocyte receptor cluster member 8 OS=Homo sapiens OX=9606 GN=LENG8 PE=1 SV=3 | 66782960 |
| RNA-binding protein 14 OS=Homo sapiens OX=9606 GN=RBM14 PE=1 SV=2 | 1.41E+08 |
| Serpin B12 OS=Homo sapiens OX=9606 GN=SERPINB12 PE=1 SV=1 | 1688127 |
| Regulation of nuclear pre-mRNA domain-containing protein 1A OS=Homo sapiens OX=9606 GN=RPRD1A PE=1 SV=1 | 255216.5 |
| Kinesin-like protein KIF16B OS=Homo sapiens OX=9606 GN=KIF16B PE=1 SV=2 | 12259593 |
| Beta-1,3-galactosyltransferase 6 OS=Homo sapiens OX=9606 GN=B3GALT6 PE=1 SV=2 | 1658051 |
| N-terminal kinase-like protein OS=Homo sapiens OX=9606 GN=SCYL1 PE=1 SV=1 | 931161.4 |
| Hypermethylated in cancer 2 protein OS=Homo sapiens OX=9606 GN=HIC2 PE=1 SV=2 | 4726521 |
| ATP synthase membrane subunit K, mitochondrial OS=Homo sapiens OX=9606 GN=ATP5MK PE=1 SV=1 | 630573.1 |
| RPS4X protein (Fragment) OS=Homo sapiens OX=9606 GN=RPS4X PE=2 SV=2 | 2.79E+09 |
| RCC1-like G exchanging factor-like protein OS=Homo sapiens OX=9606 GN=RCC1L PE=1 SV=2 | 1365199 |
| Splicing factor 45 OS=Homo sapiens OX=9606 GN=RBM17 PE=1 SV=1 | 9761482 |
| Far upstream element-binding protein 3 OS=Homo sapiens OX=9606 GN=FUBP3 PE=1 SV=2 | 4422320 |
| DDRGK domain-containing protein 1 OS=Homo sapiens OX=9606 GN=DDRGK1 PE=1 SV=2 | 4081701 |
| Serine/threonine-protein phosphatase PGAM5, mitochondrial OS=Homo sapiens OX=9606 GN=PGAM5 PE=1 SV=2 | 91554541 |
| Zinc finger C2HC domain-containing protein 1A OS=Homo sapiens OX=9606 GN=ZC2HC1A PE=1 SV=2 | 6772164 |
| Probable ATP-dependent RNA helicase DDX27 OS=Homo sapiens OX=9606 GN=DDX27 PE=1 SV=2 | 2140775 |
| Target of EGR1 protein 1 OS=Homo sapiens OX=9606 GN=TOE1 PE=1 SV=1 | 3099597 |
| 39S ribosomal protein L48, mitochondrial OS=Homo sapiens OX=9606 GN=MRPL48 PE=1 SV=2 | 12753057 |
| Protein LTV1 homolog OS=Homo sapiens OX=9606 GN=LTV1 PE=1 SV=1 | 17340913 |
| THO complex subunit 1 OS=Homo sapiens OX=9606 GN=THOC1 PE=1 SV=1 | 2198371 |
| Processing of 1, ribonuclease P/MRP subunit (S. cerevisiae) OS=Homo sapiens OX=9606 GN=POP1 PE=2 SV=1 | 4038359 |
| Pentatricopeptide repeat domain-containing protein 3, mitochondrial OS=Homo sapiens OX=9606 GN=PTCD3 PE=1 SV=3 | 1117038 |
| DnaJ homolog subfamily A member 3, mitochondrial OS=Homo sapiens OX=9606 GN=DNAJA3 PE=1 SV=2 | 3094095 |
| Cytoplasmic dynein 2 intermediate chain 2 OS=Homo sapiens OX=9606 GN=DYNC2I2 PE=1 SV=2 | 2212202 |
| Ribosomal RNA processing protein 36 homolog OS=Homo sapiens OX=9606 GN=RRP36 PE=1 SV=1 |  |
| SAGA-associated factor 29 OS=Homo sapiens OX=9606 GN=SGF29 PE=1 SV=1 | 1144522 |
| THAP domain-containing protein 11 OS=Homo sapiens OX=9606 GN=THAP11 PE=1 SV=2 | 1631133 |
| 39S ribosomal protein L38, mitochondrial OS=Homo sapiens OX=9606 GN=MRPL38 PE=1 SV=2 | 676909.3 |
| Zinc finger and BTB domain-containing protein 10 OS=Homo sapiens OX=9606 GN=ZBTB10 PE=1 SV=2 | 15107758 |
| U5 small nuclear ribonucleoprotein 40 kDa protein OS=Homo sapiens OX=9606 GN=SNRNP40 PE=1 SV=1 | 5219669 |
| R3H domain-containing protein 4 OS=Homo sapiens OX=9606 GN=R3HDM4 PE=1 SV=3 | 4088103 |
| Coiled-coil domain-containing protein 124 OS=Homo sapiens OX=9606 GN=CCDC124 PE=1 SV=1 | 7645160 |
| Ribosome biogenesis protein NOP53 (Fragment) OS=Homo sapiens OX=9606 GN=GLTSCR2 PE=2 SV=2 | 14013433 |
| FBL protein (Fragment) OS=Homo sapiens OX=9606 GN=FBL PE=2 SV=2 | 1.16E+08 |
| Transcriptional adapter 1 OS=Homo sapiens OX=9606 GN=TADA1 PE=1 SV=1 | 2157457 |
| Crossover junction endonuclease EME1 OS=Homo sapiens OX=9606 GN=EME1 PE=1 SV=2 | 2618319 |
| Leucine-rich repeat-containing protein 59 OS=Homo sapiens OX=9606 GN=LRRC59 PE=1 SV=1 | 1.36E+08 |
| PAT complex subunit CCDC47 OS=Homo sapiens OX=9606 GN=CCDC47 PE=1 SV=1 | 6973378 |
| Trace amine-associated receptor 8 OS=Homo sapiens OX=9606 GN=TAAR8 PE=2 SV=1 | 3240324 |
| U7 snRNA-associated Sm-like protein LSm10 OS=Homo sapiens OX=9606 GN=LSM10 PE=1 SV=1 | 2923751 |
| Ubiquitin carboxyl-terminal hydrolase 7 OS=Homo sapiens OX=9606 GN=USP7 PE=1 SV=2 | 6946836 |
| Polyribonucleotide 5'-hydroxyl-kinase Clp1 OS=Homo sapiens OX=9606 GN=CLP1 PE=1 SV=1 | 4088500 |
| Ribosomal RNA small subunit methyltransferase NEP1 OS=Homo sapiens OX=9606 GN=EMG1 PE=1 SV=4 | 3986607 |
| snRNA-activating protein complex subunit 3 OS=Homo sapiens OX=9606 GN=SNAPC3 PE=1 SV=1 | 191351.9 |
| Far upstream element-binding protein 2 OS=Homo sapiens OX=9606 GN=KHSRP PE=1 SV=4 | 1560422 |
| DNA repair protein RAD50 OS=Homo sapiens OX=9606 GN=RAD50 PE=1 SV=1 | 18116793 |
| TATA-binding protein-associated factor 2N OS=Homo sapiens OX=9606 GN=TAF15 PE=1 SV=1 | 1.79E+08 |
| Symplekin OS=Homo sapiens OX=9606 GN=SYMPK PE=1 SV=2 | 46848906 |
| General transcription factor IIH subunit 4 OS=Homo sapiens OX=9606 GN=GTF2H4 PE=1 SV=1 | 1730943 |
| Receptor-type tyrosine-protein phosphatase U OS=Homo sapiens OX=9606 GN=PTPRU PE=1 SV=2 | 4179929 |
| La-related protein 4B OS=Homo sapiens OX=9606 GN=LARP4B PE=1 SV=3 | 27285538 |
| Histone H1.10 OS=Homo sapiens OX=9606 GN=H1-10 PE=1 SV=1 | 53926236 |
| DnaJ homolog subfamily C member 9 OS=Homo sapiens OX=9606 GN=DNAJC9 PE=1 SV=1 | 78034997 |
| Transcriptional repressor p66-beta OS=Homo sapiens OX=9606 GN=GATAD2B PE=1 SV=1 | 12272421 |
| Stonin-2 OS=Homo sapiens OX=9606 GN=STON2 PE=1 SV=1 | 3304348 |
| U4/U6 small nuclear ribonucleoprotein Prp31 OS=Homo sapiens OX=9606 GN=PRPF31 PE=1 SV=2 | 15125381 |
| Ataxin-2-like protein OS=Homo sapiens OX=9606 GN=ATXN2L PE=1 SV=2 | 97607571 |
| ZNF277 protein OS=Homo sapiens OX=9606 GN=ZNF277 PE=1 SV=1 | 11656172 |
| Heterogeneous nuclear ribonucleoprotein L-like OS=Homo sapiens OX=9606 GN=HNRNPLL PE=1 SV=1 | 3718502 |
| Protein POF1B OS=Homo sapiens OX=9606 GN=POF1B PE=1 SV=3 |  |
| Cytoplasmic dynein 2 intermediate chain 1 OS=Homo sapiens OX=9606 GN=DYNC2I1 PE=1 SV=3 |  |
| Dimethyladenosine transferase 1, mitochondrial OS=Homo sapiens OX=9606 GN=TFB1M PE=1 SV=1 | 6415722 |
| U4/U6.U5 small nuclear ribonucleoprotein 27 kDa protein OS=Homo sapiens OX=9606 GN=SNRNP27 PE=1 SV=1 | 795335.4 |
| Small integral membrane protein 4 OS=Homo sapiens OX=9606 GN=SMIM4 PE=1 SV=2 | 1276684 |
| GATA zinc finger domain-containing protein 1 OS=Homo sapiens OX=9606 GN=GATAD1 PE=1 SV=1 | 509897.6 |
| POLDIP3 protein OS=Homo sapiens OX=9606 GN=POLDIP3 PE=1 SV=1 |  |
| Cactin OS=Homo sapiens OX=9606 GN=CACTIN PE=1 SV=3 | 4779761 |
| Gem (Nuclear organelle) associated protein 4 OS=Homo sapiens OX=9606 GN=GEMIN4 PE=2 SV=1 | 5352300 |
| Phosphatidylglycerophosphatase and protein-tyrosine phosphatase 1 OS=Homo sapiens OX=9606 GN=PTPMT1 PE=1 SV=1 | 973564.1 |
| PHD finger protein 10 OS=Homo sapiens OX=9606 GN=PHF10 PE=1 SV=3 | 4201013 |
| General transcription factor 3C polypeptide 2 OS=Homo sapiens OX=9606 GN=GTF3C2 PE=1 SV=2 | 2520151 |
| Peptidyl-prolyl cis-trans isomerase-like 4 OS=Homo sapiens OX=9606 GN=PPIL4 PE=1 SV=1 | 4186530 |
| Zinc finger CCCH domain-containing protein 15 OS=Homo sapiens OX=9606 GN=ZC3H15 PE=1 SV=1 | 49229379 |
| Nucleolar complex protein 3 homolog OS=Homo sapiens OX=9606 GN=NOC3L PE=1 SV=1 | 645776.9 |
| ATP-dependent RNA helicase DDX54 OS=Homo sapiens OX=9606 GN=DDX54 PE=1 SV=2 | 52310876 |
| MLL/AF4 fusion protein (Fragment) OS=Homo sapiens OX=9606 PE=2 SV=1 | 286122.4 |
| Dolichyl-diphosphooligosaccharide--protein glycosyltransferase subunit STT3B OS=Homo sapiens OX=9606 GN=STT3B PE=1 SV=1 | 1659331 |
| Uncharacterized protein (Fragment) OS=Homo sapiens OX=9606 PE=2 SV=1 | 1382981 |
| Fusion (Involved in t(1216) in malignant liposarcoma) OS=Homo sapiens OX=9606 GN=FUS PE=2 SV=1 | 2.06E+09 |
| Pumilio homolog 2 OS=Homo sapiens OX=9606 GN=PUM2 PE=1 SV=2 | 261150.6 |
| Similar to cytoskeleton-associated protein 4 (Fragment) OS=Homo sapiens OX=9606 PE=2 SV=1 | 66006509 |
| ATP synthase subunit gamma (Fragment) OS=Homo sapiens OX=9606 PE=2 SV=1 | 4377812 |
| M-phase-specific PLK1-interacting protein OS=Homo sapiens OX=9606 GN=MPLKIP PE=1 SV=1 | 2972020 |
| Smad nuclear-interacting protein 1 OS=Homo sapiens OX=9606 GN=SNIP1 PE=1 SV=1 | 9969431 |
| Ribosomal protein S2 OS=Homo sapiens OX=9606 GN=OK/KNS-cl.6 PE=2 SV=1 | 4154114 |
| THO complex subunit 2 OS=Homo sapiens OX=9606 GN=THOC2 PE=1 SV=2 | 3139101 |
| Olfactory receptor 6N2 OS=Homo sapiens OX=9606 GN=OR6N2 PE=3 SV=1 | 1315142 |
| N-acylneuraminate cytidylyltransferase OS=Homo sapiens OX=9606 GN=CMAS PE=1 SV=2 | 75839795 |
| Cytosolic endo-beta-N-acetylglucosaminidase OS=Homo sapiens OX=9606 GN=ENGASE PE=1 SV=1 |  |
| Neuroguidin OS=Homo sapiens OX=9606 GN=NGDN PE=1 SV=1 | 5071099 |
| MTMR1 protein OS=Homo sapiens OX=9606 GN=MTMR1 PE=1 SV=1 | 944149.8 |
| Divergent protein kinase domain 2A OS=Homo sapiens OX=9606 GN=DIPK2A PE=1 SV=1 | 2326439 |
| Putative RNA-binding protein 15B OS=Homo sapiens OX=9606 GN=RBM15B PE=1 SV=3 | 1821101 |
| Protein LSM14 homolog A OS=Homo sapiens OX=9606 GN=LSM14A PE=1 SV=3 | 94118341 |
| NFATC2-interacting protein OS=Homo sapiens OX=9606 GN=NFATC2IP PE=1 SV=1 | 17339869 |
| Plasminogen activator inhibitor 1 RNA-binding protein OS=Homo sapiens OX=9606 GN=SERBP1 PE=1 SV=2 | 2.87E+09 |
| tRNA-uridine aminocarboxypropyltransferase 2 OS=Homo sapiens OX=9606 GN=DTWD2 PE=1 SV=1 | 1276455 |
| Pre-mRNA-splicing factor 38A OS=Homo sapiens OX=9606 GN=PRPF38A PE=1 SV=1 | 4253491 |
| Activating signal cointegrator 1 complex subunit 1 OS=Homo sapiens OX=9606 GN=ASCC1 PE=1 SV=1 | 2134609 |
| Uncharacterized protein C19orf47 OS=Homo sapiens OX=9606 GN=C19orf47 PE=1 SV=1 | 23521686 |
| ATP-citrate synthase OS=Homo sapiens OX=9606 PE=2 SV=1 | 277873.4 |
| RNA polymerase II-associated factor 1 homolog OS=Homo sapiens OX=9606 GN=PAF1 PE=1 SV=2 | 807294.3 |
| EF-hand calcium-binding domain-containing protein 3 OS=Homo sapiens OX=9606 GN=EFCAB3 PE=1 SV=1 | 2860376 |
| Tubulin beta chain OS=Homo sapiens OX=9606 GN=TUBB2C PE=2 SV=1 | 22591702 |
| Cleavage and polyadenylation specificity factor subunit 7 OS=Homo sapiens OX=9606 GN=CPSF7 PE=1 SV=1 | 31675826 |
| Ribonuclease P protein subunit p25-like protein OS=Homo sapiens OX=9606 GN=RPP25L PE=1 SV=1 | 1563006 |
| S1 RNA-binding domain-containing protein 1 OS=Homo sapiens OX=9606 GN=SRBD1 PE=1 SV=2 | 2300638 |
| Zinc finger CCCH-type with G patch domain-containing protein OS=Homo sapiens OX=9606 GN=ZGPAT PE=1 SV=3 | 3123092 |
| Zinc finger CCHC domain-containing protein 9 OS=Homo sapiens OX=9606 GN=ZCCHC9 PE=1 SV=2 | 1138096 |
| Tuftelin-interacting protein 11 OS=Homo sapiens OX=9606 GN=TFIP11 PE=2 SV=1 | 536315.9 |
| Uncharacterized protein DKFZp451A052 OS=Homo sapiens OX=9606 GN=DKFZp451A052 PE=2 SV=2 | 1337881 |
| 1-phosphatidylinositol 4,5-bisphosphate phosphodiesterase delta-3 OS=Homo sapiens OX=9606 GN=PLCD3 PE=1 SV=3 | 827791.3 |
| Activating signal cointegrator 1 complex subunit 3 OS=Homo sapiens OX=9606 GN=ASCC3 PE=1 SV=3 | 4051044 |
| Histone H2B type 3-B OS=Homo sapiens OX=9606 GN=H2BU1 PE=1 SV=3 | 13378870 |
| Keratin, type II cytoskeletal 78 OS=Homo sapiens OX=9606 GN=KRT78 PE=1 SV=2 | 8220453 |
| Putative inactive cytochrome P450 family member 4Z2 OS=Homo sapiens OX=9606 GN=CYP4Z2P PE=5 SV=2 | 43256776 |
| Leucine-rich repeat-containing protein 47 OS=Homo sapiens OX=9606 GN=LRRC47 PE=1 SV=1 | 5696152 |
| 75 kDa glucose-regulated protein (Fragment) OS=Homo sapiens OX=9606 GN=HSPA9 PE=2 SV=1 | 19905686 |
| Putative ribosome-binding factor A, mitochondrial OS=Homo sapiens OX=9606 GN=RBFA PE=1 SV=3 | 9337032 |
| Selenoprotein H OS=Homo sapiens OX=9606 GN=SELENOH PE=1 SV=2 | 22594455 |
| Mucolipin-2 OS=Homo sapiens OX=9606 GN=MCOLN2 PE=1 SV=2 | 489836.8 |
| 5'-3' exoribonuclease 1 OS=Homo sapiens OX=9606 GN=XRN1 PE=1 SV=1 | 3179265 |
| tRNA (uracil-5-)-methyltransferase homolog A OS=Homo sapiens OX=9606 GN=TRMT2A PE=1 SV=2 | 3016603 |
| pre-rRNA 2'-O-ribose RNA methyltransferase FTSJ3 OS=Homo sapiens OX=9606 GN=FTSJ3 PE=1 SV=2 | 61991067 |
| Probable ATP-dependent RNA helicase DHX37 OS=Homo sapiens OX=9606 GN=DHX37 PE=1 SV=1 | 25625015 |
| Solute carrier family 15 member 3 OS=Homo sapiens OX=9606 GN=SLC15A3 PE=1 SV=2 | 217389 |
| 39S ribosomal protein L41, mitochondrial OS=Homo sapiens OX=9606 GN=MRPL41 PE=1 SV=1 | 1212482 |
| Calcium homeostasis endoplasmic reticulum protein OS=Homo sapiens OX=9606 GN=CHERP PE=1 SV=3 | 19663863 |
| Zinc finger CCCH domain-containing protein 7A OS=Homo sapiens OX=9606 GN=ZC3H7A PE=1 SV=1 | 688042.1 |
| MAP7 domain-containing protein 3 OS=Homo sapiens OX=9606 GN=MAP7D3 PE=1 SV=2 | 746067.3 |
| Hornerin OS=Homo sapiens OX=9606 GN=HRNR PE=1 SV=2 |  |
| Copine-8 OS=Homo sapiens OX=9606 GN=CPNE8 PE=1 SV=2 | 641672 |
| Transcriptional repressor p66-alpha OS=Homo sapiens OX=9606 GN=GATAD2A PE=1 SV=1 | 5713547 |
| Spermatogenesis-associated serine-rich protein 2 OS=Homo sapiens OX=9606 GN=SPATS2 PE=1 SV=1 | 427639.6 |
| Active regulator of SIRT1 OS=Homo sapiens OX=9606 GN=RPS19BP1 PE=1 SV=1 | 777138.4 |
| Liprin-beta-1 OS=Homo sapiens OX=9606 GN=PPFIBP1 PE=1 SV=2 | 2812454 |
| THO complex subunit 6 homolog OS=Homo sapiens OX=9606 GN=THOC6 PE=1 SV=1 | 490354.2 |
| RNA helicase (Fragment) OS=Homo sapiens OX=9606 GN=DDX10 PE=2 SV=1 | 4174248 |
| Leucine zipper protein 1 OS=Homo sapiens OX=9606 GN=LUZP1 PE=1 SV=2 | 3043430 |
| N-alpha-acetyltransferase 40 OS=Homo sapiens OX=9606 GN=NAA40 PE=1 SV=1 | 660259.1 |
| Telomerase-binding protein EST1A OS=Homo sapiens OX=9606 GN=SMG6 PE=1 SV=2 | 16434195 |
| E3 ubiquitin-protein ligase ZNF598 OS=Homo sapiens OX=9606 GN=ZNF598 PE=1 SV=1 | 34433503 |
| Serine/threonine-protein kinase tousled-like 2 OS=Homo sapiens OX=9606 GN=TLK2 PE=1 SV=2 | 1754498 |
| Protein LYRIC OS=Homo sapiens OX=9606 GN=MTDH PE=1 SV=2 | 7115392 |
| N6-adenosine-methyltransferase catalytic subunit OS=Homo sapiens OX=9606 GN=METTL3 PE=1 SV=2 | 11267251 |
| Polyadenylate-binding protein 2 OS=Homo sapiens OX=9606 GN=PABPN1 PE=1 SV=3 | 8784095 |
| Tubulin epsilon and delta complex protein 1 OS=Homo sapiens OX=9606 GN=TEDC1 PE=2 SV=2 | 626915.1 |
| 39S ribosomal protein L55, mitochondrial OS=Homo sapiens OX=9606 GN=MRPL55 PE=1 SV=1 | 7485527 |
| Keratin, type II cytoskeletal 1b OS=Homo sapiens OX=9606 GN=KRT77 PE=1 SV=3 | 2095846 |
| Uncharacterized protein DKFZp779I2251 (Fragment) OS=Homo sapiens OX=9606 GN=DKFZp779I2251 PE=2 SV=1 | 992390.4 |
| Vomeronasal type-1 receptor 5 OS=Homo sapiens OX=9606 GN=VN1R5 PE=2 SV=2 | 241890 |
| HEAT repeat-containing protein 3 OS=Homo sapiens OX=9606 GN=HEATR3 PE=1 SV=2 | 2759578 |
| CLIP-associating protein 1 OS=Homo sapiens OX=9606 GN=CLASP1 PE=1 SV=1 | 1868897 |
| Nuclear fragile X mental retardation-interacting protein 2 OS=Homo sapiens OX=9606 GN=NUFIP2 PE=1 SV=1 | 61015123 |
| Zinc finger CCCH-type antiviral protein 1 OS=Homo sapiens OX=9606 GN=ZC3HAV1 PE=1 SV=3 | 7414786 |
| Heparan sulfate 2-O-sulfotransferase 1 OS=Homo sapiens OX=9606 GN=HS2ST1 PE=1 SV=1 | 1877779 |
| Rab11 family-interacting protein 2 OS=Homo sapiens OX=9606 GN=RAB11FIP2 PE=1 SV=1 | 859088 |
| Protein MCM10 homolog OS=Homo sapiens OX=9606 GN=MCM10 PE=1 SV=2 | 1357924 |
| 7SK snRNA methylphosphate capping enzyme OS=Homo sapiens OX=9606 GN=MEPCE PE=1 SV=1 | 9857471 |
| Eukaryotic translation initiation factor 3 subunit M OS=Homo sapiens OX=9606 GN=EIF3M PE=1 SV=1 | 13907899 |
| tRNA methyltransferase 10 homolog C OS=Homo sapiens OX=9606 GN=TRMT10C PE=1 SV=2 | 13271478 |
| E3 ubiquitin-protein ligase Hakai OS=Homo sapiens OX=9606 GN=CBLL1 PE=1 SV=1 | 10863917 |
| Uncharacterized protein IFRD1 (Fragment) OS=Homo sapiens OX=9606 GN=IFRD1 PE=4 SV=1 | 2880314 |
| 40S ribosomal protein S26 OS=Homo sapiens OX=9606 GN=RPS26 PE=3 SV=1 | 3.33E+08 |
| Uncharacterized protein WBSCR22 OS=Homo sapiens OX=9606 GN=WBSCR22 PE=3 SV=1 | 45741074 |
| Peptidyl-prolyl cis-trans isomerase OS=Homo sapiens OX=9606 PE=2 SV=1 | 4141918 |
| 40S ribosomal protein S27-like OS=Homo sapiens OX=9606 GN=RPS27L PE=1 SV=3 | 1029817 |
| Histone H2A.V OS=Homo sapiens OX=9606 GN=H2AZ2 PE=1 SV=3 | 32565680 |
| Dyad symmetry binding protein (Fragment) OS=Homo sapiens OX=9606 PE=2 SV=1 | 21729878 |
| La-related protein 4 OS=Homo sapiens OX=9606 GN=LARP4 PE=1 SV=3 | 18407226 |
| Inhibitor of nuclear factor kappa-B kinase-interacting protein OS=Homo sapiens OX=9606 GN=IKBIP PE=1 SV=1 | 5387632 |
| LHFPL tetraspan subfamily member 2 protein OS=Homo sapiens OX=9606 GN=LHFPL2 PE=1 SV=2 | 1181449 |
| Uncharacterized protein C1orf122 OS=Homo sapiens OX=9606 GN=C1orf122 PE=4 SV=2 | 1088392 |
| cDNA FLJ27420 fis, clone WMC07143 OS=Homo sapiens OX=9606 PE=2 SV=1 | 1784100 |
| WD repeat-containing protein 82 OS=Homo sapiens OX=9606 GN=WDR82 PE=1 SV=1 | 87579048 |
| Pre-mRNA 3'-end-processing factor FIP1 OS=Homo sapiens OX=9606 GN=FIP1L1 PE=1 SV=1 | 55772930 |
| La-related protein 1 OS=Homo sapiens OX=9606 GN=LARP1 PE=1 SV=2 | 1.02E+08 |
| Kinesin-like protein (Fragment) OS=Homo sapiens OX=9606 GN=KIF5C PE=2 SV=1 |  |
| Zinc finger CCCH domain-containing protein 14 OS=Homo sapiens OX=9606 GN=ZC3H14 PE=1 SV=1 | 6073313 |
| RRP1 protein (Fragment) OS=Homo sapiens OX=9606 GN=RRP1 PE=2 SV=2 | 51842128 |
| F-box/LRR-repeat protein 19 OS=Homo sapiens OX=9606 GN=FBXL19 PE=1 SV=3 | 319062.3 |
| Lysine-specific demethylase RSBN1L OS=Homo sapiens OX=9606 GN=RSBN1L PE=1 SV=2 | 7471542 |
| Integrator complex subunit 5 OS=Homo sapiens OX=9606 GN=INTS5 PE=1 SV=1 | 213862.9 |
| RNA demethylase ALKBH5 OS=Homo sapiens OX=9606 GN=ALKBH5 PE=1 SV=2 | 24425250 |
| Serine/threonine-protein kinase N3 OS=Homo sapiens OX=9606 GN=PKN3 PE=1 SV=1 | 637303.4 |
| Sideroflexin-4 OS=Homo sapiens OX=9606 GN=SFXN4 PE=1 SV=1 | 1388865 |
| Peptidyl-prolyl cis-trans isomerase (Fragment) OS=Homo sapiens OX=9606 GN=PPIG PE=2 SV=1 | 9779941 |
| Pre-mRNA-processing-splicing factor 8 OS=Homo sapiens OX=9606 GN=PRPF8 PE=1 SV=2 | 65614518 |
| Transcription initiation factor TFIID subunit 2 OS=Homo sapiens OX=9606 GN=TAF2 PE=1 SV=3 | 14166929 |
| Coiled-coil and C2 domain-containing protein 1A OS=Homo sapiens OX=9606 GN=CC2D1A PE=1 SV=1 | 6768239 |
| 39S ribosomal protein L14, mitochondrial OS=Homo sapiens OX=9606 GN=MRPL14 PE=1 SV=1 | 4019509 |
| Parafibromin OS=Homo sapiens OX=9606 GN=CDC73 PE=1 SV=1 | 16517752 |
| Putative ATP-dependent RNA helicase DHX57 OS=Homo sapiens OX=9606 GN=DHX57 PE=1 SV=2 | 13704157 |
| THRAP3 protein (Fragment) OS=Homo sapiens OX=9606 GN=THRAP3 PE=2 SV=1 | 7090891 |
| Mitochondrial mRNA pseudouridine synthase RPUSD3 OS=Homo sapiens OX=9606 GN=RPUSD3 PE=1 SV=3 | 6139899 |
| BRD4 protein (Fragment) OS=Homo sapiens OX=9606 GN=BRD4 PE=2 SV=1 | 1196695 |
| Cysteine-rich DPF motif domain-containing protein 1 OS=Homo sapiens OX=9606 GN=CDPF1 PE=1 SV=1 | 630550.9 |
| Glutathione peroxidase OS=Homo sapiens OX=9606 GN=GPX1 PE=2 SV=1 | 945989.3 |
| DNA oxidative demethylase ALKBH2 OS=Homo sapiens OX=9606 GN=ALKBH2 PE=1 SV=1 | 2298901 |
| Uncharacterized protein DKFZp686I15196 OS=Homo sapiens OX=9606 GN=DKFZp686I15196 PE=2 SV=1 | 5409555 |
| RFC5 protein (Fragment) OS=Homo sapiens OX=9606 GN=RFC5 PE=2 SV=1 | 6169334 |
| Keratin, type II cytoskeletal 80 OS=Homo sapiens OX=9606 GN=KRT80 PE=1 SV=2 | 255977.7 |
| Polyadenylate-binding protein OS=Homo sapiens OX=9606 GN=PABPC4 PE=2 SV=1 | 1.96E+08 |
| 60S ribosomal protein L14 OS=Homo sapiens OX=9606 GN=RPL14 PE=1 SV=1 | 34143720 |
| U11/U12 small nuclear ribonucleoprotein 48 kDa protein OS=Homo sapiens OX=9606 GN=SNRNP48 PE=1 SV=2 | 500080.7 |
| GRB2 protein (Fragment) OS=Homo sapiens OX=9606 GN=GRB2 PE=2 SV=1 | 1236795 |
| SRPR protein OS=Homo sapiens OX=9606 GN=SRPR PE=2 SV=1 | 1240562 |
| Elongator complex protein 2 OS=Homo sapiens OX=9606 GN=ELP2 PE=1 SV=2 | 6053091 |
| Serine/threonine-protein phosphatase OS=Homo sapiens OX=9606 GN=PPP2CA PE=2 SV=1 | 1216323 |
| BAF53A protein OS=Homo sapiens OX=9606 GN=BAF53A PE=2 SV=1 | 6160012 |
| G3BP protein OS=Homo sapiens OX=9606 GN=G3BP PE=2 SV=1 | 7.61E+08 |
| HOXA5 protein (Fragment) OS=Homo sapiens OX=9606 GN=HOXA5 PE=2 SV=1 | 6324736 |
| Acetyl-coenzyme A synthetase (Fragment) OS=Homo sapiens OX=9606 GN=ACSS2 PE=2 SV=1 | 37237488 |
| 60S ribosomal protein L7-like 1 OS=Homo sapiens OX=9606 GN=RPL7L1 PE=1 SV=2 | 4693626 |
| Zinc finger protein 787 OS=Homo sapiens OX=9606 GN=ZNF787 PE=1 SV=4 | 2045425 |
| Uncharacterized protein DKFZp686A111 OS=Homo sapiens OX=9606 GN=DKFZp686A111 PE=2 SV=1 | 8134339 |
| Protein virilizer homolog OS=Homo sapiens OX=9606 GN=VIRMA PE=1 SV=2 | 37973441 |
| AP complex subunit beta OS=Homo sapiens OX=9606 GN=DKFZp781K0743 PE=2 SV=1 | 10611724 |
| Calcium-transporting ATPase OS=Homo sapiens OX=9606 GN=DKFZp686M088 PE=2 SV=1 | 871743.4 |
| Uncharacterized protein DKFZp686P12272 OS=Homo sapiens OX=9606 GN=DKFZp686P12272 PE=4 SV=2 | 4308940 |
| U3 small nucleolar RNA-associated protein 25 homolog OS=Homo sapiens OX=9606 GN=UTP25 PE=1 SV=2 | 1513804 |
| Uncharacterized protein DKFZp686P17171 OS=Homo sapiens OX=9606 GN=DKFZp686P17171 PE=2 SV=1 | 21384059 |
| Partitioning defective 3 homolog OS=Homo sapiens OX=9606 GN=PARD3 PE=1 SV=1 | 830649.3 |
| Lysine-specific demethylase 9 OS=Homo sapiens OX=9606 GN=RSBN1 PE=1 SV=2 | 7528096 |
| Focadhesin OS=Homo sapiens OX=9606 GN=FOCAD PE=1 SV=1 | 37113367 |
| 60S ribosomal protein L11 OS=Homo sapiens OX=9606 GN=RPL11 PE=1 SV=2 | 6.62E+08 |
| Oleoyl-[acyl-carrier-protein] hydrolase OS=Homo sapiens OX=9606 GN=OLAH PE=1 SV=1 | 401888.3 |
| Pre-mRNA-splicing factor 38B OS=Homo sapiens OX=9606 GN=PRPF38B PE=1 SV=1 | 2470622 |
| Regulation of nuclear pre-mRNA domain-containing protein 2 OS=Homo sapiens OX=9606 GN=RPRD2 PE=1 SV=1 | 813056.7 |
| B lymphoma Mo-MLV insertion region (Mouse) OS=Homo sapiens OX=9606 PE=2 SV=1 | 1051328 |
| Cancer-related nucleoside-triphosphatase OS=Homo sapiens OX=9606 GN=NTPCR PE=1 SV=1 | 2506086 |
| E3 ubiquitin-protein ligase RNF220 (Fragment) OS=Homo sapiens OX=9606 GN=RNF220 PE=1 SV=1 | 1140343 |
| SH3 and PX domain-containing protein 2A OS=Homo sapiens OX=9606 GN=SH3PXD2A PE=1 SV=1 | 451612.2 |
| RNA-binding protein 26 OS=Homo sapiens OX=9606 GN=RBM26 PE=1 SV=3 | 2.27E+08 |
| Serine/arginine-rich-splicing factor 11 (Fragment) OS=Homo sapiens OX=9606 GN=SRSF11 PE=1 SV=1 | 8870982 |
| Skin-specific protein 32 OS=Homo sapiens OX=9606 GN=XP32 PE=1 SV=1 | 4125525 |
| Keratinocyte proline-rich protein OS=Homo sapiens OX=9606 GN=KPRP PE=1 SV=1 | 8169591 |
| Ubiquitin-associated protein 2 OS=Homo sapiens OX=9606 GN=UBAP2 PE=1 SV=1 | 93821214 |
| Calmodulin-regulated spectrin-associated protein 1 OS=Homo sapiens OX=9606 GN=CAMSAP1 PE=1 SV=2 | 2033705 |
| G patch domain-containing protein 4 OS=Homo sapiens OX=9606 GN=GPATCH4 PE=1 SV=2 | 10623302 |
| OTU domain-containing protein 3 OS=Homo sapiens OX=9606 GN=OTUD3 PE=1 SV=1 | 5973591 |
| Zinc finger CCCH domain-containing protein 13 OS=Homo sapiens OX=9606 GN=ZC3H13 PE=1 SV=1 | 45509833 |
| snRNA-activating protein complex subunit 4 OS=Homo sapiens OX=9606 GN=SNAPC4 PE=1 SV=1 | 314335.8 |
| Centrosomal protein of 170 kDa OS=Homo sapiens OX=9606 GN=CEP170 PE=1 SV=1 | 32610026 |
| RNA helicase OS=Homo sapiens OX=9606 GN=DDX39B PE=1 SV=2 | 8279322 |
| Exosome complex component MTR3 OS=Homo sapiens OX=9606 GN=EXOSC6 PE=1 SV=1 | 7424767 |
| Heterogeneous nuclear ribonucleoprotein U OS=Homo sapiens OX=9606 GN=HNRNPU PE=1 SV=9 | 923579.3 |
| Ectonucleoside triphosphate diphosphohydrolase 6 OS=Homo sapiens OX=9606 GN=ENTPD6 PE=1 SV=2 | 3017163 |
| Histone H2B type 2-F OS=Homo sapiens OX=9606 GN=H2BC18 PE=1 SV=3 | 4.56E+08 |
| Deoxynucleotidyltransferase terminal-interacting protein 2 OS=Homo sapiens OX=9606 GN=DNTTIP2 PE=1 SV=2 | 619519.9 |
| NKAP-like protein OS=Homo sapiens OX=9606 GN=NKAPL PE=1 SV=3 | 1242748 |
| Ribosomal RNA-processing protein 43 OS=Homo sapiens OX=9606 GN=DKFZp564C0482 PE=3 SV=1 | 419665.3 |
| Intracellular hyaluronan-binding protein 4 OS=Homo sapiens OX=9606 GN=HABP4 PE=1 SV=1 | 5864513 |
| Discs, large homolog 3 (Neuroendocrine-dlg, Drosophila), isoform CRA_b OS=Homo sapiens OX=9606 GN=DLG3 PE=1 SV=1 | 680874.6 |
| Testis-expressed protein 30 OS=Homo sapiens OX=9606 GN=TEX30 PE=2 SV=1 |  |
| RRP12-like protein OS=Homo sapiens OX=9606 GN=RRP12 PE=1 SV=2 | 14586210 |
| Protein PRRC2B OS=Homo sapiens OX=9606 GN=PRRC2B PE=1 SV=2 | 5929222 |
| RNA helicase OS=Homo sapiens OX=9606 GN=MOV10 PE=1 SV=1 | 32854717 |
| Uncharacterized protein DKFZp686E1893 OS=Homo sapiens OX=9606 GN=DKFZp686E1893 PE=2 SV=1 | 14336791 |
| Protein FAM76B OS=Homo sapiens OX=9606 GN=FAM76B PE=1 SV=3 | 2613604 |
| Melanoma-associated antigen D2 OS=Homo sapiens OX=9606 GN=MAGED2 PE=1 SV=2 | 1519590 |
| DnaJ homolog subfamily C member 21 OS=Homo sapiens OX=9606 GN=DNAJC21 PE=1 SV=2 | 2771450 |
| Filaggrin-2 OS=Homo sapiens OX=9606 GN=FLG2 PE=1 SV=1 | 6951725 |
| Fibrous sheath-interacting protein 2 OS=Homo sapiens OX=9606 GN=FSIP2 PE=2 SV=4 | 597109.2 |
| Nucleolar MIF4G domain-containing protein 1 OS=Homo sapiens OX=9606 GN=NOM1 PE=1 SV=1 | 28360393 |
| Importin subunit alpha OS=Homo sapiens OX=9606 GN=KPNA1 PE=2 SV=1 | 10920744 |
| DBIRD complex subunit ZNF326 OS=Homo sapiens OX=9606 GN=ZNF326 PE=1 SV=2 |  |
| Sigma intracellular receptor 2 OS=Homo sapiens OX=9606 GN=TMEM97 PE=1 SV=1 | 771520.1 |
| RNA binding motif protein 5 variant (Fragment) OS=Homo sapiens OX=9606 PE=2 SV=1 | 851501.8 |
| T-complex protein 1 subunit gamma (Fragment) OS=Homo sapiens OX=9606 PE=2 SV=1 | 1482761 |
| Tripartite motif-containing 25 variant (Fragment) OS=Homo sapiens OX=9606 PE=2 SV=1 | 1826781 |
| 60S ribosomal protein L21 (Fragment) OS=Homo sapiens OX=9606 PE=2 SV=1 | 1.27E+08 |
| Thyroid hormone receptor interactor 3 variant (Fragment) OS=Homo sapiens OX=9606 PE=2 SV=1 | 3049615 |
| Splicing factor, arginine/serine-rich 10 (Transformer 2 homolog, Drosophila) variant (Fragment) OS=Homo sapiens OX=9606 PE=2 SV=1 | 61238341 |
| Activated RNA polymerase II transcriptional coactivator p15 (Fragment) OS=Homo sapiens OX=9606 PE=2 SV=1 | 1243096 |
| DNA polymerase (Fragment) OS=Homo sapiens OX=9606 PE=2 SV=1 | 1048362 |
| Transducin beta-like 1X variant (Fragment) OS=Homo sapiens OX=9606 PE=2 SV=1 | 508152.6 |
| CS0DF038YO05 variant (Fragment) OS=Homo sapiens OX=9606 PE=3 SV=1 | 25822304 |
| Low molecular weight cytosolic acid phosphatase (Fragment) OS=Homo sapiens OX=9606 PE=2 SV=1 | 2097168 |
| Serine/threonine-protein kinase PLK (Fragment) OS=Homo sapiens OX=9606 PE=2 SV=1 |  |
| Adenosine deaminase, RNA-specific isoform ADAR-a variant (Fragment) OS=Homo sapiens OX=9606 PE=2 SV=1 | 4229328 |
| SWI/SNF related, matrix associated, actin dependent regulator of chromatin, subfamily c, member 1 OS=Homo sapiens OX=9606 GN=SMARCC1 PE=2 SV=1 | 3716517 |
| Serine/threonine-protein phosphatase 2A 55 kDa regulatory subunit B OS=Homo sapiens OX=9606 PE=2 SV=1 | 1397703 |
| PDCD7 protein (Fragment) OS=Homo sapiens OX=9606 GN=PDCD7 PE=2 SV=1 | 851582.1 |
| Glutaminyl-tRNA synthetase (Fragment) OS=Homo sapiens OX=9606 PE=2 SV=1 | 14150086 |
| Elongation factor 1-alpha (Fragment) OS=Homo sapiens OX=9606 PE=2 SV=1 | 1.33E+08 |
| Splicing factor 3a, subunit 3 variant (Fragment) OS=Homo sapiens OX=9606 PE=2 SV=1 | 8527178 |
| Replication factor C 3 isoform 1 variant (Fragment) OS=Homo sapiens OX=9606 PE=2 SV=1 | 8872357 |
| Eukaryotic translation initiation factor 3, subunit 3 gamma, 40kDa variant (Fragment) OS=Homo sapiens OX=9606 PE=2 SV=1 | 38238269 |
| RNA helicase (Fragment) OS=Homo sapiens OX=9606 PE=2 SV=1 | 16445003 |
| Pyrroline-5-carboxylate reductase 3 OS=Homo sapiens OX=9606 GN=PYCR3 PE=1 SV=3 |  |
| U4/U6.U5 tri-snRNP-associated protein 2 OS=Homo sapiens OX=9606 GN=USP39 PE=1 SV=2 | 21947256 |
| Wolf-Hirschhorn syndrome candidate 2 protein variant (Fragment) OS=Homo sapiens OX=9606 PE=2 SV=1 | 7566820 |
| Solute carrier family 25, member 13 (Citrin) variant (Fragment) OS=Homo sapiens OX=9606 PE=2 SV=1 | 7319122 |
| FUS interacting protein (Serine-arginine rich) 1 isoform 2 variant (Fragment) OS=Homo sapiens OX=9606 PE=2 SV=1 | 19040158 |
| Beta actin variant (Fragment) OS=Homo sapiens OX=9606 PE=2 SV=1 | 90656057 |
| Small nuclear ribonucleoprotein polypeptide A' variant (Fragment) OS=Homo sapiens OX=9606 PE=2 SV=1 | 53797525 |
| Small nuclear ribonucleoprotein polypeptide C variant (Fragment) OS=Homo sapiens OX=9606 PE=2 SV=1 | 1044743 |
| Serine/arginine-rich splicing factor 2 (Fragment) OS=Homo sapiens OX=9606 PE=2 SV=1 | 11522895 |
| DNA replication licensing factor MCM5 (Fragment) OS=Homo sapiens OX=9606 PE=2 SV=1 | 4380276 |
| Daxx (Fragment) OS=Homo sapiens OX=9606 PE=2 SV=1 | 82193018 |
| Zinc finger protein 444 variant (Fragment) OS=Homo sapiens OX=9606 PE=2 SV=1 | 2290512 |
| Heterogeneous nuclear ribonucleoprotein H3 isoform a variant (Fragment) OS=Homo sapiens OX=9606 PE=2 SV=1 | 11961883 |
| Nuclear cap-binding protein subunit 3 OS=Homo sapiens OX=9606 GN=NCBP3 PE=1 SV=2 | 19142442 |
| Cleavage and polyadenylation specific factor 3, 73kDa variant (Fragment) OS=Homo sapiens OX=9606 PE=2 SV=1 | 1.09E+08 |
| Centrosomal protein of 55 kDa OS=Homo sapiens OX=9606 GN=CEP55 PE=1 SV=3 | 3510952 |
| MUS81 endonuclease homolog (Fragment) OS=Homo sapiens OX=9606 PE=2 SV=1 | 584750.6 |
| Dolichyl-diphosphooligosaccharide--protein glycosyltransferase subunit 1 (Fragment) OS=Homo sapiens OX=9606 PE=2 SV=1 | 21476995 |
| Protein FAM98B OS=Homo sapiens OX=9606 GN=FAM98B PE=1 SV=2 | 3540877 |
| von Willebrand factor A domain-containing protein 3B OS=Homo sapiens OX=9606 GN=VWA3B PE=1 SV=3 | 741154.5 |
| Uncharacterized protein R3HDM (Fragment) OS=Homo sapiens OX=9606 GN=R3HDM PE=4 SV=1 | 1014372 |
| Histone cluster 1, H1e OS=Homo sapiens OX=9606 GN=HIST1H1E PE=2 SV=1 | 4261713 |
| TBC1 domain family member 10B OS=Homo sapiens OX=9606 GN=TBC1D10B PE=1 SV=3 | 28824875 |
| La-related protein 7 OS=Homo sapiens OX=9606 GN=LARP7 PE=1 SV=1 | 5762448 |
| Small nuclear ribonucleoprotein G OS=Homo sapiens OX=9606 GN=SNRPG PE=1 SV=1 | 5510465 |
| Eukaryotic translation initiation factor 4E OS=Homo sapiens OX=9606 GN=EIF4E PE=2 SV=1 | 5052965 |
| RBM1 (Fragment) OS=Homo sapiens OX=9606 PE=4 SV=1 | 575355.4 |
| WD40 repeat-containing protein SMU1 OS=Homo sapiens OX=9606 GN=SMU1 PE=1 SV=2 | 3785077 |
| Polynucleotide adenylyltransferase (Fragment) OS=Homo sapiens OX=9606 GN=PAPOLG PE=2 SV=1 | 1788493 |
| Pre-rRNA-processing protein TSR1 homolog OS=Homo sapiens OX=9606 GN=TSR1 PE=1 SV=1 | 1.2E+08 |
| ABC50 protein OS=Homo sapiens OX=9606 GN=ABCF1 PE=4 SV=1 | 1.08E+08 |
| Lysine-rich nucleolar protein 1 OS=Homo sapiens OX=9606 GN=KNOP1 PE=1 SV=1 | 5839922 |
| Histone H2A type 2-C OS=Homo sapiens OX=9606 GN=H2AC20 PE=1 SV=4 | 2.34E+08 |
| Histone H3.1t OS=Homo sapiens OX=9606 GN=H3-4 PE=1 SV=3 | 2E+08 |
| Serine/arginine-rich splicing factor 7 OS=Homo sapiens OX=9606 GN=SRSF7 PE=1 SV=1 | 1.65E+08 |
| DNA damage-binding protein 1 OS=Homo sapiens OX=9606 GN=DDB1 PE=1 SV=1 | 1491372 |
| Programmed cell death protein 2 OS=Homo sapiens OX=9606 GN=PDCD2 PE=1 SV=2 | 350242.5 |
| Vacuolar protein sorting-associated protein 72 homolog OS=Homo sapiens OX=9606 GN=VPS72 PE=1 SV=1 | 447853.8 |
| ELAV-like protein 1 OS=Homo sapiens OX=9606 GN=ELAVL1 PE=1 SV=2 | 25151069 |
| Transformation-related protein (Fragment) OS=Homo sapiens OX=9606 PE=2 SV=1 | 2325432 |
| Mediator of RNA polymerase II transcription subunit 1 OS=Homo sapiens OX=9606 GN=MED1 PE=1 SV=4 | 2164305 |
| Pachytene checkpoint protein 2 homolog OS=Homo sapiens OX=9606 GN=TRIP13 PE=1 SV=2 | 392126.1 |
| Translocating chain-associated membrane protein 1 OS=Homo sapiens OX=9606 GN=TRAM1 PE=1 SV=3 | 286311.9 |
| Transcription initiation factor TFIID subunit 7 OS=Homo sapiens OX=9606 GN=TAF7 PE=1 SV=1 | 509965.1 |
| Surfeit locus protein 2 OS=Homo sapiens OX=9606 GN=SURF2 PE=1 SV=3 | 683809.8 |
| Splicing factor 3A subunit 1 OS=Homo sapiens OX=9606 GN=SF3A1 PE=1 SV=1 | 21967584 |
| Scaffold attachment factor B1 OS=Homo sapiens OX=9606 GN=SAFB PE=1 SV=4 | 26149227 |
| Splicing factor 3B subunit 3 OS=Homo sapiens OX=9606 GN=SF3B3 PE=1 SV=4 | 50947316 |
| Mitochondrial import receptor subunit TOM20 homolog OS=Homo sapiens OX=9606 GN=TOMM20 PE=1 SV=1 | 26452164 |
| Elongin-B OS=Homo sapiens OX=9606 GN=ELOB PE=1 SV=1 | 2711619 |
| Poly(rC)-binding protein 1 OS=Homo sapiens OX=9606 GN=PCBP1 PE=1 SV=2 | 66739752 |
| Leucine-rich repeat-containing protein 41 OS=Homo sapiens OX=9606 GN=LRRC41 PE=1 SV=3 | 3036080 |
| Lethal(2) giant larvae protein homolog 1 OS=Homo sapiens OX=9606 GN=LLGL1 PE=1 SV=3 | 211534.3 |
| Periodic tryptophan protein 2 homolog OS=Homo sapiens OX=9606 GN=PWP2 PE=2 SV=2 | 1459634 |
| Small nuclear ribonucleoprotein-associated protein OS=Homo sapiens OX=9606 GN=SNRPB PE=2 SV=1 | 1.45E+08 |
| Polycystic kidney disease-associated protein OS=Homo sapiens OX=9606 GN=PKD1 PE=3 SV=1 |  |
| [Pyruvate dehydrogenase (acetyl-transferring)] kinase isozyme 3, mitochondrial OS=Homo sapiens OX=9606 GN=PDK3 PE=1 SV=1 | 341492.9 |
| IQ calmodulin-binding motif-containing protein 1 OS=Homo sapiens OX=9606 GN=IQCB1 PE=1 SV=1 | 1987860 |
| Ribosome biogenesis regulatory protein homolog OS=Homo sapiens OX=9606 GN=RRS1 PE=1 SV=2 | 3208263 |
| Lysine--tRNA ligase OS=Homo sapiens OX=9606 GN=KARS1 PE=1 SV=3 | 3514975 |
| Exosome complex component RRP42 OS=Homo sapiens OX=9606 GN=EXOSC7 PE=1 SV=3 | 3724503 |
| Importin subunit beta-1 OS=Homo sapiens OX=9606 GN=KPNB1 PE=1 SV=2 | 2590791 |
| Zinc finger protein 638 OS=Homo sapiens OX=9606 GN=ZNF638 PE=1 SV=2 | 34189677 |
| Kinesin-like protein KIF22 OS=Homo sapiens OX=9606 GN=KIF22 PE=1 SV=5 | 452517.3 |
| Chromobox protein homolog 2 OS=Homo sapiens OX=9606 GN=CBX2 PE=1 SV=2 | 951941.4 |
| Ribosome biogenesis protein BMS1 homolog OS=Homo sapiens OX=9606 GN=BMS1 PE=1 SV=1 | 11216427 |
| Ribosomal RNA processing protein 1 homolog B OS=Homo sapiens OX=9606 GN=RRP1B PE=1 SV=3 | 2428543 |
| Separin OS=Homo sapiens OX=9606 GN=ESPL1 PE=1 SV=3 | 40630019 |
| Plastin-1 OS=Homo sapiens OX=9606 GN=PLS1 PE=1 SV=2 | 600631.3 |
| Caprin-1 OS=Homo sapiens OX=9606 GN=CAPRIN1 PE=1 SV=2 | 6.24E+08 |
| Protein FRG1 OS=Homo sapiens OX=9606 GN=FRG1 PE=1 SV=1 | 13084648 |
| Ensconsin OS=Homo sapiens OX=9606 GN=MAP7 PE=1 SV=1 | 8753193 |
| Ubiquitin-associated protein 2-like OS=Homo sapiens OX=9606 GN=UBAP2L PE=1 SV=2 | 4.7E+08 |
| Probable ATP-dependent RNA helicase DHX34 OS=Homo sapiens OX=9606 GN=DHX34 PE=1 SV=2 | 4826430 |
| Cytoskeleton-associated protein 5 OS=Homo sapiens OX=9606 GN=CKAP5 PE=1 SV=3 | 25101880 |
| Bystin OS=Homo sapiens OX=9606 GN=BYSL PE=1 SV=3 | 83511543 |
| General transcription factor IIH subunit 2 OS=Homo sapiens OX=9606 GN=GTF2H2 PE=1 SV=1 | 820469.4 |
| Exosome complex component RRP4 OS=Homo sapiens OX=9606 GN=EXOSC2 PE=1 SV=2 | 6355462 |
| Bleomycin hydrolase OS=Homo sapiens OX=9606 GN=BLMH PE=1 SV=1 | 4720652 |
| Nucleolar GTP-binding protein 2 OS=Homo sapiens OX=9606 GN=GNL2 PE=1 SV=1 | 37131875 |
| Mannosyl-oligosaccharide glucosidase OS=Homo sapiens OX=9606 GN=MOGS PE=1 SV=5 | 43094853 |
| KRR1 small subunit processome component homolog OS=Homo sapiens OX=9606 GN=KRR1 PE=1 SV=4 | 1540636 |
| Transformer-2 protein homolog alpha OS=Homo sapiens OX=9606 GN=TRA2A PE=1 SV=1 | 17368297 |
| Diacylglycerol kinase zeta OS=Homo sapiens OX=9606 GN=DGKZ PE=1 SV=4 | 3506424 |
| SNW domain-containing protein 1 OS=Homo sapiens OX=9606 GN=SNW1 PE=1 SV=1 | 20577909 |
| Histone deacetylase 1 OS=Homo sapiens OX=9606 GN=HDAC1 PE=1 SV=1 | 11669953 |
| Splicing factor 3B subunit 2 OS=Homo sapiens OX=9606 GN=SF3B2 PE=1 SV=2 | 45711597 |
| Serine/arginine-rich splicing factor 9 OS=Homo sapiens OX=9606 GN=SRSF9 PE=1 SV=1 | 58524815 |
| Syntaxin-5 OS=Homo sapiens OX=9606 GN=STX5 PE=1 SV=2 | 742345.7 |
| Chromobox protein homolog 3 OS=Homo sapiens OX=9606 GN=CBX3 PE=1 SV=4 | 4832089 |
| Aminoacyl tRNA synthase complex-interacting multifunctional protein 2 OS=Homo sapiens OX=9606 GN=AIMP2 PE=1 SV=2 | 1378621 |
| Heterogeneous nuclear ribonucleoprotein A0 OS=Homo sapiens OX=9606 GN=HNRNPA0 PE=1 SV=1 | 4636364 |
| Cell division cycle protein 16 homolog OS=Homo sapiens OX=9606 GN=CDC16 PE=1 SV=2 | 1069483 |
| Cleavage stimulation factor subunit 3 OS=Homo sapiens OX=9606 GN=CSTF3 PE=1 SV=1 | 10396931 |
| Transcriptional repressor NF-X1 OS=Homo sapiens OX=9606 GN=NFX1 PE=1 SV=2 | 306275.9 |
| Tyrosine-protein phosphatase non-receptor type 13 OS=Homo sapiens OX=9606 GN=PTPN13 PE=1 SV=2 | 1926927 |
| Interleukin enhancer-binding factor 3 OS=Homo sapiens OX=9606 GN=ILF3 PE=1 SV=3 |  |
| Aminoacyl tRNA synthase complex-interacting multifunctional protein 1 OS=Homo sapiens OX=9606 GN=AIMP1 PE=1 SV=2 | 4476962 |
| Chromodomain-helicase-DNA-binding protein 3 OS=Homo sapiens OX=9606 GN=CHD3 PE=1 SV=3 | 2709061 |
| Splicing factor, suppressor of white-apricot homolog OS=Homo sapiens OX=9606 GN=SFSWAP PE=1 SV=3 | 10970668 |
| Kinesin heavy chain isoform 5A OS=Homo sapiens OX=9606 GN=KIF5A PE=1 SV=2 | 4555432 |
| Cell division cycle protein 20 homolog OS=Homo sapiens OX=9606 GN=CDC20 PE=1 SV=2 | 11507066 |
| Aspartyl/asparaginyl beta-hydroxylase OS=Homo sapiens OX=9606 GN=ASPH PE=1 SV=3 | 1712960 |
| Cleavage and polyadenylation specificity factor subunit 1 OS=Homo sapiens OX=9606 GN=CPSF1 PE=1 SV=2 | 1.51E+08 |
| Heat shock 70 kDa protein 14 OS=Homo sapiens OX=9606 GN=HSPA14 PE=1 SV=1 | 2198464 |
| ATP synthase subunit beta (Fragment) OS=Homo sapiens OX=9606 GN=ATP5B PE=2 SV=1 | 1137793 |
| Splicing factor, arginine/serine-rich 15 OS=Homo sapiens OX=9606 GN=SFRS15 PE=2 SV=1 | 10368159 |
| Nuclear cap-binding protein subunit 1 OS=Homo sapiens OX=9606 GN=NCBP1 PE=1 SV=1 | 2235616 |
| Histone-binding protein RBBP4 OS=Homo sapiens OX=9606 GN=RBBP4 PE=1 SV=3 | 37754464 |
| RNA cytosine C(5)-methyltransferase NSUN2 OS=Homo sapiens OX=9606 GN=NSUN2 PE=1 SV=2 | 6190051 |
| Calmodulin-regulated spectrin-associated protein 2 OS=Homo sapiens OX=9606 GN=CAMSAP2 PE=1 SV=3 | 2482806 |
| FACT complex subunit SSRP1 OS=Homo sapiens OX=9606 GN=SSRP1 PE=1 SV=1 | 4083527 |
| ATP-dependent RNA helicase A OS=Homo sapiens OX=9606 GN=DHX9 PE=1 SV=4 | 57006331 |
| Protein-glutamine gamma-glutamyltransferase E OS=Homo sapiens OX=9606 GN=TGM3 PE=1 SV=4 | 3817202 |
| KH domain-containing, RNA-binding, signal transduction-associated protein 1 OS=Homo sapiens OX=9606 GN=KHDRBS1 PE=1 SV=1 | 1.26E+09 |
| Peroxiredoxin-1 OS=Homo sapiens OX=9606 GN=PRDX1 PE=1 SV=1 | 70081538 |
| Synaptic functional regulator FMR1 OS=Homo sapiens OX=9606 GN=FMR1 PE=1 SV=1 | 92098968 |
| mRNA (guanine-N(7)-)-methyltransferase (Fragment) OS=Homo sapiens OX=9606 GN=RNMT PE=2 SV=1 | 302041.5 |
| ADD2 protein (Fragment) OS=Homo sapiens OX=9606 GN=ADD2 PE=2 SV=1 | 3389868 |
| SF3A2 protein (Fragment) OS=Homo sapiens OX=9606 GN=SF3A2 PE=2 SV=1 | 7388111 |
| PREB protein OS=Homo sapiens OX=9606 GN=PREB PE=2 SV=1 | 777279.6 |
| Double-strand break repair protein MRE11 (Fragment) OS=Homo sapiens OX=9606 GN=MRE11A PE=2 SV=1 | 11803830 |
| VRK2 protein (Fragment) OS=Homo sapiens OX=9606 GN=VRK2 PE=2 SV=1 | 1541268 |
| GTF3C4 protein (Fragment) OS=Homo sapiens OX=9606 GN=GTF3C4 PE=2 SV=1 | 3208160 |
| Keratin, type I cytoskeletal 17 OS=Homo sapiens OX=9606 GN=KRT17 PE=1 SV=2 | 10658187 |
| CCAAT/enhancer-binding protein zeta OS=Homo sapiens OX=9606 GN=CEBPZ PE=1 SV=3 | 288305.5 |
| Protein ENL OS=Homo sapiens OX=9606 GN=MLLT1 PE=1 SV=2 | 871226.4 |
| Mitochondrial 2-oxoglutarate/malate carrier protein OS=Homo sapiens OX=9606 GN=SLC25A11 PE=1 SV=3 | 6734093 |
| DNA topoisomerase 2-beta OS=Homo sapiens OX=9606 GN=TOP2B PE=1 SV=3 | 3222160 |
| 60S ribosomal protein L6 OS=Homo sapiens OX=9606 GN=RPL6 PE=1 SV=3 | 2.69E+08 |
| Desmoglein-1 OS=Homo sapiens OX=9606 GN=DSG1 PE=1 SV=2 | 10544341 |
| A-kinase anchor protein 17A OS=Homo sapiens OX=9606 GN=AKAP17A PE=1 SV=2 | 7889823 |
| OTU domain-containing protein 4 OS=Homo sapiens OX=9606 GN=OTUD4 PE=1 SV=4 | 21540648 |
| Exosome component 10 OS=Homo sapiens OX=9606 GN=EXOSC10 PE=1 SV=2 | 12289908 |
| Splicing factor U2AF 35 kDa subunit OS=Homo sapiens OX=9606 GN=U2AF1 PE=1 SV=3 | 32673951 |
| Heterogeneous nuclear ribonucleoprotein U OS=Homo sapiens OX=9606 GN=HNRNPU PE=1 SV=6 | 1.09E+09 |
| Cdc42 effector protein 1 OS=Homo sapiens OX=9606 GN=CDC42EP1 PE=1 SV=1 | 594393.7 |
| Transcriptional activator protein Pur-alpha OS=Homo sapiens OX=9606 GN=PURA PE=1 SV=2 | 4548638 |
| Cyclin-dependent-like kinase 5 OS=Homo sapiens OX=9606 GN=CDK5 PE=1 SV=3 | 677320.8 |
| Transcription initiation factor IIB OS=Homo sapiens OX=9606 GN=GTF2B PE=1 SV=1 | 604344.3 |
| Vigilin OS=Homo sapiens OX=9606 GN=HDLBP PE=1 SV=2 | 3070412 |
| Serine/arginine-rich splicing factor 3 OS=Homo sapiens OX=9606 GN=SRSF3 PE=1 SV=1 | 96358798 |
| Enhancer of rudimentary homolog OS=Homo sapiens OX=9606 GN=ERH PE=1 SV=1 | 1.08E+08 |
| 60S ribosomal protein L24 OS=Homo sapiens OX=9606 GN=RPL24 PE=1 SV=1 | 4.21E+08 |
| U7 snRNA-associated Sm-like protein LSm11 OS=Homo sapiens OX=9606 GN=LSM11 PE=1 SV=2 | 7409173 |
| 28S ribosomal protein S9, mitochondrial OS=Homo sapiens OX=9606 GN=MRPS9 PE=1 SV=2 | 907965.7 |
| 28S ribosomal protein S6, mitochondrial OS=Homo sapiens OX=9606 GN=MRPS6 PE=1 SV=3 | 690808.1 |
| 28S ribosomal protein S15, mitochondrial OS=Homo sapiens OX=9606 GN=MRPS15 PE=1 SV=1 | 1999878 |
| 28S ribosomal protein S25, mitochondrial OS=Homo sapiens OX=9606 GN=MRPS25 PE=1 SV=1 |  |
| 28S ribosomal protein S22, mitochondrial OS=Homo sapiens OX=9606 GN=MRPS22 PE=1 SV=1 | 5702620 |
| Dermcidin OS=Homo sapiens OX=9606 GN=DCD PE=1 SV=2 | 49576414 |
| Endonuclease III-like protein 1 OS=Homo sapiens OX=9606 GN=NTHL1 PE=1 SV=2 | 6556832 |
| DNA-dependent protein kinase catalytic subunit OS=Homo sapiens OX=9606 GN=PRKDC PE=1 SV=3 | 29394173 |
| Polyhomeotic 2 homolog OS=Homo sapiens OX=9606 GN=HPH2 PE=2 SV=1 | 707341 |
| SRSF protein kinase 2 OS=Homo sapiens OX=9606 GN=SRPK2 PE=1 SV=3 | 9111320 |
| Ribonuclease P protein subunit p30 OS=Homo sapiens OX=9606 GN=RPP30 PE=1 SV=1 | 3141398 |
| Ribonuclease P protein subunit p38 OS=Homo sapiens OX=9606 GN=RPP38 PE=1 SV=2 | 1340166 |
| Y-box-binding protein 1 OS=Homo sapiens OX=9606 GN=YBX1 PE=1 SV=3 | 9570053 |
| Receptor of activated protein C kinase 1 OS=Homo sapiens OX=9606 GN=RACK1 PE=1 SV=3 | 8497325 |
| 40S ribosomal protein S21 OS=Homo sapiens OX=9606 GN=RPS21 PE=1 SV=1 | 1614333 |
| 60S ribosomal protein L38 OS=Homo sapiens OX=9606 GN=RPL38 PE=1 SV=2 | 1.2E+08 |
| 14-3-3 protein zeta/delta OS=Homo sapiens OX=9606 GN=YWHAZ PE=1 SV=1 | 3297000 |
| Ubiquitin-60S ribosomal protein L40 OS=Homo sapiens OX=9606 GN=UBA52 PE=1 SV=2 | 2182701 |
| Ubiquitin-40S ribosomal protein S27a OS=Homo sapiens OX=9606 GN=RPS27A PE=1 SV=2 | 1.17E+08 |
| 60S ribosomal protein L8 OS=Homo sapiens OX=9606 GN=RPL8 PE=1 SV=2 | 8.3E+08 |
| 60S ribosomal protein L10a OS=Homo sapiens OX=9606 GN=RPL10A PE=1 SV=2 | 75462411 |
| 60S ribosomal protein L39 OS=Homo sapiens OX=9606 GN=RPL39 PE=1 SV=2 | 1219130 |
| 40S ribosomal protein S30 OS=Homo sapiens OX=9606 GN=FAU PE=1 SV=1 | 1.79E+08 |
| 40S ribosomal protein S28 OS=Homo sapiens OX=9606 GN=RPS28 PE=1 SV=1 | 11505748 |
| 40S ribosomal protein S25 OS=Homo sapiens OX=9606 GN=RPS25 PE=1 SV=1 | 2.77E+08 |
| 60S ribosomal protein L23 OS=Homo sapiens OX=9606 GN=RPL23 PE=1 SV=1 | 7.36E+08 |
| Histone H4 OS=Homo sapiens OX=9606 GN=H4C1 PE=1 SV=2 | 1.05E+08 |
| 40S ribosomal protein S6 OS=Homo sapiens OX=9606 GN=RPS6 PE=1 SV=1 | 2.1E+09 |
| 60S ribosomal protein L7a OS=Homo sapiens OX=9606 GN=RPL7A PE=1 SV=2 | 3.15E+08 |
| Small nuclear ribonucleoprotein Sm D3 OS=Homo sapiens OX=9606 GN=SNRPD3 PE=1 SV=1 | 4.96E+08 |
| Small nuclear ribonucleoprotein Sm D2 OS=Homo sapiens OX=9606 GN=SNRPD2 PE=1 SV=1 | 2.81E+08 |
| Small nuclear ribonucleoprotein Sm D1 OS=Homo sapiens OX=9606 GN=SNRPD1 PE=1 SV=1 | 1E+08 |
| U6 snRNA-associated Sm-like protein LSm6 OS=Homo sapiens OX=9606 GN=LSM6 PE=1 SV=1 | 2449752 |
| U6 snRNA-associated Sm-like protein LSm3 OS=Homo sapiens OX=9606 GN=LSM3 PE=1 SV=2 | 4095531 |
| Small nuclear ribonucleoprotein F OS=Homo sapiens OX=9606 GN=SNRPF PE=1 SV=1 | 39295929 |
| 40S ribosomal protein S11 OS=Homo sapiens OX=9606 GN=RPS11 PE=1 SV=3 | 1.38E+09 |
| 40S ribosomal protein S13 OS=Homo sapiens OX=9606 GN=RPS13 PE=1 SV=2 | 4.73E+08 |
| 40S ribosomal protein S29 OS=Homo sapiens OX=9606 GN=RPS29 PE=1 SV=2 | 18930554 |
| 40S ribosomal protein S18 OS=Homo sapiens OX=9606 GN=RPS18 PE=1 SV=3 | 8.81E+08 |
| 40S ribosomal protein S23 OS=Homo sapiens OX=9606 GN=RPS23 PE=1 SV=3 | 2.01E+08 |
| 40S ribosomal protein S8 OS=Homo sapiens OX=9606 GN=RPS8 PE=1 SV=2 | 2.82E+08 |
| Serine/threonine-protein phosphatase PP1-beta catalytic subunit OS=Homo sapiens OX=9606 GN=PPP1CB PE=1 SV=3 | 3928581 |
| Serine/threonine-protein phosphatase PP1-alpha catalytic subunit OS=Homo sapiens OX=9606 GN=PPP1CA PE=1 SV=1 | 10481722 |
| 40S ribosomal protein S7 OS=Homo sapiens OX=9606 GN=RPS7 PE=1 SV=1 | 4.65E+08 |
| WD repeat-containing protein 5 OS=Homo sapiens OX=9606 GN=WDR5 PE=1 SV=1 | 1446948 |
| DDB1- and CUL4-associated factor 7 OS=Homo sapiens OX=9606 GN=DCAF7 PE=1 SV=1 | 992736.9 |
| 60S ribosomal protein L37 OS=Homo sapiens OX=9606 GN=RPL37 PE=1 SV=2 | 10757596 |
| 60S ribosomal protein L37a OS=Homo sapiens OX=9606 GN=RPL37A PE=1 SV=2 | 1.02E+08 |
| Protein mago nashi homolog OS=Homo sapiens OX=9606 GN=MAGOH PE=1 SV=1 | 7505313 |
| 60S ribosomal protein L26 OS=Homo sapiens OX=9606 GN=RPL26 PE=1 SV=1 | 5.98E+08 |
| 40S ribosomal protein S3a OS=Homo sapiens OX=9606 GN=RPS3A PE=1 SV=2 | 9.67E+08 |
| Ras-related protein Rab-10 OS=Homo sapiens OX=9606 GN=RAB10 PE=1 SV=1 | 1505878 |
| 40S ribosomal protein S20 OS=Homo sapiens OX=9606 GN=RPS20 PE=1 SV=1 | 90474958 |
| Protein transport protein Sec61 subunit beta OS=Homo sapiens OX=9606 GN=SEC61B PE=1 SV=2 | 6943518 |
| Transcription elongation factor 1 homolog OS=Homo sapiens OX=9606 GN=ELOF1 PE=1 SV=1 | 1225136 |
| Neurotrypsin OS=Homo sapiens OX=9606 GN=PRSS12 PE=2 SV=2 |  |
| C-terminal-binding protein 2 OS=Homo sapiens OX=9606 GN=CTBP2 PE=1 SV=1 | 254096.3 |
| Myc-associated zinc finger protein OS=Homo sapiens OX=9606 GN=MAZ PE=1 SV=1 | 17037230 |
| Methionine--tRNA ligase, cytoplasmic OS=Homo sapiens OX=9606 GN=MARS1 PE=1 SV=2 | 14973088 |
| Heterogeneous nuclear ribonucleoprotein H2 OS=Homo sapiens OX=9606 GN=HNRNPH2 PE=1 SV=1 | 1566842 |
| RNA polymerase II elongation factor ELL OS=Homo sapiens OX=9606 GN=ELL PE=1 SV=1 | 323794.8 |
| Trifunctional enzyme subunit beta, mitochondrial OS=Homo sapiens OX=9606 GN=HADHB PE=1 SV=3 | 11347929 |
| Microfibrillar-associated protein 1 OS=Homo sapiens OX=9606 GN=MFAP1 PE=1 SV=2 | 17910876 |
| Arginine--tRNA ligase, cytoplasmic OS=Homo sapiens OX=9606 GN=RARS1 PE=1 SV=2 | 21899871 |
| Heterogeneous nuclear ribonucleoprotein F OS=Homo sapiens OX=9606 GN=HNRNPF PE=1 SV=3 | 3707157 |
| Nuclear cap-binding protein subunit 2 OS=Homo sapiens OX=9606 GN=NCBP2 PE=1 SV=1 | 1059429 |
| Heterogeneous nuclear ribonucleoprotein A3 OS=Homo sapiens OX=9606 GN=HNRNPA3 PE=1 SV=2 | 79166750 |
| Transcription activator BRG1 OS=Homo sapiens OX=9606 GN=SMARCA4 PE=1 SV=2 | 509249.3 |
| Fragile X mental retardation syndrome-related protein 2 OS=Homo sapiens OX=9606 GN=FXR2 PE=1 SV=2 | 96813750 |
| Fragile X mental retardation syndrome-related protein 1 OS=Homo sapiens OX=9606 GN=FXR1 PE=1 SV=3 | 2.15E+08 |
| T-complex protein 1 subunit delta OS=Homo sapiens OX=9606 GN=CCT4 PE=1 SV=4 | 7203438 |
| T-complex protein 1 subunit theta OS=Homo sapiens OX=9606 GN=CCT8 PE=1 SV=4 | 1584502 |
| Emerin OS=Homo sapiens OX=9606 GN=EMD PE=1 SV=1 | 5884112 |
| Transcription initiation factor TFIID subunit 6 OS=Homo sapiens OX=9606 GN=TAF6 PE=1 SV=1 | 475635.9 |
| RNA-binding protein 25 OS=Homo sapiens OX=9606 GN=RBM25 PE=1 SV=3 | 61458461 |
| YLP motif-containing protein 1 OS=Homo sapiens OX=9606 GN=YLPM1 PE=1 SV=4 | 77218009 |
| Casein kinase I isoform epsilon OS=Homo sapiens OX=9606 GN=CSNK1E PE=1 SV=1 | 7879335 |
| Signal recognition particle 9 kDa protein OS=Homo sapiens OX=9606 GN=SRP9 PE=1 SV=2 | 13863461 |
| Fatty acid synthase OS=Homo sapiens OX=9606 GN=FASN PE=1 SV=3 | 651949 |
| 60S ribosomal protein L34 OS=Homo sapiens OX=9606 GN=RPL34 PE=1 SV=3 | 97275961 |
| ATP synthase subunit O, mitochondrial OS=Homo sapiens OX=9606 GN=ATP5PO PE=1 SV=1 | 6238648 |
| Dolichyl-diphosphooligosaccharide--protein glycosyltransferase subunit STT3A OS=Homo sapiens OX=9606 GN=STT3A PE=1 SV=2 | 295483.3 |
| 40S ribosomal protein S10 OS=Homo sapiens OX=9606 GN=RPS10 PE=1 SV=1 | 1.36E+08 |
| 40S ribosomal protein S9 OS=Homo sapiens OX=9606 GN=RPS9 PE=1 SV=3 | 9.8E+08 |
| Probable 28S rRNA (cytosine(4447)-C(5))-methyltransferase OS=Homo sapiens OX=9606 GN=NOP2 PE=1 SV=2 | 2785353 |
| ATP-dependent DNA helicase Q1 OS=Homo sapiens OX=9606 GN=RECQL PE=1 SV=3 | 896291.5 |
| Proliferation marker protein Ki-67 OS=Homo sapiens OX=9606 GN=MKI67 PE=1 SV=2 | 699397.7 |
| Neuronal vesicle trafficking-associated protein 1 OS=Homo sapiens OX=9606 GN=NSG1 PE=1 SV=1 | 3494929 |
| 60S ribosomal protein L35 OS=Homo sapiens OX=9606 GN=RPL35 PE=1 SV=2 | 1.92E+09 |
| Probable helicase with zinc finger domain OS=Homo sapiens OX=9606 GN=HELZ PE=1 SV=2 | 731288.3 |
| 40S ribosomal protein S27 OS=Homo sapiens OX=9606 GN=RPS27 PE=1 SV=3 | 4.58E+08 |
| Exosome RNA helicase MTR4 OS=Homo sapiens OX=9606 GN=MTREX PE=1 SV=3 | 8540025 |
| Lamina-associated polypeptide 2, isoforms beta/gamma OS=Homo sapiens OX=9606 GN=TMPO PE=1 SV=2 | 54177405 |
| Protein kinase C iota type OS=Homo sapiens OX=9606 GN=PRKCI PE=1 SV=2 | 1994918 |
| Eukaryotic translation initiation factor 2 subunit 3 OS=Homo sapiens OX=9606 GN=EIF2S3 PE=1 SV=3 | 63597546 |
| 60S ribosomal protein L13a OS=Homo sapiens OX=9606 GN=RPL13A PE=1 SV=2 | 2.49E+08 |
| Alpha-taxilin OS=Homo sapiens OX=9606 GN=TXLNA PE=1 SV=3 | 12755443 |
| 60S ribosomal protein L3 OS=Homo sapiens OX=9606 GN=RPL3 PE=1 SV=2 | 2.86E+08 |
| 40S ribosomal protein S19 OS=Homo sapiens OX=9606 GN=RPS19 PE=1 SV=2 | 2.1E+08 |
| Eukaryotic initiation factor 4A-III OS=Homo sapiens OX=9606 GN=EIF4A3 PE=1 SV=4 | 47520102 |
| Coilin OS=Homo sapiens OX=9606 GN=COIL PE=1 SV=1 | 6109425 |
| RNA-binding motif protein, X chromosome OS=Homo sapiens OX=9606 GN=RBMX PE=1 SV=3 | 7.61E+08 |
| Signal recognition particle 14 kDa protein OS=Homo sapiens OX=9606 GN=SRP14 PE=1 SV=2 | 36432027 |
| Serine/threonine-protein phosphatase PP1-gamma catalytic subunit OS=Homo sapiens OX=9606 GN=PPP1CC PE=1 SV=1 | 3781484 |
| 60S ribosomal protein L4 OS=Homo sapiens OX=9606 GN=RPL4 PE=1 SV=5 | 3.58E+08 |
| Keratin, type II cytoskeletal 2 epidermal OS=Homo sapiens OX=9606 GN=KRT2 PE=1 SV=2 | 8.13E+08 |
| Keratin, type I cytoskeletal 9 OS=Homo sapiens OX=9606 GN=KRT9 PE=1 SV=3 | 1.33E+09 |
| Replication factor C subunit 1 OS=Homo sapiens OX=9606 GN=RFC1 PE=1 SV=4 | 4992406 |
| Replication factor C subunit 2 OS=Homo sapiens OX=9606 GN=RFC2 PE=1 SV=3 | 5736160 |
| Replication factor C subunit 4 OS=Homo sapiens OX=9606 GN=RFC4 PE=1 SV=2 | 7561521 |
| Cleavage stimulation factor subunit 2 OS=Homo sapiens OX=9606 GN=CSTF2 PE=1 SV=1 | 9584419 |
| Caspase-14 OS=Homo sapiens OX=9606 GN=CASP14 PE=1 SV=2 | 1457068 |
| DnaJ homolog subfamily A member 1 OS=Homo sapiens OX=9606 GN=DNAJA1 PE=1 SV=2 | 5804256 |
| Lipocalin-1 OS=Homo sapiens OX=9606 GN=LCN1 PE=1 SV=1 | 1234711 |
| 60S ribosomal protein L12 OS=Homo sapiens OX=9606 GN=RPL12 PE=1 SV=1 | 1.89E+08 |
| Thioredoxin-dependent peroxide reductase, mitochondrial OS=Homo sapiens OX=9606 GN=PRDX3 PE=1 SV=3 | 1035955 |
| Peroxiredoxin-5, mitochondrial OS=Homo sapiens OX=9606 GN=PRDX5 PE=1 SV=4 | 495588.9 |
| Elongation factor 1-delta OS=Homo sapiens OX=9606 GN=EEF1D PE=1 SV=5 |  |
| Mitogen-activated protein kinase 1 OS=Homo sapiens OX=9606 GN=MAPK1 PE=1 SV=3 | 1623660 |
| Microtubule-associated protein 4 OS=Homo sapiens OX=9606 GN=MAP4 PE=1 SV=3 | 41307378 |
| DNA-(apurinic or apyrimidinic site) endonuclease OS=Homo sapiens OX=9606 GN=APEX1 PE=1 SV=2 | 3403030 |
| 14-3-3 protein theta OS=Homo sapiens OX=9606 GN=YWHAQ PE=1 SV=1 | 17974233 |
| Elongation factor 1-gamma OS=Homo sapiens OX=9606 GN=EEF1G PE=1 SV=3 | 2958082 |
| 60S ribosomal protein L13 OS=Homo sapiens OX=9606 GN=RPL13 PE=1 SV=4 | 4.28E+08 |
| Probable ATP-dependent RNA helicase DDX6 OS=Homo sapiens OX=9606 GN=DDX6 PE=1 SV=2 | 5.34E+08 |
| ATP synthase subunit alpha, mitochondrial OS=Homo sapiens OX=9606 GN=ATP5F1A PE=1 SV=1 | 3205023 |
| 40S ribosomal protein S12 OS=Homo sapiens OX=9606 GN=RPS12 PE=1 SV=3 | 1.51E+08 |
| Zinc-alpha-2-glycoprotein OS=Homo sapiens OX=9606 GN=AZGP1 PE=1 SV=2 | 769620.3 |
| DNA replication licensing factor MCM3 OS=Homo sapiens OX=9606 GN=MCM3 PE=1 SV=3 | 6760179 |
| Cyclin-dependent kinase 2 OS=Homo sapiens OX=9606 GN=CDK2 PE=1 SV=2 | 288809.9 |
| 40S ribosomal protein S3 OS=Homo sapiens OX=9606 GN=RPS3 PE=1 SV=2 | 8.31E+08 |
| Splicing factor, proline- and glutamine-rich OS=Homo sapiens OX=9606 GN=SFPQ PE=1 SV=2 | 6.48E+09 |
| Small proline-rich protein 2D OS=Homo sapiens OX=9606 GN=SPRR2D PE=2 SV=2 | 1284661 |
| Cornifin-B OS=Homo sapiens OX=9606 GN=SPRR1B PE=1 SV=2 | 2716877 |
| Protein-L-isoaspartate(D-aspartate) O-methyltransferase OS=Homo sapiens OX=9606 GN=PCMT1 PE=1 SV=4 | 2386484 |
| Midkine OS=Homo sapiens OX=9606 GN=MDK PE=1 SV=1 | 1872974 |
| Eukaryotic translation initiation factor 2 subunit 2 OS=Homo sapiens OX=9606 GN=EIF2S2 PE=1 SV=2 | 55652646 |
| E3 ubiquitin-protein ligase TRIM21 OS=Homo sapiens OX=9606 GN=TRIM21 PE=1 SV=1 | 2274948 |
| Nucleolin OS=Homo sapiens OX=9606 GN=NCL PE=1 SV=3 | 58837783 |
| Regulator of chromosome condensation OS=Homo sapiens OX=9606 GN=RCC1 PE=1 SV=1 | 3957768 |
| Protein SON OS=Homo sapiens OX=9606 GN=SON PE=1 SV=4 | 2261029 |
| 60S ribosomal protein L7 OS=Homo sapiens OX=9606 GN=RPL7 PE=1 SV=1 | 1.23E+08 |
| 60S ribosomal protein L35a OS=Homo sapiens OX=9606 GN=RPL35A PE=1 SV=2 | 1.7E+08 |
| Homeobox protein Hox-B9 OS=Homo sapiens OX=9606 GN=HOXB9 PE=1 SV=2 | 8196744 |
| Y-box-binding protein 3 OS=Homo sapiens OX=9606 GN=YBX3 PE=1 SV=4 | 14088415 |
| Histone H1.2 OS=Homo sapiens OX=9606 GN=H1-2 PE=1 SV=2 | 4.01E+08 |
| Desmoplakin OS=Homo sapiens OX=9606 GN=DSP PE=1 SV=3 | 40664769 |
| 40S ribosomal protein S2 OS=Homo sapiens OX=9606 GN=RPS2 PE=1 SV=2 | 8.58E+08 |
| Junction plakoglobin OS=Homo sapiens OX=9606 GN=JUP PE=1 SV=3 | 16920507 |
| Aspartate--tRNA ligase, cytoplasmic OS=Homo sapiens OX=9606 GN=DARS1 PE=1 SV=2 | 42839029 |
| Beta-enolase OS=Homo sapiens OX=9606 GN=ENO3 PE=1 SV=5 | 436210.3 |
| Keratin, type II cytoskeletal 5 OS=Homo sapiens OX=9606 GN=KRT5 PE=1 SV=3 | 2.28E+08 |
| Elongation factor 2 OS=Homo sapiens OX=9606 GN=EEF2 PE=1 SV=4 | 6619360 |
| X-ray repair cross-complementing protein 5 OS=Homo sapiens OX=9606 GN=XRCC5 PE=1 SV=3 | 12811636 |
| Creatine kinase U-type, mitochondrial OS=Homo sapiens OX=9606 GN=CKMT1A PE=1 SV=1 | 3.18E+08 |
| Prolactin-inducible protein OS=Homo sapiens OX=9606 GN=PIP PE=1 SV=1 | 1101549 |
| Nucleoprotein TPR OS=Homo sapiens OX=9606 GN=TPR PE=1 SV=3 | 4802705 |
| ADP/ATP translocase 1 OS=Homo sapiens OX=9606 GN=SLC25A4 PE=1 SV=4 | 17209612 |
| DNA topoisomerase 2-alpha OS=Homo sapiens OX=9606 GN=TOP2A PE=1 SV=3 | 15607471 |
| Heat shock cognate 71 kDa protein OS=Homo sapiens OX=9606 GN=HSPA8 PE=1 SV=1 | 61757284 |
| Endoplasmic reticulum chaperone BiP OS=Homo sapiens OX=9606 GN=HSPA5 PE=1 SV=2 | 12598189 |
| Thioredoxin OS=Homo sapiens OX=9606 GN=TXN PE=1 SV=3 | 5695223 |
| Protein POLR1D, isoform 2 OS=Homo sapiens OX=9606 GN=POLR1D PE=1 SV=1 | 725035.5 |
| Protein FAM200B OS=Homo sapiens OX=9606 GN=FAM200B PE=3 SV=1 | 10416266 |
| Putative cytochrome b-c1 complex subunit Rieske-like protein 1 OS=Homo sapiens OX=9606 GN=UQCRFS1P1 PE=5 SV=1 | 3888346 |
| U1 small nuclear ribonucleoprotein A OS=Homo sapiens OX=9606 GN=SNRPA PE=1 SV=3 | 8103644 |
| 40S ribosomal protein SA OS=Homo sapiens OX=9606 GN=RPSA PE=1 SV=4 | 4798063 |
| Keratin, type I cytoskeletal 16 OS=Homo sapiens OX=9606 GN=KRT16 PE=1 SV=4 | 35633135 |
| Vimentin OS=Homo sapiens OX=9606 GN=VIM PE=1 SV=4 | 1619361 |
| U1 small nuclear ribonucleoprotein 70 kDa OS=Homo sapiens OX=9606 GN=SNRNP70 PE=1 SV=2 | 85038838 |
| U2 small nuclear ribonucleoprotein B'' OS=Homo sapiens OX=9606 GN=SNRPB2 PE=1 SV=1 | 72221454 |
| Heat shock protein HSP 90-beta OS=Homo sapiens OX=9606 GN=HSP90AB1 PE=1 SV=4 | 13746783 |
| Thrombospondin-1 OS=Homo sapiens OX=9606 GN=THBS1 PE=1 SV=2 | 2162506 |
| Heat shock protein HSP 90-alpha OS=Homo sapiens OX=9606 GN=HSP90AA1 PE=1 SV=5 | 1068339 |
| Bifunctional glutamate/proline--tRNA ligase OS=Homo sapiens OX=9606 GN=EPRS1 PE=1 SV=5 | 69976961 |
| Annexin A2 OS=Homo sapiens OX=9606 GN=ANXA2 PE=1 SV=2 | 8934495 |
| Histone H1.0 OS=Homo sapiens OX=9606 GN=H1-0 PE=1 SV=3 | 42351546 |
| Cyclin-dependent kinase 1 OS=Homo sapiens OX=9606 GN=CDK1 PE=1 SV=3 | 2213375 |
| DNA-directed RNA polymerase III subunit RPC4 OS=Homo sapiens OX=9606 GN=POLR3D PE=1 SV=2 | 447818.1 |
| 60S acidic ribosomal protein P0 OS=Homo sapiens OX=9606 GN=RPLP0 PE=1 SV=1 | 2.3E+08 |
| 60S acidic ribosomal protein P2 OS=Homo sapiens OX=9606 GN=RPLP2 PE=1 SV=1 | 88802400 |
| 60S acidic ribosomal protein P1 OS=Homo sapiens OX=9606 GN=RPLP1 PE=1 SV=1 | 24637077 |
| Eukaryotic translation initiation factor 2 subunit 1 OS=Homo sapiens OX=9606 GN=EIF2S1 PE=1 SV=3 | 58353739 |
| ADP/ATP translocase 2 OS=Homo sapiens OX=9606 GN=SLC25A5 PE=1 SV=7 | 2.96E+08 |
| Protein S100-A8 OS=Homo sapiens OX=9606 GN=S100A8 PE=1 SV=1 | 2315894 |
| Keratin, type II cytoskeletal 6B OS=Homo sapiens OX=9606 GN=KRT6B PE=1 SV=5 | 8152805 |
| Keratin, type II cytoskeletal 6A OS=Homo sapiens OX=9606 GN=KRT6A PE=1 SV=3 | 65309162 |
| Keratin, type I cytoskeletal 14 OS=Homo sapiens OX=9606 GN=KRT14 PE=1 SV=4 | 2.56E+08 |
| Cystatin-A OS=Homo sapiens OX=9606 GN=CSTA PE=1 SV=1 | 1518350 |
| Complement C3 OS=Homo sapiens OX=9606 GN=C3 PE=1 SV=2 | 913276.6 |
| Glutathione reductase, mitochondrial OS=Homo sapiens OX=9606 GN=GSR PE=1 SV=2 |  |
| Serine/threonine-protein kinase PAK 4 OS=Homo sapiens OX=9606 GN=PAK4 PE=1 SV=1 | 1334632 |
| Methyl-CpG-binding domain protein 3 OS=Homo sapiens OX=9606 GN=MBD3 PE=1 SV=1 | 5578340 |
| Apoptosis-inducing factor 1, mitochondrial OS=Homo sapiens OX=9606 GN=AIFM1 PE=1 SV=1 | 3704174 |
| Double-stranded RNA-binding protein Staufen homolog 1 OS=Homo sapiens OX=9606 GN=STAU1 PE=1 SV=2 | 23053403 |
| AP-2 complex subunit alpha-1 OS=Homo sapiens OX=9606 GN=AP2A1 PE=1 SV=3 | 8152345 |
| Cleavage and polyadenylation specificity factor subunit 4 OS=Homo sapiens OX=9606 GN=CPSF4 PE=1 SV=1 | 25267087 |
| Chromobox protein homolog 6 OS=Homo sapiens OX=9606 GN=CBX6 PE=1 SV=1 | 1523081 |
| Sphingosine-1-phosphate lyase 1 OS=Homo sapiens OX=9606 GN=SGPL1 PE=1 SV=3 | 3593851 |
| Myotubularin-related protein 5 OS=Homo sapiens OX=9606 GN=SBF1 PE=1 SV=4 | 5205055 |
| Kinesin-like protein KIF20A OS=Homo sapiens OX=9606 GN=KIF20A PE=1 SV=1 |  |
| Mitochondrial proton/calcium exchanger protein OS=Homo sapiens OX=9606 GN=LETM1 PE=1 SV=1 | 3528548 |
| NADH dehydrogenase [ubiquinone] 1 beta subcomplex subunit 4 OS=Homo sapiens OX=9606 GN=NDUFB4 PE=1 SV=3 | 1220867 |
| Ribonuclease P protein subunit p14 OS=Homo sapiens OX=9606 GN=RPP14 PE=1 SV=3 | 508000 |
| Lysine-specific demethylase 4B OS=Homo sapiens OX=9606 GN=KDM4B PE=1 SV=4 | 501868.6 |
| Pre-mRNA cleavage complex 2 protein Pcf11 OS=Homo sapiens OX=9606 GN=PCF11 PE=1 SV=3 | 10397107 |
| Pre-mRNA-processing factor 6 OS=Homo sapiens OX=9606 GN=PRPF6 PE=1 SV=1 | 33573979 |
| FERM, ARHGEF and pleckstrin domain-containing protein 2 OS=Homo sapiens OX=9606 GN=FARP2 PE=1 SV=3 | 2805436 |
| E3 UFM1-protein ligase 1 OS=Homo sapiens OX=9606 GN=UFL1 PE=1 SV=2 | 6600418 |
| TOX high mobility group box family member 4 OS=Homo sapiens OX=9606 GN=TOX4 PE=1 SV=1 | 13448423 |
| Metastasis-associated protein MTA2 OS=Homo sapiens OX=9606 GN=MTA2 PE=1 SV=1 | 17564311 |
| Signal recognition particle subunit SRP72 OS=Homo sapiens OX=9606 GN=SRP72 PE=1 SV=3 | 37512099 |
| Ribosomal L1 domain-containing protein 1 OS=Homo sapiens OX=9606 GN=RSL1D1 PE=1 SV=3 | 5334519 |
| Survival of motor neuron-related-splicing factor 30 OS=Homo sapiens OX=9606 GN=SMNDC1 PE=1 SV=1 | 12955735 |
| Pre-mRNA-splicing factor SPF27 OS=Homo sapiens OX=9606 GN=BCAS2 PE=1 SV=1 | 11232944 |
| Cyclin-K OS=Homo sapiens OX=9606 GN=CCNK PE=1 SV=2 | 11247472 |
| Ribonuclease P protein subunit p20 OS=Homo sapiens OX=9606 GN=POP7 PE=1 SV=2 | 2770238 |
| U5 small nuclear ribonucleoprotein 200 kDa helicase OS=Homo sapiens OX=9606 GN=SNRNP200 PE=1 SV=2 | 1.05E+08 |
| Cold shock domain-containing protein E1 OS=Homo sapiens OX=9606 GN=CSDE1 PE=1 SV=2 | 3651930 |
| Splicing factor 3B subunit 1 OS=Homo sapiens OX=9606 GN=SF3B1 PE=1 SV=3 | 39851683 |
| TAF5-like RNA polymerase II p300/CBP-associated factor-associated factor 65 kDa subunit 5L OS=Homo sapiens OX=9606 GN=TAF5L PE=1 SV=1 | 1274323 |
| KH domain-containing, RNA-binding, signal transduction-associated protein 3 OS=Homo sapiens OX=9606 GN=KHDRBS3 PE=1 SV=1 | 4000433 |
| PC4 and SFRS1-interacting protein OS=Homo sapiens OX=9606 GN=PSIP1 PE=1 SV=1 | 5407376 |
| Katanin p60 ATPase-containing subunit A1 OS=Homo sapiens OX=9606 GN=KATNA1 PE=1 SV=1 | 12654250 |
| Pre-mRNA-processing factor 40 homolog A OS=Homo sapiens OX=9606 GN=PRPF40A PE=1 SV=2 | 13643957 |
| Hyaluronan mediated motility receptor OS=Homo sapiens OX=9606 GN=HMMR PE=1 SV=2 | 11774000 |
| Gamma-glutamylcyclotransferase OS=Homo sapiens OX=9606 GN=GGCT PE=1 SV=1 | 932060.5 |
| Lysine-specific demethylase PHF2 OS=Homo sapiens OX=9606 GN=PHF2 PE=1 SV=4 | 338933.6 |
| DnaJ homolog subfamily A member 2 OS=Homo sapiens OX=9606 GN=DNAJA2 PE=1 SV=1 | 5990955 |
| DNA/RNA-binding protein KIN17 OS=Homo sapiens OX=9606 GN=KIN PE=1 SV=2 | 7309358 |
| Eukaryotic translation initiation factor 5B OS=Homo sapiens OX=9606 GN=EIF5B PE=1 SV=4 | 1.65E+08 |
| H/ACA ribonucleoprotein complex subunit DKC1 OS=Homo sapiens OX=9606 GN=DKC1 PE=1 SV=3 | 65896244 |
| PRA1 family protein 2 OS=Homo sapiens OX=9606 GN=PRAF2 PE=1 SV=1 | 452276.3 |
| Protein arginine N-methyltransferase 3 OS=Homo sapiens OX=9606 GN=PRMT3 PE=1 SV=4 | 89241786 |
| Ran-binding protein 6 OS=Homo sapiens OX=9606 GN=RANBP6 PE=1 SV=2 | 616523.8 |
| Zinc finger C3H1 domain-containing protein OS=Homo sapiens OX=9606 GN=ZFC3H1 PE=1 SV=3 | 1924616 |
| SWI/SNF-related matrix-associated actin-dependent regulator of chromatin subfamily A member 5 OS=Homo sapiens OX=9606 GN=SMARCA5 PE=1 SV=1 | 26456929 |
| Kinesin-like protein KIF1C OS=Homo sapiens OX=9606 GN=KIF1C PE=1 SV=3 | 7486475 |
| Putative GTP-binding protein 6 OS=Homo sapiens OX=9606 GN=GTPBP6 PE=1 SV=4 | 8874139 |
| U3 small nucleolar RNA-interacting protein 2 OS=Homo sapiens OX=9606 GN=RRP9 PE=1 SV=1 | 551368 |
| Mitotic checkpoint protein BUB3 OS=Homo sapiens OX=9606 GN=BUB3 PE=1 SV=1 | 30341520 |
| Pleiotropic regulator 1 OS=Homo sapiens OX=9606 GN=PLRG1 PE=1 SV=1 | 40295712 |
| Density-regulated protein OS=Homo sapiens OX=9606 GN=DENR PE=1 SV=2 | 4692040 |
| Eukaryotic translation initiation factor 4 gamma 3 OS=Homo sapiens OX=9606 GN=EIF4G3 PE=1 SV=2 | 420112.5 |
| Heterogeneous nuclear ribonucleoprotein R OS=Homo sapiens OX=9606 GN=HNRNPR PE=1 SV=1 | 4.06E+08 |
| RNA-binding protein Musashi homolog 1 OS=Homo sapiens OX=9606 GN=MSI1 PE=1 SV=1 | 518032.5 |
| U4/U6.U5 tri-snRNP-associated protein 1 OS=Homo sapiens OX=9606 GN=SART1 PE=1 SV=1 | 1.33E+08 |
| D-3-phosphoglycerate dehydrogenase OS=Homo sapiens OX=9606 GN=PHGDH PE=1 SV=4 | 2238612 |
| Pre-mRNA-splicing factor ATP-dependent RNA helicase DHX15 OS=Homo sapiens OX=9606 GN=DHX15 PE=1 SV=2 | 70924364 |
| Nuclear valosin-containing protein-like OS=Homo sapiens OX=9606 GN=NVL PE=1 SV=1 | 2044270 |
| Eukaryotic translation initiation factor 3 subunit D OS=Homo sapiens OX=9606 GN=EIF3D PE=1 SV=1 | 23917739 |
| 28S ribosomal protein S12, mitochondrial OS=Homo sapiens OX=9606 GN=MRPS12 PE=1 SV=1 | 11165740 |
| NF-kappa-B-repressing factor OS=Homo sapiens OX=9606 GN=NKRF PE=1 SV=2 | 15859513 |
| U2 snRNP-associated SURP motif-containing protein OS=Homo sapiens OX=9606 GN=U2SURP PE=1 SV=2 | 12719666 |
| Heterogeneous nuclear ribonucleoprotein D-like OS=Homo sapiens OX=9606 GN=HNRNPDL PE=1 SV=3 | 6699996 |
| Protein phosphatase 1 regulatory subunit 12A OS=Homo sapiens OX=9606 GN=PPP1R12A PE=1 SV=1 | 19723066 |
| Aurora kinase A OS=Homo sapiens OX=9606 GN=AURKA PE=1 SV=2 | 600300.7 |
| Cytochrome b-c1 complex subunit 8 OS=Homo sapiens OX=9606 GN=UQCRQ PE=1 SV=4 | 8567615 |
| Mitochondrial import inner membrane translocase subunit Tim23 OS=Homo sapiens OX=9606 GN=TIMM23 PE=1 SV=1 | 994043.8 |
| Insulin receptor substrate 4 OS=Homo sapiens OX=9606 GN=IRS4 PE=1 SV=1 | 1.06E+08 |
| Chromodomain-helicase-DNA-binding protein 2 OS=Homo sapiens OX=9606 GN=CHD2 PE=1 SV=2 | 8085563 |
| AP-3 complex subunit delta-1 OS=Homo sapiens OX=9606 GN=AP3D1 PE=1 SV=1 | 20801365 |
| ATP-dependent RNA helicase DDX3X OS=Homo sapiens OX=9606 GN=DDX3X PE=1 SV=3 | 6.64E+08 |
| Importin subunit alpha-4 OS=Homo sapiens OX=9606 GN=KPNA3 PE=1 SV=2 | 3475204 |
| Insulin-like growth factor 2 mRNA-binding protein 3 OS=Homo sapiens OX=9606 GN=IGF2BP3 PE=1 SV=2 | 30169564 |
| Histone deacetylase complex subunit SAP18 OS=Homo sapiens OX=9606 GN=SAP18 PE=1 SV=1 | 7538364 |
| p40 OS=Homo sapiens OX=9606 PE=4 SV=1 | 1283501 |
| Heterogeneous nuclear ribonucleoprotein U-like protein 1 (Fragment) OS=Homo sapiens OX=9606 GN=HNRNPUL1 PE=1 SV=1 | 586684.9 |
| E3 ubiquitin-protein ligase NOSIP (Fragment) OS=Homo sapiens OX=9606 GN=NOSIP PE=1 SV=8 | 807271.4 |
| 40S ribosomal protein S16 OS=Homo sapiens OX=9606 GN=RPS16 PE=1 SV=1 | 4.87E+08 |
| N-acetyltransferase 14 (Fragment) OS=Homo sapiens OX=9606 GN=NAT14 PE=1 SV=1 | 741360.3 |
| 40S ribosomal protein S5 OS=Homo sapiens OX=9606 GN=RPS5 PE=1 SV=1 | 2.09E+08 |
| Unconventional myosin-IXb OS=Homo sapiens OX=9606 GN=MYO9B PE=1 SV=1 | 3736820 |
| Exosome complex component RRP46 OS=Homo sapiens OX=9606 GN=EXOSC5 PE=1 SV=1 | 1010599 |
| Acid phosphatase type 7 (Fragment) OS=Homo sapiens OX=9606 GN=ACP7 PE=1 SV=1 | 38209972 |
| Splicing factor U2AF 65 kDa subunit (Fragment) OS=Homo sapiens OX=9606 GN=U2AF2 PE=4 SV=1 | 10724575 |
| Zinc finger CCCH domain-containing protein 4 (Fragment) OS=Homo sapiens OX=9606 GN=ZC3H4 PE=1 SV=1 | 67011069 |
| Alternative protein FUNDC2 OS=Homo sapiens OX=9606 GN=FUNDC2 PE=4 SV=1 | 2010019 |
| Telomerase RNA component-interacting RNase OS=Homo sapiens OX=9606 GN=TRIR PE=1 SV=1 | 2272117 |
| Glutathione peroxidase (Fragment) OS=Homo sapiens OX=9606 GN=GPX4 PE=1 SV=1 | 9899943 |
| Keratin, type I cytoskeletal 13 OS=Homo sapiens OX=9606 GN=KRT13 PE=1 SV=1 | 856781.8 |
| Eukaryotic translation initiation factor 3 subunit G (Fragment) OS=Homo sapiens OX=9606 GN=EIF3G PE=1 SV=1 | 340165.3 |
| Phenylalanine--tRNA ligase OS=Homo sapiens OX=9606 GN=FARSA PE=1 SV=1 | 1.23E+08 |
| 40S ribosomal protein S15 OS=Homo sapiens OX=9606 GN=RPS15 PE=1 SV=2 | 1.44E+08 |
| Ubiquitin-conjugating enzyme E2 S (Fragment) OS=Homo sapiens OX=9606 GN=UBE2S PE=1 SV=1 | 845890.3 |
| Cactin (Fragment) OS=Homo sapiens OX=9606 GN=CACTIN PE=1 SV=1 | 1174668 |
| 60S ribosomal protein L22 (Fragment) OS=Homo sapiens OX=9606 GN=RPL22 PE=1 SV=1 | 1.35E+08 |
| Protein LSM12 homolog OS=Homo sapiens OX=9606 GN=LSM12 PE=1 SV=1 | 41123095 |
| 39S ribosomal protein L4, mitochondrial (Fragment) OS=Homo sapiens OX=9606 GN=MRPL4 PE=1 SV=1 | 1805325 |
| 60S ribosomal protein L27 (Fragment) OS=Homo sapiens OX=9606 GN=RPL27 PE=1 SV=1 | 69490450 |
| Eukaryotic translation initiation factor 3 subunit G (Fragment) OS=Homo sapiens OX=9606 GN=EIF3G PE=1 SV=8 | 37965857 |
| Beta-arrestin-2 (Fragment) OS=Homo sapiens OX=9606 GN=ARRB2 PE=1 SV=1 | 1089766 |
| Periplakin OS=Homo sapiens OX=9606 GN=PPL PE=1 SV=1 | 8947000 |
| 60S ribosomal protein L23a (Fragment) OS=Homo sapiens OX=9606 GN=RPL23A PE=1 SV=1 | 3.62E+09 |
| p14ARF/FAM230A fusion protein transcript variant 1 OS=Homo sapiens OX=9606 PE=2 SV=2 | 3361875 |
| Eukaryotic translation initiation factor 3 subunit A OS=Homo sapiens OX=9606 GN=eIF3a PE=2 SV=1 | 1.3E+08 |
| Uncharacterized protein (Fragment) OS=Homo sapiens OX=9606 PE=4 SV=1 | 4223348 |
| ERA-like protein 1 (Fragment) OS=Homo sapiens OX=9606 GN=ERAL1 PE=1 SV=2 | 493847 |
| Proline-rich protein 11 (Fragment) OS=Homo sapiens OX=9606 GN=PRR11 PE=1 SV=1 | 1856703 |
| YTH domain-containing protein 1 OS=Homo sapiens OX=9606 GN=YTHDC1 PE=1 SV=1 | 1534837 |
| 28S ribosomal protein S23, mitochondrial OS=Homo sapiens OX=9606 GN=MRPS23 PE=1 SV=1 | 5255500 |
| SAP30-binding protein (Fragment) OS=Homo sapiens OX=9606 GN=SAP30BP PE=1 SV=1 | 4365394 |
| Serine/arginine-rich splicing factor 1 OS=Homo sapiens OX=9606 GN=SRSF1 PE=1 SV=1 | 1.68E+08 |
| Rotatin OS=Homo sapiens OX=9606 GN=RTTN PE=1 SV=1 | 1583477 |
| DEAD box protein 5 OS=Homo sapiens OX=9606 GN=DDX5 PE=1 SV=1 | 8.73E+08 |
| Mannose-P-dolichol utilization defect 1 protein (Fragment) OS=Homo sapiens OX=9606 GN=MPDU1 PE=1 SV=1 | 310955.6 |
| Receptor-type tyrosine-protein phosphatase mu (Fragment) OS=Homo sapiens OX=9606 GN=PTPRM PE=1 SV=1 | 3246744 |
| 28S ribosomal protein S7, mitochondrial (Fragment) OS=Homo sapiens OX=9606 GN=MRPS7 PE=1 SV=1 | 1167771 |
| Chromobox protein homolog 1 (Fragment) OS=Homo sapiens OX=9606 GN=CBX1 PE=1 SV=8 | 2159528 |
| 60S ribosomal protein L17 (Fragment) OS=Homo sapiens OX=9606 GN=RPL17 PE=1 SV=1 | 4.51E+08 |
| 60S ribosomal protein L36a OS=Homo sapiens OX=9606 GN=RPL36A PE=1 SV=1 | 6.15E+08 |
| BRCA1-associated protein OS=Homo sapiens OX=9606 GN=BRAP PE=1 SV=1 | 1676596 |
| Family with sequence similarity 98, member A OS=Homo sapiens OX=9606 GN=FAM98A PE=2 SV=1 | 1.41E+08 |
| Fatty acid-binding protein 5 OS=Homo sapiens OX=9606 GN=FABP5 PE=1 SV=1 | 1454264 |
| Interleukin-4 receptor subunit alpha (Fragment) OS=Homo sapiens OX=9606 GN=IL4R PE=1 SV=1 | 2.04E+08 |
| E3 SUMO-protein ligase CBX4 (Fragment) OS=Homo sapiens OX=9606 GN=CBX4 PE=1 SV=1 | 1544237 |
| rRNA methyltransferase 3, mitochondrial (Fragment) OS=Homo sapiens OX=9606 GN=MRM3 PE=1 SV=1 | 2545196 |
| 40S ribosomal protein S15a OS=Homo sapiens OX=9606 GN=RPS15A PE=1 SV=1 | 2.99E+08 |
| Eukaryotic translation initiation factor 5A (Fragment) OS=Homo sapiens OX=9606 GN=EIF5A PE=1 SV=8 | 24091299 |
| Myb-binding protein 1A (Fragment) OS=Homo sapiens OX=9606 GN=MYBBP1A PE=1 SV=1 | 24201721 |
| MAPK-regulated corepressor-interacting protein 2 OS=Homo sapiens OX=9606 GN=MCRIP2 PE=1 SV=1 | 2626488 |
| Truncated profilaggrin OS=Homo sapiens OX=9606 GN=FLG PE=4 SV=1 | 2081817 |
| 28S ribosomal protein S28, mitochondrial (Fragment) OS=Homo sapiens OX=9606 GN=MRPS28 PE=1 SV=8 | 1080448 |
| Helicase-like transcription factor (Fragment) OS=Homo sapiens OX=9606 GN=HLTF PE=1 SV=1 | 1121196 |
| Follistatin-related protein 1 (Fragment) OS=Homo sapiens OX=9606 GN=FSTL1 PE=1 SV=1 | 623356.9 |
| Peroxiredoxin-4 (Fragment) OS=Homo sapiens OX=9606 GN=PRDX4 PE=1 SV=1 | 3346792 |
| Mediator of RNA polymerase II transcription subunit 14 (Fragment) OS=Homo sapiens OX=9606 GN=MED14 PE=1 SV=1 | 563336 |
| Non-POU domain-containing octamer-binding protein (Fragment) OS=Homo sapiens OX=9606 GN=NONO PE=1 SV=8 | 763482.4 |
| 60S ribosomal protein L31 (Fragment) OS=Homo sapiens OX=9606 GN=RPL31 PE=1 SV=1 | 3.17E+08 |
| Nucleolar protein 7 (Fragment) OS=Homo sapiens OX=9606 GN=NOL7 PE=1 SV=1 |  |
| ATPase MORC2 (Fragment) OS=Homo sapiens OX=9606 GN=MORC2 PE=1 SV=1 | 346260.9 |
| Ankyrin repeat and SAM domain-containing protein 6 (Fragment) OS=Homo sapiens OX=9606 GN=ANKS6 PE=1 SV=1 | 355750.4 |
| Protein piccolo (Fragment) OS=Homo sapiens OX=9606 GN=PCLO PE=1 SV=1 | 18872324 |
| RNA-binding protein EWS (Fragment) OS=Homo sapiens OX=9606 GN=EWSR1 PE=1 SV=1 | 640102.7 |
| RNA helicase OS=Homo sapiens OX=9606 GN=DHX30 PE=1 SV=1 | 42585763 |
| Cytokeratin-1 OS=Homo sapiens OX=9606 GN=KRT1 PE=3 SV=1 | 4.57E+09 |
| Hepatocellular carcinoma related protein 2 OS=Homo sapiens OX=9606 PE=2 SV=1 | 28476279 |
| Carnitine O-palmitoyltransferase 1, liver isoform (Fragment) OS=Homo sapiens OX=9606 GN=CPT1A PE=1 SV=1 | 508771.2 |
| Cytochrome b-c1 complex subunit 2, mitochondrial OS=Homo sapiens OX=9606 GN=UQCRC2 PE=1 SV=1 | 1628728 |
| Ataxin-2-like protein OS=Homo sapiens OX=9606 GN=ATXN2L PE=1 SV=1 | 504637.3 |
| E3 ubiquitin-protein ligase CHIP (Fragment) OS=Homo sapiens OX=9606 GN=STUB1 PE=1 SV=2 | 312655.2 |
| Zinc finger protein 768 OS=Homo sapiens OX=9606 GN=ZNF768 PE=1 SV=2 | 4636868 |
| Protein-serine/threonine kinase OS=Homo sapiens OX=9606 GN=BCKDK PE=1 SV=1 | 442282.7 |
| Pyruvate kinase OS=Homo sapiens OX=9606 GN=PKM PE=1 SV=1 | 4110189 |
| Zinc finger protein 785 (Fragment) OS=Homo sapiens OX=9606 GN=ZNF785 PE=1 SV=2 | 525450.4 |
| HCG2044799 OS=Homo sapiens OX=9606 GN=HNRNPUL2-BSCL2 PE=4 SV=1 | 1004172 |
| Endonuclease III-like protein 1 (Fragment) OS=Homo sapiens OX=9606 GN=NTHL1 PE=1 SV=1 | 3147354 |
| Mothers against decapentaplegic homolog 3 (Fragment) OS=Homo sapiens OX=9606 GN=SMAD3 PE=1 SV=1 | 1781407 |
| Casein kinase II subunit alpha' OS=Homo sapiens OX=9606 GN=CSNK2A2 PE=1 SV=2 | 233518.6 |
| RNA-binding protein with serine-rich domain 1 (Fragment) OS=Homo sapiens OX=9606 GN=RNPS1 PE=1 SV=1 | 12164424 |
| SRSF protein kinase 1 (Fragment) OS=Homo sapiens OX=9606 GN=SRPK1 PE=1 SV=1 | 32905406 |
| 40S ribosomal protein S17 OS=Homo sapiens OX=9606 GN=RPS17 PE=1 SV=1 | 1.99E+08 |
| CTD small phosphatase-like protein 2 (Fragment) OS=Homo sapiens OX=9606 GN=CTDSPL2 PE=1 SV=1 | 6553118 |
| AP-3 complex subunit sigma-2 OS=Homo sapiens OX=9606 GN=AP3S2 PE=1 SV=1 | 3077300 |
| Activating signal cointegrator 1 OS=Homo sapiens OX=9606 GN=TRIP4 PE=1 SV=1 | 1801875 |
| 60S ribosomal protein L28 OS=Homo sapiens OX=9606 GN=RPL28 PE=1 SV=1 | 1.88E+08 |
| Translation initiation factor eIF-2B subunit beta (Fragment) OS=Homo sapiens OX=9606 GN=EIF2B2 PE=1 SV=1 | 615757.2 |
| LETM1 domain-containing protein 1 (Fragment) OS=Homo sapiens OX=9606 GN=LETMD1 PE=1 SV=1 | 4775270 |
| BICD family-like cargo adapter 1 (Fragment) OS=Homo sapiens OX=9606 GN=BICDL1 PE=4 SV=1 | 1198216 |
| Keratin, type II cytoskeletal 5 (Fragment) OS=Homo sapiens OX=9606 GN=KRT5 PE=1 SV=1 | 2040869 |
| Arginine/serine-rich coiled-coil protein 2 (Fragment) OS=Homo sapiens OX=9606 GN=RSRC2 PE=1 SV=1 | 2981479 |
| Translation initiation factor eIF-2B subunit alpha (Fragment) OS=Homo sapiens OX=9606 GN=EIF2B1 PE=1 SV=1 | 742411.1 |
| Enoyl-CoA hydratase OS=Homo sapiens OX=9606 GN=HADHA PE=1 SV=2 | 2442057 |
| Protein Atg16l2 (Fragment) OS=Homo sapiens OX=9606 GN=ATG16L2 PE=1 SV=1 | 551680.3 |
| 40S ribosomal protein S3 (Fragment) OS=Homo sapiens OX=9606 GN=RPS3 PE=1 SV=1 | 5199489 |
| 40S ribosomal protein S3 (Fragment) OS=Homo sapiens OX=9606 GN=RPS3 PE=1 SV=1 | 14332568 |
| Polyadenylate-binding protein 4 (Fragment) OS=Homo sapiens OX=9606 GN=PABPC4 PE=1 SV=1 | 2346586 |
| Pumilio homolog 1 (Fragment) OS=Homo sapiens OX=9606 GN=PUM1 PE=1 SV=1 | 565131.2 |
| Eukaryotic translation initiation factor 3 subunit F OS=Homo sapiens OX=9606 GN=EIF3F PE=1 SV=2 | 54306972 |
| REST corepressor 3 (Fragment) OS=Homo sapiens OX=9606 GN=RCOR3 PE=1 SV=1 | 629492.1 |
| Gem-associated protein 2 (Fragment) OS=Homo sapiens OX=9606 GN=GEMIN2 PE=1 SV=9 | 3679244 |
| Translation machinery-associated protein 16 (Fragment) OS=Homo sapiens OX=9606 GN=TMA16 PE=1 SV=1 | 52658224 |
| E3 ubiquitin-protein ligase TRIM41 (Fragment) OS=Homo sapiens OX=9606 GN=TRIM41 PE=1 SV=1 | 554971.9 |
| DEK oncogene (DNA binding), isoform CRA_b OS=Homo sapiens OX=9606 GN=DEK PE=1 SV=2 | 15413863 |
| Heterogeneous nuclear ribonucleoprotein D0 (Fragment) OS=Homo sapiens OX=9606 GN=HNRNPD PE=1 SV=8 | 8670384 |
| U3 small nucleolar ribonucleoprotein protein IMP4 (Fragment) OS=Homo sapiens OX=9606 GN=IMP4 PE=1 SV=1 | 2044696 |
| Lethal(3)malignant brain tumor-like protein 2 (Fragment) OS=Homo sapiens OX=9606 GN=L3MBTL2 PE=1 SV=1 | 513187.8 |
| Ubiquitin-associated protein 2-like (Fragment) OS=Homo sapiens OX=9606 GN=UBAP2L PE=1 SV=1 | 1498339 |
| Uncharacterized protein C1orf167 (Fragment) OS=Homo sapiens OX=9606 GN=C1orf167 PE=1 SV=1 | 1165284 |
| Transformation/transcription domain-associated protein OS=Homo sapiens OX=9606 GN=TRRAP PE=1 SV=2 | 5494248 |
| Histone lysine demethylase PHF8 (Fragment) OS=Homo sapiens OX=9606 GN=PHF8 PE=1 SV=1 | 8194449 |
| BUB3-interacting and GLEBS motif-containing protein ZNF207 (Fragment) OS=Homo sapiens OX=9606 GN=ZNF207 PE=1 SV=2 | 6220709 |
| RNA-binding protein 33 (Fragment) OS=Homo sapiens OX=9606 GN=RBM33 PE=1 SV=1 | 22612133 |
| Dolichol-phosphate mannosyltransferase subunit 1 OS=Homo sapiens OX=9606 GN=DPM1 PE=1 SV=2 | 19263134 |
| Netrin receptor DCC (Fragment) OS=Homo sapiens OX=9606 GN=DCC PE=1 SV=2 | 3276899 |
| YWHAE/FAM22B fusion protein (Fragment) OS=Homo sapiens OX=9606 GN=YWHAE/FAM22B fusion PE=2 SV=1 | 8205578 |
| Usher syndrome type-1C protein-binding protein 1 OS=Homo sapiens OX=9606 GN=USHBP1 PE=1 SV=1 | 21535906 |
| SWI/SNF-related matrix-associated actin-dependent regulator of chromatin subfamily B member 1 OS=Homo sapiens OX=9606 GN=SMARCB1 PE=1 SV=1 | 886087.5 |
| Nuclear export mediator factor NEMF (Fragment) OS=Homo sapiens OX=9606 GN=NEMF PE=1 SV=8 | 24462624 |
| Kinectin OS=Homo sapiens OX=9606 GN=KTN1 PE=1 SV=1 | 1275176 |
| Protein transport protein SEC23 OS=Homo sapiens OX=9606 GN=SEC23A PE=1 SV=1 | 387803.3 |
| ATP5MF-PTCD1 readthrough OS=Homo sapiens OX=9606 GN=ATP5MF-PTCD1 PE=4 SV=1 | 9608605 |
| 60S ribosomal protein L18 OS=Homo sapiens OX=9606 GN=RPL18 PE=1 SV=1 | 1.99E+08 |
| Tight junction protein ZO-1 OS=Homo sapiens OX=9606 GN=TJP1 PE=1 SV=2 | 1730747 |
| Cleavage and polyadenylation specificity factor subunit 6 OS=Homo sapiens OX=9606 GN=CPSF6 PE=1 SV=1 | 22312455 |
| Pyruvate dehydrogenase E1 component subunit beta OS=Homo sapiens OX=9606 GN=PDHB PE=1 SV=1 | 485322.8 |
| Asparagine synthetase [glutamine-hydrolyzing] OS=Homo sapiens OX=9606 GN=ASNS PE=1 SV=1 | 953197.7 |
| PHD and RING finger domain-containing protein 1 OS=Homo sapiens OX=9606 GN=PHRF1 PE=1 SV=1 | 8753551 |
| ADP-ribosylation factor-like protein 6-interacting protein 4 OS=Homo sapiens OX=9606 GN=ARL6IP4 PE=1 SV=1 | 1455008 |
| THO complex subunit 5 homolog OS=Homo sapiens OX=9606 GN=THOC5 PE=1 SV=1 | 2019386 |
| Translocation protein SEC62 OS=Homo sapiens OX=9606 GN=SEC62 PE=1 SV=1 | 575155.4 |
| 60S ribosomal protein L10 OS=Homo sapiens OX=9606 GN=RPL10 PE=1 SV=2 | 1.82E+08 |
| Helix-destabilizing protein OS=Homo sapiens OX=9606 GN=HNRNPA1 PE=1 SV=2 | 44355522 |
| Keratin, type II cytoskeletal 74 OS=Homo sapiens OX=9606 GN=KRT74 PE=1 SV=1 | 8374249 |
| Chromosome 17 open reading frame 49 OS=Homo sapiens OX=9606 GN=C17orf49 PE=1 SV=1 | 1103469 |
| Poly(rC)-binding protein 2 OS=Homo sapiens OX=9606 GN=PCBP2 PE=1 SV=1 | 8928205 |
| ADP-ribosylation factor-like protein 1 OS=Homo sapiens OX=9606 GN=ARL1 PE=1 SV=1 | 600827.4 |
| Dynein light chain (Fragment) OS=Homo sapiens OX=9606 GN=DYNLL1 PE=1 SV=1 | 1569090 |
| Nucleosome assembly protein 1-like 1 OS=Homo sapiens OX=9606 GN=NAP1L1 PE=1 SV=1 | 8712127 |
| Periphilin-1 OS=Homo sapiens OX=9606 GN=PPHLN1 PE=1 SV=1 | 1254245 |
| Protein CUSTOS OS=Homo sapiens OX=9606 GN=C12orf43 PE=1 SV=2 | 2193579 |
| Tubulin alpha chain OS=Homo sapiens OX=9606 GN=TUBA1C PE=1 SV=1 | 40154728 |
| Mediator complex subunit 24 OS=Homo sapiens OX=9606 GN=MED24 PE=1 SV=2 | 884097.9 |
| Prohibitin OS=Homo sapiens OX=9606 GN=PHB2 PE=1 SV=1 | 1842394 |
| NF110b OS=Homo sapiens OX=9606 PE=2 SV=1 | 1.5E+08 |
| U6 snRNA-associated Sm-like protein LSm8 OS=Homo sapiens OX=9606 GN=LSM8 PE=1 SV=1 | 4734368 |
| SOGA OS=Homo sapiens OX=9606 GN=SOGA PE=2 SV=1 | 253779.1 |
| 40S ribosomal protein S3 OS=Homo sapiens OX=9606 GN=RPS3 PE=1 SV=1 | 762843.6 |
| Uncharacterized protein C11orf98 OS=Homo sapiens OX=9606 GN=C11orf98 PE=4 SV=2 | 35772965 |
| Cofilin, non-muscle isoform (Fragment) OS=Homo sapiens OX=9606 GN=CFL1 PE=1 SV=1 | 1584933 |
| Rae1 protein homolog OS=Homo sapiens OX=9606 GN=RAE1 PE=1 SV=1 | 415231.9 |
| Tyrosine-protein kinase OS=Homo sapiens OX=9606 GN=TYK2 PE=1 SV=1 | 772688.3 |
| Microtubule-actin cross-linking factor 1, isoforms 1/2/3/5 (Fragment) OS=Homo sapiens OX=9606 GN=MACF1 PE=1 SV=1 | 383352.3 |
| Telomerase Cajal body protein 1 OS=Homo sapiens OX=9606 GN=WRAP53 PE=1 SV=1 | 689512.9 |
| Chloride channel, nucleotide sensitive 1A OS=Homo sapiens OX=9606 GN=CLNS1A PE=1 SV=1 |  |
| VKc domain-containing protein (Fragment) OS=Homo sapiens OX=9606 PE=4 SV=1 | 608346.8 |
| Coiled-coil domain-containing protein 90B, mitochondrial OS=Homo sapiens OX=9606 GN=CCDC90B PE=1 SV=1 | 2738262 |
| Pleckstrin homology domain-containing family A member 7 OS=Homo sapiens OX=9606 GN=PLEKHA7 PE=1 SV=1 | 909369.2 |
| Bcl-2-associated transcription factor 1 OS=Homo sapiens OX=9606 GN=BCLAF1 PE=1 SV=1 | 5.57E+08 |
| Mitochondrial glutamate carrier 1 (Fragment) OS=Homo sapiens OX=9606 GN=SLC25A22 PE=1 SV=5 | 1446455 |
| Protein piccolo OS=Homo sapiens OX=9606 GN=PCLO PE=1 SV=1 | 13349269 |
| 28S ribosomal protein S17, mitochondrial (Fragment) OS=Homo sapiens OX=9606 GN=MRPS17 PE=1 SV=1 | 3120148 |
| Ankyrin repeat and KH domain-containing protein 1 (Fragment) OS=Homo sapiens OX=9606 GN=ANKHD1 PE=1 SV=1 | 2301335 |
| Unconventional myosin-Ib OS=Homo sapiens OX=9606 GN=MYO1B PE=1 SV=1 | 7089553 |
| Heterogeneous nuclear ribonucleoprotein H OS=Homo sapiens OX=9606 GN=HNRNPH1 PE=1 SV=1 | 1.42E+08 |
| Slit homolog 2 protein (Fragment) OS=Homo sapiens OX=9606 GN=SLIT2 PE=1 SV=1 |  |
| G2/mitotic-specific cyclin-B1 (Fragment) OS=Homo sapiens OX=9606 GN=CCNB1 PE=1 SV=1 | 1976666 |
| Histone-binding protein RBBP7 OS=Homo sapiens OX=9606 GN=RBBP7 PE=1 SV=1 | 5022999 |
| Acylglycerol kinase, mitochondrial OS=Homo sapiens OX=9606 GN=AGK PE=1 SV=1 | 3950216 |
| THO complex subunit 4 OS=Homo sapiens OX=9606 GN=ALYREF PE=1 SV=1 | 1.19E+09 |
| Eukaryotic translation initiation factor 4 gamma 1 OS=Homo sapiens OX=9606 GN=EIF4G1 PE=1 SV=1 | 1.24E+08 |
| ATP-dependent DNA/RNA helicase DHX36 (Fragment) OS=Homo sapiens OX=9606 GN=DHX36 PE=1 SV=1 | 4243738 |
| Ras GTPase-activating-like protein IQGAP2 (Fragment) OS=Homo sapiens OX=9606 GN=IQGAP2 PE=1 SV=1 | 3736894 |
| CLIP-associating protein 2 OS=Homo sapiens OX=9606 GN=CLASP2 PE=1 SV=1 | 1671734 |
| Glyceraldehyde-3-phosphate dehydrogenase OS=Homo sapiens OX=9606 GN=GAPDH PE=1 SV=1 | 8369435 |
| Non-specific serine/threonine protein kinase OS=Homo sapiens OX=9606 GN=MARK2 PE=1 SV=2 | 11736296 |
| Treacle protein OS=Homo sapiens OX=9606 GN=TCOF1 PE=1 SV=1 | 52551020 |
| Metastasis-associated protein MTA1 OS=Homo sapiens OX=9606 GN=MTA1 PE=1 SV=1 | 9084632 |
| Translation initiation factor eIF-2B subunit delta OS=Homo sapiens OX=9606 GN=EIF2B4 PE=1 SV=1 | 2848402 |
| CCT-alpha OS=Homo sapiens OX=9606 GN=TCP1 PE=1 SV=1 | 587787.1 |
| Protein FAM91A1 OS=Homo sapiens OX=9606 GN=FAM91A1 PE=1 SV=1 | 535178.9 |
| Alpha-adducin OS=Homo sapiens OX=9606 GN=ADD1 PE=1 SV=1 | 340898.1 |
| Mitogen-activated protein kinase kinase kinase kinase 4 OS=Homo sapiens OX=9606 GN=MAP4K4 PE=1 SV=1 | 841291.4 |
| S-phase kinase-associated protein 1 OS=Homo sapiens OX=9606 GN=SKP1 PE=1 SV=1 | 3196588 |
| 39S ribosomal protein L15, mitochondrial (Fragment) OS=Homo sapiens OX=9606 GN=MRPL15 PE=1 SV=2 | 354452.9 |
| 60S ribosomal protein L30 (Fragment) OS=Homo sapiens OX=9606 GN=RPL30 PE=1 SV=1 | 1.73E+08 |
| Glutamate-rich protein 1 OS=Homo sapiens OX=9606 GN=ERICH1 PE=1 SV=1 | 607839.4 |
| F-box/WD repeat-containing protein 11 OS=Homo sapiens OX=9606 GN=FBXW11 PE=1 SV=9 | 2122114 |
| Mitochondrial transcription factor A OS=Homo sapiens OX=9606 PE=4 SV=1 | 2194776 |
| Plasma membrane citrate carrier OS=Homo sapiens OX=9606 GN=SLC25A1 PE=2 SV=1 | 3108093 |
| DING protein p38 OS=Homo sapiens OX=9606 PE=3 SV=1 | 4337594 |
| Cold-inducible RNA-binding protein OS=Homo sapiens OX=9606 GN=CIRBP PE=1 SV=1 | 10811196 |
| Exosome complex component RRP45 OS=Homo sapiens OX=9606 GN=EXOSC9 PE=1 SV=1 | 538032.6 |
| THO complex subunit 3 OS=Homo sapiens OX=9606 GN=THOC3 PE=1 SV=1 | 4314879 |
| Sideroflexin-1 (Fragment) OS=Homo sapiens OX=9606 GN=SFXN1 PE=1 SV=8 | 1585859 |
| Eukaryotic translation elongation factor 1 epsilon-1 (Fragment) OS=Homo sapiens OX=9606 GN=EEF1E1 PE=1 SV=1 | 723515.8 |
| 60S ribosomal protein L32 (Fragment) OS=Homo sapiens OX=9606 GN=RPL32 PE=1 SV=1 | 3.53E+08 |
| Glyoxylate reductase 1 homolog OS=Homo sapiens OX=9606 GN=N-PAC PE=3 SV=1 |  |
| Formin binding protein 4, isoform CRA_d OS=Homo sapiens OX=9606 GN=FNBP4 PE=4 SV=1 | 8693609 |
| Fibrinogen beta chain OS=Homo sapiens OX=9606 GN=FGB PE=4 SV=1 | 5648643 |
| Catenin delta-1 OS=Homo sapiens OX=9606 GN=CTNND1 PE=1 SV=2 | 810068.4 |
| 60S ribosomal protein L22-like 1 OS=Homo sapiens OX=9606 GN=RPL22L1 PE=1 SV=1 | 1.96E+08 |
| Rab-like protein 3 OS=Homo sapiens OX=9606 GN=RABL3 PE=1 SV=1 | 3237690 |
| Holliday junction recognition protein (Fragment) OS=Homo sapiens OX=9606 GN=HJURP PE=1 SV=1 | 512726.8 |
| F-actin-capping protein subunit alpha OS=Homo sapiens OX=9606 GN=CAPZA2 PE=1 SV=1 | 1247085 |
| Peptidyl-prolyl cis-trans isomerase OS=Homo sapiens OX=9606 GN=PPIH PE=1 SV=2 | 8075965 |
| DNA-directed RNA polymerases I, II, and III subunit RPABC3 (Fragment) OS=Homo sapiens OX=9606 GN=POLR2H PE=1 SV=8 | 6515807 |
| Cathepsin D OS=Homo sapiens OX=9606 GN=CTSD PE=1 SV=2 | 2016251 |
| Eukaryotic translation initiation factor 4E type 2 (Fragment) OS=Homo sapiens OX=9606 GN=EIF4E2 PE=1 SV=1 | 3784616 |
| Protein BUD31 homolog OS=Homo sapiens OX=9606 GN=BUD31 PE=1 SV=1 | 1798965 |
| Signal sequence receptor subunit gamma OS=Homo sapiens OX=9606 GN=SSR3 PE=1 SV=1 | 1055713 |
| Arf-GAP with GTPase, ANK repeat and PH domain-containing protein 3 OS=Homo sapiens OX=9606 GN=AGAP3 PE=1 SV=1 | 5178927 |
| Protein downstream neighbor of Son OS=Homo sapiens OX=9606 GN=DONSON PE=1 SV=1 | 5551402 |
| Signal sequence receptor subunit alpha OS=Homo sapiens OX=9606 GN=SSR1 PE=1 SV=1 | 1287893 |
| Protein CMSS1 (Fragment) OS=Homo sapiens OX=9606 GN=CMSS1 PE=1 SV=1 | 18803839 |
| RNA-binding protein 12B OS=Homo sapiens OX=9606 GN=RBM12B PE=1 SV=2 | 4411769 |
| DNA topoisomerase I OS=Homo sapiens OX=9606 GN=TOP1 PE=2 SV=1 | 51605579 |
| Dual-specificity protein kinase CLK1 OS=Homo sapiens OX=9606 GN=CLK1 PE=1 SV=1 | 7388179 |
| Mediator complex subunit 26 OS=Homo sapiens OX=9606 PE=3 SV=2 | 395811.1 |
| EPC1/ASXL2b fusion protein OS=Homo sapiens OX=9606 PE=2 SV=1 | 308595.4 |
| IBTK protein OS=Homo sapiens OX=9606 GN=IBTK PE=2 SV=1 |  |
| GEMIN5 protein OS=Homo sapiens OX=9606 GN=GEMIN5 PE=2 SV=1 | 1386942 |
| eIF2AK2 protein OS=Homo sapiens OX=9606 GN=EIF2AK2 PE=2 SV=1 | 2284015 |
| Eukaryotic translation initiation factor 6 (Fragment) OS=Homo sapiens OX=9606 GN=EIF6 PE=1 SV=1 | 1455933 |
| cDNA, FLJ79369, highly similar to JmjC domain-containing histone demethylation protein 1B OS=Homo sapiens OX=9606 PE=2 SV=1 | 596234.1 |
| DNA polymerase delta subunit 3 OS=Homo sapiens OX=9606 PE=2 SV=1 | 779795.5 |
| E3 ubiquitin-protein ligase CBL OS=Homo sapiens OX=9606 PE=2 SV=1 | 10096868 |
| RNA helicase OS=Homo sapiens OX=9606 PE=2 SV=1 | 1198992 |
| cDNA FLJ61633, highly similar to Homo sapiens ubiquitin associated protein 2 (UBAP2), transcript variant 1, mRNA OS=Homo sapiens OX=9606 PE=2 SV=1 | 2628156 |
| Myosin light polypeptide 6 OS=Homo sapiens OX=9606 GN=MYL6 PE=1 SV=1 | 5310017 |
| DNA ligase OS=Homo sapiens OX=9606 PE=2 SV=1 | 855759.9 |
| Sec1 family domain-containing protein 1 OS=Homo sapiens OX=9606 GN=SCFD1 PE=1 SV=1 |  |
| Calcium load-activated calcium channel OS=Homo sapiens OX=9606 GN=TMCO1 PE=2 SV=1 | 3465454 |
| 60 kDa chaperonin OS=Homo sapiens OX=9606 PE=2 SV=1 | 12825799 |
| Carbohydrate sulfotransferase OS=Homo sapiens OX=9606 PE=2 SV=1 | 2322585 |
| cDNA FLJ54733, highly similar to General transcription factor 3C polypeptide 5 OS=Homo sapiens OX=9606 PE=2 SV=1 | 3991423 |
| Non-specific serine/threonine protein kinase OS=Homo sapiens OX=9606 PE=2 SV=1 | 3125650 |
| IMP dehydrogenase OS=Homo sapiens OX=9606 PE=2 SV=1 | 3356354 |
| Pre-mRNA-splicing factor CWC22 homolog (Fragment) OS=Homo sapiens OX=9606 GN=CWC22 PE=1 SV=1 | 420472.9 |
| Peroxisome proliferator-activated receptor gamma OS=Homo sapiens OX=9606 GN=NR1C3 PE=2 SV=1 | 826144 |
| GTP-binding nuclear protein Ran OS=Homo sapiens OX=9606 GN=RAN PE=1 SV=1 | 4265420 |
| Pescadillo homolog OS=Homo sapiens OX=9606 GN=PES1 PE=1 SV=1 | 30053749 |
| Sororin OS=Homo sapiens OX=9606 GN=CDCA5 PE=1 SV=1 | 1771342 |
| RuvB-like helicase (Fragment) OS=Homo sapiens OX=9606 GN=RUVBL1 PE=2 SV=1 | 8967269 |
| Shugoshin-like 1 (S. pombe), isoform CRA_a OS=Homo sapiens OX=9606 GN=SGOL1 PE=2 SV=1 | 283530.1 |
| U2 snRNP auxiliary factor large subunit OS=Homo sapiens OX=9606 GN=U2AF2 PE=2 SV=1 | 89524236 |
| cDNA FLJ54293 OS=Homo sapiens OX=9606 PE=2 SV=1 | 5613086 |
| cDNA FLJ56024 OS=Homo sapiens OX=9606 PE=2 SV=1 | 570517.7 |
| cDNA FLJ56606, highly similar to Serine/threonine-protein kinase TAO2 OS=Homo sapiens OX=9606 PE=2 SV=1 | 5058518 |
| cDNA FLJ54081, highly similar to Keratin, type II cytoskeletal 5 OS=Homo sapiens OX=9606 PE=2 SV=1 |  |
| Poly [ADP-ribose] polymerase OS=Homo sapiens OX=9606 PE=2 SV=1 | 55669958 |
| PRP4 pre-mRNA-processing factor 4 homolog OS=Homo sapiens OX=9606 PE=2 SV=1 | 10936674 |
| cDNA FLJ56367 OS=Homo sapiens OX=9606 PE=2 SV=1 | 9475625 |
| cDNA FLJ59571, highly similar to Eukaryotic translation initiation factor 4gamma 2 OS=Homo sapiens OX=9606 PE=2 SV=1 | 24495720 |
| DNA-directed RNA polymerase OS=Homo sapiens OX=9606 PE=2 SV=1 | 2862273 |
| 5'-3' exoribonuclease OS=Homo sapiens OX=9606 PE=2 SV=1 | 14852484 |
| cDNA FLJ58490, highly similar to Homo sapiens CCR4-NOT transcription complex, subunit 1 (CNOT1), transcript variant 1, mRNA OS=Homo sapiens OX=9606 PE=2 SV=1 | 1783344 |
| Interleukin enhancer-binding factor 2 OS=Homo sapiens OX=9606 GN=ILF2 PE=1 SV=1 | 89821503 |
| Heterogeneous nuclear ribonucleoproteins C1/C2 OS=Homo sapiens OX=9606 GN=HNRNPC PE=1 SV=1 | 23833882 |
| Flap endonuclease 1 OS=Homo sapiens OX=9606 GN=FEN1 PE=2 SV=1 | 11528098 |
| cDNA FLJ61181, highly similar to Homo sapiens hydroxysteroid (17-beta) dehydrogenase 12 (HSD17B12), mRNA OS=Homo sapiens OX=9606 PE=2 SV=1 | 8410288 |
| Paraspeckle component 1 OS=Homo sapiens OX=9606 PE=2 SV=1 | 87633933 |
| RNA helicase OS=Homo sapiens OX=9606 PE=2 SV=1 | 9877181 |
| cDNA FLJ56640, highly similar to Mus musculus embryonic ectoderm development (Eed), mRNA OS=Homo sapiens OX=9606 PE=2 SV=1 | 531304.9 |
| Eukaryotic translation initiation factor 3 subunit C OS=Homo sapiens OX=9606 GN=EIF3C PE=2 SV=1 | 1.01E+08 |
| cDNA FLJ58285, highly similar to Homo sapiens pre-B-cell leukemia transcription factor interacting protein 1 (PBXIP1), mRNA OS=Homo sapiens OX=9606 PE=2 SV=1 | 773900.7 |
| Eukaryotic translation initiation factor 3 subunit B OS=Homo sapiens OX=9606 GN=EIF3B PE=2 SV=1 | 54397173 |
| cDNA FLJ58284, moderately similar to Probable transcription factor PML OS=Homo sapiens OX=9606 PE=2 SV=1 | 785209.4 |
| Heterogeneous nuclear ribonucleoprotein K OS=Homo sapiens OX=9606 PE=2 SV=1 | 6.77E+08 |
| cDNA FLJ60152, highly similar to Zinc finger protein 295 OS=Homo sapiens OX=9606 PE=2 SV=1 | 3298489 |
| cDNA FLJ61387, highly similar to Homo sapiens conserved nuclear protein NHN1 (NHN1), mRNA OS=Homo sapiens OX=9606 PE=2 SV=1 | 2162263 |
| Protein SDA1 OS=Homo sapiens OX=9606 PE=2 SV=1 | 8729964 |
| cDNA FLJ61383, highly similar to Serine/threonine-protein kinase 24 OS=Homo sapiens OX=9606 PE=2 SV=1 | 587736.5 |
| cDNA FLJ59402, highly similar to Eukaryotic translation initiation factor 4B OS=Homo sapiens OX=9606 PE=2 SV=1 | 10145357 |
| cDNA FLJ51286, highly similar to WD repeat protein 12 OS=Homo sapiens OX=9606 PE=2 SV=1 | 1928948 |
| cDNA FLJ58459, highly similar to RNA-binding region-containing protein 2 OS=Homo sapiens OX=9606 PE=2 SV=1 | 1.43E+08 |
| cDNA FLJ51519, highly similar to Homo sapiens calcium homeostasis endoplasmic reticulum protein (CHERP), mRNA OS=Homo sapiens OX=9606 PE=2 SV=1 |  |
| cDNA FLJ58778, highly similar to Plasminogen OS=Homo sapiens OX=9606 PE=2 SV=1 | 397395.9 |
| cDNA FLJ60132, highly similar to Zinc finger protein ZIC 2 OS=Homo sapiens OX=9606 PE=2 SV=1 | 486450.3 |
| DnaJ (Hsp40) homolog, subfamily B, member 4, isoform CRA_b OS=Homo sapiens OX=9606 GN=DNAJB4 PE=2 SV=1 | 1964352 |
| AP-2 complex subunit mu OS=Homo sapiens OX=9606 PE=2 SV=1 | 7192737 |
| cDNA FLJ52703, highly similar to Asparaginyl-tRNA synthetase, cytoplasmic (EC6.1.1.22) OS=Homo sapiens OX=9606 PE=2 SV=1 | 942608.7 |
| Brain-specific angiogenesis inhibitor 1-associated protein 2 OS=Homo sapiens OX=9606 PE=2 SV=1 | 1704452 |
| cDNA FLJ58174, highly similar to WW domain-binding protein 11 OS=Homo sapiens OX=9606 PE=2 SV=1 | 3271252 |
| Kinesin-like protein OS=Homo sapiens OX=9606 PE=2 SV=1 | 11638919 |
| 60S ribosomal protein L18a OS=Homo sapiens OX=9606 PE=2 SV=1 | 1.5E+08 |
| cDNA FLJ54020, highly similar to Heterogeneous nuclear ribonucleoprotein U OS=Homo sapiens OX=9606 PE=2 SV=1 |  |
| cDNA FLJ60124, highly similar to Mitochondrial dicarboxylate carrier OS=Homo sapiens OX=9606 PE=2 SV=1 | 4407376 |
| cDNA FLJ58196, highly similar to Zinc finger CCCH domain-containing protein 11A OS=Homo sapiens OX=9606 PE=2 SV=1 | 6394012 |
| Upstream binding transcription factor, RNA polymerase I, isoform CRA_a OS=Homo sapiens OX=9606 GN=UBTF PE=2 SV=1 | 2383026 |
| cDNA FLJ57525, highly similar to Cell division cycle protein 27 homolog OS=Homo sapiens OX=9606 PE=2 SV=1 | 9379572 |
| cDNA FLJ56547, highly similar to Homo sapiens DEAD/H (Asp-Glu-Ala-Asp/His) box polypeptide 26 (DDX26), mRNA OS=Homo sapiens OX=9606 PE=2 SV=1 | 908810.3 |
| RNA helicase OS=Homo sapiens OX=9606 PE=2 SV=1 | 1894012 |
| cDNA FLJ53353, highly similar to ATP-binding cassette sub-family D member 3 OS=Homo sapiens OX=9606 PE=2 SV=1 | 798547.4 |
| Programmed cell death protein 4 OS=Homo sapiens OX=9606 PE=2 SV=1 | 757840.3 |
| Monocarboxylate transporter 1 OS=Homo sapiens OX=9606 PE=2 SV=1 | 13829331 |
| cDNA FLJ58539, highly similar to Keratin, type II cytoskeletal 4 OS=Homo sapiens OX=9606 PE=2 SV=1 | 1335099 |
| cDNA FLJ54799, highly similar to Mitochondrial 28S ribosomal protein S27 OS=Homo sapiens OX=9606 PE=2 SV=1 | 3479415 |
| Optic atrophy 3 protein OS=Homo sapiens OX=9606 GN=OPA3 PE=1 SV=1 | 2883200 |
| Splicing factor 1 OS=Homo sapiens OX=9606 PE=2 SV=1 | 8885728 |
| L-lactate dehydrogenase OS=Homo sapiens OX=9606 PE=2 SV=1 | 962346.1 |
| Interferon-inducible double-stranded RNA-dependent protein kinase activator A OS=Homo sapiens OX=9606 GN=PRKRA PE=1 SV=1 | 1671723 |
| cDNA FLJ50778, highly similar to Protein flightless-1 homolog OS=Homo sapiens OX=9606 PE=2 SV=1 | 493150.1 |
| Nucleolar protein 12 OS=Homo sapiens OX=9606 PE=2 SV=1 | 12682364 |
| Apolipoprotein D OS=Homo sapiens OX=9606 PE=2 SV=1 | 3764018 |
| cDNA FLJ59194, moderately similar to Dynein light chain 2A, cytoplasmic OS=Homo sapiens OX=9606 PE=2 SV=1 | 514435.2 |
| S-adenosylmethionine synthase OS=Homo sapiens OX=9606 GN=MAT2A PE=2 SV=1 |  |
| Mitochondrial ribosomal protein L10, isoform CRA_d OS=Homo sapiens OX=9606 GN=MRPL10 PE=2 SV=1 | 2085622 |
| cDNA FLJ53704, moderately similar to Nascent polypeptide-associated complex subunit alpha OS=Homo sapiens OX=9606 PE=2 SV=1 | 4667056 |
| Cleavage stimulation factor 50 kDa subunit OS=Homo sapiens OX=9606 PE=2 SV=1 | 957685.4 |
| TMEM214 protein (Fragment) OS=Homo sapiens OX=9606 GN=TMEM214 PE=2 SV=1 | 8161977 |
| cDNA FLJ16777 fis, clone BRHIP2029567, highly similar to Cell division cycle 5-like protein OS=Homo sapiens OX=9606 PE=2 SV=1 | 1.48E+08 |
| cDNA FLJ46277 fis, clone TESTI4029731 OS=Homo sapiens OX=9606 PE=2 SV=1 | 12870417 |
| cDNA FLJ45747 fis, clone LIVER2008465 OS=Homo sapiens OX=9606 PE=2 SV=1 | 3135377 |
| cDNA FLJ45019 fis, clone BRAWH3015825 OS=Homo sapiens OX=9606 PE=2 SV=1 | 724241.2 |
| 116 kDa U5 small nuclear ribonucleoprotein component OS=Homo sapiens OX=9606 PE=2 SV=1 | 48243176 |
| Mortality factor 4-like protein 2 OS=Homo sapiens OX=9606 PE=2 SV=1 | 32447017 |
| Histone deacetylase OS=Homo sapiens OX=9606 PE=2 SV=1 | 7846478 |
| cDNA FLJ39996 fis, clone STOMA2002166, highly similar to Splicing factor 3B subunit 4 OS=Homo sapiens OX=9606 PE=2 SV=1 | 2412336 |
| cDNA FLJ39034 fis, clone NT2RP7008085, highly similar to Homo sapiens ring finger protein 123 (RNF123), mRNA OS=Homo sapiens OX=9606 PE=2 SV=1 | 2476102 |
| cDNA FLJ39022 fis, clone NT2RP7003724, weakly similar to Serine/arginine repetitive matrix protein 1 OS=Homo sapiens OX=9606 PE=2 SV=1 | 803441.1 |
| Mortality factor 4-like protein 1 OS=Homo sapiens OX=9606 GN=MORF4L1 PE=1 SV=1 | 586638.2 |
| cDNA FLJ37362 fis, clone BRAMY2024004, highly similar to Splicing factor, arginine/serine-rich 16 OS=Homo sapiens OX=9606 PE=2 SV=1 | 1612738 |
| cDNA FLJ34439 fis, clone HLUNG2001146, highly similar to Splicing factor, arginine/serine-rich 12 OS=Homo sapiens OX=9606 PE=2 SV=1 | 4068879 |
| cDNA FLJ34373 fis, clone FEBRA2017333, highly similar to Dual specificity protein kinase CLK3 OS=Homo sapiens OX=9606 PE=2 SV=1 | 12533633 |
| DNA excision repair protein ERCC-3 OS=Homo sapiens OX=9606 PE=2 SV=1 | 6719196 |
| cDNA FLJ34015 fis, clone FCBBF2002349, highly similar to Homo sapiens DEAH (Asp-Glu-Ala-His) box polypeptide 40 (DHX40), mRNA OS=Homo sapiens OX=9606 PE=2 SV=1 | 1316380 |
| cDNA FLJ90282 fis, clone NT2RP1000551, highly similar to Interferon-related developmental regulator 2 OS=Homo sapiens OX=9606 PE=2 SV=1 | 7191020 |
| cDNA FLJ32982 fis, clone THYMU1000002, highly similar to Golgi phosphoprotein 3 OS=Homo sapiens OX=9606 PE=2 SV=1 | 496914.1 |
| cDNA FLJ32300 fis, clone PROST2002227, highly similar to U3 small nucleolar ribonucleoprotein protein MPP10 (Fragment) OS=Homo sapiens OX=9606 PE=2 SV=1 | 2393026 |
| cDNA FLJ30174 fis, clone BRACE2000975, highly similar to Sodium/potassium-transporting ATPase alpha-3 chain OS=Homo sapiens OX=9606 PE=2 SV=1 | 583889.6 |
| cDNA FLJ14648 fis, clone NT2RP2002046, highly similar to Protein FAM48A OS=Homo sapiens OX=9606 PE=2 SV=1 | 946837.1 |
| Nucleolar complex protein 2 homolog OS=Homo sapiens OX=9606 PE=2 SV=1 | 942291.4 |
| HBS1-like protein OS=Homo sapiens OX=9606 PE=2 SV=1 | 4774194 |
| cDNA FLJ13562 fis, clone PLACE1008080, highly similar to Homo sapiens hexamethylene bis-acetamide inducible 1 (HEXIM1), mRNA OS=Homo sapiens OX=9606 PE=2 SV=1 | 5246790 |
| Anaphase-promoting complex subunit 4 OS=Homo sapiens OX=9606 PE=2 SV=1 | 548074.2 |
| Very-long-chain enoyl-CoA reductase OS=Homo sapiens OX=9606 PE=2 SV=1 | 6887567 |
| cDNA FLJ10529 fis, clone NT2RP2000965, highly similar to Targeting protein for Xklp2 OS=Homo sapiens OX=9606 PE=2 SV=1 | 6717315 |
| Zinc finger RNA binding protein OS=Homo sapiens OX=9606 GN=ZFR PE=2 SV=1 | 15250988 |
| Cytoskeleton associated protein 2 OS=Homo sapiens OX=9606 GN=CKAP2 PE=2 SV=1 | 15424805 |
| Phosphate carrier protein, mitochondrial OS=Homo sapiens OX=9606 PE=2 SV=1 | 27707838 |
| cDNA, FLJ96811, highly similar to Homo sapiens G patch domain containing 1 (GPATC1), mRNA OS=Homo sapiens OX=9606 PE=2 SV=1 | 653143.1 |
| cDNA, FLJ96764, highly similar to Homo sapiens sorting nexin 8 (SNX8), mRNA OS=Homo sapiens OX=9606 PE=2 SV=1 | 451332.2 |
| cDNA, FLJ96580, highly similar to Homo sapiens hepatoma-derived growth factor (high-mobility group protein 1-like) (HDGF), mRNA OS=Homo sapiens OX=9606 PE=2 SV=1 | 2583296 |
| Protein arginine N-methyltransferase 5 OS=Homo sapiens OX=9606 PE=2 SV=1 | 1.66E+08 |
| cDNA, FLJ96442, highly similar to Homo sapiens copine II (CPNE2), mRNA OS=Homo sapiens OX=9606 PE=2 SV=1 | 1344388 |
| cDNA, FLJ96406, highly similar to Homo sapiens NOL1/NOP2/Sun domain family, member 5 (NSUN5), transcript variant 1, mRNA OS=Homo sapiens OX=9606 PE=2 SV=1 | 13524900 |
| cDNA, FLJ96114, highly similar to Homo sapiens bromodomain and WD repeat domain containing 2 (BRWD2), mRNA OS=Homo sapiens OX=9606 PE=2 SV=1 | 1712785 |
| Aurora kinase OS=Homo sapiens OX=9606 PE=2 SV=1 | 3074706 |
| cDNA, FLJ95666, highly similar to Homo sapiens albumin (ALB), mRNA OS=Homo sapiens OX=9606 PE=2 SV=1 | 1.6E+08 |
| cDNA, FLJ94450, highly similar to Homo sapiens cyclin-dependent kinase 9 (CDC2-related kinase) (CDK9), mRNA OS=Homo sapiens OX=9606 PE=2 SV=1 | 7116424 |
| cDNA, FLJ94440, highly similar to Homo sapiens chaperonin containing TCP1, subunit 6A (zeta 1)(CCT6A), mRNA OS=Homo sapiens OX=9606 PE=2 SV=1 | 486477.5 |
| cDNA, FLJ94229, highly similar to Homo sapiens heterogeneous nuclear ribonucleoprotein L (HNRPL),mRNA OS=Homo sapiens OX=9606 PE=2 SV=1 | 76035961 |
| cDNA, FLJ94136, highly similar to Homo sapiens synaptotagmin binding, cytoplasmic RNA interacting protein (SYNCRIP), mRNA OS=Homo sapiens OX=9606 PE=2 SV=1 | 5.13E+08 |
| cDNA, FLJ94025, highly similar to Homo sapiens tripartite motif-containing 28 (TRIM28), mRNA OS=Homo sapiens OX=9606 PE=2 SV=1 | 10171738 |
| cDNA, FLJ93871, highly similar to Homo sapiens melanoma antigen, family B, 2 (MAGEB2), mRNA OS=Homo sapiens OX=9606 PE=2 SV=1 | 23444192 |
| cDNA, FLJ93744, highly similar to Homo sapiens keratin 6E (KRT6E), mRNA OS=Homo sapiens OX=9606 PE=2 SV=1 | 2409666 |
| Serine/threonine-protein kinase PLK OS=Homo sapiens OX=9606 PE=2 SV=1 | 2315659 |
| RNA-binding protein PNO1 OS=Homo sapiens OX=9606 PE=2 SV=1 | 33883923 |
| cDNA, FLJ93619, highly similar to Homo sapiens PRP4 pre-mRNA processing factor 4 homolog (yeast) (PRPF4), mRNA OS=Homo sapiens OX=9606 PE=2 SV=1 | 28948511 |
| cDNA, FLJ93435, highly similar to Homo sapiens general transcription factor IIH, polypeptide 1 (62kD subunit) (GTF2H1), mRNA OS=Homo sapiens OX=9606 PE=2 SV=1 | 851508.2 |
| Pre-mRNA-splicing factor 3 OS=Homo sapiens OX=9606 PE=2 SV=1 | 1.3E+08 |
| Cleavage and polyadenylation specificity factor subunit 5 OS=Homo sapiens OX=9606 PE=2 SV=1 | 19432151 |
| cDNA, FLJ92684, highly similar to Homo sapiens IK cytokine, down-regulator of HLA II (IK), mRNA OS=Homo sapiens OX=9606 PE=2 SV=1 | 16273035 |
| cDNA, FLJ92106, highly similar to Homo sapiens adaptor-related protein complex 3, sigma 1 subunit(AP3S1), mRNA OS=Homo sapiens OX=9606 PE=2 SV=1 | 14832725 |
| URCC5 OS=Homo sapiens OX=9606 GN=URCC5 PE=2 SV=1 | 2591948 |
| Dual-specificity protein kinase CLK2 OS=Homo sapiens OX=9606 GN=CLK2 PE=1 SV=1 | 1897331 |
| Ribose-phosphate pyrophosphokinase 1 OS=Homo sapiens OX=9606 GN=PRPS1 PE=1 SV=2 | 22360464 |
| ATP-dependent DNA helicase 2 subunit 1 OS=Homo sapiens OX=9606 GN=XRCC6 PE=1 SV=1 | 14696465 |
| Heterochromatin protein 1-binding protein 3 (Fragment) OS=Homo sapiens OX=9606 GN=HP1BP3 PE=1 SV=1 | 2996804 |
| RNA-binding protein EWS OS=Homo sapiens OX=9606 GN=EWSR1 PE=1 SV=1 | 6.48E+08 |
| cDNA, FLJ79477, highly similar to Protein-glutamine gamma-glutamyltransferase K OS=Homo sapiens OX=9606 PE=2 SV=1 | 2315044 |
| Non-specific protein-tyrosine kinase OS=Homo sapiens OX=9606 GN=BCR/ABL fusion PE=2 SV=1 | 241333.8 |
| Matrin-3 OS=Homo sapiens OX=9606 GN=MATR3 PE=1 SV=1 | 39005322 |
| General transcription and DNA repair factor IIH helicase subunit XPD (Fragment) OS=Homo sapiens OX=9606 GN=ERCC2 PE=1 SV=2 | 4031713 |
| Zinc finger B-box domain-containing protein 1 OS=Homo sapiens OX=9606 GN=ZBBX PE=2 SV=3 | 10454044 |
| Afadin OS=Homo sapiens OX=9606 GN=AFDN PE=1 SV=2 | 1037379 |
| cDNA FLJ75444, highly similar to Homo sapiens protein kinase, cAMP-dependent, regulatory, type II, alpha (PRKAR2A), mRNA OS=Homo sapiens OX=9606 PE=2 SV=1 | 2655683 |
| cDNA FLJ76065 OS=Homo sapiens OX=9606 PE=2 SV=1 | 51239888 |
| rRNA adenine N(6)-methyltransferase OS=Homo sapiens OX=9606 PE=2 SV=1 | 79272805 |
| cDNA FLJ76962, highly similar to Homo sapiens nucleolar protein 5A (56kDa with KKE/D repeat) (NOL5A), mRNA OS=Homo sapiens OX=9606 PE=2 SV=1 | 28980928 |
| cDNA FLJ77771, highly similar to Homo sapiens replication initiator 1, mRNA OS=Homo sapiens OX=9606 PE=2 SV=1 | 20707621 |
| Pinin OS=Homo sapiens OX=9606 PE=2 SV=1 | 29005025 |
| cDNA FLJ78298 OS=Homo sapiens OX=9606 PE=2 SV=1 | 6510342 |
| cDNA FLJ77630, highly similar to Homo sapiens BPY2 interacting protein 1, mRNA OS=Homo sapiens OX=9606 PE=1 SV=1 | 18007916 |
| cDNA FLJ77850 OS=Homo sapiens OX=9606 PE=2 SV=1 | 7018009 |
| Centromere protein U OS=Homo sapiens OX=9606 PE=2 SV=1 | 8685131 |
| cDNA FLJ75784, highly similar to Homo sapiens CD3E antigen, epsilon polypeptide associated protein (CD3EAP), mRNA OS=Homo sapiens OX=9606 PE=2 SV=1 | 1115602 |
| RNA helicase OS=Homo sapiens OX=9606 PE=2 SV=1 | 17776335 |
| RNA helicase OS=Homo sapiens OX=9606 PE=2 SV=1 | 8042817 |
| Serine/threonine-protein phosphatase 2A 56 kDa regulatory subunit OS=Homo sapiens OX=9606 PE=2 SV=1 | 1842514 |
| cDNA FLJ78675, highly similar to Homo sapiens stroma HMG-box containing protein (HBP2) mRNA OS=Homo sapiens OX=9606 PE=2 SV=1 | 372387.8 |
| cDNA FLJ76888, highly similar to Homo sapiens RNA binding motif protein 6 (RBM6), mRNA OS=Homo sapiens OX=9606 PE=2 SV=1 | 6922920 |
| Complement component 1 Q subcomponent-binding protein, mitochondrial OS=Homo sapiens OX=9606 PE=2 SV=1 | 4767140 |
| cDNA FLJ77542, highly similar to Homo sapiens YME1-like 1 (S. cerevisiae) (YME1L1), transcript variant 3, mRNA OS=Homo sapiens OX=9606 PE=2 SV=1 | 20857020 |
| Exonuclease 1 OS=Homo sapiens OX=9606 PE=2 SV=1 | 1427034 |
| cDNA FLJ77424, highly similar to Homo sapiens anillin, actin binding protein (scraps homolog, Drosophila), mRNA OS=Homo sapiens OX=9606 PE=2 SV=1 | 13227459 |
| cDNA FLJ76823, highly similar to Homo sapiens splicing factor, arginine/serine-rich 6 (SFRS6), mRNA OS=Homo sapiens OX=9606 PE=2 SV=1 | 99942745 |
| AP-3 complex subunit beta OS=Homo sapiens OX=9606 PE=2 SV=1 | 8973488 |
| DNA topoisomerase OS=Homo sapiens OX=9606 PE=2 SV=1 | 19107515 |
| Cyclin-T1 OS=Homo sapiens OX=9606 PE=2 SV=1 | 12672079 |
| Female-lethal(2)D homolog OS=Homo sapiens OX=9606 PE=2 SV=1 | 30496993 |
| Kinesin-like protein OS=Homo sapiens OX=9606 PE=2 SV=1 | 15493794 |
| Ribosome biogenesis protein BOP1 OS=Homo sapiens OX=9606 GN=BOP1 PE=2 SV=1 | 2091023 |
| cDNA FLJ77680, highly similar to Homo sapiens protein phosphatase 2 (formerly 2A), regulatory subunit A (PR 65), alpha isoform (PPP2R1A), mRNA OS=Homo sapiens OX=9606 PE=2 SV=1 | 22136759 |
| cDNA FLJ76049 OS=Homo sapiens OX=9606 PE=2 SV=1 | 429620 |
| cDNA FLJ77715, highly similar to Homo sapiens cisplatin resistance-associated overexpressed protein(LUC7A), mRNA OS=Homo sapiens OX=9606 PE=2 SV=1 | 18758288 |
| cDNA FLJ77041, highly similar to Homo sapiens RAS protein activator like 2 (RASAL2), transcript variant 1, mRNA (Fragment) OS=Homo sapiens OX=9606 PE=2 SV=1 | 520528.2 |
| RRP15-like protein OS=Homo sapiens OX=9606 PE=2 SV=1 | 4933952 |
| cDNA FLJ75032, highly similar to Homo sapiens unc-84 homolog B (C. elegans) (UNC84B), mRNA OS=Homo sapiens OX=9606 PE=2 SV=1 | 798210.4 |
| cDNA FLJ78425, highly similar to Homo sapiens zinc fingers and homeoboxes 1 (ZHX1), mRNA OS=Homo sapiens OX=9606 PE=2 SV=1 | 950232 |
| cDNA FLJ78373, highly similar to Homo sapiens PCI domain containing 2, mRNA OS=Homo sapiens OX=9606 PE=2 SV=1 | 14372195 |
| DnaJ OS=Homo sapiens OX=9606 PE=2 SV=1 | 2833792 |
| SHINC3 OS=Homo sapiens OX=9606 GN=SHINC3 PE=2 SV=1 | 8298008 |
| Phosphatidylinositol 4-phosphate 5-kinase type-1 alpha OS=Homo sapiens OX=9606 GN=PIP5K1A PE=1 SV=2 | 4942249 |
| Mediator of RNA polymerase II transcription subunit 20 (Fragment) OS=Homo sapiens OX=9606 GN=MED20 PE=1 SV=1 | 640434.6 |
| Polypyrimidine tract-binding protein 1 OS=Homo sapiens OX=9606 GN=PTBP1 PE=1 SV=4 | 33495747 |
| Signal sequence receptor subunit delta OS=Homo sapiens OX=9606 GN=SSR4 PE=1 SV=1 | 487884.7 |
| Coiled-coil-helix-coiled-coil-helix domain-containing protein 1 OS=Homo sapiens OX=9606 GN=CHCHD1 PE=1 SV=1 | 1418572 |
| Small nuclear ribonucleoprotein E OS=Homo sapiens OX=9606 GN=SNRPE PE=1 SV=1 | 31145376 |
| Zinc finger protein 316 OS=Homo sapiens OX=9606 GN=ZNF316 PE=1 SV=1 | 1461657 |
| 28S ribosomal protein S16, mitochondrial OS=Homo sapiens OX=9606 GN=MRPS16 PE=1 SV=1 | 1113399 |
| Nibrin OS=Homo sapiens OX=9606 GN=NBN PE=2 SV=1 | 2882782 |
| Insulin receptor (Fragment) OS=Homo sapiens OX=9606 PE=4 SV=1 | 923102 |
| Mitochondrial 28S ribosomal protein S34 OS=Homo sapiens OX=9606 PE=2 SV=1 | 4165434 |
| IQ motif containing GTPase activating protein 1 OS=Homo sapiens OX=9606 GN=IQGAP1 PE=1 SV=1 | 8182628 |
| NADH-ubiquinone oxidoreductase chain 1 OS=Homo sapiens OX=9606 GN=ND1 PE=3 SV=1 | 2413316 |
| MTCH1 protein (Fragment) OS=Homo sapiens OX=9606 GN=MTCH1 PE=2 SV=1 | 2252179 |
| 40S ribosomal protein S2 OS=Homo sapiens OX=9606 GN=LOC392781 PE=3 SV=1 | 461951.8 |
| 3-methyladenine DNA glycosidase (Fragment) OS=Homo sapiens OX=9606 GN=MPG PE=1 SV=1 | 4274428 |
| Valine--tRNA ligase (Fragment) OS=Homo sapiens OX=9606 GN=VARS1 PE=1 SV=1 | 564196.9 |
| TBL3 protein (Fragment) OS=Homo sapiens OX=9606 GN=TBL3 PE=2 SV=1 | 2637989 |
| Brix domain-containing protein 2 (Fragment) OS=Homo sapiens OX=9606 GN=BXDC2 PE=2 SV=1 | 3810464 |
| EDC4 variant OS=Homo sapiens OX=9606 GN=EDC4 PE=2 SV=1 | 6569302 |
| Isoleucine--tRNA ligase, cytoplasmic OS=Homo sapiens OX=9606 GN=IARS1 PE=4 SV=1 | 34157433 |
| IGH c3104_heavy_IGHV1-69_IGHD3-22_IGHJ4 (Fragment) OS=Homo sapiens OX=9606 PE=2 SV=1 | 1.34E+08 |
| IGH c3252_heavy_IGHV3-73_IGHD6-13_IGHJ4 (Fragment) OS=Homo sapiens OX=9606 PE=2 SV=1 | 30036430 |
| Serine/threonine-protein kinase VRK1 OS=Homo sapiens OX=9606 GN=VRK1 PE=1 SV=1 | 488327.4 |
| Recombining-binding protein suppressor of hairless (Fragment) OS=Homo sapiens OX=9606 GN=RBPJ PE=1 SV=1 | 887022 |
| Oligophrenin-1 OS=Homo sapiens OX=9606 GN=OPHN1 PE=1 SV=1 | 4819422 |
| RNA helicase OS=Homo sapiens OX=9606 GN=DDX20 PE=1 SV=1 | 1990100 |
| Peptidyl-prolyl cis-trans isomerase OS=Homo sapiens OX=9606 GN=PPIB PE=1 SV=1 | 543208.5 |
| Transitional endoplasmic reticulum ATPase OS=Homo sapiens OX=9606 GN=VCP PE=1 SV=1 | 2862079 |
| Sorting nexin OS=Homo sapiens OX=9606 GN=SNX9 PE=1 SV=1 | 548301.1 |
| UPF0688 protein C1orf174 OS=Homo sapiens OX=9606 GN=C1orf174 PE=1 SV=1 | 5247098 |
| WD repeat-containing protein 26 OS=Homo sapiens OX=9606 GN=WDR26 PE=1 SV=1 | 1406622 |
| Heterogeneous nuclear ribonucleoprotein Q OS=Homo sapiens OX=9606 GN=SYNCRIP PE=1 SV=1 | 21833300 |
| Ribosomal protein L19 OS=Homo sapiens OX=9606 GN=RPL19 PE=1 SV=1 | 3.62E+08 |
| Eukaryotic translation initiation factor 3 subunit I OS=Homo sapiens OX=9606 GN=EIF3I PE=1 SV=1 | 34243498 |
| Polyadenylate-binding protein OS=Homo sapiens OX=9606 GN=PABPC1 PE=1 SV=1 | 470092.3 |
| Polyadenylate-binding protein 1 OS=Homo sapiens OX=9606 GN=PABPC1 PE=1 SV=1 | 4.75E+08 |
| Transcription factor BTF3 OS=Homo sapiens OX=9606 GN=BTF3 PE=1 SV=1 | 13903084 |
| Telomeric repeat-binding factor OS=Homo sapiens OX=9606 GN=TERF1 PE=1 SV=1 | 2130151 |
| DNA (cytosine-5)-methyltransferase OS=Homo sapiens OX=9606 GN=DNMT1 PE=1 SV=1 | 3522183 |
| Non-POU domain-containing octamer-binding protein OS=Homo sapiens OX=9606 GN=NONO PE=1 SV=1 | 4.77E+09 |
| ATP-dependent RNA helicase DDX1 OS=Homo sapiens OX=9606 GN=DDX1 PE=1 SV=1 | 6.35E+08 |
| Importin subunit alpha-1 OS=Homo sapiens OX=9606 GN=KPNA2 PE=1 SV=1 | 77347468 |
| Ras GTPase-activating protein-binding protein 2 OS=Homo sapiens OX=9606 GN=G3BP2 PE=1 SV=1 | 17418663 |
| MAP/microtubule affinity-regulating kinase 3 OS=Homo sapiens OX=9606 GN=MARK3 PE=1 SV=1 | 1201750 |
| GRB10-interacting GYF protein 2 OS=Homo sapiens OX=9606 GN=GIGYF2 PE=1 SV=1 | 9526256 |
| Heterogeneous nuclear ribonucleoproteins A2/B1 OS=Homo sapiens OX=9606 GN=HNRNPA2B1 PE=1 SV=1 | 1.28E+08 |
| Exportin-1 OS=Homo sapiens OX=9606 GN=XPO1 PE=1 SV=1 | 672314.6 |
| Eukaryotic translation initiation factor 3 subunit E OS=Homo sapiens OX=9606 GN=EIF3E PE=1 SV=1 | 73172302 |
| Ubiquitin carboxyl-terminal hydrolase OS=Homo sapiens OX=9606 PE=2 SV=1 | 1.59E+08 |
| E3 ubiquitin-protein ligase TRIP12 OS=Homo sapiens OX=9606 GN=TRIP12 PE=1 SV=1 | 2213169 |
| Kinesin-like protein OS=Homo sapiens OX=9606 GN=KIF2A PE=1 SV=1 | 85837592 |
| DNA-binding protein SMUBP-2 (Fragment) OS=Homo sapiens OX=9606 GN=IGHMBP2 PE=1 SV=1 | 42448000 |
| Golgin subfamily A member 2 OS=Homo sapiens OX=9606 GN=GOLGA2 PE=1 SV=1 | 5298077 |
| Bifunctional lysine-specific demethylase and histidyl-hydroxylase OS=Homo sapiens OX=9606 GN=MDIG PE=2 SV=1 | 3200393 |
| Guanine nucleotide binding protein-like 3 (Nucleolar)-like, isoform CRA_b OS=Homo sapiens OX=9606 GN=GNL3L PE=1 SV=1 | 45654862 |
| Treacle protein (Fragment) OS=Homo sapiens OX=9606 GN=TCOF1 PE=1 SV=1 | 1189855 |
| Leucyl-tRNA synthetase OS=Homo sapiens OX=9606 GN=LARS1 PE=1 SV=1 | 19342595 |
| CXXC-type zinc finger protein 1 OS=Homo sapiens OX=9606 GN=CXXC1 PE=1 SV=1 |  |
| Cytochrome c oxidase subunit 2 OS=Homo sapiens OX=9606 GN=COX2 PE=3 SV=1 | 753512.5 |
| RNA helicase OS=Homo sapiens OX=9606 GN=DDX17 PE=1 SV=2 | 8.49E+08 |
| Ataxin-2 OS=Homo sapiens OX=9606 GN=ATXN2 PE=1 SV=1 | 51252201 |
| Peregrin OS=Homo sapiens OX=9606 GN=BRPF1 PE=1 SV=2 | 281747.2 |
| Tyrosine-protein kinase (Fragment) OS=Homo sapiens OX=9606 GN=JAK1 PE=1 SV=1 |  |
| IG c1505_light_IGKV3-11_IGKJ4 (Fragment) OS=Homo sapiens OX=9606 PE=2 SV=1 | 1355489 |
| IG c35_light_IGKV3-11_IGKJ4 (Fragment) OS=Homo sapiens OX=9606 PE=2 SV=1 | 3530532 |
| IG c775_heavy_IGHV3-15_IGHD3-10_IGHJ4 (Fragment) OS=Homo sapiens OX=9606 PE=2 SV=1 | 8862273 |
| IG c473_heavy_IGHV3-7_IGHD5-12_IGHJ3 (Fragment) OS=Homo sapiens OX=9606 PE=2 SV=1 |  |
| IG c13_heavy_IGHV3-15_IGHD3-3_IGHJ4 (Fragment) OS=Homo sapiens OX=9606 PE=2 SV=1 | 2032275 |
| IGH c241_heavy__IGHV3-15_IGHD3-10_IGHJ4 (Fragment) OS=Homo sapiens OX=9606 PE=2 SV=1 | 18661455 |
| IGH + IGL c23_heavy_IGHV3-49_IGHD5-24_IGHJ6 (Fragment) OS=Homo sapiens OX=9606 PE=2 SV=1 | 4312137 |
| IGH + IGL c463_heavy_IGHV3-11_IGHD3-3_IGHJ5 (Fragment) OS=Homo sapiens OX=9606 PE=2 SV=1 | 3597847 |
| IGL c1933_light_IGKV3-11_IGKJ4 (Fragment) OS=Homo sapiens OX=9606 PE=2 SV=1 | 3.11E+08 |
| DNA mismatch repair protein Msh3 OS=Homo sapiens OX=9606 GN=MSH3 PE=1 SV=1 | 663654.6 |
| MHC class II antigen (Fragment) OS=Homo sapiens OX=9606 GN=HLA-DPA1 PE=3 SV=1 | 681613.3 |
| RNA-binding protein 15 OS=Homo sapiens OX=9606 GN=RBM15 PE=1 SV=1 | 37621019 |
| General transcription factor II-I repeat domain-containing protein 2B OS=Homo sapiens OX=9606 GN=GTF2IRD2B PE=4 SV=1 | 473200.1 |
| Protein KRI1 homolog OS=Homo sapiens OX=9606 GN=KRI1 PE=1 SV=1 | 1785700 |
| Constitutive coactivator of PPAR-gamma-like protein 1 (Fragment) OS=Homo sapiens OX=9606 GN=FAM120A PE=1 SV=1 | 2651223 |
| Protein arginine N-methyltransferase 1 transcript variant 22 OS=Homo sapiens OX=9606 GN=PRMT1 PE=2 SV=1 | 25689067 |
| Survival of motor neuron 1 isoform D345 OS=Homo sapiens OX=9606 GN=SMN2 PE=2 SV=1 |  |
| Ribonucloprotein OS=Homo sapiens OX=9606 GN=SNU13 PE=1 SV=1 | 10627254 |
| Uncharacterized protein OS=Homo sapiens OX=9606 PE=3 SV=1 | 3410709 |
| Coatomer subunit alpha (Fragment) OS=Homo sapiens OX=9606 GN=COPA PE=1 SV=1 | 1459884 |
| Translation initiation factor eIF-2B subunit epsilon (Fragment) OS=Homo sapiens OX=9606 GN=EIF2B5 PE=1 SV=1 | 654942.8 |
| Ricin B-type lectin domain-containing protein OS=Homo sapiens OX=9606 PE=1 SV=1 | 620268.8 |
| La-related protein 1B OS=Homo sapiens OX=9606 GN=LARP1B PE=1 SV=1 | 2013919 |
| DNA polymerase subunit gamma-1 OS=Homo sapiens OX=9606 GN=POLG PE=1 SV=2 | 2125313 |
| UPF0488 protein C8orf33 OS=Homo sapiens OX=9606 GN=C8orf33 PE=1 SV=1 | 2392100 |
| CTP synthase OS=Homo sapiens OX=9606 GN=CTPS1 PE=1 SV=1 |  |
| Tubulin beta chain (Fragment) OS=Homo sapiens OX=9606 PE=2 SV=1 | 10719417 |
| RNA helicase OS=Homo sapiens OX=9606 PE=2 SV=1 | 9361037 |
| Elongation factor Tu OS=Homo sapiens OX=9606 GN=TUFM PE=1 SV=1 | 8648195 |
| Patatin-like phospholipase domain-containing protein 6 OS=Homo sapiens OX=9606 GN=PNPLA6 PE=1 SV=1 | 4271052 |
| Forkhead box protein O3B OS=Homo sapiens OX=9606 GN=FOXO3B PE=1 SV=1 | 574091.1 |
| Complex I-B14 OS=Homo sapiens OX=9606 GN=NDUFA6 PE=1 SV=1 | 409653.8 |
| NET1 (Fragment) OS=Homo sapiens OX=9606 GN=NET1 PE=4 SV=1 | 637611.2 |
| ARHGEF40 (Fragment) OS=Homo sapiens OX=9606 GN=ARHGEF40 PE=4 SV=1 | 152725.3 |
| SR-related CTD associated factor 1 transcript variant 15 OS=Homo sapiens OX=9606 GN=SCAF1 PE=2 SV=1 | 876999.6 |
| DNA helicase OS=Homo sapiens OX=9606 GN=CHD4 PE=1 SV=1 | 32827180 |
| Casein kinase II subunit alpha OS=Homo sapiens OX=9606 GN=CSNK2A1 PE=1 SV=1 | 1015918 |
| Sorting nexin-27 OS=Homo sapiens OX=9606 GN=SNX27 PE=1 SV=1 | 728650.6 |
| E3 ubiquitin-protein ligase RBBP6 (Fragment) OS=Homo sapiens OX=9606 GN=RBBP6 PE=1 SV=1 | 8369407 |
| SWI/SNF-related matrix-associated actin-dependent regulator of chromatin subfamily E member 1 OS=Homo sapiens OX=9606 GN=SMARCE1 PE=1 SV=1 | 808655.2 |
| 40S ribosomal protein S24 OS=Homo sapiens OX=9606 GN=RPS24 PE=1 SV=1 | 1.41E+08 |
| 40S ribosomal protein S14 (Fragment) OS=Homo sapiens OX=9606 GN=RPS14 PE=1 SV=1 | 2.45E+09 |
| Nucleosome-remodeling factor subunit BPTF OS=Homo sapiens OX=9606 GN=BPTF PE=1 SV=1 | 13599491 |
| Ribosomal protein L15 (Fragment) OS=Homo sapiens OX=9606 GN=RPL15 PE=1 SV=1 | 1.7E+08 |
| 60S ribosomal protein L5 (Fragment) OS=Homo sapiens OX=9606 GN=RPL5 PE=1 SV=1 | 1.11E+08 |
| 60S ribosomal protein L9 OS=Homo sapiens OX=9606 GN=RPL9 PE=1 SV=1 | 1.49E+08 |
| Radixin OS=Homo sapiens OX=9606 GN=RDX PE=1 SV=1 | 1.16E+08 |
| Centrosomal protein of 78 kDa OS=Homo sapiens OX=9606 GN=CEP78 PE=1 SV=1 | 1236145 |
| LEMD2 OS=Homo sapiens OX=9606 GN=LEMD2 PE=2 SV=1 | 1877781 |
| GTF2I-BRAF fusion protein OS=Homo sapiens OX=9606 GN=GTF2I PE=2 SV=1 | 14233637 |
| Mediator of RNA polymerase II transcription subunit 17 OS=Homo sapiens OX=9606 GN=MED17 PE=1 SV=1 | 485060.3 |
| Glial fibrillary acidic protein (Fragment) OS=Homo sapiens OX=9606 GN=GFAP PE=1 SV=1 | 1288594 |
| PRRC2A OS=Homo sapiens OX=9606 PE=4 SV=1 | 4.19E+08 |
| Corneodesmosin OS=Homo sapiens OX=9606 PE=4 SV=1 | 730074.8 |
| SKIV2L OS=Homo sapiens OX=9606 PE=4 SV=1 | 369906.8 |
| 28S ribosomal protein S18-2, mitochondrial OS=Homo sapiens OX=9606 PE=3 SV=1 | 1033971 |
| RNA helicase OS=Homo sapiens OX=9606 GN=DHX16 PE=4 SV=1 | 6023656 |
| BRD2 OS=Homo sapiens OX=9606 PE=4 SV=1 | 2454017 |
| Probable C-mannosyltransferase DPY19L1 OS=Homo sapiens OX=9606 GN=DPY19L1 PE=1 SV=1 | 1316648 |
| Keratin, type I cytoskeletal 10 OS=Homo sapiens OX=9606 GN=KRT10 PE=1 SV=2 | 3.14E+09 |
| Ryanodine receptor 3 (Fragment) OS=Homo sapiens OX=9606 GN=RYR3 PE=4 SV=1 | 429351.3 |
| Lactotransferrin OS=Homo sapiens OX=9606 GN=LTF PE=2 SV=1 | 416364.4 |
| Testis tissue sperm-binding protein Li 90mP OS=Homo sapiens OX=9606 PE=2 SV=1 | 715069.6 |
| Non-muscle myosin heavy chain 9 OS=Homo sapiens OX=9606 GN=MYH9 PE=2 SV=1 | 3802992 |
| Casein kinase I isoform gamma-1 (Fragment) OS=Homo sapiens OX=9606 GN=CSNK1G1 PE=1 SV=1 | 953553.8 |
| Methyltransferase like 17 isoform 1 (Fragment) OS=Homo sapiens OX=9606 GN=METTL17 PE=2 SV=1 | 2483739 |
| DNA helicase (Fragment) OS=Homo sapiens OX=9606 GN=MCM8 PE=2 SV=1 | 1096691 |
| CWF19-like 1 cell cycle control isoform 3 OS=Homo sapiens OX=9606 GN=CWF19L1 PE=1 SV=1 | 420883.3 |
| Spermatid perinuclear RNA-binding protein (Fragment) OS=Homo sapiens OX=9606 GN=STRBP PE=1 SV=1 | 1266692 |
| Serine/arginine repetitive matrix 1 isoform 2 (Fragment) OS=Homo sapiens OX=9606 GN=SRRM1 PE=1 SV=1 | 72853453 |
| Epididymis secretory sperm binding protein (Fragment) OS=Homo sapiens OX=9606 GN=RBM10 PE=2 SV=1 | 1.21E+08 |
| Nucleophosmin (Fragment) OS=Homo sapiens OX=9606 GN=NPM1 PE=2 SV=1 | 2.64E+08 |
| Centromere protein V OS=Homo sapiens OX=9606 GN=CENPV PE=1 SV=1 | 1376961 |
| 60 kDa poly(U)-binding-splicing factor (Fragment) OS=Homo sapiens OX=9606 GN=PUF60 PE=1 SV=1 | 1.14E+08 |
| POM121 and ZP3 fusion protein (Fragment) OS=Homo sapiens OX=9606 GN=POMZP3 PE=4 SV=1 | 1941692 |
| ATP-dependent RNA helicase DDX3X (Fragment) OS=Homo sapiens OX=9606 GN=DDX3X PE=1 SV=2 | 5226234 |
| AP-2 complex subunit alpha-2 (Fragment) OS=Homo sapiens OX=9606 GN=AP2A2 PE=1 SV=1 | 44899384 |
| Heat shock 70 kDa protein 1B OS=Homo sapiens OX=9606 GN=HSPA1B PE=1 SV=1 | 2.75E+08 |
| Plasminogen OS=Homo sapiens OX=9606 GN=PLG PE=2 SV=1 | 523082.4 |
| PHD finger protein 6 OS=Homo sapiens OX=9606 GN=PHF6 PE=1 SV=1 | 64469452 |
| Insulin-induced gene protein OS=Homo sapiens OX=9606 GN=INSIG2 PE=1 SV=1 | 28034904 |
| Dehydrogenase/reductase SDR family member 7B OS=Homo sapiens OX=9606 GN=DHRS7B PE=1 SV=1 | 654778.4 |
| Cell division cycle and apoptosis regulator protein 1 (Fragment) OS=Homo sapiens OX=9606 GN=CCAR1 PE=1 SV=1 | 521431.3 |
| RNA helicase OS=Homo sapiens OX=9606 GN=DDX46 PE=1 SV=1 | 3364626 |
| Protein LSM14 homolog B OS=Homo sapiens OX=9606 GN=LSM14B PE=1 SV=1 | 1011660 |
| 1,4-beta-N-acetylmuramidase C OS=Homo sapiens OX=9606 GN=LYZ PE=1 SV=1 | 856603.8 |
| HCG2039996 OS=Homo sapiens OX=9606 GN=PPAN-P2RY11 PE=3 SV=1 | 20220105 |
| WD repeat-containing protein 36 OS=Homo sapiens OX=9606 GN=WDR36 PE=1 SV=1 | 3746155 |
| M-phase phosphoprotein 8 (Fragment) OS=Homo sapiens OX=9606 GN=MPHOSPH8 PE=1 SV=1 | 481754.6 |
| Chromodomain-helicase-DNA-binding protein 1-like OS=Homo sapiens OX=9606 GN=CHD1L PE=1 SV=2 | 850806 |
| Nucleolar and coiled-body phosphoprotein 1 (Fragment) OS=Homo sapiens OX=9606 GN=NOLC1 PE=1 SV=1 | 2141370 |
| Diacylglycerol O-acyltransferase 1 OS=Homo sapiens OX=9606 GN=DGAT1 PE=1 SV=1 | 372477.6 |
| Fibroblast growth factor OS=Homo sapiens OX=9606 GN=FGF2 PE=1 SV=1 | 3199166 |
| Similar to Importin alpha-2 subunit (Karyopherin alpha-2 subunit) (SRP1-alpha) (RAG cohort protein 1) OS=Homo sapiens OX=9606 GN=LOC340312 PE=3 SV=1 | 6461040 |
| ATP-binding cassette, sub-family F (GCN20), member 2 OS=Homo sapiens OX=9606 GN=ABCF2 PE=4 SV=1 | 74004303 |
| WD repeat-containing protein 6 OS=Homo sapiens OX=9606 GN=WDR6 PE=1 SV=1 | 9411636 |
| TAR DNA-binding protein 43 OS=Homo sapiens OX=9606 GN=TARDBP PE=1 SV=1 | 1731491 |
| E3 ubiquitin-protein ligase Midline-1 OS=Homo sapiens OX=9606 GN=MID1 PE=1 SV=1 | 625606.7 |
| Ankyrin repeat domain-containing protein 36B OS=Homo sapiens OX=9606 GN=ANKRD36B PE=4 SV=1 | 9692659 |
| Probable ATP-dependent RNA helicase DDX52 (Fragment) OS=Homo sapiens OX=9606 GN=DDX52 PE=1 SV=1 | 618344.2 |
| Heterogeneous nuclear ribonucleoprotein M OS=Homo sapiens OX=9606 GN=HNRNPM PE=1 SV=1 | 1.55E+08 |
| Ubiquitinyl hydrolase 1 OS=Homo sapiens OX=9606 GN=OTUD6B PE=1 SV=1 | 869780.3 |
| Kanadaptin OS=Homo sapiens OX=9606 GN=SLC4A1AP PE=1 SV=1 | 1237378 |
| RNA-binding protein 26 OS=Homo sapiens OX=9606 GN=RBM26 PE=1 SV=1 | 2187409 |
| ATP-dependent RNA helicase DHX29 OS=Homo sapiens OX=9606 GN=DHX29 PE=1 SV=1 | 8052306 |
| Nuclear mitotic apparatus protein 1 OS=Homo sapiens OX=9606 GN=NUMA1 PE=1 SV=1 | 439603.8 |
| Nucleolar protein of 40 kDa OS=Homo sapiens OX=9606 GN=ZCCHC17 PE=1 SV=1 |  |
| Chromosome 20 open reading frame 129 OS=Homo sapiens OX=9606 GN=FAM83D PE=1 SV=1 | 2005799 |
| Formyltetrahydrofolate synthetase OS=Homo sapiens OX=9606 GN=MTHFD1L PE=1 SV=1 | 1940866 |
| Uncharacterized protein OS=Homo sapiens OX=9606 PE=3 SV=1 | 1865719 |
| Oligosaccharyltransferase complex subunit OS=Homo sapiens OX=9606 GN=OSTC PE=1 SV=1 | 1564886 |
| Centromere protein P OS=Homo sapiens OX=9606 GN=CENPP PE=1 SV=1 | 506253.8 |
| DDB1- and CUL4-associated factor 13 OS=Homo sapiens OX=9606 GN=DCAF13 PE=1 SV=1 | 2943553 |
| Ribonuclease III, nuclear, isoform CRA_a OS=Homo sapiens OX=9606 GN=RNASEN PE=3 SV=1 | 1101869 |
| Heat shock 110 kDa protein OS=Homo sapiens OX=9606 GN=HSPH1 PE=3 SV=1 | 1752901 |
| Ras-GTPase activating protein SH3 domain-binding protein 2, isoform CRA_b OS=Homo sapiens OX=9606 GN=G3BP2 PE=4 SV=1 | 3.58E+08 |
| Centromere protein Q OS=Homo sapiens OX=9606 GN=C6orf139 PE=3 SV=1 | 1550981 |
| Protein regulator of cytokinesis 1, isoform CRA_e OS=Homo sapiens OX=9606 GN=PRC1 PE=4 SV=1 | 12761341 |
| Zinc finger protein 24 (KOX 17), isoform CRA_b OS=Homo sapiens OX=9606 GN=ZNF24 PE=1 SV=1 | 4958261 |
| Microtubule-associated protein 1B, isoform CRA_b OS=Homo sapiens OX=9606 GN=MAP1B PE=4 SV=1 | 1656770 |
| Elongin-A OS=Homo sapiens OX=9606 GN=ELOA PE=1 SV=1 | 14236680 |
| Septin OS=Homo sapiens OX=9606 GN=SEPT7 PE=3 SV=1 | 208151.1 |
| Mitochondrial ribosomal protein S2, isoform CRA_a OS=Homo sapiens OX=9606 GN=MRPS2 PE=3 SV=1 | 3140544 |
| UPF1 OS=Homo sapiens OX=9606 GN=UPF1 PE=2 SV=1 | 24548450 |
| Splicing factor, arginine/serine-rich 14, isoform CRA_a OS=Homo sapiens OX=9606 GN=SFRS14 PE=4 SV=1 | 767018.1 |
| Numb homolog (Drosophila), isoform CRA_f OS=Homo sapiens OX=9606 GN=NUMB PE=4 SV=1 | 7461045 |
| HCG2039447, isoform CRA_d OS=Homo sapiens OX=9606 GN=hCG_2039447 PE=1 SV=1 | 14223748 |
| 3-beta-hydroxysterol Delta (14)-reductase OS=Homo sapiens OX=9606 GN=LBR PE=3 SV=1 | 14930965 |
| Chromosome 11 open reading frame 57, isoform CRA_a OS=Homo sapiens OX=9606 GN=C11orf57 PE=4 SV=1 | 2531985 |
| 60S ribosomal protein L29 OS=Homo sapiens OX=9606 GN=RPL29 PE=1 SV=1 | 2.32E+08 |
| ADA3 homolog OS=Homo sapiens OX=9606 GN=TADA3L PE=2 SV=1 | 256981.3 |
| Tight junction protein 2 (Zona occludens 2), isoform CRA_a OS=Homo sapiens OX=9606 GN=TJP2 PE=4 SV=1 | 11084151 |
| Protein CASC3 OS=Homo sapiens OX=9606 GN=CASC3 PE=3 SV=1 | 1297426 |
| GTP-binding protein 1 OS=Homo sapiens OX=9606 GN=GTPBP1 PE=4 SV=1 | 27400130 |
| Activating signal cointegrator 1 complex subunit 2, isoform CRA_a OS=Homo sapiens OX=9606 GN=ASCC2 PE=4 SV=1 | 1204478 |
| Proline/serine-rich coiled-coil 1, isoform CRA_c OS=Homo sapiens OX=9606 GN=PSRC1 PE=4 SV=1 | 5223081 |
| Protein pelota homolog OS=Homo sapiens OX=9606 GN=hCG_2002731 PE=3 SV=1 | 1565074 |

| **Supplementary Table. S4 List of possible RAC1-interacting proteins in group 2 identified by Mass Spectrometry. (Group 2: 293T cells were transfected with Flag-RAC1 group)** | |
| --- | --- |
| Description | Abundances (Grouped): 2 |
| RAS-related C3 botulinum toxin substrate 1 OS=Homo sapiens OX=9606 GN=RPS3 PE=1 SV=1 | 157887112 |
| DNA-dependent protein kinase catalytic subunit OS=Homo sapiens OX=9606 GN=PRKDC PE=1 SV=3 | 30753304 |
| Eukaryotic translation initiation factor 5B OS=Homo sapiens OX=9606 GN=EIF5B PE=1 SV=4 | 1.89E+08 |
| Pre-mRNA-processing-splicing factor 8 OS=Homo sapiens OX=9606 GN=PRPF8 PE=1 SV=2 | 1.14E+08 |
| Splicing factor 3B subunit 3 OS=Homo sapiens OX=9606 GN=SF3B3 PE=1 SV=4 | 84978033 |
| Pre-mRNA-splicing factor ATP-dependent RNA helicase DHX15 OS=Homo sapiens OX=9606 GN=DHX15 PE=1 SV=2 | 89071930 |
| P21-activated kinases 4 OS=Homo sapiens OX=9606 GN=EIF3C PE=2 SV=1 | 66745311 |
| Poly [ADP-ribose] polymerase 1 OS=Homo sapiens OX=9606 GN=PARP1 PE=1 SV=4 | 95364048 |
| Signal recognition particle subunit SRP68 OS=Homo sapiens OX=9606 GN=SRP68 PE=1 SV=2 | 51312154 |
| Ribosome biogenesis protein BMS1 homolog OS=Homo sapiens OX=9606 GN=BMS1 PE=1 SV=1 | 29064507 |
| RNA-binding protein 28 OS=Homo sapiens OX=9606 GN=RBM28 PE=1 SV=3 | 45527613 |
| Probable ATP-dependent RNA helicase DDX27 OS=Homo sapiens OX=9606 GN=DDX27 PE=1 SV=2 | 28147554 |
| Small subunit processome component 20 homolog OS=Homo sapiens OX=9606 GN=UTP20 PE=1 SV=3 | 10438081 |
| Desmoplakin OS=Homo sapiens OX=9606 GN=DSP PE=1 SV=3 | 22520756 |
| RNA helicase OS=Homo sapiens OX=9606 GN=MOV10 PE=1 SV=1 | 31114093 |
| ESF1 homolog OS=Homo sapiens OX=9606 GN=ESF1 PE=1 SV=1 | 20178874 |
| Heat shock 70 kDa protein 1B OS=Homo sapiens OX=9606 GN=HSPA1B PE=1 SV=1 | 62692458 |
| Pre-rRNA-processing protein TSR1 homolog OS=Homo sapiens OX=9606 GN=TSR1 PE=1 SV=1 | 37830612 |
| AP-3 complex subunit delta-1 OS=Homo sapiens OX=9606 GN=AP3D1 PE=1 SV=1 | 26156089 |
| Cleavage and polyadenylation specificity factor subunit 1 OS=Homo sapiens OX=9606 GN=CPSF1 PE=1 SV=2 | 22234538 |
| cDNA FLJ76065 OS=Homo sapiens OX=9606 PE=2 SV=1 | 20978947 |
| Protein LYRIC OS=Homo sapiens OX=9606 GN=MTDH PE=1 SV=2 | 90360924 |
| PHD finger-like domain-containing protein 5A OS=Homo sapiens OX=9606 GN=PHF5A PE=1 SV=1 | 10177103 |
| 60S ribosomal protein L7 OS=Homo sapiens OX=9606 GN=RPL7 PE=1 SV=1 | 7.11E+08 |
| Cytoskeleton-associated protein 5 OS=Homo sapiens OX=9606 GN=CKAP5 PE=1 SV=3 | 13695285 |
| RNA-binding protein EWS (Fragment) OS=Homo sapiens OX=9606 GN=EWSR1 PE=1 SV=1 | 1345040 |
| 40S ribosomal protein S9 OS=Homo sapiens OX=9606 GN=RPS9 PE=1 SV=3 | 5.73E+08 |
| 60 kDa poly(U)-binding-splicing factor (Fragment) OS=Homo sapiens OX=9606 GN=PUF60 PE=1 SV=1 | 1.48E+08 |
| Probable ATP-dependent RNA helicase DDX47 OS=Homo sapiens OX=9606 GN=DDX47 PE=1 SV=1 | 34517620 |
| FBL protein (Fragment) OS=Homo sapiens OX=9606 GN=FBL PE=2 SV=2 | 20314553 |
| YLP motif-containing protein 1 OS=Homo sapiens OX=9606 GN=YLPM1 PE=1 SV=4 | 20366226 |
| 60S acidic ribosomal protein P0 OS=Homo sapiens OX=9606 GN=RPLP0 PE=1 SV=1 | 6.51E+08 |
| GTP-binding protein 4 OS=Homo sapiens OX=9606 GN=GTPBP4 PE=1 SV=3 | 55804382 |
| 60S ribosomal protein L27 (Fragment) OS=Homo sapiens OX=9606 GN=RPL27 PE=1 SV=1 | 67538807 |
| M-phase phosphoprotein 6 OS=Homo sapiens OX=9606 GN=MPHOSPH6 PE=1 SV=1 | 8112594 |
| DNA topoisomerase I OS=Homo sapiens OX=9606 GN=TOP1 PE=2 SV=1 | 1.82E+08 |
| Eukaryotic translation initiation factor 3 subunit E OS=Homo sapiens OX=9606 GN=EIF3E PE=1 SV=1 | 19051113 |
| Insulin-like growth factor 2 mRNA-binding protein 1 OS=Homo sapiens OX=9606 GN=IGF2BP1 PE=1 SV=2 | 1.95E+08 |
| MKI67 FHA domain-interacting nucleolar phosphoprotein OS=Homo sapiens OX=9606 GN=NIFK PE=1 SV=1 | 22119407 |
| Protein translocation complex beta variant (Fragment) OS=Homo sapiens OX=9606 PE=2 SV=1 | 3389574 |
| Keratin, type I cytoskeletal 17 OS=Homo sapiens OX=9606 GN=KRT17 PE=1 SV=2 | 5285347 |
| RNA-binding protein 25 OS=Homo sapiens OX=9606 GN=RBM25 PE=1 SV=3 | 2.31E+08 |
| 60S ribosomal protein L35 OS=Homo sapiens OX=9606 GN=RPL35 PE=1 SV=2 | 2.27E+08 |
| Bifunctional glutamate/proline--tRNA ligase OS=Homo sapiens OX=9606 GN=EPRS1 PE=1 SV=5 | 26549512 |
| Eukaryotic translation initiation factor 2 subunit 1 OS=Homo sapiens OX=9606 GN=EIF2S1 PE=1 SV=3 | 93547466 |
| ATP-dependent RNA helicase DDX50 OS=Homo sapiens OX=9606 GN=DDX50 PE=1 SV=1 | 44830483 |
| 60S ribosomal protein L10 OS=Homo sapiens OX=9606 GN=RPL10 PE=1 SV=2 | 97015542 |
| Heterogeneous nuclear ribonucleoproteins C1/C2 OS=Homo sapiens OX=9606 GN=HNRNPC PE=1 SV=1 | 39624361 |
| Heterogeneous nuclear ribonucleoprotein K OS=Homo sapiens OX=9606 PE=2 SV=1 | 3.69E+08 |
| cDNA, FLJ92227, highly similar to Homo sapiens ribosomal protein L36a-like (RPL36AL), mRNA OS=Homo sapiens OX=9606 PE=2 SV=1 | 3327202 |
| 40S ribosomal protein S24 OS=Homo sapiens OX=9606 GN=RPS24 PE=1 SV=1 | 28448605 |
| Peptidyl-prolyl cis-trans isomerase OS=Homo sapiens OX=9606 GN=HEL-S-39 PE=2 SV=1 | 2458020 |
| RNA-splicing ligase RtcB homolog OS=Homo sapiens OX=9606 GN=RTCB PE=1 SV=1 | 39898788 |
| Developmentally-regulated GTP-binding protein 1 OS=Homo sapiens OX=9606 GN=DRG1 PE=1 SV=1 | 32340539 |
| ATP-dependent RNA helicase DDX18 OS=Homo sapiens OX=9606 GN=DDX18 PE=1 SV=2 | 56401556 |
| RNA-binding protein 14 OS=Homo sapiens OX=9606 GN=RBM14 PE=1 SV=2 | 1.7E+08 |
| G3BP protein OS=Homo sapiens OX=9606 GN=G3BP PE=2 SV=1 | 57942152 |
| DNA topoisomerase 2-alpha OS=Homo sapiens OX=9606 GN=TOP2A PE=1 SV=3 | 15788950 |
| Splicing factor 3B subunit 2 OS=Homo sapiens OX=9606 GN=SF3B2 PE=1 SV=2 | 83051673 |
| Serine/arginine-rich splicing factor 3 OS=Homo sapiens OX=9606 GN=SRSF3 PE=1 SV=1 | 39467258 |
| Small nuclear ribonucleoprotein Sm D2 OS=Homo sapiens OX=9606 GN=SNRPD2 PE=1 SV=1 | 39744648 |
| 40S ribosomal protein S13 OS=Homo sapiens OX=9606 GN=RPS13 PE=1 SV=2 | 4.36E+08 |
| Cyclin-K (Fragment) OS=Homo sapiens OX=9606 GN=CCNK PE=1 SV=8 | 1655297 |
| DEAD box protein 5 (Fragment) OS=Homo sapiens OX=9606 GN=DDX5 PE=2 SV=1 | 2.62E+08 |
| Fragile X mental retardation syndrome-related protein 1 OS=Homo sapiens OX=9606 GN=FXR1 PE=1 SV=1 | 1.15E+08 |
| cDNA FLJ53704, moderately similar to Nascent polypeptide-associated complex subunit alpha OS=Homo sapiens OX=9606 PE=2 SV=1 | 9377280 |
| Activating signal cointegrator 1 complex subunit 3 OS=Homo sapiens OX=9606 GN=ASCC3 PE=1 SV=3 | 9418766 |
| Polyadenylate-binding protein 1 OS=Homo sapiens OX=9606 GN=PABPC1 PE=1 SV=1 | 1.35E+08 |
| Tubulin beta chain (Fragment) OS=Homo sapiens OX=9606 PE=2 SV=1 | 5891990 |
| TATA-binding protein-associated factor 2N OS=Homo sapiens OX=9606 GN=TAF15 PE=1 SV=1 | 1.61E+08 |
| Splicing factor 3A subunit 1 OS=Homo sapiens OX=9606 GN=SF3A1 PE=1 SV=1 | 39459550 |
| Elongin-B OS=Homo sapiens OX=9606 GN=ELOB PE=1 SV=1 | 5724863 |
| Proliferation marker protein Ki-67 OS=Homo sapiens OX=9606 GN=MKI67 PE=1 SV=2 | 6462538 |
| Probable 28S rRNA (cytosine(4447)-C(5))-methyltransferase OS=Homo sapiens OX=9606 GN=NOP2 PE=1 SV=2 | 97512808 |
| Protein S100-A8 OS=Homo sapiens OX=9606 GN=S100A8 PE=1 SV=1 | 2147229 |
| 40S ribosomal protein S11 OS=Homo sapiens OX=9606 GN=RPS11 PE=1 SV=3 | 5.79E+08 |
| Heterogeneous nuclear ribonucleoprotein A1 (Fragment) OS=Homo sapiens OX=9606 GN=HNRNPA1 PE=1 SV=1 | 1893180 |
| Poly(rC)-binding protein 2 OS=Homo sapiens OX=9606 GN=PCBP2 PE=1 SV=1 | 9031235 |
| Eukaryotic translation initiation factor 4C OS=Homo sapiens OX=9606 GN=EIF1AX PE=1 SV=1 | 4067418 |
| Chromatin target of PRMT1 protein OS=Homo sapiens OX=9606 GN=CHTOP PE=1 SV=1 | 40607240 |
| Putative RNA-binding protein Luc7-like 2 OS=Homo sapiens OX=9606 GN=LUC7L2 PE=1 SV=2 | 1.13E+08 |
| Uncharacterized protein C19orf47 OS=Homo sapiens OX=9606 GN=C19orf47 PE=1 SV=1 | 13074819 |
| Forkhead box protein Q1 OS=Homo sapiens OX=9606 GN=FOXQ1 PE=2 SV=2 | 2.2E+08 |
| Ribosomal RNA processing protein 1 homolog B OS=Homo sapiens OX=9606 GN=RRP1B PE=1 SV=3 | 29375533 |
| Dermcidin OS=Homo sapiens OX=9606 GN=DCD PE=1 SV=2 | 26495705 |
| Probable rRNA-processing protein EBP2 OS=Homo sapiens OX=9606 GN=EBNA1BP2 PE=1 SV=2 | 46858466 |
| Eukaryotic initiation factor 4A-III OS=Homo sapiens OX=9606 GN=EIF4A3 PE=1 SV=4 | 60235674 |
| 40S ribosomal protein S12 OS=Homo sapiens OX=9606 GN=RPS12 PE=1 SV=3 | 49319165 |
| Nucleolin OS=Homo sapiens OX=9606 GN=NCL PE=1 SV=3 | 4.49E+08 |
| Heterogeneous nuclear ribonucleoprotein U OS=Homo sapiens OX=9606 GN=HNRNPU PE=1 SV=6 | 1.15E+08 |
| Double-stranded RNA-binding protein Staufen homolog 1 OS=Homo sapiens OX=9606 GN=STAU1 PE=1 SV=2 | 49203392 |
| Vasoactive intestinal peptide (Fragment) OS=Homo sapiens OX=9606 GN=VIP PE=1 SV=1 | 1973378 |
| cDNA FLJ58459, highly similar to RNA-binding region-containing protein 2 OS=Homo sapiens OX=9606 PE=2 SV=1 | 1.38E+08 |
| Synaptic functional regulator FMR1 OS=Homo sapiens OX=9606 GN=FMR1 PE=2 SV=1 | 33299109 |
| Leucyl-tRNA synthetase OS=Homo sapiens OX=9606 GN=LARS1 PE=1 SV=1 | 8128345 |
| Receptor of activated protein C kinase 1 OS=Homo sapiens OX=9606 GN=RACK1 PE=1 SV=3 | 15737463 |
| Transducin beta-like protein 2 OS=Homo sapiens OX=9606 GN=TBL2 PE=1 SV=1 | 13720910 |
| Thyroid hormone receptor-associated protein 3 OS=Homo sapiens OX=9606 GN=THRAP3 PE=1 SV=2 | 73106832 |
| Multifunctional methyltransferase subunit TRM112-like protein OS=Homo sapiens OX=9606 GN=TRMT112 PE=1 SV=1 | 652257.1 |
| RNA cytidine acetyltransferase OS=Homo sapiens OX=9606 GN=NAT10 PE=1 SV=2 | 1.43E+08 |
| Guanine nucleotide-binding protein-like 3 OS=Homo sapiens OX=9606 GN=GNL3 PE=1 SV=2 | 87210830 |
| RcNSEP1 (Fragment) OS=Homo sapiens OX=9606 PE=4 SV=1 |  |
| Ubiquitin-associated protein 2-like OS=Homo sapiens OX=9606 GN=UBAP2L PE=1 SV=2 | 1.21E+08 |
| Bystin OS=Homo sapiens OX=9606 GN=BYSL PE=1 SV=3 | 11138553 |
| Serine/arginine-rich splicing factor 5 OS=Homo sapiens OX=9606 GN=SRSF5 PE=1 SV=1 | 51380649 |
| Zinc finger CCCH domain-containing protein 15 OS=Homo sapiens OX=9606 PE=2 SV=1 | 37696196 |
| 14-3-3 protein theta OS=Homo sapiens OX=9606 GN=YWHAQ PE=1 SV=1 | 16657005 |
| Insulin-like growth factor 2 mRNA-binding protein 3 OS=Homo sapiens OX=9606 GN=IGF2BP3 PE=1 SV=2 | 25722990 |
| DNA primase large subunit (Fragment) OS=Homo sapiens OX=9606 GN=PRIM2 PE=4 SV=1 | 454711.5 |
| 60S ribosomal protein L28 OS=Homo sapiens OX=9606 GN=RPL28 PE=1 SV=1 | 3.56E+08 |
| Eukaryotic translation initiation factor 3 subunit F OS=Homo sapiens OX=9606 GN=EIF3F PE=1 SV=2 | 23452997 |
| Endoplasmic reticulum chaperone BiP OS=Homo sapiens OX=9606 GN=HSPA5 PE=1 SV=2 | 8266355 |
| 40S ribosomal protein S5 OS=Homo sapiens OX=9606 GN=RPS5 PE=1 SV=1 | 2.4E+08 |
| Signal recognition particle 19 kDa protein OS=Homo sapiens OX=9606 GN=SRP19 PE=1 SV=1 | 1252648 |
| Hepatoma-derived growth factor-related protein 2 (Fragment) OS=Homo sapiens OX=9606 GN=HDGFL2 PE=1 SV=1 |  |
| Polyadenylate-binding protein OS=Homo sapiens OX=9606 GN=PABPC4 PE=2 SV=1 | 19327483 |
| RRP1 protein (Fragment) OS=Homo sapiens OX=9606 GN=RRP1 PE=2 SV=2 | 41963089 |
| U1 small nuclear ribonucleoprotein 70 kDa OS=Homo sapiens OX=9606 GN=SNRNP70 PE=1 SV=2 | 45451890 |
| 40S ribosomal protein S26 OS=Homo sapiens OX=9606 GN=RPS26 PE=3 SV=1 | 1.68E+08 |
| Peptidyl-prolyl cis-trans isomerase OS=Homo sapiens OX=9606 PE=2 SV=1 | 3543689 |
| Serine/arginine-rich splicing factor 2 (Fragment) OS=Homo sapiens OX=9606 PE=2 SV=1 | 2.27E+08 |
| cDNA FLJ77715, highly similar to Homo sapiens cisplatin resistance-associated overexpressed protein(LUC7A), mRNA OS=Homo sapiens OX=9606 PE=2 SV=1 | 35194251 |
| Exosome component 10 OS=Homo sapiens OX=9606 GN=EXOSC10 PE=1 SV=2 | 42607644 |
| 60S ribosomal protein L34 OS=Homo sapiens OX=9606 GN=RPL34 PE=1 SV=3 | 2.94E+08 |
| General transcription factor IIF subunit 1 OS=Homo sapiens OX=9606 GN=GTF2F1 PE=1 SV=2 | 15642357 |
| Leucine-rich repeat-containing protein 47 OS=Homo sapiens OX=9606 GN=LRRC47 PE=1 SV=1 | 15712148 |
| Creatine kinase U-type, mitochondrial OS=Homo sapiens OX=9606 GN=CKMT1A PE=1 SV=1 | 3.68E+08 |
| Kinesin-like protein OS=Homo sapiens OX=9606 PE=2 SV=1 | 8913110 |
| Thioredoxin OS=Homo sapiens OX=9606 GN=TXN PE=1 SV=3 | 13586948 |
| 60S ribosomal protein L31 (Fragment) OS=Homo sapiens OX=9606 GN=RPL31 PE=1 SV=1 | 2.84E+08 |
| General transcription factor 3C polypeptide 3 OS=Homo sapiens OX=9606 GN=GTF3C3 PE=1 SV=1 | 315017.7 |
| 60S ribosomal protein L8 (Fragment) OS=Homo sapiens OX=9606 GN=RPL8 PE=1 SV=1 | 6.54E+08 |
| Paraspeckle component 1 OS=Homo sapiens OX=9606 PE=2 SV=1 | 21560386 |
| cDNA, FLJ92106, highly similar to Homo sapiens adaptor-related protein complex 3, sigma 1 subunit(AP3S1), mRNA OS=Homo sapiens OX=9606 PE=2 SV=1 | 33243771 |
| UPF1 OS=Homo sapiens OX=9606 GN=UPF1 PE=2 SV=1 | 8709792 |
| ATP-dependent DNA/RNA helicase DHX36 OS=Homo sapiens OX=9606 GN=DHX36 PE=1 SV=2 | 5890121 |
| X-ray repair cross-complementing protein 5 OS=Homo sapiens OX=9606 GN=XRCC5 PE=1 SV=3 | 17345778 |
| Zinc finger protein 346 OS=Homo sapiens OX=9606 GN=ZNF346 PE=1 SV=1 | 18729762 |
| Probable ATP-dependent RNA helicase DDX23 OS=Homo sapiens OX=9606 GN=DDX23 PE=1 SV=3 | 32245310 |
| Serine/threonine-protein phosphatase PGAM5, mitochondrial OS=Homo sapiens OX=9606 GN=PGAM5 PE=1 SV=2 | 84597106 |
| rRNA adenine N(6)-methyltransferase OS=Homo sapiens OX=9606 PE=2 SV=1 | 31544394 |
| Hyaluronan mediated motility receptor OS=Homo sapiens OX=9606 GN=HMMR PE=1 SV=2 | 8388653 |
| Protein FRG1 OS=Homo sapiens OX=9606 GN=FRG1 PE=1 SV=1 | 5177622 |
| Interleukin enhancer-binding factor 3 OS=Homo sapiens OX=9606 GN=ILF3 PE=1 SV=3 | 1.27E+08 |
| Transducin beta-like protein 3 OS=Homo sapiens OX=9606 GN=TBL3 PE=1 SV=2 | 32437358 |
| 60S ribosomal protein L4 OS=Homo sapiens OX=9606 GN=RPL4 PE=1 SV=5 | 4.53E+08 |
| U6 snRNA-associated Sm-like protein LSm8 OS=Homo sapiens OX=9606 GN=LSM8 PE=1 SV=3 | 5218635 |
| Signal recognition particle subunit SRP72 OS=Homo sapiens OX=9606 GN=SRP72 PE=1 SV=3 | 28885391 |
| 28S ribosomal protein S23, mitochondrial OS=Homo sapiens OX=9606 GN=MRPS23 PE=1 SV=1 | 1272816 |
| Glyceraldehyde-3-phosphate dehydrogenase OS=Homo sapiens OX=9606 GN=GAPDH PE=1 SV=1 | 5000526 |
| 60S ribosomal protein L22-like 1 OS=Homo sapiens OX=9606 GN=RPL22L1 PE=1 SV=1 | 91248113 |
| Protein CMSS1 (Fragment) OS=Homo sapiens OX=9606 GN=CMSS1 PE=1 SV=1 | 25729172 |
| Cleavage and polyadenylation specificity factor subunit 5 OS=Homo sapiens OX=9606 PE=2 SV=1 | 34429311 |
| Nucleophosmin OS=Homo sapiens OX=9606 GN=NPM1 PE=2 SV=1 | 8.79E+08 |
| Cleavage and polyadenylation specificity factor subunit 2 OS=Homo sapiens OX=9606 GN=CPSF2 PE=1 SV=2 | 17012844 |
| Epididymis secretory sperm binding protein OS=Homo sapiens OX=9606 PE=2 SV=1 | 32182515 |
| PRRC2A OS=Homo sapiens OX=9606 PE=4 SV=1 | 97265283 |
| Formin binding protein 4, isoform CRA_d OS=Homo sapiens OX=9606 GN=FNBP4 PE=4 SV=1 | 11604200 |
| Histone H4 OS=Homo sapiens OX=9606 GN=H4C1 PE=1 SV=2 | 3.72E+08 |
| Bcl-2-associated transcription factor 1 OS=Homo sapiens OX=9606 GN=BCLAF1 PE=1 SV=2 | 92638170 |
| Arginine and glutamate-rich protein 1 OS=Homo sapiens OX=9606 GN=ARGLU1 PE=1 SV=1 | 1.05E+08 |
| Periodic tryptophan protein 2 homolog OS=Homo sapiens OX=9606 GN=PWP2 PE=2 SV=2 | 11888340 |
| Regulator of nonsense transcripts 3B OS=Homo sapiens OX=9606 GN=UPF3B PE=1 SV=1 | 17962368 |
| RNA helicase OS=Homo sapiens OX=9606 PE=2 SV=1 | 19813311 |
| Protein LSM14 homolog B OS=Homo sapiens OX=9606 GN=LSM14B PE=1 SV=1 | 6845923 |
| Ataxin-2-like protein OS=Homo sapiens OX=9606 GN=ATXN2L PE=1 SV=2 | 33833431 |
| Fusion (Involved in t(1216) in malignant liposarcoma) OS=Homo sapiens OX=9606 GN=FUS PE=2 SV=1 | 2.57E+08 |
| RFC5 protein (Fragment) OS=Homo sapiens OX=9606 GN=RFC5 PE=2 SV=1 | 9240464 |
| Lysine-rich nucleolar protein 1 OS=Homo sapiens OX=9606 GN=KNOP1 PE=1 SV=1 | 13897397 |
| Heterogeneous nuclear ribonucleoprotein A3 OS=Homo sapiens OX=9606 GN=HNRNPA3 PE=1 SV=2 | 59687218 |
| E3 ubiquitin-protein ligase TRIM21 OS=Homo sapiens OX=9606 GN=TRIM21 PE=1 SV=1 | 10569713 |
| Pleiotropic regulator 1 OS=Homo sapiens OX=9606 GN=PLRG1 PE=1 SV=1 | 26979698 |
| RNA helicase OS=Homo sapiens OX=9606 GN=DHX30 PE=1 SV=1 | 65156246 |
| cDNA FLJ36001 fis, clone TESTI2015213, highly similar to Homo sapiens target of EGR1, member 1 (nuclear) (TOE1), mRNA OS=Homo sapiens OX=9606 PE=2 SV=1 | 3889936 |
| ATP-dependent DNA helicase 2 subunit 1 OS=Homo sapiens OX=9606 GN=XRCC6 PE=1 SV=1 | 16918790 |
| RNA-binding protein EWS OS=Homo sapiens OX=9606 GN=EWSR1 PE=1 SV=1 | 3.3E+08 |
| Kinesin-like protein OS=Homo sapiens OX=9606 PE=2 SV=1 | 30127285 |
| Epididymis secretory sperm binding protein OS=Homo sapiens OX=9606 PE=2 SV=1 | 23264301 |
| Zinc finger RNA binding protein OS=Homo sapiens OX=9606 GN=ZFR PE=2 SV=1 | 11943083 |
| Protein RCC2 OS=Homo sapiens OX=9606 GN=RCC2 PE=1 SV=2 | 66421853 |
| Probable ATP-dependent RNA helicase DDX56 OS=Homo sapiens OX=9606 GN=DDX56 PE=1 SV=1 | 10225800 |
| 39S ribosomal protein L22, mitochondrial OS=Homo sapiens OX=9606 GN=MRPL22 PE=1 SV=1 | 1203534 |
| Interferon-inducible double-stranded RNA-dependent protein kinase activator A OS=Homo sapiens OX=9606 GN=PRKRA PE=1 SV=1 | 8200283 |
| Exosome complex component MTR3 OS=Homo sapiens OX=9606 GN=EXOSC6 PE=1 SV=1 | 27192438 |
| Similar to cytoskeleton-associated protein 4 (Fragment) OS=Homo sapiens OX=9606 PE=2 SV=1 | 10431228 |
| Active regulator of SIRT1 OS=Homo sapiens OX=9606 GN=RPS19BP1 PE=1 SV=1 | 771461.4 |
| 60S ribosomal protein L10a OS=Homo sapiens OX=9606 GN=RPL10A PE=1 SV=2 | 1.17E+08 |
| Focadhesin OS=Homo sapiens OX=9606 GN=FOCAD PE=1 SV=1 | 10239468 |
| G patch domain-containing protein 4 OS=Homo sapiens OX=9606 GN=GPATCH4 PE=1 SV=2 | 10526388 |
| Caprin-1 OS=Homo sapiens OX=9606 GN=CAPRIN1 PE=1 SV=2 | 2.52E+08 |
| ATP-dependent RNA helicase A OS=Homo sapiens OX=9606 GN=DHX9 PE=1 SV=4 | 1.96E+08 |
| 40S ribosomal protein S20 OS=Homo sapiens OX=9606 GN=RPS20 PE=1 SV=1 | 1.14E+08 |
| Caspase-14 OS=Homo sapiens OX=9606 GN=CASP14 PE=1 SV=2 | 4563703 |
| Probable ATP-dependent RNA helicase DDX6 OS=Homo sapiens OX=9606 GN=DDX6 PE=1 SV=2 | 18387606 |
| Ribosomal L1 domain-containing protein 1 OS=Homo sapiens OX=9606 GN=RSL1D1 PE=1 SV=3 | 27591380 |
| Splicing factor 3B subunit 1 OS=Homo sapiens OX=9606 GN=SF3B1 PE=1 SV=3 | 76060362 |
| 60S ribosomal protein L36a OS=Homo sapiens OX=9606 GN=RPL36A PE=1 SV=1 | 2.1E+08 |
| Pre-mRNA-processing factor 31 OS=Homo sapiens OX=9606 GN=PRPF31 PE=1 SV=1 | 19319105 |
| Zinc finger protein 638 OS=Homo sapiens OX=9606 GN=ZNF638 PE=1 SV=2 | 4045162 |
| Mortality factor 4-like protein 2 OS=Homo sapiens OX=9606 PE=2 SV=1 | 7028126 |
| Protein arginine N-methyltransferase 5 OS=Homo sapiens OX=9606 PE=2 SV=1 | 98020232 |
| cDNA FLJ78127, highly similar to Homo sapiens SFRS protein kinase 1 (SRPK1), mRNA OS=Homo sapiens OX=9606 PE=2 SV=1 | 38613782 |
| 40S ribosomal protein S15a OS=Homo sapiens OX=9606 GN=RPS15A PE=1 SV=1 | 3.38E+08 |
| RNA transcription, translation and transport factor protein OS=Homo sapiens OX=9606 GN=RTRAF PE=1 SV=1 | 11950222 |
| Ribosome-binding protein 1 OS=Homo sapiens OX=9606 GN=RRBP1 PE=1 SV=5 | 38510075 |
| 60S ribosomal protein L18 OS=Homo sapiens OX=9606 GN=RPL18 PE=1 SV=1 | 5.6E+08 |
| Kinesin-like protein KIFC1 OS=Homo sapiens OX=9606 GN=KIFC1 PE=1 SV=2 | 7615259 |
| U3 small nucleolar RNA-associated protein 14 homolog A OS=Homo sapiens OX=9606 GN=UTP14A PE=1 SV=1 | 18327527 |
| 39S ribosomal protein L48, mitochondrial OS=Homo sapiens OX=9606 GN=MRPL48 PE=1 SV=2 | 3155789 |
| Selenoprotein H OS=Homo sapiens OX=9606 GN=SELENOH PE=1 SV=2 | 9594971 |
| tRNA methyltransferase 10 homolog C OS=Homo sapiens OX=9606 GN=TRMT10C PE=1 SV=2 | 26176217 |
| Histone H1.2 OS=Homo sapiens OX=9606 GN=H1-2 PE=1 SV=2 | 2.55E+08 |
| 40S ribosomal protein S30 OS=Homo sapiens OX=9606 GN=FAU PE=1 SV=1 | 25002014 |
| 40S ribosomal protein S7 OS=Homo sapiens OX=9606 GN=RPS7 PE=1 SV=1 | 3.67E+08 |
| Pre-mRNA-splicing factor 3 OS=Homo sapiens OX=9606 PE=2 SV=1 | 14786998 |
| Elongation factor 2 OS=Homo sapiens OX=9606 GN=EEF2 PE=1 SV=4 | 49691614 |
| ADP/ATP translocase 2 OS=Homo sapiens OX=9606 GN=SLC25A5 PE=1 SV=7 | 38071801 |
| Annexin (Fragment) OS=Homo sapiens OX=9606 GN=ANXA2 PE=1 SV=1 | 6264594 |
| Heterogeneous nuclear ribonucleoprotein D0 (Fragment) OS=Homo sapiens OX=9606 GN=HNRNPD PE=1 SV=8 | 21187622 |
| THO complex subunit 4 OS=Homo sapiens OX=9606 GN=ALYREF PE=1 SV=1 | 1.54E+08 |
| THO complex subunit 3 OS=Homo sapiens OX=9606 GN=THOC3 PE=1 SV=1 | 2348414 |
| Importin subunit alpha-5 (Fragment) OS=Homo sapiens OX=9606 GN=KPNA1 PE=1 SV=1 | 3839699 |
| Aminoacyl tRNA synthase complex-interacting multifunctional protein 1 OS=Homo sapiens OX=9606 GN=AIMP1 PE=1 SV=2 | 6177794 |
| cDNA FLJ16777 fis, clone BRHIP2029567, highly similar to Cell division cycle 5-like protein OS=Homo sapiens OX=9606 PE=2 SV=1 | 60009008 |
| Splicing factor, arginine/serine-rich 4, isoform CRA_b OS=Homo sapiens OX=9606 GN=SFRS4 PE=2 SV=1 | 18465450 |
| Ribosomal protein L19 OS=Homo sapiens OX=9606 GN=RPL19 PE=1 SV=1 | 37573904 |
| ATP-dependent RNA helicase DDX1 OS=Homo sapiens OX=9606 GN=DDX1 PE=1 SV=1 | 59000097 |
| Elongation factor Tu OS=Homo sapiens OX=9606 GN=TUFM PE=1 SV=1 | 9866084 |
| RNA-binding protein 8A OS=Homo sapiens OX=9606 GN=RBM8A PE=1 SV=1 | 10025837 |
| Serine/arginine repetitive matrix protein 2 OS=Homo sapiens OX=9606 GN=SRRM2 PE=1 SV=2 | 73227555 |
| Nucleolar protein of 40 kDa OS=Homo sapiens OX=9606 GN=ZCCHC17 PE=1 SV=1 | 4761999 |
| STING ER exit protein OS=Homo sapiens OX=9606 GN=STEEP1 PE=1 SV=1 | 4279702 |
| Glutamate-rich WD repeat-containing protein 1 OS=Homo sapiens OX=9606 GN=GRWD1 PE=1 SV=1 | 29772792 |
| PHD finger protein 6 OS=Homo sapiens OX=9606 GN=PHF6 PE=1 SV=1 | 2.45E+08 |
| Nucleolar protein 58 OS=Homo sapiens OX=9606 GN=NOP58 PE=1 SV=1 | 10370756 |
| Cleavage and polyadenylation specificity factor subunit 7 OS=Homo sapiens OX=9606 GN=CPSF7 PE=1 SV=1 | 39250471 |
| Heterogeneous nuclear ribonucleoprotein R OS=Homo sapiens OX=9606 GN=HNRNPR PE=1 SV=1 | 15274588 |
| Lysine--tRNA ligase OS=Homo sapiens OX=9606 GN=KARS1 PE=1 SV=3 | 6211871 |
| KH domain-containing, RNA-binding, signal transduction-associated protein 1 OS=Homo sapiens OX=9606 GN=KHDRBS1 PE=1 SV=1 | 2.68E+08 |
| SRSF protein kinase 2 OS=Homo sapiens OX=9606 GN=SRPK2 PE=1 SV=3 | 13112229 |
| 40S ribosomal protein S23 OS=Homo sapiens OX=9606 GN=RPS23 PE=1 SV=3 | 28242210 |
| Importin subunit alpha-1 OS=Homo sapiens OX=9606 GN=KPNA2 PE=1 SV=1 | 20241249 |
| Fragile X mental retardation syndrome-related protein 2 OS=Homo sapiens OX=9606 GN=FXR2 PE=1 SV=2 | 21605464 |
| Vimentin OS=Homo sapiens OX=9606 GN=VIM PE=1 SV=4 | 16397972 |
| Tuftelin-interacting protein 11 OS=Homo sapiens OX=9606 GN=TFIP11 PE=2 SV=1 | 7171499 |
| Pre-mRNA-processing factor 6 OS=Homo sapiens OX=9606 GN=PRPF6 PE=1 SV=1 | 45325296 |
| Nuclear valosin-containing protein-like OS=Homo sapiens OX=9606 GN=NVL PE=1 SV=1 | 11331672 |
| Splicing factor U2AF 65 kDa subunit (Fragment) OS=Homo sapiens OX=9606 GN=U2AF2 PE=4 SV=1 | 12551994 |
| Protein LSM12 homolog OS=Homo sapiens OX=9606 GN=LSM12 PE=1 SV=1 | 14229277 |
| Eukaryotic translation initiation factor 3 subunit G (Fragment) OS=Homo sapiens OX=9606 GN=EIF3G PE=1 SV=8 | 14872192 |
| Myb-binding protein 1A (Fragment) OS=Homo sapiens OX=9606 GN=MYBBP1A PE=1 SV=1 | 1.27E+08 |
| Activating signal cointegrator 1 OS=Homo sapiens OX=9606 GN=TRIP4 PE=1 SV=1 | 5961426 |
| Sphingosine-1-phosphate lyase 1 (Fragment) OS=Homo sapiens OX=9606 GN=SGPL1 PE=1 SV=1 | 3367852 |
| L-lactate dehydrogenase B chain (Fragment) OS=Homo sapiens OX=9606 GN=LDHB PE=1 SV=1 | 378762.8 |
| Non-specific serine/threonine protein kinase OS=Homo sapiens OX=9606 GN=MARK2 PE=1 SV=2 | 17022589 |
| Solute carrier family 25, member 13 (Citrin) variant (Fragment) OS=Homo sapiens OX=9606 PE=2 SV=1 | 4256689 |
| cDNA FLJ43509 fis, clone PERIC2003699 OS=Homo sapiens OX=9606 GN=MGC40405 PE=2 SV=1 | 4581518 |
| Matrin-3 OS=Homo sapiens OX=9606 GN=MATR3 PE=1 SV=1 | 35704840 |
| 60S ribosomal protein L9 OS=Homo sapiens OX=9606 GN=RPL9 PE=1 SV=1 | 83102558 |
| ATP-binding cassette, sub-family F (GCN20), member 2 OS=Homo sapiens OX=9606 GN=ABCF2 PE=4 SV=1 | 28722028 |
| Signal recognition particle receptor subunit beta OS=Homo sapiens OX=9606 GN=SRPRB PE=1 SV=3 | 3439337 |
| Serine-threonine kinase receptor-associated protein OS=Homo sapiens OX=9606 GN=STRAP PE=1 SV=1 | 9651399 |
| cDNA, FLJ95666, highly similar to Homo sapiens albumin (ALB), mRNA OS=Homo sapiens OX=9606 PE=2 SV=1 | 1.23E+08 |
| ATP-dependent RNA helicase DDX24 OS=Homo sapiens OX=9606 GN=DDX24 PE=1 SV=1 | 12326143 |
| RNA exonuclease 4 OS=Homo sapiens OX=9606 GN=REXO4 PE=1 SV=2 | 8474257 |
| Serine/threonine-protein kinase RIO1 OS=Homo sapiens OX=9606 GN=RIOK1 PE=1 SV=2 | 39678303 |
| FACT complex subunit SSRP1 OS=Homo sapiens OX=9606 GN=SSRP1 PE=1 SV=1 | 15341088 |
| N-acylneuraminate cytidylyltransferase OS=Homo sapiens OX=9606 GN=CMAS PE=1 SV=2 | 7585986 |
| Protein LSM14 homolog A OS=Homo sapiens OX=9606 GN=LSM14A PE=1 SV=3 | 27300425 |
| Zinc finger CCCH-type antiviral protein 1 OS=Homo sapiens OX=9606 GN=ZC3HAV1 PE=1 SV=3 | 16657088 |
| Histone H2A.V OS=Homo sapiens OX=9606 GN=H2AZ2 PE=1 SV=3 | 3822796 |
| cDNA FLJ27420 fis, clone WMC07143 OS=Homo sapiens OX=9606 PE=2 SV=1 | 942531.6 |
| Pre-mRNA 3'-end-processing factor FIP1 OS=Homo sapiens OX=9606 GN=FIP1L1 PE=1 SV=1 | 25770760 |
| Splicing factor, suppressor of white-apricot homolog OS=Homo sapiens OX=9606 GN=SFSWAP PE=1 SV=3 | 7263285 |
| AP complex subunit beta OS=Homo sapiens OX=9606 PE=2 SV=1 | 6958760 |
| WD40 repeat-containing protein SMU1 OS=Homo sapiens OX=9606 GN=SMU1 PE=1 SV=2 | 7590545 |
| Small nuclear ribonucleoprotein Sm D3 OS=Homo sapiens OX=9606 GN=SNRPD3 PE=1 SV=1 | 82774322 |
| ATP synthase subunit O, mitochondrial OS=Homo sapiens OX=9606 GN=ATP5PO PE=1 SV=1 | 1873094 |
| 40S ribosomal protein S10 OS=Homo sapiens OX=9606 GN=RPS10 PE=1 SV=1 | 85855254 |
| U5 small nuclear ribonucleoprotein 200 kDa helicase OS=Homo sapiens OX=9606 GN=SNRNP200 PE=1 SV=2 | 81803167 |
| Glutathione peroxidase (Fragment) OS=Homo sapiens OX=9606 GN=GPX4 PE=1 SV=1 | 1847291 |
| Histone deacetylase complex subunit SAP18 (Fragment) OS=Homo sapiens OX=9606 GN=SAP18 PE=1 SV=2 | 11967596 |
| AP-3 complex subunit sigma-2 OS=Homo sapiens OX=9606 GN=AP3S2 PE=1 SV=1 | 2374462 |
| Heterogeneous nuclear ribonucleoprotein H OS=Homo sapiens OX=9606 GN=HNRNPH1 PE=1 SV=1 | 20215865 |
| Regulator of nonsense transcripts 2 OS=Homo sapiens OX=9606 GN=UPF2 PE=1 SV=1 | 5620657 |
| Trifunctional enzyme subunit beta, mitochondrial OS=Homo sapiens OX=9606 GN=HADHB PE=1 SV=1 | 5813285 |
| Eukaryotic translation initiation factor 3 subunit B OS=Homo sapiens OX=9606 GN=EIF3B PE=2 SV=1 | 24880807 |
| cDNA FLJ58196, highly similar to Zinc finger CCCH domain-containing protein 11A OS=Homo sapiens OX=9606 PE=2 SV=1 | 6321235 |
| HBS1-like protein OS=Homo sapiens OX=9606 PE=2 SV=1 | 9006701 |
| cDNA, FLJ94450, highly similar to Homo sapiens cyclin-dependent kinase 9 (CDC2-related kinase) (CDK9), mRNA OS=Homo sapiens OX=9606 PE=2 SV=1 | 67190777 |
| RNA-binding protein PNO1 OS=Homo sapiens OX=9606 PE=2 SV=1 | 11242029 |
| Non-muscle myosin heavy chain 9 OS=Homo sapiens OX=9606 GN=MYH9 PE=2 SV=1 | 2044199 |
| Ribosomal RNA-processing protein 4 OS=Homo sapiens OX=9606 GN=EXOSC2 PE=1 SV=1 | 8113976 |
| 60 kDa chaperonin OS=Homo sapiens OX=9606 GN=HSPD1 PE=1 SV=1 | 14301925 |
| RNA helicase OS=Homo sapiens OX=9606 GN=DDX46 PE=1 SV=1 | 25232601 |
| S1 RNA-binding domain-containing protein 1 OS=Homo sapiens OX=9606 GN=SRBD1 PE=1 SV=2 | 5437982 |
| Ras-GTPase activating protein SH3 domain-binding protein 2, isoform CRA_b OS=Homo sapiens OX=9606 GN=G3BP2 PE=4 SV=1 | 81615370 |
| 60S ribosomal protein L29 OS=Homo sapiens OX=9606 GN=RPL29 PE=1 SV=1 | 1.3E+08 |
| Structural maintenance of chromosomes protein OS=Homo sapiens OX=9606 GN=SMC3 PE=2 SV=1 | 3334762 |
| Pre-mRNA-processing factor 19 OS=Homo sapiens OX=9606 GN=PRPF19 PE=1 SV=1 | 1.01E+08 |
| ATPase family AAA domain-containing protein 3A OS=Homo sapiens OX=9606 GN=ATAD3A PE=1 SV=2 | 57487043 |
| Phenylalanine--tRNA ligase beta subunit OS=Homo sapiens OX=9606 GN=FARSB PE=1 SV=3 | 18829751 |
| G patch domain-containing protein 8 OS=Homo sapiens OX=9606 GN=GPATCH8 PE=1 SV=2 | 5879053 |
| rRNA-processing protein UTP23 homolog OS=Homo sapiens OX=9606 GN=UTP23 PE=1 SV=2 | 5348905 |
| H/ACA ribonucleoprotein complex subunit DKC1 OS=Homo sapiens OX=9606 GN=DKC1 PE=1 SV=3 | 16953194 |
| TOB3 OS=Homo sapiens OX=9606 PE=2 SV=1 | 1255008 |
| U5 small nuclear ribonucleoprotein 40 kDa protein OS=Homo sapiens OX=9606 GN=SNRNP40 PE=1 SV=1 | 6983519 |
| Nuclear fragile X mental retardation-interacting protein 2 OS=Homo sapiens OX=9606 GN=NUFIP2 PE=1 SV=1 | 12935299 |
| Double-stranded RNA-specific adenosine deaminase (Fragment) OS=Homo sapiens OX=9606 GN=ADAR PE=1 SV=1 | 15665017 |
| Glutathione peroxidase OS=Homo sapiens OX=9606 GN=GPX1 PE=2 SV=1 | 1514665 |
| Probable ATP-dependent RNA helicase DHX37 OS=Homo sapiens OX=9606 GN=DHX37 PE=1 SV=1 | 5374435 |
| Replication factor C 3 isoform 1 variant (Fragment) OS=Homo sapiens OX=9606 PE=2 SV=1 | 24622548 |
| SNW domain-containing protein 1 OS=Homo sapiens OX=9606 GN=SNW1 PE=1 SV=1 | 10750770 |
| RNA cytosine C(5)-methyltransferase NSUN2 OS=Homo sapiens OX=9606 GN=NSUN2 PE=1 SV=2 | 11675030 |
| ATP synthase subunit e, mitochondrial OS=Homo sapiens OX=9606 GN=ATP5ME PE=1 SV=2 |  |
| Replication factor C subunit 4 OS=Homo sapiens OX=9606 GN=RFC4 PE=1 SV=2 | 10254149 |
| Histone H1.0 OS=Homo sapiens OX=9606 GN=H1-0 PE=1 SV=3 | 8434504 |
| Surfeit locus protein 6 OS=Homo sapiens OX=9606 GN=SURF6 PE=1 SV=3 | 8951271 |
| Activator of basal transcription 1 OS=Homo sapiens OX=9606 GN=ABT1 PE=1 SV=1 | 6685696 |
| Insulin receptor substrate 4 OS=Homo sapiens OX=9606 GN=IRS4 PE=1 SV=1 | 49923089 |
| Phenylalanine--tRNA ligase OS=Homo sapiens OX=9606 GN=FARSA PE=1 SV=1 | 29549616 |
| RNA helicase (Fragment) OS=Homo sapiens OX=9606 PE=2 SV=1 | 8250347 |
| Eukaryotic translation initiation factor 4 gamma 1 OS=Homo sapiens OX=9606 GN=EIF4G1 PE=1 SV=1 | 44717752 |
| Tubulin alpha chain (Fragment) OS=Homo sapiens OX=9606 GN=TUBA8 PE=1 SV=1 | 9068682 |
| cDNA, FLJ94136, highly similar to Homo sapiens synaptotagmin binding, cytoplasmic RNA interacting protein (SYNCRIP), mRNA OS=Homo sapiens OX=9606 PE=2 SV=1 | 20492822 |
| cDNA, FLJ93619, highly similar to Homo sapiens PRP4 pre-mRNA processing factor 4 homolog (yeast) (PRPF4), mRNA OS=Homo sapiens OX=9606 PE=2 SV=1 | 15069546 |
| Ribosomal protein L27a OS=Homo sapiens OX=9606 GN=L27a PE=4 SV=1 | 3.78E+08 |
| Dolichyl-diphosphooligosaccharide--protein glycosyltransferase subunit 1 OS=Homo sapiens OX=9606 GN=RPN1 PE=1 SV=1 | 2485774 |
| RRP15-like protein OS=Homo sapiens OX=9606 PE=2 SV=1 | 2770650 |
| rRNA adenine N(6)-methyltransferase OS=Homo sapiens OX=9606 PE=2 SV=1 | 5094004 |
| Cleavage and polyadenylation specificity factor subunit 4 OS=Homo sapiens OX=9606 GN=CPSF4 PE=1 SV=1 | 5832056 |
| RNA-binding protein 15 OS=Homo sapiens OX=9606 GN=RBM15 PE=1 SV=1 | 18611967 |
| Serine/arginine repetitive matrix 1 isoform 2 (Fragment) OS=Homo sapiens OX=9606 GN=SRRM1 PE=1 SV=1 | 61060015 |
| U3 small nucleolar RNA-associated protein 18 homolog OS=Homo sapiens OX=9606 GN=UTP18 PE=1 SV=3 | 20229998 |
| FACT complex subunit SPT16 OS=Homo sapiens OX=9606 GN=SUPT16H PE=1 SV=1 | 33506633 |
| Eukaryotic translation initiation factor 3 subunit L OS=Homo sapiens OX=9606 GN=EIF3L PE=1 SV=1 | 36149522 |
| RNA-binding protein 27 OS=Homo sapiens OX=9606 GN=RBM27 PE=1 SV=2 | 1.31E+08 |
| p21-activated protein kinase-interacting protein 1 OS=Homo sapiens OX=9606 GN=PAK1IP1 PE=1 SV=2 | 9476255 |
| U3 small nucleolar ribonucleoprotein protein IMP3 OS=Homo sapiens OX=9606 GN=IMP3 PE=1 SV=1 | 1605537 |
| Tubulin alpha chain OS=Homo sapiens OX=9606 GN=TUBA1C PE=1 SV=1 | 65275335 |
| Replication factor C subunit 2 OS=Homo sapiens OX=9606 GN=RFC2 PE=1 SV=3 | 12700630 |
| pre-rRNA 2'-O-ribose RNA methyltransferase FTSJ3 OS=Homo sapiens OX=9606 GN=FTSJ3 PE=1 SV=2 | 16127602 |
| RNA helicase (Fragment) OS=Homo sapiens OX=9606 GN=DDX10 PE=2 SV=1 | 60132063 |
| RNA-binding protein 26 OS=Homo sapiens OX=9606 GN=RBM26 PE=1 SV=3 | 22797808 |
| Serine/arginine-rich-splicing factor 11 (Fragment) OS=Homo sapiens OX=9606 GN=SRSF11 PE=1 SV=1 | 10433739 |
| Splicing factor 45 OS=Homo sapiens OX=9606 GN=RBM17 PE=1 SV=1 | 16699757 |
| WD repeat-containing protein 82 OS=Homo sapiens OX=9606 GN=WDR82 PE=1 SV=1 | 13921232 |
| Small nuclear ribonucleoprotein-associated protein OS=Homo sapiens OX=9606 GN=SNRPB PE=2 SV=1 | 52158526 |
| Ribosome biogenesis regulatory protein homolog OS=Homo sapiens OX=9606 GN=RRS1 PE=1 SV=2 | 20075425 |
| Cold-inducible RNA-binding protein OS=Homo sapiens OX=9606 GN=CIRBP PE=1 SV=1 | 2887836 |
| Splicing factor U2AF 35 kDa subunit OS=Homo sapiens OX=9606 GN=U2AF1 PE=1 SV=3 | 38445443 |
| 14-3-3 protein zeta/delta OS=Homo sapiens OX=9606 GN=YWHAZ PE=1 SV=1 | 1105577 |
| 40S ribosomal protein S29 OS=Homo sapiens OX=9606 GN=RPS29 PE=1 SV=2 | 6419867 |
| Arginine--tRNA ligase, cytoplasmic OS=Homo sapiens OX=9606 GN=RARS1 PE=1 SV=2 | 11972488 |
| RuvB-like 2 OS=Homo sapiens OX=9606 GN=RUVBL2 PE=1 SV=3 | 3116062 |
| 40S ribosomal protein S19 OS=Homo sapiens OX=9606 GN=RPS19 PE=1 SV=2 | 1.13E+08 |
| Lipocalin-1 OS=Homo sapiens OX=9606 GN=LCN1 PE=1 SV=1 | 1229321 |
| Microtubule-associated protein 4 OS=Homo sapiens OX=9606 GN=MAP4 PE=1 SV=3 | 20407101 |
| La-related protein 7 OS=Homo sapiens OX=9606 GN=LARP7 PE=1 SV=1 | 8687128 |
| Cyclin-dependent kinase 11B OS=Homo sapiens OX=9606 GN=CDK11B PE=1 SV=4 | 58523467 |
| Alpha-taxilin OS=Homo sapiens OX=9606 GN=TXLNA PE=1 SV=3 | 7884277 |
| Eukaryotic translation initiation factor 2A OS=Homo sapiens OX=9606 GN=EIF2A PE=1 SV=3 | 2465621 |
| Heat shock protein HSP 90-beta OS=Homo sapiens OX=9606 GN=HSP90AB1 PE=1 SV=4 | 15810654 |
| Zinc finger protein 768 OS=Homo sapiens OX=9606 GN=ZNF768 PE=1 SV=2 | 7778702 |
| Uncharacterized protein OS=Homo sapiens OX=9606 PE=4 SV=1 | 1178725 |
| EEF1E1-BLOC1S5 readthrough (NMD candidate) OS=Homo sapiens OX=9606 GN=EEF1E1-BLOC1S5 PE=4 SV=2 | 2267919 |
| Calcium load-activated calcium channel OS=Homo sapiens OX=9606 GN=TMCO1 PE=2 SV=1 | 4035405 |
| Pescadillo homolog OS=Homo sapiens OX=9606 GN=PES1 PE=1 SV=1 | 14966470 |
| 5'-3' exoribonuclease OS=Homo sapiens OX=9606 PE=2 SV=1 | 25341816 |
| 116 kDa U5 small nuclear ribonucleoprotein component OS=Homo sapiens OX=9606 PE=2 SV=1 | 41005201 |
| 3-methyladenine DNA glycosidase (Fragment) OS=Homo sapiens OX=9606 GN=MPG PE=1 SV=1 | 949747.9 |
| cDNA, FLJ94025, highly similar to Homo sapiens tripartite motif-containing 28 (TRIM28), mRNA OS=Homo sapiens OX=9606 PE=2 SV=1 | 7187091 |
| Ubiquitin carboxyl-terminal hydrolase OS=Homo sapiens OX=9606 PE=2 SV=1 | 33870120 |
| RNA-binding protein 10 OS=Homo sapiens OX=9606 GN=RBM10 PE=1 SV=3 | 6775943 |
| Guanine nucleotide binding protein-like 3 (Nucleolar)-like, isoform CRA_b OS=Homo sapiens OX=9606 GN=GNL3L PE=1 SV=1 | 4102012 |
| IG c473_heavy_IGHV3-7_IGHD5-12_IGHJ3 (Fragment) OS=Homo sapiens OX=9606 PE=2 SV=1 | 914856.6 |
| GTP-binding protein 1 OS=Homo sapiens OX=9606 GN=GTPBP1 PE=4 SV=1 | 19583384 |
| Lamina-associated polypeptide 2, isoforms beta/gamma OS=Homo sapiens OX=9606 GN=TMPO PE=1 SV=2 | 14016352 |
| Insulin-like growth factor 2 mRNA-binding protein 2 OS=Homo sapiens OX=9606 GN=IGF2BP2 PE=1 SV=2 | 6923238 |
| U3 small nucleolar RNA-associated protein 15 homolog OS=Homo sapiens OX=9606 GN=UTP15 PE=1 SV=3 | 4836607 |
| 28S rRNA (cytosine-C(5))-methyltransferase OS=Homo sapiens OX=9606 GN=NSUN5 PE=1 SV=2 | 9243545 |
| Protein LTV1 homolog OS=Homo sapiens OX=9606 GN=LTV1 PE=1 SV=1 | 12137134 |
| Neuroguidin OS=Homo sapiens OX=9606 GN=NGDN PE=1 SV=1 | 3527659 |
| La-related protein 4 OS=Homo sapiens OX=9606 GN=LARP4 PE=1 SV=3 | 7293776 |
| La-related protein 1 OS=Homo sapiens OX=9606 GN=LARP1 PE=1 SV=2 | 51531165 |
| Putative ATP-dependent RNA helicase DHX57 OS=Homo sapiens OX=9606 GN=DHX57 PE=1 SV=2 | 20771364 |
| Lysine-specific demethylase 9 OS=Homo sapiens OX=9606 GN=RSBN1 PE=1 SV=2 | 8664455 |
| Histone H2A type 2-C OS=Homo sapiens OX=9606 GN=H2AC20 PE=1 SV=4 | 1.8E+08 |
| Aminoacyl tRNA synthase complex-interacting multifunctional protein 2 OS=Homo sapiens OX=9606 GN=AIMP2 PE=1 SV=2 | 3061325 |
| Desmoglein-1 OS=Homo sapiens OX=9606 GN=DSG1 PE=1 SV=2 | 10079696 |
| Glutaminyl-tRNA synthetase OS=Homo sapiens OX=9606 PE=2 SV=1 | 7761982 |
| U6 snRNA-associated Sm-like protein LSm3 OS=Homo sapiens OX=9606 GN=LSM3 PE=1 SV=2 | 3107552 |
| Aspartyl/asparaginyl beta-hydroxylase OS=Homo sapiens OX=9606 GN=ASPH PE=1 SV=3 | 6653085 |
| cDNA FLJ78252, highly similar to Homo sapiens heterogeneous nuclear ribonucleoprotein U-like 1 (HNRPUL1), transcript variant 1, mRNA OS=Homo sapiens OX=9606 PE=2 SV=1 | 11043639 |
| DNA topoisomerase OS=Homo sapiens OX=9606 PE=2 SV=1 | 8206406 |
| Heterogeneous nuclear ribonucleoprotein H2 OS=Homo sapiens OX=9606 GN=HNRNPH2 PE=1 SV=1 | 799069.4 |
| Structural maintenance of chromosomes protein 1A OS=Homo sapiens OX=9606 GN=SMC1A PE=1 SV=2 | 3669098 |
| Serine/threonine-protein phosphatase PP1-gamma catalytic subunit OS=Homo sapiens OX=9606 GN=PPP1CC PE=1 SV=1 | 5086538 |
| 14-3-3 protein beta/alpha OS=Homo sapiens OX=9606 GN=YWHAB PE=1 SV=3 | 541127.9 |
| U2 small nuclear ribonucleoprotein B'' OS=Homo sapiens OX=9606 GN=SNRPB2 PE=1 SV=1 | 9172177 |
| TBC1 domain family member 10B OS=Homo sapiens OX=9606 GN=TBC1D10B PE=1 SV=3 | 8323008 |
| Cold shock domain-containing protein E1 OS=Homo sapiens OX=9606 GN=CSDE1 PE=1 SV=2 | 16678534 |
| Pre-mRNA-processing factor 40 homolog A OS=Homo sapiens OX=9606 GN=PRPF40A PE=1 SV=2 | 1.21E+08 |
| cDNA FLJ59571, highly similar to Eukaryotic translation initiation factor 4gamma 2 OS=Homo sapiens OX=9606 PE=2 SV=1 | 3499666 |
| Protein virilizer homolog OS=Homo sapiens OX=9606 GN=VIRMA PE=1 SV=2 | 3994236 |
| Nucleolar protein 56 OS=Homo sapiens OX=9606 GN=NOP56 PE=1 SV=4 | 9752749 |
| 60S ribosomal protein L22 (Fragment) OS=Homo sapiens OX=9606 GN=RPL22 PE=1 SV=1 | 36401244 |
| Protein piccolo (Fragment) OS=Homo sapiens OX=9606 GN=PCLO PE=1 SV=1 | 28069318 |
| Hepatocellular carcinoma related protein 2 OS=Homo sapiens OX=9606 PE=2 SV=1 | 9757073 |
| E3 ubiquitin-protein ligase ZNF598 OS=Homo sapiens OX=9606 GN=ZNF598 PE=1 SV=1 | 14120609 |
| S-phase kinase-associated protein 1 OS=Homo sapiens OX=9606 GN=SKP1 PE=1 SV=1 | 2840488 |
| DNA (cytosine-5)-methyltransferase 1 (Fragment) OS=Homo sapiens OX=9606 PE=2 SV=1 | 4837548 |
| cDNA FLJ51284, highly similar to Adapter-relatedprotein complex 3 mu-1 subunit OS=Homo sapiens OX=9606 PE=2 SV=1 | 2308277 |
| RNA helicase OS=Homo sapiens OX=9606 PE=2 SV=1 | 11616886 |
| GRB10-interacting GYF protein 2 OS=Homo sapiens OX=9606 GN=GIGYF2 PE=1 SV=1 | 10168471 |
| DNA helicase OS=Homo sapiens OX=9606 GN=CHD4 PE=1 SV=1 | 4953133 |
| Protein SON OS=Homo sapiens OX=9606 GN=SON PE=1 SV=4 | 14862555 |
| Uncharacterized protein OS=Homo sapiens OX=9606 PE=3 SV=1 | 1290379 |
| RNA helicase OS=Homo sapiens OX=9606 PE=2 SV=1 | 14415911 |
| IBM-B1 light chain variable region (Fragment) OS=Homo sapiens OX=9606 PE=2 SV=1 | 569232.5 |
| RNA-binding protein OS=Homo sapiens OX=9606 PE=2 SV=1 | 7430040 |
| WD repeat-containing protein 3 OS=Homo sapiens OX=9606 GN=WDR3 PE=1 SV=1 | 43436195 |
| Splicing factor, arginine/serine-rich 15 OS=Homo sapiens OX=9606 GN=SFRS15 PE=2 SV=1 | 6489401 |
| Vigilin OS=Homo sapiens OX=9606 GN=HDLBP PE=1 SV=2 | 4908009 |
| Keratin, type II cytoskeletal 78 OS=Homo sapiens OX=9606 GN=KRT78 PE=1 SV=2 | 2691621 |
| 75 kDa glucose-regulated protein (Fragment) OS=Homo sapiens OX=9606 GN=HSPA9 PE=2 SV=1 | 13379776 |
| RRP12-like protein OS=Homo sapiens OX=9606 GN=RRP12 PE=1 SV=2 | 37992379 |
| Eukaryotic translation initiation factor 3, subunit 3 gamma, 40kDa variant (Fragment) OS=Homo sapiens OX=9606 PE=2 SV=1 | 8210203 |
| Leucine-rich repeat-containing protein 59 OS=Homo sapiens OX=9606 GN=LRRC59 PE=1 SV=1 | 41724629 |
| Eukaryotic translation initiation factor 3 subunit I OS=Homo sapiens OX=9606 GN=EIF3I PE=1 SV=1 | 13283074 |
| Cilia- and flagella-associated protein 20 OS=Homo sapiens OX=9606 GN=CFAP20 PE=1 SV=1 | 11674711 |
| Brix domain-containing protein 2 OS=Homo sapiens OX=9606 GN=BXDC2 PE=2 SV=1 | 22384743 |
| Eukaryotic translation initiation factor 5A (Fragment) OS=Homo sapiens OX=9606 GN=EIF5A PE=1 SV=8 | 20625730 |
| Actin-related protein 2/3 complex subunit 4 OS=Homo sapiens OX=9606 GN=ARPC4 PE=1 SV=3 | 488010.5 |
| Eukaryotic translation initiation factor 6 OS=Homo sapiens OX=9606 GN=EIF6 PE=1 SV=1 | 3691622 |
| 60S ribosomal protein L21 (Fragment) OS=Homo sapiens OX=9606 PE=2 SV=1 | 1.86E+08 |
| Protein-L-isoaspartate(D-aspartate) O-methyltransferase OS=Homo sapiens OX=9606 GN=PCMT1 PE=1 SV=4 | 4681546 |
| Junction plakoglobin OS=Homo sapiens OX=9606 GN=JUP PE=1 SV=3 | 7087214 |
| 60S ribosomal protein L11 OS=Homo sapiens OX=9606 GN=RPL11 PE=1 SV=2 | 4.41E+08 |
| Cyclin-dependent kinase 1 OS=Homo sapiens OX=9606 GN=CDK1 PE=1 SV=3 | 3174630 |
| Y-box-binding protein 3 OS=Homo sapiens OX=9606 GN=YBX3 PE=1 SV=4 | 10434134 |
| Thioredoxin-like protein 4A OS=Homo sapiens OX=9606 GN=TXNL4A PE=1 SV=1 | 446648.3 |
| Heparan sulfate 2-O-sulfotransferase 1 (Fragment) OS=Homo sapiens OX=9606 GN=HS2ST1 PE=1 SV=1 |  |
| Beta-arrestin-2 (Fragment) OS=Homo sapiens OX=9606 GN=ARRB2 PE=1 SV=1 | 751490.8 |
| Eukaryotic translation initiation factor 3 subunit A OS=Homo sapiens OX=9606 GN=eIF3a PE=2 SV=1 | 44030871 |
| Mothers against decapentaplegic homolog 3 (Fragment) OS=Homo sapiens OX=9606 GN=SMAD3 PE=1 SV=1 | 879288.6 |
| Nucleolar protein 16 OS=Homo sapiens OX=9606 GN=NOP16 PE=1 SV=2 | 7618836 |
| Thyroid transcription factor 1-associated protein 26 (Fragment) OS=Homo sapiens OX=9606 GN=CCDC59 PE=1 SV=1 | 1881094 |
| 60S ribosome subunit biogenesis protein NIP7 homolog OS=Homo sapiens OX=9606 GN=NIP7 PE=1 SV=1 | 22184159 |
| Serine/arginine-rich splicing factor 7 OS=Homo sapiens OX=9606 GN=SRSF7 PE=1 SV=1 | 76200072 |
| Staphylococcal nuclease domain-containing protein OS=Homo sapiens OX=9606 PE=2 SV=1 | 10323446 |
| Light chain variable region (Fragment) OS=Homo sapiens OX=9606 PE=2 SV=1 |  |
| U6 snRNA-associated Sm-like protein LSm4 (Fragment) OS=Homo sapiens OX=9606 GN=LSM4 PE=1 SV=1 | 10904282 |
| UPF0488 protein C8orf33 OS=Homo sapiens OX=9606 GN=C8orf33 PE=1 SV=1 | 22886927 |
| rRNA methyltransferase 3, mitochondrial OS=Homo sapiens OX=9606 GN=MRM3 PE=1 SV=2 | 12956793 |
| 3'-5' RNA helicase YTHDC2 OS=Homo sapiens OX=9606 GN=YTHDC2 PE=1 SV=2 | 21176138 |
| 39S ribosomal protein L46, mitochondrial OS=Homo sapiens OX=9606 GN=MRPL46 PE=1 SV=1 | 3138050 |
| Microsomal glutathione S-transferase 2 OS=Homo sapiens OX=9606 GN=MGST2 PE=1 SV=1 | 715973.3 |
| Processing of 1, ribonuclease P/MRP subunit (S. cerevisiae) OS=Homo sapiens OX=9606 GN=POP1 PE=2 SV=1 | 22645046 |
| 28S ribosomal protein S26, mitochondrial OS=Homo sapiens OX=9606 GN=MRPS26 PE=1 SV=1 | 9908198 |
| ATP-dependent RNA helicase DDX54 OS=Homo sapiens OX=9606 GN=DDX54 PE=1 SV=2 | 28253817 |
| THO complex subunit 6 homolog OS=Homo sapiens OX=9606 GN=THOC6 PE=1 SV=1 | 3928978 |
| Nucleolar MIF4G domain-containing protein 1 OS=Homo sapiens OX=9606 GN=NOM1 PE=1 SV=1 | 8705146 |
| Methylosome protein 50 OS=Homo sapiens OX=9606 GN=WDR77 PE=1 SV=1 | 21591929 |
| Heterogeneous nuclear ribonucleoprotein H3 isoform a variant (Fragment) OS=Homo sapiens OX=9606 PE=2 SV=1 | 6478066 |
| CD3EAP protein (Fragment) OS=Homo sapiens OX=9606 GN=CD3EAP PE=2 SV=1 | 3315228 |
| Zinc finger CCHC domain-containing protein 3 OS=Homo sapiens OX=9606 GN=ZCCHC3 PE=1 SV=2 | 5563325 |
| Pre-mRNA-splicing regulator WTAP OS=Homo sapiens OX=9606 GN=WTAP PE=1 SV=2 | 3777316 |
| Single-stranded DNA-binding protein, mitochondrial OS=Homo sapiens OX=9606 GN=SSBP1 PE=1 SV=1 | 1765540 |
| Heterogeneous nuclear ribonucleoprotein F OS=Homo sapiens OX=9606 GN=HNRNPF PE=1 SV=3 | 8603477 |
| Exosome complex component RRP45 OS=Homo sapiens OX=9606 GN=EXOSC9 PE=1 SV=3 | 8524117 |
| Elongation factor 1-gamma OS=Homo sapiens OX=9606 GN=EEF1G PE=1 SV=3 | 4903804 |
| ADP/ATP translocase 3 OS=Homo sapiens OX=9606 GN=SLC25A6 PE=1 SV=4 | 6387036 |
| Heat shock protein HSP 90-alpha OS=Homo sapiens OX=9606 GN=HSP90AA1 PE=1 SV=5 | 11241480 |
| DnaJ homolog subfamily A member 1 OS=Homo sapiens OX=9606 GN=DNAJA1 PE=1 SV=2 | 2714751 |
| SAP30-binding protein (Fragment) OS=Homo sapiens OX=9606 GN=SAP30BP PE=1 SV=1 | 2880937 |
| YWHAE/FAM22B fusion protein (Fragment) OS=Homo sapiens OX=9606 GN=YWHAE/FAM22B fusion PE=2 SV=1 | 7606079 |
| Cleavage and polyadenylation specificity factor subunit 6 OS=Homo sapiens OX=9606 GN=CPSF6 PE=1 SV=1 | 12364713 |
| Cyclic AMP-dependent transcription factor ATF-2 OS=Homo sapiens OX=9606 GN=ATF2 PE=1 SV=1 | 3773764 |
| Histone-binding protein RBBP7 OS=Homo sapiens OX=9606 GN=RBBP7 PE=1 SV=1 | 4284841 |
| Plasma membrane citrate carrier OS=Homo sapiens OX=9606 GN=SLC25A1 PE=2 SV=1 | 3045096 |
| RuvB-like helicase (Fragment) OS=Homo sapiens OX=9606 GN=RUVBL1 PE=2 SV=1 | 4997209 |
| High glucose-regulated protein 8 OS=Homo sapiens OX=9606 GN=YTHDF2 PE=2 SV=1 | 7232525 |
| U2 snRNP auxiliary factor large subunit OS=Homo sapiens OX=9606 GN=U2AF2 PE=2 SV=1 | 35408121 |
| cDNA FLJ60148, highly similar to Homo sapiens heterogeneous nuclear ribonucleoprotein D-like (HNRPDL), transcript variant 2, mRNA OS=Homo sapiens OX=9606 PE=2 SV=1 | 1121098 |
| Nucleolar protein 10 OS=Homo sapiens OX=9606 GN=NOL10 PE=1 SV=1 | 4073845 |
| cDNA FLJ34439 fis, clone HLUNG2001146, highly similar to Splicing factor, arginine/serine-rich 12 OS=Homo sapiens OX=9606 PE=2 SV=1 | 6775531 |
| cDNA, FLJ93871, highly similar to Homo sapiens melanoma antigen, family B, 2 (MAGEB2), mRNA OS=Homo sapiens OX=9606 PE=2 SV=1 | 14674323 |
| Protein arginine N-methyltransferase 3 OS=Homo sapiens OX=9606 GN=PRMT3 PE=1 SV=4 | 1812550 |
| Uncharacterized protein DKFZp686E1893 OS=Homo sapiens OX=9606 GN=DKFZp686E1893 PE=2 SV=1 | 2134004 |
| cDNA FLJ77771, highly similar to Homo sapiens replication initiator 1, mRNA OS=Homo sapiens OX=9606 PE=2 SV=1 | 3889111 |
| TRMT1-like protein OS=Homo sapiens OX=9606 GN=TRMT1L PE=1 SV=2 | 5108615 |
| IGL c1933_light_IGKV3-11_IGKJ4 (Fragment) OS=Homo sapiens OX=9606 PE=2 SV=1 | 2.8E+08 |
| cDNA FLJ61517, highly similar to Homo sapiens ubiquitin associated protein 2 (UBAP2), transcript variant 1, mRNA OS=Homo sapiens OX=9606 PE=2 SV=1 | 4819100 |
| Casein kinase I isoform alpha OS=Homo sapiens OX=9606 GN=CSNK1A1 PE=1 SV=2 | 1844111 |
| Protein PRRC2C OS=Homo sapiens OX=9606 GN=PRRC2C PE=1 SV=4 | 44979105 |
| RNA 3'-terminal phosphate cyclase-like protein OS=Homo sapiens OX=9606 GN=RCL1 PE=1 SV=3 | 2624936 |
| Protein argonaute-2 OS=Homo sapiens OX=9606 GN=AGO2 PE=1 SV=3 | 4278854 |
| Probable ribosome biogenesis protein RLP24 OS=Homo sapiens OX=9606 GN=RSL24D1 PE=1 SV=1 | 410825.2 |
| La-related protein 4B OS=Homo sapiens OX=9606 GN=LARP4B PE=1 SV=3 | 4929645 |
| U3 small nucleolar RNA-associated protein 25 homolog OS=Homo sapiens OX=9606 GN=UTP25 PE=1 SV=2 | 2733874 |
| 39S ribosomal protein L40, mitochondrial OS=Homo sapiens OX=9606 GN=MRPL40 PE=1 SV=1 | 383398.1 |
| Exosome complex component RRP41 OS=Homo sapiens OX=9606 GN=EXOSC4 PE=1 SV=3 | 16542912 |
| Large subunit GTPase 1 homolog OS=Homo sapiens OX=9606 GN=LSG1 PE=1 SV=2 | 21799057 |
| Leucine zipper protein 1 OS=Homo sapiens OX=9606 GN=LUZP1 PE=1 SV=2 | 2161977 |
| Nucleolar and spindle-associated protein 1 OS=Homo sapiens OX=9606 GN=NUSAP1 PE=1 SV=1 | 1827583 |
| DNA replication licensing factor MCM3 OS=Homo sapiens OX=9606 GN=MCM3 PE=1 SV=3 | 2083756 |
| Structural maintenance of chromosomes protein OS=Homo sapiens OX=9606 PE=2 SV=1 | 1137181 |
| MAZ protein OS=Homo sapiens OX=9606 PE=2 SV=1 | 7047946 |
| Centrosomal protein 170kDa OS=Homo sapiens OX=9606 GN=CEP170 PE=2 SV=1 | 4193337 |
| Ras GTPase-activating-like protein IQGAP1 OS=Homo sapiens OX=9606 GN=IQGAP1 PE=1 SV=1 | 1869330 |
| Transformer-2 protein homolog alpha OS=Homo sapiens OX=9606 GN=TRA2A PE=1 SV=1 | 12889598 |
| THO complex subunit 1 OS=Homo sapiens OX=9606 GN=THOC1 PE=1 SV=1 | 2499686 |
| Telomerase-binding protein EST1A OS=Homo sapiens OX=9606 GN=SMG6 PE=1 SV=2 | 3656636 |
| CLIP-associating protein 2 OS=Homo sapiens OX=9606 GN=CLASP2 PE=1 SV=1 | 3341340 |
| ATP synthase subunit beta (Fragment) OS=Homo sapiens OX=9606 GN=ATP5B PE=2 SV=1 | 915390.2 |
| 60S ribosomal protein L18a OS=Homo sapiens OX=9606 PE=2 SV=1 | 1.32E+08 |
| Ribonuclease P protein subunit p30 OS=Homo sapiens OX=9606 GN=RPP30 PE=1 SV=1 | 1322444 |
| Nucleosome assembly protein 1-like 1 OS=Homo sapiens OX=9606 GN=NAP1L1 PE=1 SV=1 | 6843834 |
| Gastrotropin OS=Homo sapiens OX=9606 GN=FABP6 PE=1 SV=2 | 12700944 |
| Exosome RNA helicase MTR4 OS=Homo sapiens OX=9606 GN=MTREX PE=1 SV=3 | 28461009 |
| Replication factor C subunit 1 OS=Homo sapiens OX=9606 GN=RFC1 PE=1 SV=4 | 13240469 |
| Tubulin beta chain OS=Homo sapiens OX=9606 GN=TUBB2C PE=2 SV=1 | 32835978 |
| Zinc finger Ran-binding domain-containing protein 2 OS=Homo sapiens OX=9606 GN=ZRANB2 PE=1 SV=2 | 4140331 |
| Helix-destabilizing protein OS=Homo sapiens OX=9606 GN=HNRNPA1 PE=1 SV=2 | 1.19E+08 |
| Nuclear export mediator factor NEMF OS=Homo sapiens OX=9606 GN=NEMF PE=1 SV=4 | 23352162 |
| Proteasome activator complex subunit 3 OS=Homo sapiens OX=9606 GN=PSME3 PE=1 SV=1 | 418793.2 |
| 40S ribosomal protein S15 OS=Homo sapiens OX=9606 GN=RPS15 PE=1 SV=2 | 66241006 |
| RNA helicase (Fragment) OS=Homo sapiens OX=9606 GN=DDX39A PE=1 SV=1 | 278205.8 |
| Cytosolic endo-beta-N-acetylglucosaminidase OS=Homo sapiens OX=9606 GN=ENGASE PE=1 SV=1 | 250371.9 |
| Family with sequence similarity 98, member A OS=Homo sapiens OX=9606 GN=FAM98A PE=2 SV=1 | 7175856 |
| 60S ribosomal protein L36 OS=Homo sapiens OX=9606 GN=RPL36 PE=1 SV=3 | 1.41E+08 |
| Interferon-related developmental regulator 1 (Fragment) OS=Homo sapiens OX=9606 GN=IFRD1 PE=1 SV=1 | 210137.5 |
| Apoptotic chromatin condensation inducer in the nucleus OS=Homo sapiens OX=9606 GN=ACIN1 PE=1 SV=2 | 18806426 |
| 40S ribosomal protein S25 OS=Homo sapiens OX=9606 GN=RPS25 PE=1 SV=1 | 2.57E+08 |
| Histone HIST2H3PS2 OS=Homo sapiens OX=9606 GN=H3-2 PE=1 SV=1 | 1.51E+08 |
| eIF2AK2 protein OS=Homo sapiens OX=9606 GN=EIF2AK2 PE=2 SV=1 | 5082481 |
| Histone H1.10 OS=Homo sapiens OX=9606 GN=H1-10 PE=1 SV=1 | 32584902 |
| GTP-binding nuclear protein Ran OS=Homo sapiens OX=9606 GN=RAN PE=1 SV=1 | 2319778 |
| PRP4 pre-mRNA-processing factor 4 homolog OS=Homo sapiens OX=9606 PE=2 SV=1 | 29512241 |
| Phosphate carrier protein, mitochondrial OS=Homo sapiens OX=9606 PE=2 SV=1 | 14291012 |
| DCN1-like protein (Fragment) OS=Homo sapiens OX=9606 GN=DCUN1D5 PE=1 SV=1 | 2151717 |
| Peptidyl-prolyl cis-trans isomerase OS=Homo sapiens OX=9606 GN=PPIH PE=1 SV=2 | 7255796 |
| F-actin-capping protein subunit beta OS=Homo sapiens OX=9606 PE=2 SV=1 | 1659979 |
| Pinin OS=Homo sapiens OX=9606 PE=2 SV=1 | 41734794 |
| Pre-mRNA-splicing factor SPF27 (Fragment) OS=Homo sapiens OX=9606 PE=2 SV=1 | 5572205 |
| IGH c3104_heavy_IGHV1-69_IGHD3-22_IGHJ4 (Fragment) OS=Homo sapiens OX=9606 PE=2 SV=1 | 47722503 |
| IGH + IGL c23_heavy_IGHV3-49_IGHD5-24_IGHJ6 (Fragment) OS=Homo sapiens OX=9606 PE=2 SV=1 | 4196454 |
| Zinc-alpha-2-glycoprotein OS=Homo sapiens OX=9606 GN=AZGP1 PE=1 SV=2 | 4794511 |
| Ribonucloprotein OS=Homo sapiens OX=9606 GN=SNU13 PE=1 SV=1 | 9937463 |
| H/ACA ribonucleoprotein complex subunit 1 OS=Homo sapiens OX=9606 GN=GAR1 PE=1 SV=1 | 26681909 |
| Mitochondrial ribosomal protein S2, isoform CRA_a OS=Homo sapiens OX=9606 GN=MRPS2 PE=3 SV=1 | 1273475 |
| SPATS2-like protein OS=Homo sapiens OX=9606 GN=SPATS2L PE=1 SV=2 | 3664769 |
| Ribosome production factor 2 homolog OS=Homo sapiens OX=9606 GN=RPF2 PE=1 SV=2 | 4574484 |
| cDNA FLJ77421, highly similar to Homo sapiens autoantigen p542 mRNA OS=Homo sapiens OX=9606 PE=2 SV=1 | 2473875 |
| Nucleolar complex protein 4 homolog OS=Homo sapiens OX=9606 GN=NOC4L PE=1 SV=1 | 13097206 |
| DAZ-associated protein 1 OS=Homo sapiens OX=9606 GN=DAZAP1 PE=1 SV=1 | 1951297 |
| Putative ribosome-binding factor A, mitochondrial OS=Homo sapiens OX=9606 GN=RBFA PE=1 SV=3 | 3580939 |
| Ribosomal RNA small subunit methyltransferase NEP1 OS=Homo sapiens OX=9606 GN=EMG1 PE=1 SV=4 | 3490247 |
| 28S ribosomal protein S22, mitochondrial OS=Homo sapiens OX=9606 GN=MRPS22 PE=1 SV=1 | 3825019 |
| cDNA FLJ56606, highly similar to Serine/threonine-protein kinase TAO2 OS=Homo sapiens OX=9606 PE=2 SV=1 | 7104825 |
| WD repeat-containing protein 74 OS=Homo sapiens OX=9606 GN=WDR74 PE=1 SV=1 | 1147780 |
| DnaJ homolog subfamily C member 21 OS=Homo sapiens OX=9606 GN=DNAJC21 PE=1 SV=2 | 3953173 |
| Pre-mRNA-splicing factor 38B OS=Homo sapiens OX=9606 GN=PRPF38B PE=1 SV=1 | 5874432 |
| Parafibromin OS=Homo sapiens OX=9606 GN=CDC73 PE=1 SV=1 | 7001948 |
| U4/U6.U5 tri-snRNP-associated protein 2 OS=Homo sapiens OX=9606 GN=USP39 PE=1 SV=2 | 10249615 |
| Small nuclear ribonucleoprotein polypeptide A' variant (Fragment) OS=Homo sapiens OX=9606 PE=2 SV=1 | 7763696 |
| 26S proteasome regulatory subunit RPN11 (Fragment) OS=Homo sapiens OX=9606 GN=PSMD14 PE=4 SV=1 | 129004.9 |
| ELAV-like protein 1 OS=Homo sapiens OX=9606 GN=ELAVL1 PE=1 SV=2 | 9596113 |
| DNA-directed RNA polymerase I subunit RPA49 OS=Homo sapiens OX=9606 GN=POLR1E PE=1 SV=3 | 4340492 |
| Heat shock 70 kDa protein 14 OS=Homo sapiens OX=9606 GN=HSPA14 PE=1 SV=1 | 6410260 |
| Arginine/serine-rich coiled-coil protein 2 OS=Homo sapiens OX=9606 GN=RSRC2 PE=1 SV=1 | 6770731 |
| Mitochondrial 2-oxoglutarate/malate carrier protein OS=Homo sapiens OX=9606 GN=SLC25A11 PE=1 SV=3 | 1958219 |
| T-complex protein 1 subunit theta OS=Homo sapiens OX=9606 GN=CCT8 PE=1 SV=4 | 1151485 |
| 60S ribosomal protein L37 OS=Homo sapiens OX=9606 GN=RPL37 PE=1 SV=2 | 3320792 |
| Microfibrillar-associated protein 1 OS=Homo sapiens OX=9606 GN=MFAP1 PE=1 SV=2 | 2708101 |
| DNA/RNA-binding protein KIN17 OS=Homo sapiens OX=9606 GN=KIN PE=1 SV=2 | 2475497 |
| Translation machinery-associated protein 16 (Fragment) OS=Homo sapiens OX=9606 GN=TMA16 PE=1 SV=1 | 7926694 |
| cDNA FLJ60124, highly similar to Mitochondrial dicarboxylate carrier OS=Homo sapiens OX=9606 PE=2 SV=1 | 2090740 |
| Peroxiredoxin-6 OS=Homo sapiens OX=9606 GN=PRDX6 PE=1 SV=3 | 698720.6 |
| Nucleoside diphosphate kinase A OS=Homo sapiens OX=9606 GN=NME1 PE=1 SV=1 | 968502.8 |
| Alpha-enolase OS=Homo sapiens OX=9606 GN=ENO1 PE=1 SV=2 | 1071150 |
| NADH dehydrogenase [ubiquinone] 1 beta subcomplex subunit 4 OS=Homo sapiens OX=9606 GN=NDUFB4 PE=1 SV=3 | 140594.8 |
| Nucleoplasmin-3 OS=Homo sapiens OX=9606 GN=NPM3 PE=1 SV=3 | 2206620 |
| DnaJ homolog subfamily A member 2 OS=Homo sapiens OX=9606 GN=DNAJA2 PE=1 SV=1 | 2333221 |
| Serine/threonine-protein kinase PLK OS=Homo sapiens OX=9606 PE=2 SV=1 | 1848073 |
| D-3-phosphoglycerate dehydrogenase OS=Homo sapiens OX=9606 GN=PHGDH PE=1 SV=4 | 2670644 |
| U2 snRNP-associated SURP motif-containing protein OS=Homo sapiens OX=9606 GN=U2SURP PE=1 SV=2 | 40602749 |
| E3 ubiquitin/ISG15 ligase TRIM25 OS=Homo sapiens OX=9606 GN=TRIM25 PE=1 SV=1 | 2376125 |
| T-complex protein 1 subunit delta OS=Homo sapiens OX=9606 GN=CCT4 PE=1 SV=4 | 1981533 |
| Something about silencing protein 10 OS=Homo sapiens OX=9606 GN=UTP3 PE=1 SV=1 | 11368567 |
| Zinc finger CCCH domain-containing protein 4 (Fragment) OS=Homo sapiens OX=9606 GN=ZC3H4 PE=1 SV=1 | 7479389 |
| Importin-5 (Fragment) OS=Homo sapiens OX=9606 GN=IPO5 PE=1 SV=2 | 322028.7 |
| Treacle protein OS=Homo sapiens OX=9606 GN=TCOF1 PE=1 SV=1 | 26707347 |
| cDNA FLJ58174, highly similar to WW domain-binding protein 11 OS=Homo sapiens OX=9606 PE=2 SV=1 | 2939639 |
| Autoantigen La (Fragment) OS=Homo sapiens OX=9606 GN=SSB PE=2 SV=1 | 5474220 |
| cDNA FLJ58619, highly similar to YTH domain protein 1 OS=Homo sapiens OX=9606 PE=2 SV=1 | 1107452 |
| Translation initiation factor eIF-2B subunit delta OS=Homo sapiens OX=9606 GN=EIF2B4 PE=1 SV=1 | 2326689 |
| Nuclear RNA export factor 1 OS=Homo sapiens OX=9606 GN=NXF1 PE=1 SV=1 | 4520996 |
| DDB1- and CUL4-associated factor 13 OS=Homo sapiens OX=9606 GN=DCAF13 PE=1 SV=1 | 2154650 |
| cDNA, FLJ92684, highly similar to Homo sapiens IK cytokine, down-regulator of HLA II (IK), mRNA OS=Homo sapiens OX=9606 PE=2 SV=1 | 12090379 |
| Heterochromatin protein 1-binding protein 3 (Fragment) OS=Homo sapiens OX=9606 GN=HP1BP3 PE=1 SV=1 | 1137066 |
| AP-3 complex subunit beta OS=Homo sapiens OX=9606 PE=2 SV=1 | 8324710 |
| Mitochondrial 28S ribosomal protein S34 OS=Homo sapiens OX=9606 PE=2 SV=1 | 3860957 |
| DNA-binding protein SMUBP-2 OS=Homo sapiens OX=9606 GN=IGHMBP2 PE=1 SV=3 | 2220080 |
| Ataxin-2 OS=Homo sapiens OX=9606 GN=ATXN2 PE=1 SV=1 | 11306342 |
| Protein KRI1 homolog OS=Homo sapiens OX=9606 GN=KRI1 PE=1 SV=1 | 11508016 |
| Pre-mRNA-splicing factor ATP-dependent RNA helicase DHX16 OS=Homo sapiens OX=9606 GN=DHX16 PE=1 SV=2 | 1828756 |
| Eukaryotic translation initiation factor 3 subunit D OS=Homo sapiens OX=9606 GN=EIF3D PE=1 SV=1 | 5073483 |
| Peroxisomal multifunctional enzyme type 2 OS=Homo sapiens OX=9606 GN=HSD17B4 PE=4 SV=1 | 3432289 |
| Heat shock 110 kDa protein OS=Homo sapiens OX=9606 GN=HSPH1 PE=3 SV=1 | 3104191 |
| WD repeat-containing protein 36 OS=Homo sapiens OX=9606 GN=WDR36 PE=1 SV=1 | 11238900 |
| T-complex protein 1 subunit eta OS=Homo sapiens OX=9606 GN=CCT7 PE=1 SV=2 | 3100103 |
| tRNA (adenine(58)-N(1))-methyltransferase non-catalytic subunit TRM6 OS=Homo sapiens OX=9606 GN=TRMT6 PE=1 SV=1 | 2545496 |
| Constitutive coactivator of PPAR-gamma-like protein 1 OS=Homo sapiens OX=9606 GN=FAM120A PE=1 SV=2 | 6205052 |
| Cell death regulator Aven OS=Homo sapiens OX=9606 GN=AVEN PE=1 SV=1 | 2765363 |
| Cleavage and polyadenylation specific factor 3, 73kDa variant (Fragment) OS=Homo sapiens OX=9606 PE=2 SV=1 | 4899645 |
| Probable ATP-dependent RNA helicase DDX31 OS=Homo sapiens OX=9606 GN=DDX31 PE=1 SV=2 | 7123603 |
| WD repeat-containing protein 43 OS=Homo sapiens OX=9606 GN=WDR43 PE=1 SV=3 | 5148935 |
| pre-mRNA 3' end processing protein WDR33 OS=Homo sapiens OX=9606 GN=WDR33 PE=1 SV=2 | 9000432 |
| PWP1 homolog (S. cerevisiae) OS=Homo sapiens OX=9606 GN=PWP1 PE=2 SV=1 | 17624277 |
| Nucleolar GTP-binding protein 2 OS=Homo sapiens OX=9606 GN=GNL2 PE=1 SV=1 | 4346744 |
| DnaJ homolog subfamily A member 3, mitochondrial OS=Homo sapiens OX=9606 GN=DNAJA3 PE=1 SV=2 | 3262659 |
| Protein-glutamine gamma-glutamyltransferase E OS=Homo sapiens OX=9606 GN=TGM3 PE=1 SV=4 | 1275265 |
| U4/U6.U5 small nuclear ribonucleoprotein 27 kDa protein OS=Homo sapiens OX=9606 GN=SNRNP27 PE=1 SV=1 | 335683.4 |
| Calcium homeostasis endoplasmic reticulum protein OS=Homo sapiens OX=9606 GN=CHERP PE=1 SV=3 | 21464296 |
| KRR1 small subunit processome component homolog OS=Homo sapiens OX=9606 GN=KRR1 PE=1 SV=4 | 8348549 |
| Cyclin-dependent kinase 12 OS=Homo sapiens OX=9606 GN=CDK12 PE=1 SV=2 | 1834378 |
| Squamous cell carcinoma antigen recognized by T-cells 3 OS=Homo sapiens OX=9606 GN=SART3 PE=1 SV=1 | 1928422 |
| Protein arginine methyltransferase 1 isoform 4 OS=Homo sapiens OX=9606 GN=HRMT1L2 PE=2 SV=1 | 1449323 |
| SWI/SNF-related matrix-associated actin-dependent regulator of chromatin subfamily A member 5 OS=Homo sapiens OX=9606 GN=SMARCA5 PE=1 SV=1 | 2037251 |
| Zinc finger protein 207 variant (Fragment) OS=Homo sapiens OX=9606 PE=2 SV=1 | 20462728 |
| cDNA FLJ61387, highly similar to Homo sapiens conserved nuclear protein NHN1 (NHN1), mRNA OS=Homo sapiens OX=9606 PE=2 SV=1 | 3327689 |
| Elongation factor 1-alpha (Fragment) OS=Homo sapiens OX=9606 PE=2 SV=1 | 95591732 |
| Splicing factor 3a, subunit 3 variant (Fragment) OS=Homo sapiens OX=9606 PE=2 SV=1 | 10936944 |
| LUC7-like isoform b variant (Fragment) OS=Homo sapiens OX=9606 PE=2 SV=1 | 1980078 |
| Eukaryotic translation initiation factor 4E OS=Homo sapiens OX=9606 GN=EIF4E PE=2 SV=1 | 4769978 |
| Pumilio homolog 3 OS=Homo sapiens OX=9606 GN=PUM3 PE=1 SV=3 | 6641611 |
| Ensconsin OS=Homo sapiens OX=9606 GN=MAP7 PE=1 SV=1 | 5232551 |
| cDNA, FLJ96811, highly similar to Homo sapiens G patch domain containing 1 (GPATC1), mRNA OS=Homo sapiens OX=9606 PE=2 SV=1 | 4051911 |
| RNA-binding protein 5 OS=Homo sapiens OX=9606 GN=RBM5 PE=1 SV=2 | 737669.4 |
| cDNA FLJ32300 fis, clone PROST2002227, highly similar to U3 small nucleolar ribonucleoprotein protein MPP10 (Fragment) OS=Homo sapiens OX=9606 PE=2 SV=1 | 7540905 |
| Nucleolar protein 6 OS=Homo sapiens OX=9606 GN=NOL6 PE=1 SV=1 | 1.11E+08 |
| cDNA FLJ77055, highly similar to Homo sapiens WD repeat domain 75 (WDR75), mRNA (Fragment) OS=Homo sapiens OX=9606 PE=2 SV=1 | 2317334 |
| Desmocollin 1, isoform CRA_b OS=Homo sapiens OX=9606 GN=DSC1 PE=4 SV=1 | 2216421 |
| RNA-binding protein 33 OS=Homo sapiens OX=9606 GN=RBM33 PE=1 SV=3 | 3434717 |
| Decreased expression in renal and prostate cancer protein OS=Homo sapiens OX=9606 GN=DERPC PE=1 SV=1 | 1348826 |
| THO complex subunit 2 OS=Homo sapiens OX=9606 GN=THOC2 PE=1 SV=2 | 2378652 |
| Ankyrin repeat and KH domain-containing protein 1 (Fragment) OS=Homo sapiens OX=9606 GN=ANKHD1 PE=1 SV=1 | 1628612 |
| CDP-diacylglycerol--inositol 3-phosphatidyltransferase OS=Homo sapiens OX=9606 GN=CDIPT PE=1 SV=1 | 556263.3 |
| Translation initiation factor eIF-2B subunit alpha (Fragment) OS=Homo sapiens OX=9606 GN=EIF2B1 PE=1 SV=1 | 623364.5 |
| Protein ZNRD2 (Fragment) OS=Homo sapiens OX=9606 GN=ZNRD2 PE=1 SV=1 | 470378 |
| PHD and RING finger domain-containing protein 1 OS=Homo sapiens OX=9606 GN=PHRF1 PE=1 SV=1 | 1587099 |
| EDC4 variant OS=Homo sapiens OX=9606 GN=EDC4 PE=2 SV=1 | 1392858 |
| THO complex subunit 5 homolog OS=Homo sapiens OX=9606 GN=THOC5 PE=1 SV=1 | 1294624 |
| Periphilin-1 OS=Homo sapiens OX=9606 GN=PPHLN1 PE=1 SV=1 | 772647.5 |
| Nuclear cap-binding protein subunit 3 OS=Homo sapiens OX=9606 GN=NCBP3 PE=1 SV=2 | 3870218 |
| 28S ribosomal protein S17, mitochondrial (Fragment) OS=Homo sapiens OX=9606 GN=MRPS17 PE=1 SV=1 | 1839228 |
| ATP synthase subunit alpha, mitochondrial OS=Homo sapiens OX=9606 GN=ATP5F1A PE=1 SV=1 | 4449713 |
| cDNA, FLJ94440, highly similar to Homo sapiens chaperonin containing TCP1, subunit 6A (zeta 1)(CCT6A), mRNA OS=Homo sapiens OX=9606 PE=2 SV=1 | 3244373 |
| Lymphoid-specific helicase OS=Homo sapiens OX=9606 GN=HELLS PE=1 SV=1 | 2466710 |
| ATP-dependent DNA helicase Q1 OS=Homo sapiens OX=9606 GN=RECQL PE=1 SV=3 | 3424285 |
| cDNA FLJ53294 OS=Homo sapiens OX=9606 PE=2 SV=1 | 1169539 |
| Uncharacterized protein DKFZp686A111 OS=Homo sapiens OX=9606 GN=DKFZp686A111 PE=2 SV=1 | 2975041 |
| Coiled-coil and C2 domain-containing protein 1A OS=Homo sapiens OX=9606 GN=CC2D1A PE=1 SV=1 | 841451.6 |
| DNA damage-binding protein 1 OS=Homo sapiens OX=9606 GN=DDB1 PE=1 SV=1 | 345129.2 |
| Alpha-1,3-glucosyltransferase OS=Homo sapiens OX=9606 PE=2 SV=1 | 1901137 |
| Protein DEK OS=Homo sapiens OX=9606 GN=DEK PE=1 SV=1 | 478821.1 |
| cDNA FLJ10529 fis, clone NT2RP2000965, highly similar to Targeting protein for Xklp2 OS=Homo sapiens OX=9606 PE=2 SV=1 | 1833136 |
| Gamma-glutamylcyclotransferase OS=Homo sapiens OX=9606 GN=GGCT PE=1 SV=1 | 599499.5 |
| Casein kinase II subunit beta OS=Homo sapiens OX=9606 GN=CSNK2B PE=1 SV=1 | 5032522 |
| Plasminogen activator inhibitor 1 RNA-binding protein OS=Homo sapiens OX=9606 GN=SERBP1 PE=1 SV=2 | 2.87E+08 |
| Histone H2B type 2-F OS=Homo sapiens OX=9606 GN=H2BC18 PE=1 SV=3 | 3.15E+08 |
| cDNA FLJ76888, highly similar to Homo sapiens RNA binding motif protein 6 (RBM6), mRNA OS=Homo sapiens OX=9606 PE=2 SV=1 | 7774561 |
| 60S ribosomal protein L38 OS=Homo sapiens OX=9606 GN=RPL38 PE=1 SV=2 | 28033170 |
| Isoleucine--tRNA ligase, cytoplasmic OS=Homo sapiens OX=9606 GN=IARS1 PE=4 SV=1 | 12891613 |
| Transcription factor BTF3 OS=Homo sapiens OX=9606 GN=BTF3 PE=1 SV=1 | 6197363 |
| Vesicle-fusing ATPase OS=Homo sapiens OX=9606 GN=SPATA5 PE=1 SV=1 | 5396994 |
| NF-kappa-B-activating protein (Fragment) OS=Homo sapiens OX=9606 GN=NKAP PE=1 SV=1 | 96890109 |
| Cystatin-A OS=Homo sapiens OX=9606 GN=CSTA PE=1 SV=1 | 518262.5 |
| Homeobox protein NK-2 homolog E (Fragment) OS=Homo sapiens OX=9606 GN=NKX 2-5 PE=3 SV=1 | 165808.7 |
| Methyltransferase like 17 isoform 1 (Fragment) OS=Homo sapiens OX=9606 GN=METTL17 PE=2 SV=1 | 2205827 |
| Small nuclear ribonucleoprotein F OS=Homo sapiens OX=9606 GN=SNRPF PE=1 SV=1 | 9864182 |
| T cell receptor beta variable 4-1 (Fragment) OS=Homo sapiens OX=9606 GN=TRBV4-1 PE=4 SV=1 | 805805.9 |
| Ribosome biogenesis protein NSA2 homolog OS=Homo sapiens OX=9606 GN=NSA2 PE=1 SV=1 | 423174.1 |
| Fibroblast growth factor OS=Homo sapiens OX=9606 GN=FGF2 PE=1 SV=1 | 2502253 |
| Exosome complex component CSL4 OS=Homo sapiens OX=9606 GN=EXOSC1 PE=1 SV=1 | 1955873 |
| ATP-dependent RNA helicase DHX29 OS=Homo sapiens OX=9606 GN=DHX29 PE=1 SV=1 | 7063112 |
| Transcription initiation factor TFIID 150 kDa subunit OS=Homo sapiens OX=9606 GN=TAF2 PE=3 SV=1 | 12107843 |
| Prolactin-inducible protein OS=Homo sapiens OX=9606 GN=PIP PE=1 SV=1 | 1310715 |
| 28S ribosomal protein S16, mitochondrial OS=Homo sapiens OX=9606 GN=MRPS16 PE=1 SV=1 | 2804770 |
| Nucleolar protein 7 OS=Homo sapiens OX=9606 GN=NOL7 PE=1 SV=2 | 1906969 |
| Peptidyl-prolyl cis-trans isomerase-like 1 OS=Homo sapiens OX=9606 GN=PPIL1 PE=1 SV=1 | 3741638 |
| CDKN2A-interacting protein OS=Homo sapiens OX=9606 GN=CDKN2AIP PE=1 SV=3 | 1461725 |
| cDNA FLJ58391, highly similar to Exosome complex exonuclease RRP42 OS=Homo sapiens OX=9606 PE=2 SV=1 | 3280145 |
| 1,4-beta-N-acetylmuramidase C OS=Homo sapiens OX=9606 GN=LYZ PE=1 SV=1 | 2058220 |
| Coiled-coil domain-containing protein 86 OS=Homo sapiens OX=9606 GN=CCDC86 PE=1 SV=1 | 1686472 |
| Spermatogenesis-associated protein 5-like protein 1 OS=Homo sapiens OX=9606 GN=SPATA5L1 PE=1 SV=2 | 1796483 |
| Small nuclear ribonucleoprotein polypeptide C variant (Fragment) OS=Homo sapiens OX=9606 PE=2 SV=1 | 6286783 |
| Serine/threonine-protein phosphatase 1 regulatory subunit 10 OS=Homo sapiens OX=9606 GN=PPP1R10 PE=1 SV=1 | 16071849 |
| Ribosomal RNA processing protein 36 homolog OS=Homo sapiens OX=9606 GN=RRP36 PE=1 SV=1 | 530671.7 |
| Splicing regulatory glutamine/lysine-rich protein 1 OS=Homo sapiens OX=9606 GN=SREK1 PE=1 SV=1 | 19857926 |
| ZNF277 protein OS=Homo sapiens OX=9606 GN=ZNF277 PE=1 SV=1 | 742812.1 |
| Cytoplasmic dynein 2 intermediate chain 1 OS=Homo sapiens OX=9606 GN=DYNC2I1 PE=1 SV=3 | 3597100 |
| Nucleolar complex protein 3 homolog OS=Homo sapiens OX=9606 GN=NOC3L PE=1 SV=1 | 7974240 |
| Protein FAM76A OS=Homo sapiens OX=9606 GN=FAM76A PE=2 SV=1 | 648139.3 |
| Ribonuclease P protein subunit p25-like protein OS=Homo sapiens OX=9606 GN=RPP25L PE=1 SV=1 | 745560.4 |
| Uncharacterized protein DKFZp451A052 OS=Homo sapiens OX=9606 GN=DKFZp451A052 PE=2 SV=2 | 281555.1 |
| Protein mago nashi homolog OS=Homo sapiens OX=9606 GN=MAGOH PE=1 SV=1 | 11526651 |
| Tubulin epsilon and delta complex protein 1 OS=Homo sapiens OX=9606 GN=TEDC1 PE=2 SV=2 | 1994646 |
| Keratin, type II cytoskeletal 1b OS=Homo sapiens OX=9606 GN=KRT77 PE=1 SV=3 | 1245811 |
| Nuclear cap-binding protein subunit 2 OS=Homo sapiens OX=9606 GN=NCBP2 PE=1 SV=1 | 1850402 |
| 7SK snRNA methylphosphate capping enzyme OS=Homo sapiens OX=9606 GN=MEPCE PE=1 SV=1 | 964566.9 |
| LKHP9428 OS=Homo sapiens OX=9606 GN=UNQ9428 PE=2 SV=1 | 18179494 |
| RNA helicase OS=Homo sapiens OX=9606 GN=DHX35 PE=1 SV=1 | 5116011 |
| Deoxynucleotidyltransferase terminal-interacting protein 2 OS=Homo sapiens OX=9606 GN=DNTTIP2 PE=1 SV=2 | 3886569 |
| Ribonuclease P protein subunit p14 OS=Homo sapiens OX=9606 GN=RPP14 PE=1 SV=3 | 1628595 |
| Nucleoside diphosphate kinase OS=Homo sapiens OX=9606 GN=NME4 PE=1 SV=1 | 800950.8 |
| 39S ribosomal protein L24, mitochondrial OS=Homo sapiens OX=9606 GN=MRPL24 PE=1 SV=1 | 1888410 |
| Splicing factor, arginine/serine-rich 10 (Transformer 2 homolog, Drosophila) variant (Fragment) OS=Homo sapiens OX=9606 PE=2 SV=1 | 10307322 |
| Small nuclear ribonucleoprotein Sm D1 OS=Homo sapiens OX=9606 GN=SNRPD1 PE=1 SV=1 | 18257554 |
| Exosome complex component RRP40 OS=Homo sapiens OX=9606 GN=EXOSC3 PE=1 SV=3 | 7136472 |
| Ribosomal RNA-processing protein 8 OS=Homo sapiens OX=9606 GN=RRP8 PE=1 SV=1 | 2337403 |
| Peptidyl-prolyl cis-trans isomerase (Fragment) OS=Homo sapiens OX=9606 GN=PPIG PE=2 SV=1 | 13814861 |
| AP-2 complex subunit alpha-1 OS=Homo sapiens OX=9606 GN=AP2A1 PE=1 SV=3 | 7538750 |
| PRA1 family protein 2 OS=Homo sapiens OX=9606 GN=PRAF2 PE=1 SV=1 | 124141 |
| Heterogeneous nuclear ribonucleoprotein A0 OS=Homo sapiens OX=9606 GN=HNRNPA0 PE=1 SV=1 | 25658425 |
| Probable ATP-dependent RNA helicase DHX40 (Fragment) OS=Homo sapiens OX=9606 GN=DHX40 PE=1 SV=1 |  |
| Chromobox protein homolog 1 (Fragment) OS=Homo sapiens OX=9606 GN=CBX1 PE=1 SV=8 | 2140047 |
| 28S ribosomal protein S28, mitochondrial (Fragment) OS=Homo sapiens OX=9606 GN=MRPS28 PE=1 SV=8 | 1294285 |
| Casein kinase I isoform delta OS=Homo sapiens OX=9606 GN=CSNK1D PE=1 SV=2 | 2377604 |
| Coiled-coil domain-containing protein 124 OS=Homo sapiens OX=9606 GN=CCDC124 PE=1 SV=1 | 4348401 |
| Protein FAM98B OS=Homo sapiens OX=9606 GN=FAM98B PE=1 SV=2 | 1418875 |
| Histone lysine demethylase PHF8 (Fragment) OS=Homo sapiens OX=9606 GN=PHF8 PE=1 SV=1 | 4898095 |
| Acylglycerol kinase, mitochondrial OS=Homo sapiens OX=9606 GN=AGK PE=1 SV=1 | 1490328 |
| Survival of motor neuron-related-splicing factor 30 OS=Homo sapiens OX=9606 GN=SMNDC1 PE=1 SV=1 | 3569583 |
| DING protein p38 OS=Homo sapiens OX=9606 PE=3 SV=1 | 9616543 |
| cDNA FLJ55568, highly similar to Rho/Rac guanine nucleotide exchange factor 2 OS=Homo sapiens OX=9606 PE=2 SV=1 | 1543762 |
| DnaJ homolog subfamily C member 9 OS=Homo sapiens OX=9606 GN=DNAJC9 PE=1 SV=1 | 4899943 |
| Protein transport protein Sec61 subunit alpha isoform 1 OS=Homo sapiens OX=9606 GN=SEC61A1 PE=1 SV=1 | 3507239 |
| Myosin light polypeptide 6 OS=Homo sapiens OX=9606 GN=MYL6 PE=1 SV=1 | 1945604 |
| Exosome complex component RRP43 OS=Homo sapiens OX=9606 GN=EXOSC8 PE=1 SV=1 | 19654799 |
| Emerin OS=Homo sapiens OX=9606 GN=EMD PE=1 SV=1 | 1763131 |
| Ribonuclease P protein subunit p38 OS=Homo sapiens OX=9606 GN=RPP38 PE=1 SV=2 | 3039611 |
| cDNA, FLJ96442, highly similar to Homo sapiens copine II (CPNE2), mRNA OS=Homo sapiens OX=9606 PE=2 SV=1 | 2287454 |
| Sideroflexin-1 OS=Homo sapiens OX=9606 GN=SFXN1 PE=1 SV=4 | 1346316 |
| SF3A2 protein (Fragment) OS=Homo sapiens OX=9606 GN=SF3A2 PE=2 SV=1 | 4924591 |
| Ribosome biogenesis protein BOP1 OS=Homo sapiens OX=9606 GN=BOP1 PE=2 SV=1 | 4891963 |
| cDNA FLJ78373, highly similar to Homo sapiens PCI domain containing 2, mRNA OS=Homo sapiens OX=9606 PE=2 SV=1 | 1185078 |
| Polypyrimidine tract-binding protein 1 OS=Homo sapiens OX=9606 GN=PTBP1 PE=1 SV=4 | 73945782 |
| IG c509_light_IGKV3-15_IGKJ1 (Fragment) OS=Homo sapiens OX=9606 PE=2 SV=1 | 1014408 |
| Prohibitin OS=Homo sapiens OX=9606 GN=PHB2 PE=1 SV=1 | 1490792 |
| E3 ubiquitin-protein ligase RBBP6 (Fragment) OS=Homo sapiens OX=9606 GN=RBBP6 PE=1 SV=1 | 9648089 |
| Mitochondrial transcription factor A OS=Homo sapiens OX=9606 PE=4 SV=1 | 738427 |
| Protein kinase C and casein kinase substrate in neurons protein 3 (Fragment) OS=Homo sapiens OX=9606 GN=PACSIN3 PE=1 SV=1 | 1527694 |
| HCG2039996 OS=Homo sapiens OX=9606 GN=PPAN-P2RY11 PE=3 SV=1 | 21734494 |
| Ribose-phosphate pyrophosphokinase 1 OS=Homo sapiens OX=9606 GN=PRPS1 PE=1 SV=2 | 2248349 |
| DnaJ OS=Homo sapiens OX=9606 PE=2 SV=1 | 3211855 |
| Elongin-A OS=Homo sapiens OX=9606 GN=ELOA PE=1 SV=1 | 6453505 |
| Ubiquitinyl hydrolase 1 OS=Homo sapiens OX=9606 GN=OTUD6B PE=1 SV=1 | 1823744 |
| A-kinase anchor protein 17A OS=Homo sapiens OX=9606 GN=AKAP17A PE=1 SV=1 | 3671146 |
| 60S ribosomal protein L7-like 1 (Fragment) OS=Homo sapiens OX=9606 GN=RPL7L1 PE=1 SV=2 | 778753.3 |
| Double-stranded RNA-binding protein Staufen homolog 2 OS=Homo sapiens OX=9606 GN=STAU2 PE=4 SV=1 | 771811.2 |
| 18S rRNA aminocarboxypropyltransferase OS=Homo sapiens OX=9606 GN=TSR3 PE=1 SV=1 | 1216103 |
| Malignant T-cell-amplified sequence 1 OS=Homo sapiens OX=9606 GN=MCTS1 PE=1 SV=1 | 900700.4 |
| General transcription factor 3C polypeptide 4 OS=Homo sapiens OX=9606 GN=GTF3C4 PE=1 SV=2 | 2295054 |
| NTF2-related export protein 1 OS=Homo sapiens OX=9606 GN=NXT1 PE=1 SV=1 | 4011602 |
| RNA-binding protein NOB1 OS=Homo sapiens OX=9606 GN=NOB1 PE=1 SV=1 | 3165482 |
| CGG triplet repeat-binding protein 1 OS=Homo sapiens OX=9606 GN=CGGBP1 PE=1 SV=2 | 556259.8 |
| Uncharacterized protein ORC5L (Fragment) OS=Homo sapiens OX=9606 GN=ORC5L PE=3 SV=1 | 517319.5 |
| Cyclin-L1 OS=Homo sapiens OX=9606 GN=CCNL1 PE=1 SV=1 | 5826050 |
| Malonyl-CoA-acyl carrier protein transacylase, mitochondrial OS=Homo sapiens OX=9606 GN=MCAT PE=1 SV=2 | 838891.8 |
| cDNA FLJ20187 fis, clone COLF0433 OS=Homo sapiens OX=9606 PE=2 SV=1 | 1404024 |
| Poly(A) RNA polymerase, mitochondrial OS=Homo sapiens OX=9606 GN=MTPAP PE=1 SV=1 | 3171775 |
| RNA-binding protein with serine-rich domain 1 (Fragment) OS=Homo sapiens OX=9606 GN=RNPS1 PE=1 SV=1 | 6039688 |
| cDNA FLJ13989 fis, clone Y79AA1002083, highly similar to H.sapiens MUF1 protein OS=Homo sapiens OX=9606 PE=2 SV=1 | 635389.4 |
| ATP-dependent RNA helicase DHX33 OS=Homo sapiens OX=9606 GN=DHX33 PE=1 SV=2 | 2136962 |
| Obg-like ATPase 1 OS=Homo sapiens OX=9606 GN=OLA1 PE=1 SV=2 | 5017060 |
| WD repeat-containing protein 18 OS=Homo sapiens OX=9606 GN=WDR18 PE=1 SV=2 | 2082831 |
| BUD13 homolog OS=Homo sapiens OX=9606 GN=BUD13 PE=1 SV=1 | 4544488 |
| Plakophilin-2 OS=Homo sapiens OX=9606 GN=PKP2 PE=1 SV=2 | 6378235 |
| Uncharacterized protein (Fragment) OS=Homo sapiens OX=9606 PE=2 SV=1 | 1879632 |
| U3 small nucleolar ribonucleoprotein protein IMP4 (Fragment) OS=Homo sapiens OX=9606 GN=IMP4 PE=1 SV=1 | 13053334 |
| Peptidyl-prolyl cis-trans isomerase-like 4 OS=Homo sapiens OX=9606 GN=PPIL4 PE=1 SV=1 | 2195624 |
| Activating signal cointegrator 1 complex subunit 1 OS=Homo sapiens OX=9606 GN=ASCC1 PE=1 SV=1 | 1393533 |
| cDNA FLJ51014, highly similar to WD repeat protein 46 OS=Homo sapiens OX=9606 PE=2 SV=1 | 468465.4 |
| cDNA FLJ90282 fis, clone NT2RP1000551, highly similar to Interferon-related developmental regulator 2 OS=Homo sapiens OX=9606 PE=2 SV=1 | 1006934 |
| Zinc finger CCCH-type with G patch domain-containing protein OS=Homo sapiens OX=9606 GN=ZGPAT PE=1 SV=3 | 437521.3 |
| cDNA, FLJ94570, Homo sapiens mesenchymal stem cell protein DSC43 (LOC51333), mRNA OS=Homo sapiens OX=9606 PE=2 SV=1 | 985773.1 |
| Solute carrier family 25 (Mitochondrial carrier: glutamate), member 22, isoform CRA_a OS=Homo sapiens OX=9606 GN=SLC25A22 PE=3 SV=1 | 1071648 |
| RNA polymerase II-associated factor 1 homolog OS=Homo sapiens OX=9606 GN=PAF1 PE=1 SV=2 | 463765.6 |
| Lysine-specific demethylase RSBN1L OS=Homo sapiens OX=9606 GN=RSBN1L PE=1 SV=2 | 19151901 |
| RNA demethylase ALKBH5 OS=Homo sapiens OX=9606 GN=ALKBH5 PE=1 SV=2 | 4343121 |
| Zinc finger CCCH domain-containing protein 14 OS=Homo sapiens OX=9606 GN=FLJ11806 PE=3 SV=1 | 3087741 |
| Glutaminyl-peptide cyclotransferase-like protein OS=Homo sapiens OX=9606 GN=QPCTL PE=1 SV=2 | 1739530 |
| Testis-expressed protein 30 OS=Homo sapiens OX=9606 GN=TEX30 PE=2 SV=1 |  |
| Ubiquitin-like modifier-activating enzyme 1 (Fragment) OS=Homo sapiens OX=9606 GN=UBA1 PE=1 SV=8 | 348221.4 |
| FUS interacting protein (Serine-arginine rich) 1 isoform 2 variant (Fragment) OS=Homo sapiens OX=9606 PE=2 SV=1 | 4023375 |
| Magnesium transporter protein 1 OS=Homo sapiens OX=9606 GN=MAGT1 PE=1 SV=1 | 1689836 |
| Polynucleotide adenylyltransferase (Fragment) OS=Homo sapiens OX=9606 GN=PAPOLG PE=2 SV=1 | 595571.7 |
| Serine/threonine-protein phosphatase 2A 55 kDa regulatory subunit B OS=Homo sapiens OX=9606 PE=2 SV=1 | 3139819 |
| Mitochondrial ribosomal protein L11 isoform a variant (Fragment) OS=Homo sapiens OX=9606 PE=2 SV=1 | 635975.3 |
| Flap endonuclease 1 OS=Homo sapiens OX=9606 GN=FEN1 PE=1 SV=1 | 1010356 |
| Radixin OS=Homo sapiens OX=9606 GN=RDX PE=1 SV=1 | 2201300 |
| Mitotic spindle assembly checkpoint protein MAD2A OS=Homo sapiens OX=9606 GN=MAD2L1 PE=1 SV=1 | 564224.6 |
| Cytoplasmic dynein 2 intermediate chain 2 OS=Homo sapiens OX=9606 GN=DYNC2I2 PE=1 SV=2 | 753350.5 |
| tRNA (uracil-5-)-methyltransferase homolog A OS=Homo sapiens OX=9606 GN=TRMT2A PE=1 SV=2 | 672426.6 |
| CWF19-like protein 1 OS=Homo sapiens OX=9606 GN=CWF19L1 PE=1 SV=2 | 797004.5 |
| Fibrinogen beta chain OS=Homo sapiens OX=9606 GN=FGB PE=4 SV=1 | 2268346 |
| cDNA FLJ75526, highly similar to Homo sapiens proliferation-associated 2G4, 38kDa (PA2G4), mRNA (Fragment) OS=Homo sapiens OX=9606 PE=2 SV=1 | 3858757 |
| Keratinocyte proline-rich protein OS=Homo sapiens OX=9606 GN=KPRP PE=1 SV=1 | 1959678 |
| Methionine--tRNA ligase, cytoplasmic OS=Homo sapiens OX=9606 GN=MARS1 PE=1 SV=2 | 8770399 |
| Centrosomal protein of 55 kDa OS=Homo sapiens OX=9606 GN=CEP55 PE=1 SV=3 | 1852608 |
| IQ calmodulin-binding motif-containing protein 1 OS=Homo sapiens OX=9606 GN=IQCB1 PE=1 SV=1 | 1742824 |
| T-complex protein 1 subunit beta OS=Homo sapiens OX=9606 GN=CCT2 PE=1 SV=4 | 13625675 |
| Eukaryotic translation initiation factor 2D OS=Homo sapiens OX=9606 GN=EIF2D PE=1 SV=3 | 1132471 |
| Alpha-adducin OS=Homo sapiens OX=9606 GN=ADD1 PE=1 SV=1 | 2073036 |
| cDNA FLJ45688 fis, clone FCBBF3021191, highly similar to Protein phosphatase 2C isoform gamma OS=Homo sapiens OX=9606 PE=2 SV=1 | 27782003 |
| NADH dehydrogenase [ubiquinone] iron-sulfur protein 3, mitochondrial OS=Homo sapiens OX=9606 GN=NDUFS3 PE=1 SV=1 | 166642.9 |
| cDNA FLJ39022 fis, clone NT2RP7003724, weakly similar to Serine/arginine repetitive matrix protein 1 OS=Homo sapiens OX=9606 PE=2 SV=1 | 2155168 |
| Probable helicase with zinc finger domain OS=Homo sapiens OX=9606 GN=HELZ PE=1 SV=1 | 1554149 |
| PAX3- and PAX7-binding protein 1 OS=Homo sapiens OX=9606 GN=PAXBP1 PE=1 SV=2 | 1010577 |
| Protein ECT2 (Fragment) OS=Homo sapiens OX=9606 GN=ECT2 PE=1 SV=1 | 758428.9 |
| HCG2044799 OS=Homo sapiens OX=9606 GN=HNRNPUL2-BSCL2 PE=4 SV=1 | 2235999 |
| Nucleolar protein 11 OS=Homo sapiens OX=9606 GN=NOL11 PE=1 SV=1 | 981653.5 |
| Asparagine--tRNA ligase, cytoplasmic OS=Homo sapiens OX=9606 GN=NARS1 PE=1 SV=1 | 556255.2 |
| Complex I-20kD OS=Homo sapiens OX=9606 GN=NDUFS7 PE=1 SV=1 | 328284.7 |
| DnaJ homolog subfamily C member 2 OS=Homo sapiens OX=9606 GN=DNAJC2 PE=1 SV=4 | 3877810 |
| Protein SDA1 OS=Homo sapiens OX=9606 PE=2 SV=1 | 1796530 |
| RNA helicase OS=Homo sapiens OX=9606 GN=DDX20 PE=2 SV=1 | 1112116 |
| MAP7 domain-containing protein 3 OS=Homo sapiens OX=9606 GN=MAP7D3 PE=1 SV=2 | 945104.8 |
| cDNA, FLJ79026, highly similar to Dual specificity protein kinase CLK1 (EC2.7.12.1) OS=Homo sapiens OX=9606 PE=2 SV=1 | 1880994 |
| Sodium/potassium-transporting ATPase subunit alpha (Fragment) OS=Homo sapiens OX=9606 PE=2 SV=1 | 1909058 |
| Transcription initiation factor TFIID subunit 3 OS=Homo sapiens OX=9606 GN=TAF3 PE=1 SV=1 | 111121.9 |
| cDNA FLJ55299, highly similar to RNA exonuclease 1 homolog OS=Homo sapiens OX=9606 PE=2 SV=1 | 449126.2 |
| Nuclear cap-binding protein subunit 1 OS=Homo sapiens OX=9606 GN=NCBP1 PE=1 SV=1 | 1386979 |
| cDNA FLJ53652, highly similar to Mitochondrial 28S ribosomal protein S27 OS=Homo sapiens OX=9606 PE=2 SV=1 | 2265160 |
| HCG2039447, isoform CRA_d OS=Homo sapiens OX=9606 GN=hCG_2039447 PE=1 SV=1 | 1574660 |
| 39S ribosomal protein L9, mitochondrial OS=Homo sapiens OX=9606 PE=2 SV=1 | 1526594 |
| cDNA FLJ57701, highly similar to Homo sapiens Cas-Br-M (murine) ecotropic retroviral transforming sequence-like 1 (CBLL1), mRNA OS=Homo sapiens OX=9606 PE=2 SV=1 | 221008.2 |
| cDNA FLJ90427 fis, clone NT2RP3000481, highly similar to Importin-7 OS=Homo sapiens OX=9606 PE=2 SV=1 | 851432.1 |
| Cytoskeleton associated protein 2 OS=Homo sapiens OX=9606 GN=CKAP2 PE=2 SV=1 | 4332491 |
| Mediator of RNA polymerase II transcription subunit 17 OS=Homo sapiens OX=9606 GN=MED17 PE=1 SV=1 | 1199792 |
| cDNA, FLJ94423, highly similar to Homo sapiens mitochondrial ribosomal protein L23 (MRPL23), nuclear gene encoding mitochondrial protein, mRNA OS=Homo sapiens OX=9606 PE=2 SV=1 | 2515597 |
| Band 4.1-like protein 5 OS=Homo sapiens OX=9606 GN=EPB41L5 PE=1 SV=3 | 2081501 |
| Cyclin-T1 OS=Homo sapiens OX=9606 PE=2 SV=1 | 4138088 |
| Nck-associated protein 1 OS=Homo sapiens OX=9606 GN=NCKAP1 PE=1 SV=1 | 447317.7 |
| Zinc finger CCCH domain-containing protein 7A OS=Homo sapiens OX=9606 GN=ZC3H7A PE=1 SV=1 | 1256936 |
| Serine/threonine-protein kinase VRK1 OS=Homo sapiens OX=9606 GN=VRK1 PE=1 SV=1 | 857111.6 |
| Splicing factor 1 OS=Homo sapiens OX=9606 GN=SF1 PE=1 SV=1 | 10716920 |
| Sorting nexin OS=Homo sapiens OX=9606 GN=SNX9 PE=1 SV=1 | 1248125 |
| Bifunctional lysine-specific demethylase and histidyl-hydroxylase OS=Homo sapiens OX=9606 GN=MDIG PE=2 SV=1 | 2796750 |
| DNA polymerase OS=Homo sapiens OX=9606 GN=POLD1 PE=1 SV=1 | 543159.2 |
| cDNA FLJ56024 OS=Homo sapiens OX=9606 PE=2 SV=1 | 5457923 |
| cDNA FLJ54437, highly similar to RRP5 protein homolog (Fragment) OS=Homo sapiens OX=9606 PE=2 SV=1 | 822004.4 |
| Protein regulator of cytokinesis 1, isoform CRA_e OS=Homo sapiens OX=9606 GN=PRC1 PE=4 SV=1 | 5116653 |
| Dedicator of cytokinesis protein 7 OS=Homo sapiens OX=9606 GN=DOCK7 PE=1 SV=4 | 862846.1 |
| AP complex subunit sigma OS=Homo sapiens OX=9606 GN=AP2S1 PE=1 SV=1 | 1024506 |
| Pantothenate kinase 2, mitochondrial OS=Homo sapiens OX=9606 GN=PANK2 PE=1 SV=2 | 3045692 |
| Probable RNA-binding protein 19 OS=Homo sapiens OX=9606 GN=RBM19 PE=1 SV=3 | 1240362 |
| Mediator of RNA polymerase II transcription subunit 1 OS=Homo sapiens OX=9606 GN=MED1 PE=1 SV=4 | 1253184 |
| Kinesin-like protein KIF14 OS=Homo sapiens OX=9606 GN=KIF14 PE=1 SV=1 | 696548.6 |
| Nucleolar complex protein 2 homolog OS=Homo sapiens OX=9606 PE=2 SV=1 | 2142099 |
| Complement C3 OS=Homo sapiens OX=9606 GN=C3 PE=1 SV=2 | 1869256 |
| Non-structural maintenance of chromosomes element 4 homolog A OS=Homo sapiens OX=9606 GN=NSMCE4A PE=1 SV=2 | 215050.6 |
| Tight junction protein 2 (Zona occludens 2), isoform CRA_c OS=Homo sapiens OX=9606 GN=TJP2 PE=4 SV=1 | 1109367 |
| Exonuclease 3'-5' domain-containing protein 2 OS=Homo sapiens OX=9606 GN=EXD2 PE=1 SV=2 | 1367218 |
| Eukaryotic translation initiation factor 4E transporter OS=Homo sapiens OX=9606 GN=EIF4ENIF1 PE=1 SV=1 | 1154448 |
| Calcyclin-binding protein OS=Homo sapiens OX=9606 GN=CACYBP PE=1 SV=2 | 525105.2 |
| Nicotinamide/nicotinic acid mononucleotide adenylyltransferase 1 OS=Homo sapiens OX=9606 GN=NMNAT1 PE=1 SV=1 | 2456360 |
| Receptor expression-enhancing protein 4 OS=Homo sapiens OX=9606 GN=REEP4 PE=1 SV=1 | 607620.4 |
| Tudor domain-containing protein 3 OS=Homo sapiens OX=9606 GN=TDRD3 PE=1 SV=1 | 972899.3 |
| NEDD4-like E3 ubiquitin-protein ligase WWP1 OS=Homo sapiens OX=9606 GN=WWP1 PE=1 SV=1 | 2931023 |
| Ribosome biogenesis protein WDR12 OS=Homo sapiens OX=9606 GN=WDR12 PE=1 SV=2 | 1645042 |
| Serrate RNA effector molecule homolog OS=Homo sapiens OX=9606 GN=SRRT PE=1 SV=1 | 7548908 |
| Structural maintenance of chromosomes protein 5 OS=Homo sapiens OX=9606 GN=SMC5 PE=1 SV=2 | 1373476 |
| Far upstream element-binding protein 3 OS=Homo sapiens OX=9606 GN=FUBP3 PE=1 SV=2 | 1492333 |
| DNA excision repair protein ERCC-3 OS=Homo sapiens OX=9606 PE=2 SV=1 | 2824740 |
| Leukocyte receptor cluster member 8 OS=Homo sapiens OX=9606 GN=LENG8 PE=1 SV=1 | 2533446 |
| Liprin-alpha-1 OS=Homo sapiens OX=9606 GN=PPFIA1 PE=1 SV=1 | 1826067 |
| CCAAT/enhancer-binding protein zeta OS=Homo sapiens OX=9606 GN=CEBPZ PE=1 SV=3 | 1176963 |
| Separin OS=Homo sapiens OX=9606 GN=ESPL1 PE=1 SV=3 | 1036605 |
| Fatty acid synthase OS=Homo sapiens OX=9606 GN=FASN PE=1 SV=3 | 546329.7 |
| Cactin OS=Homo sapiens OX=9606 GN=CACTIN PE=1 SV=3 | 1510424 |
| Tight junction protein ZO-1 OS=Homo sapiens OX=9606 GN=TJP1 PE=1 SV=2 | 313654.4 |
| 60S ribosomal protein L14 OS=Homo sapiens OX=9606 PE=2 SV=1 | 3.28E+08 |
| 39S ribosomal protein L55, mitochondrial (Fragment) OS=Homo sapiens OX=9606 GN=MRPL55 PE=1 SV=1 | 2007374 |
| Zinc finger CCHC domain-containing protein 9 OS=Homo sapiens OX=9606 GN=ZCCHC9 PE=1 SV=2 | 1335915 |
| IG c775_heavy_IGHV3-15_IGHD3-10_IGHJ4 (Fragment) OS=Homo sapiens OX=9606 PE=2 SV=1 | 9764246 |
| 5'-3' exoribonuclease 1 OS=Homo sapiens OX=9606 GN=XRN1 PE=1 SV=1 | 5438607 |
| Treacle protein (Fragment) OS=Homo sapiens OX=9606 GN=TCOF1 PE=1 SV=1 | 1129259 |
| Copine-8 OS=Homo sapiens OX=9606 GN=CPNE8 PE=1 SV=2 | 3262682 |
| Valine--tRNA ligase (Fragment) OS=Homo sapiens OX=9606 GN=VARS1 PE=1 SV=1 | 141335.9 |
| Sideroflexin-4 OS=Homo sapiens OX=9606 GN=SFXN4 PE=1 SV=1 | 591309.4 |
| Transformation/transcription domain-associated protein OS=Homo sapiens OX=9606 GN=TRRAP PE=1 SV=2 | 430356.4 |
| Casein kinase I isoform gamma-1 OS=Homo sapiens OX=9606 GN=CSNK1G1 PE=1 SV=1 | 418494.1 |
| 28S ribosomal protein S7, mitochondrial (Fragment) OS=Homo sapiens OX=9606 GN=MRPS7 PE=1 SV=1 | 238007.3 |
| BRI3-binding protein OS=Homo sapiens OX=9606 GN=BRI3BP PE=1 SV=1 | 672531.1 |
| Monocarboxylate transporter 1 (Fragment) OS=Homo sapiens OX=9606 GN=SLC16A1 PE=1 SV=1 | 849830.9 |
| Keratin, type I cytoskeletal 27 OS=Homo sapiens OX=9606 GN=KRT27 PE=1 SV=2 | 598653.1 |
| CWF19L2 protein OS=Homo sapiens OX=9606 GN=CWF19L2 PE=2 SV=1 | 1161696 |
| Uncharacterized protein DKFZp686P23184 OS=Homo sapiens OX=9606 GN=DKFZp686P23184 PE=2 SV=1 |  |
| T-complex protein 1 subunit gamma (Fragment) OS=Homo sapiens OX=9606 PE=2 SV=1 | 936111.8 |
| Protein FAM76B OS=Homo sapiens OX=9606 GN=FAM76B PE=1 SV=3 | 1024872 |
| Citrate synthase (Fragment) OS=Homo sapiens OX=9606 GN=CS PE=2 SV=1 |  |
| 28S ribosomal protein S25, mitochondrial OS=Homo sapiens OX=9606 GN=MRPS25 PE=1 SV=1 | 1652830 |
| E3 ubiquitin-protein ligase RING1 OS=Homo sapiens OX=9606 PE=2 SV=1 | 347998.7 |
| Hepatoma-derived growth factor-related protein 3 OS=Homo sapiens OX=9606 GN=HDGFL3 PE=1 SV=1 | 645056.3 |
| CST complex subunit STN1 OS=Homo sapiens OX=9606 GN=STN1 PE=1 SV=2 | 501711.5 |
| Transcriptional activator protein Pur-beta OS=Homo sapiens OX=9606 GN=PURB PE=1 SV=3 | 1219119 |
| DDRGK domain-containing protein 1 OS=Homo sapiens OX=9606 GN=DDRGK1 PE=1 SV=2 | 459033.8 |
| Solute carrier family 25 member 36 OS=Homo sapiens OX=9606 GN=SLC25A36 PE=1 SV=1 |  |
| FLJ00144 protein (Fragment) OS=Homo sapiens OX=9606 GN=FLJ00144 PE=2 SV=1 | 881403.5 |
| tRNA-uridine aminocarboxypropyltransferase 2 OS=Homo sapiens OX=9606 GN=DTWD2 PE=1 SV=1 | 331878.5 |
| MICOS complex subunit OS=Homo sapiens OX=9606 GN=DKFZp779P1227 PE=2 SV=1 | 1071648 |
| GTP-binding protein Di-Ras2 (Fragment) OS=Homo sapiens OX=9606 GN=DIRAS2 PE=1 SV=1 | 500900.5 |
| Peptidyl-prolyl cis-trans isomerase NIMA-interacting 1 OS=Homo sapiens OX=9606 GN=PIN1 PE=1 SV=1 | 374487.9 |
| 60S ribosomal protein L26-like 1 OS=Homo sapiens OX=9606 GN=RPL26L1 PE=1 SV=1 | 5330821 |
| N-alpha-acetyltransferase 10 OS=Homo sapiens OX=9606 GN=NAA10 PE=1 SV=1 | 436611 |
| Heterogeneous nuclear ribonucleoprotein L (Fragment) OS=Homo sapiens OX=9606 GN=HNRNPL PE=1 SV=1 | 652865.8 |
| General transcription factor 3C polypeptide 2 (Fragment) OS=Homo sapiens OX=9606 GN=GTF3C2 PE=1 SV=8 | 706244.3 |
| IGH c1578_heavy_IGHV5-51_IGHD3-10_IGHJ3 (Fragment) OS=Homo sapiens OX=9606 PE=2 SV=1 | 10727228 |
| HIV Tat-specific factor 1 (Fragment) OS=Homo sapiens OX=9606 GN=HTATSF1 PE=1 SV=1 | 363138.8 |
| Keratin, type II cytoskeletal 7 OS=Homo sapiens OX=9606 GN=KRT7 PE=1 SV=5 | 207222.2 |
| Keratin, type I cytoskeletal 18 OS=Homo sapiens OX=9606 GN=KRT18 PE=1 SV=2 | 9945332 |
| BAG family molecular chaperone regulator 2 OS=Homo sapiens OX=9606 GN=BAG2 PE=1 SV=1 | 1163081 |
| Geranylgeranyl transferase type-1 subunit beta OS=Homo sapiens OX=9606 GN=PGGT1B PE=1 SV=2 |  |
| Galectin-7 OS=Homo sapiens OX=9606 GN=LGALS7 PE=1 SV=2 | 659966.4 |
| Membrane-associated progesterone receptor component 1 OS=Homo sapiens OX=9606 GN=PGRMC1 PE=1 SV=3 | 478701.8 |
| Splicing factor 3B subunit 5 OS=Homo sapiens OX=9606 GN=SF3B5 PE=1 SV=1 |  |
| Peripheral-type benzodiazepine receptor (Fragment) OS=Homo sapiens OX=9606 GN=PBR PE=3 SV=1 | 802068.2 |
| Small proline-rich protein OS=Homo sapiens OX=9606 PE=2 SV=1 | 1275941 |
| DNA-binding protein SMUBP-2 (Fragment) OS=Homo sapiens OX=9606 GN=IGHMBP2 PE=1 SV=1 | 17673516 |
| Small nuclear ribonucleoprotein E OS=Homo sapiens OX=9606 GN=SNRPE PE=1 SV=1 | 3699257 |
| Serine/threonine-protein phosphatase PP1-beta catalytic subunit OS=Homo sapiens OX=9606 GN=PPP1CB PE=1 SV=3 | 1535760 |
| IG c1327_heavy_IGHV3-9_IGHD2-2_IGHJ4 (Fragment) OS=Homo sapiens OX=9606 PE=2 SV=1 | 1.04E+08 |
| Serine/threonine-protein phosphatase PP1-alpha catalytic subunit OS=Homo sapiens OX=9606 GN=PPP1CA PE=1 SV=1 | 3485596 |
| Glyoxylate reductase 1 homolog OS=Homo sapiens OX=9606 GN=N-PAC PE=3 SV=1 | 894117.9 |
| Tumor susceptibility gene 101, isoform CRA_a OS=Homo sapiens OX=9606 GN=TSG101 PE=4 SV=1 | 709478 |
| ABC-type oligopeptide transporter ABCB9 (Fragment) OS=Homo sapiens OX=9606 GN=ABCB9 PE=4 SV=1 | 2248367 |
| Splicing factor 3B subunit 6 OS=Homo sapiens OX=9606 GN=SF3B6 PE=1 SV=1 | 190085.9 |
| cDNA FLJ58272, highly similar to Zinc finger protein 207 OS=Homo sapiens OX=9606 PE=2 SV=1 | 133733.8 |
| cDNA FLJ56640, highly similar to Mus musculus embryonic ectoderm development (Eed), mRNA OS=Homo sapiens OX=9606 PE=2 SV=1 |  |
| Segment polarity protein dishevelled homolog DVL-3 OS=Homo sapiens OX=9606 GN=DVL3 PE=1 SV=1 | 385127.8 |
| cDNA FLJ59402, highly similar to Eukaryotic translation initiation factor 4B OS=Homo sapiens OX=9606 PE=2 SV=1 | 2901728 |
| Exosome complex component RRP46 OS=Homo sapiens OX=9606 GN=EXOSC5 PE=1 SV=1 | 2212024 |
| IGH + IGL c463_heavy_IGHV3-11_IGHD3-3_IGHJ5 (Fragment) OS=Homo sapiens OX=9606 PE=2 SV=1 | 1.28E+08 |
| Upstream binding transcription factor, RNA polymerase I, isoform CRA_a OS=Homo sapiens OX=9606 GN=UBTF PE=2 SV=1 | 1132796 |
| General transcription factor IIF subunit 2 OS=Homo sapiens OX=9606 GN=GTF2F2 PE=1 SV=2 | 788126.7 |
| SND1-BRAF fusion OS=Homo sapiens OX=9606 PE=2 SV=1 | 15101486 |
| Carboxypeptidase OS=Homo sapiens OX=9606 PE=2 SV=1 | 758042.2 |
| Ras-related protein Rab-15 (Fragment) OS=Homo sapiens OX=9606 GN=RAB15 PE=1 SV=1 | 729194.5 |
| Aurora kinase OS=Homo sapiens OX=9606 GN=AURKC PE=1 SV=1 | 557196.9 |
| PX domain-containing protein kinase-like protein (Fragment) OS=Homo sapiens OX=9606 GN=PXK PE=1 SV=1 | 1382438 |
| Epididymis secretory sperm binding protein Li 169mP OS=Homo sapiens OX=9606 GN=HEL-S-169mP PE=2 SV=1 |  |
| Biogenesis of lysosome-related organelles complex 1 subunit 6 OS=Homo sapiens OX=9606 GN=BLOC1S6 PE=1 SV=1 | 6003138 |
| RNA helicase OS=Homo sapiens OX=9606 PE=2 SV=1 | 4847374 |
| SHINC3 OS=Homo sapiens OX=9606 GN=SHINC3 PE=2 SV=1 | 2174742 |
| Protein AATF OS=Homo sapiens OX=9606 GN=AATF PE=1 SV=1 | 1167503 |
| RING-type E3 ubiquitin-protein ligase PPIL2 OS=Homo sapiens OX=9606 GN=PPIL2 PE=1 SV=1 | 463695.7 |
| Glycylpeptide N-tetradecanoyltransferase OS=Homo sapiens OX=9606 GN=NMT1 PE=1 SV=1 | 2323509 |
| Cytochrome b-c1 complex subunit 2, mitochondrial OS=Homo sapiens OX=9606 GN=UQCRC2 PE=1 SV=1 | 245361.5 |
| Protein lin-28 homolog B OS=Homo sapiens OX=9606 GN=LIN28B PE=1 SV=1 | 621856.3 |
| AP-2 complex subunit alpha-2 (Fragment) OS=Homo sapiens OX=9606 GN=AP2A2 PE=1 SV=1 | 10752937 |
| Protein POLR1D, isoform 2 OS=Homo sapiens OX=9606 GN=POLR1D PE=1 SV=1 | 251273.9 |
| LETM1 domain-containing protein 1 (Fragment) OS=Homo sapiens OX=9606 GN=LETMD1 PE=1 SV=1 | 649213.5 |
| Formyltetrahydrofolate synthetase OS=Homo sapiens OX=9606 GN=MTHFD1L PE=1 SV=1 | 3544932 |
| Nucleolar protein 12 OS=Homo sapiens OX=9606 PE=2 SV=1 | 731429.3 |
| Pyruvate kinase OS=Homo sapiens OX=9606 GN=PKM2 PE=3 SV=1 | 2857038 |
| Non-specific serine/threonine protein kinase OS=Homo sapiens OX=9606 GN=MARK3 PE=1 SV=1 | 732405.9 |
| Mediator of RNA polymerase II transcription subunit 4 (Fragment) OS=Homo sapiens OX=9606 GN=MED4 PE=2 SV=1 | 1020399 |
| Spermatid perinuclear RNA-binding protein (Fragment) OS=Homo sapiens OX=9606 GN=STRBP PE=1 SV=1 | 384478.3 |
| FtsH homolog OS=Homo sapiens OX=9606 PE=2 SV=1 | 322561.9 |
| Hydroxysteroid dehydrogenase 10 isoform 2 OS=Homo sapiens OX=9606 GN=HSD17B10 PE=2 SV=1 | 288882.4 |
| Carcinoembryonic antigen-related cell adhesion molecule 21 (Fragment) OS=Homo sapiens OX=9606 GN=CEACAM21 PE=1 SV=1 | 799451.8 |
| Armadillo repeat-containing X-linked protein 3 OS=Homo sapiens OX=9606 GN=ARMCX3 PE=1 SV=1 | 392191.3 |
| Eukaryotic translation initiation factor 3 subunit K OS=Homo sapiens OX=9606 GN=EIF3K PE=1 SV=1 | 2351950 |
| DNA topoisomerase 2-beta OS=Homo sapiens OX=9606 GN=TOP2B PE=1 SV=3 | 911100.1 |
| Low molecular weight cytosolic acid phosphatase (Fragment) OS=Homo sapiens OX=9606 PE=2 SV=1 | 339219.2 |
| ADP-ribosylation factor-like protein 6-interacting protein 4 OS=Homo sapiens OX=9606 GN=ARL6IP4 PE=1 SV=1 | 1886329 |
| Ribonucloprotein (Fragment) OS=Homo sapiens OX=9606 GN=NHP2 PE=1 SV=1 | 1892023 |
| Heterogeneous nuclear ribonucleoprotein A/B OS=Homo sapiens OX=9606 GN=HNRNPAB PE=1 SV=1 | 3113089 |
| Ribosome production factor 1 OS=Homo sapiens OX=9606 GN=RPF1 PE=1 SV=2 | 2942826 |
| Mannosyl-oligosaccharide glucosidase (Fragment) OS=Homo sapiens OX=9606 GN=MOGS PE=1 SV=1 | 360521.3 |
| Profilin OS=Homo sapiens OX=9606 PE=2 SV=1 | 1961680 |
| Chromobox protein homolog 3 OS=Homo sapiens OX=9606 GN=CBX3 PE=1 SV=4 | 488549.8 |
| Histone H2A OS=Homo sapiens OX=9606 GN=MACROH2A1 PE=1 SV=1 | 296225.3 |
| RNA-binding protein 42 OS=Homo sapiens OX=9606 GN=RBM42 PE=1 SV=1 | 1923190 |
| WD repeat domain-containing protein 83 OS=Homo sapiens OX=9606 GN=WDR83 PE=1 SV=1 | 1686933 |
| rRNA-processing protein FCF1 homolog OS=Homo sapiens OX=9606 GN=FCF1 PE=1 SV=1 | 710582.9 |
| GRB2 protein (Fragment) OS=Homo sapiens OX=9606 GN=GRB2 PE=2 SV=1 | 560365.8 |
| Sigma intracellular receptor 2 OS=Homo sapiens OX=9606 GN=TMEM97 PE=1 SV=1 | 334131.9 |
| mRNA (guanine-N(7)-)-methyltransferase (Fragment) OS=Homo sapiens OX=9606 GN=RNMT PE=2 SV=1 | 217202.4 |
| Sorting nexin-27 OS=Homo sapiens OX=9606 GN=SNX27 PE=1 SV=2 | 1495024 |
| Proteasome subunit alpha type (Fragment) OS=Homo sapiens OX=9606 GN=PSMA7 PE=2 SV=1 | 338027.7 |
| Zinc finger C2HC domain-containing protein 1A OS=Homo sapiens OX=9606 GN=ZC2HC1A PE=1 SV=2 | 1586128 |
| Keratin, type II cuticular Hb4 OS=Homo sapiens OX=9606 GN=KRT84 PE=2 SV=2 | 821344.1 |
| Homeobox protein Hox-B9 OS=Homo sapiens OX=9606 GN=HOXB9 PE=1 SV=2 | 388204.8 |
| 60S ribosomal export protein NMD3 OS=Homo sapiens OX=9606 GN=NMD3 PE=1 SV=1 | 1064384 |
| DNA-directed RNA polymerases I and III subunit RPAC1 OS=Homo sapiens OX=9606 GN=POLR1C PE=1 SV=1 |  |
| Zinc finger protein 622 OS=Homo sapiens OX=9606 GN=ZNF622 PE=1 SV=1 | 392940.5 |
| Far upstream element-binding protein 2 OS=Homo sapiens OX=9606 GN=KHSRP PE=1 SV=4 | 2357225 |
| Protein TFG OS=Homo sapiens OX=9606 GN=TFG PE=1 SV=2 | 3726005 |
| Fatty acyl-CoA reductase 1 OS=Homo sapiens OX=9606 GN=FAR1 PE=1 SV=1 |  |
| Translation initiation factor eIF-2B subunit beta (Fragment) OS=Homo sapiens OX=9606 GN=EIF2B2 PE=1 SV=1 | 539517.3 |
| Keratin, type II cytoskeletal 74 OS=Homo sapiens OX=9606 GN=KRT74 PE=1 SV=1 | 11394511 |
| Centrosomal AT-AC-splicing factor OS=Homo sapiens OX=9606 GN=CENATAC PE=1 SV=1 | 461671.9 |
| ATP synthase subunit gamma (Fragment) OS=Homo sapiens OX=9606 PE=2 SV=1 | 931901.2 |
| 39S ribosomal protein L15, mitochondrial OS=Homo sapiens OX=9606 GN=MRPL15 PE=1 SV=1 | 438985.8 |
| Beta-1,4-galactosyltransferase 7 OS=Homo sapiens OX=9606 GN=B4GALT7 PE=1 SV=1 | 196586.9 |
| Transcription factor 25 OS=Homo sapiens OX=9606 GN=TCF25 PE=1 SV=1 | 1319860 |
| Mothers against decapentaplegic homolog 5 OS=Homo sapiens OX=9606 GN=SMAD5 PE=1 SV=1 | 205774 |
| Hornerin OS=Homo sapiens OX=9606 GN=HRNR PE=1 SV=2 | 6839826 |
| Eukaryotic translation initiation factor 4E type 2 (Fragment) OS=Homo sapiens OX=9606 GN=EIF4E2 PE=1 SV=1 | 1240140 |
| UAP56-interacting factor OS=Homo sapiens OX=9606 GN=FYTTD1 PE=1 SV=3 | 4398494 |
| Transcription initiation factor TFIID subunit 8 OS=Homo sapiens OX=9606 GN=TAF8 PE=1 SV=1 | 575103.9 |
| Mitochondrial ribosomal protein L10, isoform CRA_d OS=Homo sapiens OX=9606 GN=MRPL10 PE=2 SV=1 | 1368663 |
| cDNA FLJ39778 fis, clone SPLEN2001710, highly similar to Kelch domain-containing protein 4 OS=Homo sapiens OX=9606 PE=2 SV=1 | 897712.6 |
| Crn-related protein kim1 OS=Homo sapiens OX=9606 PE=2 SV=1 | 1129971 |
| Centromere protein U OS=Homo sapiens OX=9606 GN=MLF1IP PE=2 SV=1 | 849023.8 |
| IGH c236_heavy__IGHV2-5_IGHD3-16_IGHJ3 (Fragment) OS=Homo sapiens OX=9606 PE=2 SV=1 | 3908526 |
| Zinc finger protein 787 OS=Homo sapiens OX=9606 GN=ZNF787 PE=1 SV=4 | 1314528 |
| HCG20693, isoform CRA_a OS=Homo sapiens OX=9606 GN=hCG_20693 PE=4 SV=1 | 529405.3 |
| Very-long-chain (3R)-3-hydroxyacyl-CoA dehydratase 3 OS=Homo sapiens OX=9606 GN=HACD3 PE=1 SV=2 | 19392362 |
| Transmembrane protein 160 OS=Homo sapiens OX=9606 GN=TMEM160 PE=1 SV=1 | 298163.3 |
| Transmembrane protein 208 OS=Homo sapiens OX=9606 GN=TMEM208 PE=1 SV=1 |  |
| Zinc finger CCCH domain-containing protein 13 OS=Homo sapiens OX=9606 GN=ZC3H13 PE=1 SV=1 | 5680480 |
| Methionine aminopeptidase (Fragment) OS=Homo sapiens OX=9606 PE=2 SV=1 | 985318.3 |
| DNA-directed RNA polymerase (Fragment) OS=Homo sapiens OX=9606 PE=2 SV=1 | 4164421 |
| Uncharacterized protein LOC84524 (Fragment) OS=Homo sapiens OX=9606 GN=LOC84524 PE=4 SV=1 | 627357.4 |
| Serine/arginine-rich splicing factor 12 OS=Homo sapiens OX=9606 GN=SRSF12 PE=2 SV=1 | 430331.6 |
| Probable proline--tRNA ligase, mitochondrial OS=Homo sapiens OX=9606 GN=PARS2 PE=1 SV=1 | 1128310 |
| Uncharacterized protein WBSCR22 OS=Homo sapiens OX=9606 GN=WBSCR22 PE=3 SV=1 | 567715.3 |
| Pre-mRNA-splicing factor 38A OS=Homo sapiens OX=9606 GN=PRPF38A PE=1 SV=1 | 366257.4 |
| Mediator of RNA polymerase II transcription subunit 14 (Fragment) OS=Homo sapiens OX=9606 GN=MED14 PE=1 SV=1 | 371190.6 |
| Cancer-related nucleoside-triphosphatase OS=Homo sapiens OX=9606 GN=NTPCR PE=1 SV=1 | 842724.2 |
| Outer mitochondrial membrane protein porin 2 (Fragment) OS=Homo sapiens OX=9606 GN=VDAC2 PE=1 SV=8 | 1999188 |
| ACAD11 protein OS=Homo sapiens OX=9606 GN=ACAD11 PE=2 SV=1 | 401509.2 |
| Protein SET OS=Homo sapiens OX=9606 GN=SET PE=1 SV=3 | 649274.1 |
| WD repeat-containing protein 5 OS=Homo sapiens OX=9606 GN=WDR5 PE=1 SV=1 | 950307.3 |
| 28S ribosomal protein S29, mitochondrial OS=Homo sapiens OX=9606 GN=DAP3 PE=1 SV=1 |  |
| GMP synthase [glutamine-hydrolyzing] OS=Homo sapiens OX=9606 GN=GMPS PE=1 SV=1 | 559574.4 |
| Moesin OS=Homo sapiens OX=9606 GN=MSN PE=1 SV=3 | 4374001 |
| cDNA FLJ51764, highly similar to Histone deacetylase 1 OS=Homo sapiens OX=9606 PE=2 SV=1 | 641571.4 |
| Elongation factor 1-delta OS=Homo sapiens OX=9606 GN=EEF1D PE=1 SV=5 | 542268.1 |
| Pre-mRNA cleavage complex 2 protein Pcf11 OS=Homo sapiens OX=9606 GN=PCF11 PE=1 SV=3 | 2979564 |
| Erlin-1 OS=Homo sapiens OX=9606 GN=ERLIN1 PE=1 SV=2 | 167201.8 |
| Zinc finger C3H1 domain-containing protein OS=Homo sapiens OX=9606 GN=ZFC3H1 PE=1 SV=3 | 5073157 |
| S-adenosylmethionine synthase OS=Homo sapiens OX=9606 GN=MAT2A PE=2 SV=1 | 375239.4 |
| Modulator of non-genomic activity of estrogen receptor (Fragment) OS=Homo sapiens OX=9606 PE=2 SV=2 | 390857.1 |
| Copine-3 OS=Homo sapiens OX=9606 GN=CPNE3 PE=1 SV=1 | 803539.1 |
| E3 SUMO-protein ligase CBX4 OS=Homo sapiens OX=9606 GN=CBX4 PE=1 SV=3 |  |
| 5'-nucleotidase domain-containing protein 2 (Fragment) OS=Homo sapiens OX=9606 GN=NT5DC2 PE=1 SV=1 | 203339.2 |
| tRNA-dihydrouridine(16/17) synthase [NAD(P)(+)]-like (Fragment) OS=Homo sapiens OX=9606 GN=DUS1L PE=1 SV=2 | 228186.2 |
| Polyadenylate-binding protein 2 OS=Homo sapiens OX=9606 GN=PABPN1 PE=1 SV=1 | 1780412 |
| Mitochondrial thiamine pyrophosphate carrier OS=Homo sapiens OX=9606 GN=SLC25A19 PE=1 SV=1 | 282825.2 |
| Transcription initiation factor IIB OS=Homo sapiens OX=9606 GN=GTF2B PE=1 SV=1 | 304894.7 |
| Histone RNA hairpin-binding protein OS=Homo sapiens OX=9606 GN=SLBP PE=1 SV=1 | 531455.3 |
| WD repeat-containing protein 55 OS=Homo sapiens OX=9606 GN=WDR55 PE=1 SV=2 | 929334.8 |
| cDNA FLJ61069, highly similar to N6-adenosine-methyltransferase 70 kDa subunit OS=Homo sapiens OX=9606 PE=2 SV=1 | 487835.6 |
| cDNA FLJ61542, highly similar to Myosin-5C OS=Homo sapiens OX=9606 PE=2 SV=1 | 954629.6 |
| Mitogen-activated protein kinase kinase kinase kinase 4 OS=Homo sapiens OX=9606 GN=MAP4K4 PE=1 SV=1 | 4012137 |
| cDNA FLJ61181, highly similar to Homo sapiens hydroxysteroid (17-beta) dehydrogenase 12 (HSD17B12), mRNA OS=Homo sapiens OX=9606 PE=2 SV=1 | 754196.7 |
| cDNA FLJ56861, highly similar to Dual specificity testis-specific protein kinase 1 OS=Homo sapiens OX=9606 PE=2 SV=1 | 1248378 |
| Coiled-coil domain-containing protein 47 OS=Homo sapiens OX=9606 GN=CCDC47 PE=1 SV=1 | 2520436 |
| Mediator complex subunit 26 OS=Homo sapiens OX=9606 PE=3 SV=2 | 298395.5 |
| Dolichyl-diphosphooligosaccharide--protein glycosyltransferase subunit STT3B OS=Homo sapiens OX=9606 GN=STT3B PE=1 SV=1 |  |
| Nuclear migration protein nudC OS=Homo sapiens OX=9606 GN=NUDC PE=1 SV=1 | 833653.8 |
| Serine/threonine-protein kinase TAO2 OS=Homo sapiens OX=9606 GN=TAOK2 PE=1 SV=2 | 1165422 |
| cDNA FLJ54585 OS=Homo sapiens OX=9606 PE=2 SV=1 | 606510.8 |
| Pre-mRNA-splicing factor CWC22 homolog (Fragment) OS=Homo sapiens OX=9606 GN=CWC22 PE=1 SV=1 | 1492752 |
| cDNA FLJ58218, highly similar to Ankyrin repeat and zinc fingerdomain-containing protein 1 OS=Homo sapiens OX=9606 PE=2 SV=1 | 336412.4 |
| Bleomycin hydrolase OS=Homo sapiens OX=9606 PE=2 SV=1 | 348708.8 |
| Trinucleotide repeat-containing gene 18 protein (Fragment) OS=Homo sapiens OX=9606 GN=TNRC18 PE=1 SV=1 | 5043498 |
| N-alpha-acetyltransferase 40 OS=Homo sapiens OX=9606 GN=NAA40 PE=1 SV=1 | 497094.8 |
| Dolichyl-diphosphooligosaccharide--protein glycosyltransferase subunit 2 OS=Homo sapiens OX=9606 PE=2 SV=1 | 605033.8 |
| 39S ribosomal protein L44, mitochondrial OS=Homo sapiens OX=9606 GN=MRPL44 PE=1 SV=1 | 3675477 |
| Mitochondrial mRNA pseudouridine synthase RPUSD3 OS=Homo sapiens OX=9606 GN=RPUSD3 PE=1 SV=3 | 495380.8 |
| Ubiquitously transcribed tetratricopeptide repeat protein Y-linked transcript variant 153 OS=Homo sapiens OX=9606 GN=UTY PE=2 SV=1 | 12004965 |
| THAP domain-containing protein 11 OS=Homo sapiens OX=9606 GN=THAP11 PE=1 SV=2 | 681344.4 |
| cDNA, FLJ96114, highly similar to Homo sapiens bromodomain and WD repeat domain containing 2 (BRWD2), mRNA OS=Homo sapiens OX=9606 PE=2 SV=1 | 2329121 |
| cDNA, FLJ95468, highly similar to Homo sapiens transcriptional coactivator tubedown-100 (TBDN100),transcript variant 1, mRNA OS=Homo sapiens OX=9606 PE=2 SV=1 | 3071352 |
| cDNA FLJ76187 OS=Homo sapiens OX=9606 PE=2 SV=1 |  |
| PIN2/TERF1-interacting telomerase inhibitor 1 OS=Homo sapiens OX=9606 GN=PINX1 PE=1 SV=2 | 1394505 |
| MTCH1 protein (Fragment) OS=Homo sapiens OX=9606 GN=MTCH1 PE=2 SV=1 | 448285.3 |
| Heterogeneous nuclear ribonucleoprotein L-like OS=Homo sapiens OX=9606 GN=HNRNPLL PE=1 SV=1 | 1558380 |
| Non-specific serine/threonine protein kinase OS=Homo sapiens OX=9606 GN=MARK1 PE=1 SV=1 | 190538.3 |
| Methenyltetrahydrofolate cyclohydrolase OS=Homo sapiens OX=9606 GN=MTHFD2 PE=1 SV=1 | 412657.1 |
| CTP synthase OS=Homo sapiens OX=9606 GN=CTPS1 PE=1 SV=1 | 405611.3 |
| Smad nuclear-interacting protein 1 OS=Homo sapiens OX=9606 GN=SNIP1 PE=1 SV=1 | 2477395 |
| Divergent protein kinase domain 2A OS=Homo sapiens OX=9606 GN=DIPK2A PE=1 SV=1 | 470035.6 |
| Uncharacterized protein C1orf131 OS=Homo sapiens OX=9606 GN=C1orf131 PE=1 SV=4 |  |
| Putative transferase CAF17, mitochondrial OS=Homo sapiens OX=9606 GN=IBA57 PE=1 SV=1 | 901191.3 |
| Putative methyltransferase C9orf114 OS=Homo sapiens OX=9606 GN=SPOUT1 PE=1 SV=3 | 736519.9 |
| Plasminogen OS=Homo sapiens OX=9606 GN=PLG PE=2 SV=1 | 1317452 |
| Adenosylhomocysteinase OS=Homo sapiens OX=9606 GN=AHCY PE=2 SV=1 | 726854.8 |
| Translocating chain-associated membrane protein 1 OS=Homo sapiens OX=9606 GN=TRAM1 PE=1 SV=3 | 1196073 |
| PREB protein OS=Homo sapiens OX=9606 GN=PREB PE=2 SV=1 | 617388.3 |
| Transcriptional activator protein Pur-alpha OS=Homo sapiens OX=9606 GN=PURA PE=1 SV=2 | 1167514 |
| Splicing factor, arginine/serine-rich 14, isoform CRA_a OS=Homo sapiens OX=9606 GN=SFRS14 PE=4 SV=1 | 1476033 |
| 3-beta-hydroxysterol Delta (14)-reductase OS=Homo sapiens OX=9606 GN=LBR PE=3 SV=1 | 6672584 |
| Corneodesmosin OS=Homo sapiens OX=9606 PE=4 SV=1 | 404689.6 |
| ATPase MORC2 OS=Homo sapiens OX=9606 GN=MORC2 PE=1 SV=2 | 712143.2 |
| Ubiquitin carboxyl-terminal hydrolase 16 OS=Homo sapiens OX=9606 GN=USP16 PE=1 SV=1 | 2334843 |
| CD2 antigen cytoplasmic tail-binding protein 2 OS=Homo sapiens OX=9606 GN=CD2BP2 PE=1 SV=1 | 1083287 |
| Testicular tissue protein Li 70 OS=Homo sapiens OX=9606 PE=2 SV=1 | 588637.9 |
| Nuclear fragile X mental retardation-interacting protein 1 OS=Homo sapiens OX=9606 GN=NUFIP1 PE=1 SV=2 |  |
| Uncharacterized protein KIAA1522 OS=Homo sapiens OX=9606 GN=KIAA1522 PE=1 SV=2 | 529259.6 |
| Abl interactor 2 OS=Homo sapiens OX=9606 GN=ABI2 PE=1 SV=1 |  |
| 28S ribosomal protein S9, mitochondrial OS=Homo sapiens OX=9606 GN=MRPS9 PE=1 SV=2 | 1424509 |
| 28S ribosomal protein S5, mitochondrial OS=Homo sapiens OX=9606 GN=MRPS5 PE=1 SV=2 | 419396.7 |
| DDB1- and CUL4-associated factor 7 OS=Homo sapiens OX=9606 GN=DCAF7 PE=1 SV=1 | 513286 |
| Ribonuclease inhibitor OS=Homo sapiens OX=9606 GN=RNH1 PE=1 SV=2 | 201736.7 |
| Pleckstrin homology domain-containing family A member 5 OS=Homo sapiens OX=9606 GN=PLEKHA5 PE=1 SV=1 | 764719 |
| Chromosome 11 open reading frame 57, isoform CRA_a OS=Homo sapiens OX=9606 GN=C11orf57 PE=4 SV=1 | 1275237 |
| Splicing factor, arginine/serine-rich 19 OS=Homo sapiens OX=9606 GN=SCAF1 PE=1 SV=3 | 1095739 |
| Activating signal cointegrator 1 complex subunit 2 OS=Homo sapiens OX=9606 GN=ASCC2 PE=1 SV=3 | 929619.8 |
| Carboxypeptidase A4 OS=Homo sapiens OX=9606 GN=CPA4 PE=1 SV=2 | 365473.7 |
| p40 OS=Homo sapiens OX=9606 PE=4 SV=1 | 1165701 |
| Cell division control protein 6 homolog OS=Homo sapiens OX=9606 GN=CDC6 PE=1 SV=1 | 258957.2 |
| Coiled-coil domain-containing protein 106 OS=Homo sapiens OX=9606 GN=CCDC106 PE=1 SV=1 | 1920571 |
| Holliday junction recognition protein (Fragment) OS=Homo sapiens OX=9606 GN=HJURP PE=1 SV=1 | 735787.4 |
| Serpin B12 OS=Homo sapiens OX=9606 GN=SERPINB12 PE=1 SV=1 | 1333075 |
| Cell division cycle protein 27 homolog (Fragment) OS=Homo sapiens OX=9606 GN=CDC27 PE=1 SV=1 |  |
| T-complex protein 1 subunit alpha OS=Homo sapiens OX=9606 GN=TCP1 PE=1 SV=1 | 825074.2 |
| Leucine-rich repeat-containing protein 40 OS=Homo sapiens OX=9606 GN=LRRC40 PE=1 SV=1 | 146563.2 |
| Ubiquitin carboxyl-terminal hydrolase 7 OS=Homo sapiens OX=9606 GN=USP7 PE=1 SV=2 | 1772263 |
| Polyribonucleotide 5'-hydroxyl-kinase Clp1 OS=Homo sapiens OX=9606 GN=CLP1 PE=1 SV=1 | 1353983 |
| Mitogen-activated protein kinase kinase 7, isoform CRA_b OS=Homo sapiens OX=9606 GN=MAP2K7 PE=4 SV=1 | 319914.8 |
| Ribosomal protein L15 OS=Homo sapiens OX=9606 GN=LOC136321 PE=3 SV=1 | 2166022 |
| Endophilin-A2 OS=Homo sapiens OX=9606 GN=SH3GL1 PE=1 SV=1 | 1001506 |
| Atypical kinase COQ8A, mitochondrial OS=Homo sapiens OX=9606 GN=COQ8A PE=1 SV=1 | 180600.8 |
| Insulin-induced gene protein OS=Homo sapiens OX=9606 GN=INSIG2 PE=1 SV=1 | 17619590 |
| Tripartite motif-containing 3, isoform CRA_f (Fragment) OS=Homo sapiens OX=9606 GN=TRIM3 PE=3 SV=1 | 326188 |
| Putative RNA-binding protein 15B OS=Homo sapiens OX=9606 GN=RBM15B PE=1 SV=3 | 773056.1 |
| Putative inactive cytochrome P450 family member 4Z2 OS=Homo sapiens OX=9606 GN=CYP4Z2P PE=5 SV=2 | 11137522 |
| DNA mismatch repair protein Msh6 OS=Homo sapiens OX=9606 GN=MSH6 PE=1 SV=1 | 400953.9 |
| DNA polymerase delta subunit 3 OS=Homo sapiens OX=9606 PE=2 SV=1 |  |
| cDNA FLJ50387 OS=Homo sapiens OX=9606 PE=2 SV=1 | 889324.6 |
| Corepressor interacting with RBPJ 1 OS=Homo sapiens OX=9606 GN=CIR1 PE=1 SV=1 | 551871.3 |
| Gamma-taxilin OS=Homo sapiens OX=9606 GN=TXLNG PE=1 SV=2 | 1220635 |
| IMP dehydrogenase OS=Homo sapiens OX=9606 PE=2 SV=1 | 238978.8 |
| General transcription factor IIH subunit 4 OS=Homo sapiens OX=9606 PE=2 SV=1 | 508393.1 |
| U3 small nucleolar RNA-associated protein 4 homolog (Fragment) OS=Homo sapiens OX=9606 GN=UTP4 PE=1 SV=1 | 345853.2 |
| Poly(A) RNA polymerase GLD2 OS=Homo sapiens OX=9606 GN=TENT2 PE=1 SV=1 | 133851.4 |
| BRD4 protein (Fragment) OS=Homo sapiens OX=9606 GN=BRD4 PE=2 SV=1 | 1126667 |
| GTP-binding protein 2 OS=Homo sapiens OX=9606 GN=GTPBP2 PE=1 SV=1 | 269944.3 |
| Similar to RP9 protein OS=Homo sapiens OX=9606 GN=LOC402478 PE=4 SV=1 | 1828402 |
| Pre-mRNA-splicing factor CWC25 homolog OS=Homo sapiens OX=9606 GN=CWC25 PE=1 SV=1 | 759942.8 |
| Terminal uridylyltransferase 7 OS=Homo sapiens OX=9606 GN=TUT7 PE=1 SV=1 | 724845.5 |
| B lymphoma Mo-MLV insertion region (Mouse) OS=Homo sapiens OX=9606 PE=2 SV=1 | 392317.4 |
| Pre-mRNA-splicing factor RBM22 OS=Homo sapiens OX=9606 GN=RBM22 PE=1 SV=1 | 3782837 |
| Uncharacterized protein DKFZp779I2251 (Fragment) OS=Homo sapiens OX=9606 GN=DKFZp779I2251 PE=2 SV=1 | 701138.3 |
| Mediator of RNA polymerase II transcription subunit 25 OS=Homo sapiens OX=9606 GN=MED25 PE=1 SV=2 | 149588.6 |
| [Pyruvate dehydrogenase (acetyl-transferring)] kinase isozyme 3, mitochondrial OS=Homo sapiens OX=9606 GN=PDK3 PE=1 SV=1 | 155233.9 |
| Translation initiation factor eIF-2B subunit gamma OS=Homo sapiens OX=9606 GN=EIF2B3 PE=1 SV=1 | 631863.2 |
| Hyaluronan-binding protein 2 OS=Homo sapiens OX=9606 GN=HABP2 PE=1 SV=1 | 1012292 |
| DNA-directed RNA polymerase I subunit RPA2 (Fragment) OS=Homo sapiens OX=9606 GN=POLR1B PE=2 SV=1 |  |
| Flt3-interacting zinc finger protein 1 OS=Homo sapiens OX=9606 GN=FIZ1 PE=1 SV=2 | 144383 |
| Cyclin-L2 OS=Homo sapiens OX=9606 GN=CCNL2 PE=1 SV=1 | 497195.4 |
| G-patch domain and KOW motifs-containing protein OS=Homo sapiens OX=9606 GN=GPKOW PE=1 SV=2 | 808036.9 |
| Transcription initiation factor TFIID subunit 4B OS=Homo sapiens OX=9606 GN=TAF4B PE=1 SV=2 | 148129.3 |
| Histamine receptor H4 OS=Homo sapiens OX=9606 GN=H4 PE=2 SV=1 | 6266899 |
| Ribosome biogenesis protein NOP53 (Fragment) OS=Homo sapiens OX=9606 GN=GLTSCR2 PE=2 SV=2 | 624533.3 |
| Olfactory receptor 6N2 OS=Homo sapiens OX=9606 GN=OR6N2 PE=3 SV=1 | 1877353 |
| Crossover junction endonuclease EME1 OS=Homo sapiens OX=9606 GN=EME1 PE=1 SV=2 | 834187.6 |
| 28S ribosomal protein S31, mitochondrial OS=Homo sapiens OX=9606 GN=MRPS31 PE=1 SV=3 |  |
| UDP-N-acetylglucosamine-2-epimerase / N-acetylmannosamine kinase OS=Homo sapiens OX=9606 GN=GNE PE=2 SV=1 | 174726.2 |
| Regulator of chromosome condensation OS=Homo sapiens OX=9606 GN=RCC1 PE=1 SV=1 | 1316174 |
| Protein kinase C iota type OS=Homo sapiens OX=9606 GN=PRKCI PE=1 SV=2 | 822708.4 |
| Spermatogenesis-associated serine-rich protein 2 OS=Homo sapiens OX=9606 GN=SPATS2 PE=1 SV=1 | 466718.9 |
| Paraneoplastic antigen-like protein 8A OS=Homo sapiens OX=9606 GN=PNMA8A PE=1 SV=2 | 869113.9 |
| BAF53A protein OS=Homo sapiens OX=9606 GN=BAF53A PE=2 SV=1 | 1527045 |
| PC4 and SFRS1-interacting protein OS=Homo sapiens OX=9606 GN=PSIP1 PE=1 SV=1 | 3068602 |
| Exocyst complex component 3 OS=Homo sapiens OX=9606 GN=EXOC3 PE=1 SV=3 | 1695209 |
| Putative GTP-binding protein 6 OS=Homo sapiens OX=9606 GN=GTPBP6 PE=1 SV=4 | 1245007 |
| PAP associated domain containing protein 5 variant (Fragment) OS=Homo sapiens OX=9606 PE=2 SV=1 | 47203660 |
| Fructose-bisphosphate aldolase A OS=Homo sapiens OX=9606 GN=ALDOA PE=1 SV=2 | 470852.3 |
| Uncharacterized protein DKFZp762M013 (Fragment) OS=Homo sapiens OX=9606 GN=DKFZp762M013 PE=2 SV=1 | 853092.6 |
| Pre-mRNA splicing factor 17 variant (Fragment) OS=Homo sapiens OX=9606 PE=2 SV=1 | 1032590 |
| Protein disulfide-isomerase A5 OS=Homo sapiens OX=9606 GN=PDIA5 PE=1 SV=1 | 534812.4 |
| Serine beta-lactamase-like protein LACTB, mitochondrial OS=Homo sapiens OX=9606 GN=LACTB PE=1 SV=2 | 831242.1 |
| RNA polymerase II elongation factor ELL OS=Homo sapiens OX=9606 GN=ELL PE=1 SV=1 | 343989.6 |
| Transcription initiation factor TFIID subunit 6 OS=Homo sapiens OX=9606 GN=TAF6 PE=1 SV=1 | 764607.6 |
| Alpha-actinin-4 OS=Homo sapiens OX=9606 GN=ACTN4 PE=1 SV=2 |  |
| Serpin B4 OS=Homo sapiens OX=9606 GN=SERPINB4 PE=1 SV=2 | 746231.5 |
| Coilin OS=Homo sapiens OX=9606 GN=COIL PE=1 SV=1 | 308712.5 |
| Non-specific serine/threonine protein kinase OS=Homo sapiens OX=9606 PE=2 SV=1 | 193536.4 |
| Chitinase-3-like protein 1 OS=Homo sapiens OX=9606 GN=CHI3L1 PE=1 SV=2 | 40812484 |
| Structural maintenance of chromosomes protein 6 (Fragment) OS=Homo sapiens OX=9606 GN=SMC6 PE=1 SV=1 | 342039.5 |
| Cleavage stimulation factor subunit 2 OS=Homo sapiens OX=9606 GN=CSTF2 PE=1 SV=1 | 395317.9 |
| cDNA FLJ53145, highly similar to Ankycorbin (Fragment) OS=Homo sapiens OX=9606 PE=2 SV=1 | 193718.7 |
| Replication protein A 70 kDa DNA-binding subunit OS=Homo sapiens OX=9606 GN=RPA1 PE=1 SV=2 | 1504138 |
| Guanine nucleotide-binding protein subunit alpha-14 OS=Homo sapiens OX=9606 GN=GNA14 PE=1 SV=1 | 3869042 |
| U3 small nucleolar RNA-interacting protein 2 OS=Homo sapiens OX=9606 GN=RRP9 PE=1 SV=1 | 1113862 |
| Eukaryotic translation initiation factor 4 gamma 3 OS=Homo sapiens OX=9606 GN=EIF4G3 PE=1 SV=2 | 392641.4 |
| cDNA FLJ55372, highly similar to FH1/FH2 domain-containing protein OS=Homo sapiens OX=9606 PE=2 SV=1 | 947204.1 |
| cDNA FLJ16485 fis, clone BRTHA3004307, highly similar to Dual specificity protein kinase CLK3 OS=Homo sapiens OX=9606 PE=2 SV=1 | 652319.6 |
| TAF5-like RNA polymerase II p300/CBP-associated factor-associated factor 65 kDa subunit 5L OS=Homo sapiens OX=9606 GN=TAF5L PE=1 SV=1 | 566914.9 |
| Muscleblind-like 2 (Drosophila), isoform CRA_a OS=Homo sapiens OX=9606 GN=MBNL2 PE=2 SV=1 | 1873604 |
| Mediator of RNA polymerase II transcription subunit 16 (Fragment) OS=Homo sapiens OX=9606 GN=MED16 PE=1 SV=1 | 414904.9 |
| Brain-specific angiogenesis inhibitor 1-associated protein 2 OS=Homo sapiens OX=9606 PE=2 SV=1 | 982219.4 |
| Very-long-chain enoyl-CoA reductase OS=Homo sapiens OX=9606 GN=TECR PE=1 SV=1 | 1656764 |
| ATIII-T1 OS=Homo sapiens OX=9606 PE=3 SV=1 | 1633242 |
| cDNA FLJ38538 fis, clone HCHON2001407, highly similar to LanC-like protein 2 OS=Homo sapiens OX=9606 PE=2 SV=1 | 581689.2 |
| Complement subcomponent C1r OS=Homo sapiens OX=9606 GN=C1R PE=4 SV=1 | 978923.4 |
| Testis tissue sperm-binding protein Li 90mP OS=Homo sapiens OX=9606 PE=2 SV=1 | 223979 |
| MAJIN OS=Homo sapiens OX=9606 GN=MAJIN PE=2 SV=1 | 83803016 |
| Bromodomain-containing protein 2 (Fragment) OS=Homo sapiens OX=9606 GN=BRD2 PE=1 SV=1 | 724996.6 |
| Endoplasmin OS=Homo sapiens OX=9606 GN=HSP90B1 PE=1 SV=1 | 258828 |
| WD repeat-containing protein 6 OS=Homo sapiens OX=9606 GN=WDR6 PE=1 SV=1 | 3432056 |
| FAM193_C domain-containing protein OS=Homo sapiens OX=9606 GN=FLJ10404 PE=3 SV=1 | 597076.3 |
| cDNA FLJ56210, highly similar to Zinc phosphodiesterase ELAC protein 2 OS=Homo sapiens OX=9606 PE=2 SV=1 | 125520.2 |
| TOX high mobility group box family member 4 OS=Homo sapiens OX=9606 GN=TOX4 PE=1 SV=1 |  |
| Asteroid homolog 1 (Drosophila), isoform CRA_c OS=Homo sapiens OX=9606 GN=ASTE1 PE=1 SV=1 | 315715.5 |
| Histidine--tRNA ligase OS=Homo sapiens OX=9606 PE=2 SV=1 | 1529309 |
| Translation initiation factor eIF-2B subunit epsilon (Fragment) OS=Homo sapiens OX=9606 GN=EIF2B5 PE=1 SV=1 | 275613 |
| Forkhead box C1 OS=Homo sapiens OX=9606 GN=FOXC1 PE=4 SV=1 | 443295.4 |
| AP-2 complex subunit mu OS=Homo sapiens OX=9606 PE=2 SV=1 | 1494000 |
| Programmed cell death protein 4 OS=Homo sapiens OX=9606 PE=2 SV=1 | 319585.2 |
| Cellular tumor antigen p53 OS=Homo sapiens OX=9606 GN=TP53 PE=2 SV=1 | 385586.5 |
| Similar to Importin alpha-2 subunit (Karyopherin alpha-2 subunit) (SRP1-alpha) (RAG cohort protein 1) OS=Homo sapiens OX=9606 GN=LOC340312 PE=3 SV=1 | 2343023 |
| AF4/FMR2 family member 4 OS=Homo sapiens OX=9606 GN=AFF4 PE=1 SV=1 | 410451.2 |
| Protein pelota homolog OS=Homo sapiens OX=9606 GN=hCG_2002731 PE=3 SV=1 | 764802 |
| Coiled-coil domain-containing protein 55 OS=Homo sapiens OX=9606 GN=NSRP1 PE=1 SV=1 | 697441.8 |
| Ca++-dependent secretion activator 2 isoform C (Fragment) OS=Homo sapiens OX=9606 GN=CADPS2 PE=2 SV=1 | 274407 |
| Adenosylhomocysteinase OS=Homo sapiens OX=9606 GN=FGFR2-AHCYL1 PE=2 SV=1 | 538186.4 |
| AFG3-like protein 2 OS=Homo sapiens OX=9606 GN=AFG3L2 PE=1 SV=2 | 262103.4 |
| Exosome complex exonuclease RRP44 OS=Homo sapiens OX=9606 GN=DIS3 PE=1 SV=2 | 733946.7 |
| Death-inducer obliterator 1 OS=Homo sapiens OX=9606 GN=DIDO1 PE=1 SV=5 | 2469859 |
| Cytosolic carboxypeptidase 1 OS=Homo sapiens OX=9606 GN=AGTPBP1 PE=1 SV=3 |  |
| Rac GTPase-activating protein 1 OS=Homo sapiens OX=9606 GN=RACGAP1 PE=1 SV=1 | 233690.1 |
| Transcription termination factor 2 OS=Homo sapiens OX=9606 GN=TTF2 PE=1 SV=2 | 786225.8 |
| Mediator of RNA polymerase II transcription subunit 23 OS=Homo sapiens OX=9606 GN=MED23 PE=1 SV=2 |  |
| E3 ISG15--protein ligase HERC5 OS=Homo sapiens OX=9606 GN=HERC5 PE=1 SV=2 | 1348982 |
| Zinc finger CCCH domain-containing protein 7B OS=Homo sapiens OX=9606 GN=ZC3H7B PE=1 SV=2 |  |
| WD repeat-containing protein 48 OS=Homo sapiens OX=9606 GN=WDR48 PE=1 SV=1 | 417772 |
| Zinc finger protein 629 OS=Homo sapiens OX=9606 GN=ZNF629 PE=1 SV=2 | 379637.1 |
| Cap-specific mRNA (nucleoside-2'-O-)-methyltransferase 1 OS=Homo sapiens OX=9606 GN=CMTR1 PE=1 SV=1 | 871755.4 |
| Regulatory-associated protein of mTOR OS=Homo sapiens OX=9606 GN=RPTOR PE=1 SV=1 | 226768.4 |
| Calmodulin-regulated spectrin-associated protein 3 OS=Homo sapiens OX=9606 GN=CAMSAP3 PE=1 SV=2 | 301490.1 |
| Structural maintenance of chromosomes protein 4 OS=Homo sapiens OX=9606 GN=SMC4 PE=1 SV=2 | 667014.1 |
| Serine/threonine-protein kinase TAO1 OS=Homo sapiens OX=9606 GN=TAOK1 PE=1 SV=1 | 265269.1 |
| RING finger protein unkempt homolog OS=Homo sapiens OX=9606 GN=UNK PE=1 SV=2 | 739352.5 |
| Kelch-like protein 35 OS=Homo sapiens OX=9606 GN=KLHL35 PE=1 SV=3 | 706669.3 |
| RPA-related protein RADX OS=Homo sapiens OX=9606 GN=RADX PE=1 SV=2 | 8316501 |
| ATP-dependent RNA helicase DDX42 OS=Homo sapiens OX=9606 GN=DDX42 PE=1 SV=1 | 220671 |
| Exportin-6 OS=Homo sapiens OX=9606 GN=XPO6 PE=1 SV=1 | 1300291 |
| Protein SCAF11 OS=Homo sapiens OX=9606 GN=SCAF11 PE=1 SV=2 | 477203.4 |
| IQ motif containing GTPase activating protein 2 variant (Fragment) OS=Homo sapiens OX=9606 PE=2 SV=1 | 259085.5 |
| PHD finger protein 3 OS=Homo sapiens OX=9606 GN=PHF3 PE=1 SV=3 | 773083.1 |
| Coiled-coil domain-containing protein 141 OS=Homo sapiens OX=9606 GN=CCDC141 PE=1 SV=2 | 818309.8 |
| Kinesin family member 1Bbeta isoform II OS=Homo sapiens OX=9606 GN=KIF1B PE=1 SV=1 | 486011.1 |
| Plakophilin-4 OS=Homo sapiens OX=9606 GN=PKP4 PE=1 SV=2 | 239988.5 |
| RAD50 protein (Fragment) OS=Homo sapiens OX=9606 GN=RAD50 PE=2 SV=1 | 792089.5 |
| Filaggrin-2 OS=Homo sapiens OX=9606 GN=FLG2 PE=1 SV=1 | 2206223 |
| SEC14-like protein 1 OS=Homo sapiens OX=9606 GN=SEC14L1 PE=1 SV=2 | 1340264 |
| Importin subunit beta-1 OS=Homo sapiens OX=9606 GN=KPNB1 PE=1 SV=2 | 665744.9 |
| Oxidation resistance protein 1 OS=Homo sapiens OX=9606 GN=OXR1 PE=1 SV=2 | 1440790 |
| HEAT repeat-containing protein 3 OS=Homo sapiens OX=9606 GN=HEATR3 PE=1 SV=2 | 664220.3 |
| Adducin 3 isoform a variant (Fragment) OS=Homo sapiens OX=9606 PE=2 SV=1 | 525136.6 |
| Tyrosine-protein phosphatase non-receptor type 13 OS=Homo sapiens OX=9606 GN=PTPN13 PE=1 SV=2 |  |
| Serine/threonine-protein kinase MRCK alpha OS=Homo sapiens OX=9606 GN=CDC42BPA PE=1 SV=1 | 119703.7 |
| Uncharacterized protein EPAS1 (Fragment) OS=Homo sapiens OX=9606 GN=EPAS1 PE=4 SV=1 | 60181.62 |
| ADD2 protein (Fragment) OS=Homo sapiens OX=9606 GN=ADD2 PE=2 SV=1 | 1487072 |
| Selenocysteine-specific elongation factor OS=Homo sapiens OX=9606 GN=EEFSEC PE=1 SV=4 | 380635.5 |
| Kinesin-like protein (Fragment) OS=Homo sapiens OX=9606 GN=KIF18A PE=2 SV=1 | 2359626 |
| Calmodulin-regulated spectrin-associated protein 2 OS=Homo sapiens OX=9606 GN=CAMSAP2 PE=1 SV=3 | 408684.9 |
| Plastin-1 OS=Homo sapiens OX=9606 GN=PLS1 PE=1 SV=2 | 504649.9 |
| Unhealthy ribosome biogenesis protein 2 homolog OS=Homo sapiens OX=9606 GN=URB2 PE=1 SV=2 | 180502.8 |
| Chromodomain-helicase-DNA-binding protein 3 OS=Homo sapiens OX=9606 GN=CHD3 PE=1 SV=3 |  |
| ATP-citrate synthase OS=Homo sapiens OX=9606 GN=ACLY PE=1 SV=3 | 360634.8 |
| Mannan-binding lectin serine protease 1 OS=Homo sapiens OX=9606 GN=MASP1 PE=1 SV=3 | 1234225 |
| E3 UFM1-protein ligase 1 OS=Homo sapiens OX=9606 GN=UFL1 PE=1 SV=2 | 803813.9 |
| Transcription activator BRG1 OS=Homo sapiens OX=9606 GN=SMARCA4 PE=1 SV=2 | 347201.3 |
| Atrial natriuretic peptide receptor 2 OS=Homo sapiens OX=9606 GN=NPR2 PE=1 SV=1 | 506397.7 |
| Unconventional myosin-IXb OS=Homo sapiens OX=9606 GN=MYO9B PE=1 SV=1 | 723899.9 |
| Zinc finger and BTB domain-containing protein 11 OS=Homo sapiens OX=9606 GN=ZBTB11 PE=1 SV=2 |  |
| RNA helicase aquarius OS=Homo sapiens OX=9606 GN=AQR PE=1 SV=4 | 337527.1 |
| Symplekin OS=Homo sapiens OX=9606 GN=SYMPK PE=1 SV=1 | 515961.8 |
| YTH domain-containing protein 1 OS=Homo sapiens OX=9606 GN=YTHDC1 PE=1 SV=1 | 777320.4 |
| Coatomer subunit beta' OS=Homo sapiens OX=9606 GN=COPB2 PE=1 SV=2 |  |
| P-type Ca(2+) transporter (Fragment) OS=Homo sapiens OX=9606 GN=ATP2A2 PE=1 SV=1 | 302653.2 |
| Pumilio homolog 1 (Fragment) OS=Homo sapiens OX=9606 GN=PUM1 PE=1 SV=1 | 1346160 |
| Mediator complex subunit 24 OS=Homo sapiens OX=9606 GN=MED24 PE=1 SV=2 | 256416.1 |
| MICOS complex subunit MIC60 (Fragment) OS=Homo sapiens OX=9606 GN=IMMT PE=1 SV=1 | 460418.3 |
| Protein SMG7 OS=Homo sapiens OX=9606 GN=SMG7 PE=1 SV=1 |  |
| Adhesion G protein-coupled receptor L3 OS=Homo sapiens OX=9606 GN=ADGRL3 PE=1 SV=1 | 403393.8 |
| C-1-tetrahydrofolate synthase, cytoplasmic OS=Homo sapiens OX=9606 PE=2 SV=1 | 242846.1 |
| Exocyst complex component Sec8 OS=Homo sapiens OX=9606 PE=2 SV=1 |  |
| cDNA FLJ59523, highly similar to Scaffold attachment factor B OS=Homo sapiens OX=9606 PE=2 SV=1 | 1414193 |
| Catenin delta-1 OS=Homo sapiens OX=9606 GN=CTNND1 PE=1 SV=2 | 266046.6 |
| cDNA FLJ58490, highly similar to Homo sapiens CCR4-NOT transcription complex, subunit 1 (CNOT1), transcript variant 1, mRNA OS=Homo sapiens OX=9606 PE=2 SV=1 | 191753 |
| cDNA FLJ56236, highly similar to Exportin-2 OS=Homo sapiens OX=9606 PE=2 SV=1 | 220697.7 |
| cDNA FLJ58223, highly similar to Ubiquitin ligase protein DZIP3 OS=Homo sapiens OX=9606 PE=2 SV=1 | 584166.3 |
| cDNA FLJ53353, highly similar to ATP-binding cassette sub-family D member 3 OS=Homo sapiens OX=9606 PE=2 SV=1 | 576716.1 |
| Enoyl-CoA hydratase OS=Homo sapiens OX=9606 PE=2 SV=1 | 1483689 |
| Polypeptide N-acetylgalactosaminyltransferase OS=Homo sapiens OX=9606 PE=2 SV=1 | 356106.2 |
| cDNA FLJ16136 fis, clone BRACE3000520, highly similar to Mus musculus steroid receptor-interacting SNF2 domain protein OS=Homo sapiens OX=9606 PE=2 SV=1 |  |
| cDNA FLJ61235, weakly similar to WD repeat protein YBL104C OS=Homo sapiens OX=9606 PE=2 SV=1 | 8841887 |
| cDNA FLJ77630, highly similar to Homo sapiens BPY2 interacting protein 1, mRNA OS=Homo sapiens OX=9606 PE=1 SV=1 | 471008 |
| cDNA, FLJ93435, highly similar to Homo sapiens general transcription factor IIH, polypeptide 1 (62kD subunit) (GTF2H1), mRNA OS=Homo sapiens OX=9606 PE=2 SV=1 | 549609.9 |
| N-alpha-acetyltransferase 35, NatC auxiliary subunit OS=Homo sapiens OX=9606 PE=2 SV=1 |  |
| cDNA FLJ78675, highly similar to Homo sapiens stroma HMG-box containing protein (HBP2) mRNA OS=Homo sapiens OX=9606 PE=2 SV=1 | 1147841 |
| Phosphatidylinositol 4-phosphate 5-kinase type-1 alpha OS=Homo sapiens OX=9606 GN=PIP5K1A PE=1 SV=2 | 1743298 |
| Nucleoporin GLE1 OS=Homo sapiens OX=9606 GN=GLE1 PE=4 SV=1 | 449446.8 |
| Leucine-rich PPR motif-containing protein, mitochondrial OS=Homo sapiens OX=9606 GN=LRPPRC PE=4 SV=1 | 155130.9 |
| Exportin-1 OS=Homo sapiens OX=9606 GN=XPO1 PE=1 SV=1 | 265377.3 |
| E3 ubiquitin-protein ligase TRIP12 OS=Homo sapiens OX=9606 GN=TRIP12 PE=1 SV=1 | 172159.4 |
| Complex I assembly factor ACAD9, mitochondrial OS=Homo sapiens OX=9606 GN=ACAD9 PE=1 SV=1 | 175461.6 |
| General transcription factor II-I repeat domain-containing protein 2B OS=Homo sapiens OX=9606 GN=GTF2IRD2B PE=4 SV=1 | 463711.1 |
| Coatomer subunit alpha (Fragment) OS=Homo sapiens OX=9606 GN=COPA PE=1 SV=1 | 186120.7 |
| Patatin-like phospholipase domain-containing protein 6 OS=Homo sapiens OX=9606 GN=PNPLA6 PE=1 SV=1 |  |
| Lactotransferrin OS=Homo sapiens OX=9606 GN=LTF PE=2 SV=1 | 697928.9 |
| Cullin-associated NEDD8-dissociated protein 1 (Fragment) OS=Homo sapiens OX=9606 GN=CAND1 PE=1 SV=1 | 173593.5 |
| Cell division cycle and apoptosis regulator protein 1 (Fragment) OS=Homo sapiens OX=9606 GN=CCAR1 PE=1 SV=1 | 384673.4 |
| Terminal uridylyltransferase 4 OS=Homo sapiens OX=9606 GN=TUT4 PE=1 SV=1 | 106683.1 |
| Nucleolar and coiled-body phosphoprotein 1 (Fragment) OS=Homo sapiens OX=9606 GN=NOLC1 PE=1 SV=1 | 729632.1 |
| Kanadaptin OS=Homo sapiens OX=9606 GN=SLC4A1AP PE=1 SV=1 | 644601.8 |
| Cat eye syndrome critical region protein 2 OS=Homo sapiens OX=9606 GN=CECR2 PE=1 SV=1 | 1757080 |
| Ribonuclease III, nuclear, isoform CRA_a OS=Homo sapiens OX=9606 GN=RNASEN PE=3 SV=1 | 750568.6 |
| Protein unc-13 homolog C OS=Homo sapiens OX=9606 GN=UNC13C PE=2 SV=3 | 7412038 |
| Zinc finger protein 318 OS=Homo sapiens OX=9606 GN=ZNF318 PE=1 SV=2 | 304696.9 |
| CAD protein OS=Homo sapiens OX=9606 GN=CAD PE=1 SV=3 | 691841.1 |
| Chromodomain-helicase-DNA-binding protein 2 OS=Homo sapiens OX=9606 GN=CHD2 PE=1 SV=2 | 109317.6 |
| Chromodomain-helicase-DNA-binding protein 1 OS=Homo sapiens OX=9606 GN=CHD1 PE=1 SV=2 | 659543.9 |
| Truncated profilaggrin OS=Homo sapiens OX=9606 GN=FLG PE=4 SV=1 | 946698.2 |
| Microtubule-associated protein 1B, isoform CRA_b OS=Homo sapiens OX=9606 GN=MAP1B PE=4 SV=1 | 308255.4 |

**Supplementary Table S5**

1. **Primers sequence used in this study.**

| Gene Name | Direction | Primer Sequence (5’-3’) |
| --- | --- | --- |
| RAC1 | Forward | ATGTCCGTGCAAAGTGGTATC |
|  | Reverse | CTCGGATCGCTTCGTCAAACA |
| PAK4 | Forward | GGACATCAAGAGCGACTCGAT |
|  | Reverse | CGACCAGCGACTTCCTTCG |
| β-actin | Forward | TCCTCTCCCAAGTCCACACA |
|  | Reverse | GCACGAAGGCTCATCATTCA |

| \| 1. **siRNA**  \| **Sequence for siRNA** \| **Sense (5’-3’)** \| \| --- \| --- \| \| RAC1-siRNA1 \| ACGGTTAATTTCTGTCAAACA \| \| RAC1-siRNA2 \| GGGCGTTGAGTCCATATTTAA \| \| PAK4-siRNA1 \| AATTGGATCCATGTTTGGGAAGAGGAAGAAGC \| \| PAK4-siRNA2 \| AATTGCGGCCGCTTACTTGTCATCGTCGTCCTTGTAGTCTCTGGTGCGGTTCTGGCGCA \| \| Si-Control (NC) \| UUCUCCGAACGUGUCACGUTT \| \|  \| \| --- \| --- \| --- \| --- \| --- \| --- \| --- \| --- \| --- \| --- \| --- \| --- \| --- \| --- \|  1. **Plasmid vector**  \| **Vector/plasmid** \|  \| \| --- \| --- \| \| **RAC1 vector** \| PGMLV-CMV-MCS-Flag-PGK-Puro \| \| **RAC11 plasmid** \| PGMLV-CMV-H_RAC11-Flag-PGK-Puro \| \| **PAK4 vector** \| PGMLV-CMV-MCS-Flag-PGK-Puro \| \| **PAK4 plasmid** \| PGMLV-CMV-H_PAK4-Flag-PGK-Puro \|   **Supplementary Table S6. Antibodies used in this study.**   \| Name \| Company \| Catalog Number \| Assay \| \| --- \| --- \| --- \| --- \| \| RAC1 \| Abcam \| Ab155938 \| WB, IHC \| \| Cleaved Caspase-3 \| Abcam \| Ab32042 \| WB, IHC \| \| Cleaved Caspase-1 \| Cell Signaling Technology \| 89332 \| WB, IP \| \| GSDMD-N \| Abcam \| Ab215203 \| WB \| \| A-RAF \| Abcam \| Ab200653 \| WB, IF, IHC \| \| RAS \| Abcam \| Ab52939 \| WB, IHC, IP \| \| MAPK \| Abcam \| Ab308333 \| WB \| \| C-jun \| Abcam \| Ab40766 \| WB, IHC, IF \| \| PAK4 \| Abcam \| Ab300505 \| WB, IF \| \| β-actin \| Abcam \| Ab8226 \| WB, IHC, IF IP \| \| Flag \| Proteintech \| 20543-1-AP \| WB, IP, Co-IP \| \| IgG \| Santa Cruz \| sc-51993 \| WB, IP \| |
| --- | --- | --- | --- | --- | --- | --- | --- | --- | --- | --- | --- | --- | --- | --- | --- | --- | --- | --- | --- | --- | --- | --- | --- | --- | --- | --- | --- | --- | --- | --- | --- | --- | --- | --- | --- | --- | --- | --- | --- | --- | --- | --- | --- | --- | --- | --- | --- | --- | --- | --- | --- | --- | --- | --- | --- | --- | --- | --- | --- | --- | --- | --- | --- | --- | --- | --- | --- | --- | --- | --- | --- | --- | --- | --- | --- | --- |
